# Identification of Neuropeptides and Their Receptors in the Ectoparasitoid, *Habrobracon hebetor*

**DOI:** 10.3389/fphys.2020.575655

**Published:** 2020-10-16

**Authors:** Kaili Yu, Shijiao Xiong, Gang Xu, Xinhai Ye, Hongwei Yao, Fang Wang, Qi Fang, Qisheng Song, Gongyin Ye

**Affiliations:** ^1^State Key Laboratory of Rice Biology and Key Laboratory of Agricultural Entomology of Ministry of Agriculture, Institute of Insect Sciences, Zhejiang University, Hangzhou, China; ^2^College of Horticulture and Plant Protection, Yangzhou University, Yangzhou, China; ^3^Division of Plant Sciences, College of Agriculture, Food and Natural Resources, University of Missouri, Columbia, MO, United States

**Keywords:** parasitic wasp, neuropeptides, neuropeptide receptors, expression profiles, transcriptome, genome

## Abstract

Neuropeptides are a group of signal molecules that regulate many physiological and behavioral processes by binding to corresponding receptors, most of which are G-protein-coupled receptors (GPCRs). Using bioinformatic methods, we screened genomic and transcriptomic data of the ectoparasitoid wasp, *Habrobracon hebetor*, and annotated 34 neuropeptide candidate precursor genes and 44 neuropeptide receptor candidate genes. The candidate neuropeptide genes were found to encode all known insect neuropeptides except allatotropin, neuropeptide F, pigment dispersing factor, and CCHamides. When compared with the endoparasitic wasp *Pteromalus puparum* and the ectoparasitic wasp *Nasonia vitripennis*, trissin and FMRFamide were found only in *H. hebetor*. A similar result held for the neuropeptide receptor genes, for the receptors were found in *H. hebetor* except the receptors of CCHamides and neuroparsin. Furthermore, we compared and analyzed the differences in neuropeptides in eight Braconidae wasps and identified natalisin in *H. hebetor, Diachasma alloeum, Fopius arisanus* and *Microplitis demolitor*, but not in the other wasps. We also analyzed the transcriptome data and qRT-PCR data from different developmental stages and tissues to reveal the expression patterns of the neuropeptides and their receptors. In this study, we revealed composition of neuropeptides and neuropeptide receptors in *H. hebetor*, which may contribute to future neurobiological studies.

## Introduction

Neuropeptides and peptide hormones function as key signals in orchestrating the regulation of numerous physiological processes and behaviors (Schmitt et al., 2015; Wang L. et al., 2018). Neuropeptides constitute a large and diverse class of signaling molecules that are produced by various types of neurons, neurosecretory cells, endocrine cells and other cells (Nassel et al., 2019). Being a kind of endogenous active substance, neuropeptides widely occur in multicellular biological nerve tissues, where they are involved in the functioning of the nervous system, mostly acting upon neuropeptide receptors (Schoofs et al., 2017).

In insects, neuropeptides and their receptors also play significant roles in controlling myriad physiological processes, including development, reproduction, feeding, homeostasis, courtship, circadian rhythm, olfaction, movement, water, and ion homeostasis, among others (Schoofs et al., 2017; Dickinson et al., 2019; Pandit et al., 2019). Due to their specificity and high activity at low concentrations, neuropeptides have also been investigated as potential leads for developing new environmentally-friendly pest control agents (Xiong et al., 2019). Importantly, many neuropeptides are capable of pleiotropic actions such as enabling them to function as neuromodulators, co-transmitters or circulating hormones, some of effects may be exerted simultaneously (Nassel et al., 2019). Neuropeptides are produced from larger precursor proteins called pre-propeptides. A given insect species can harbor 50 neuropeptide precursor-encoding genes, with some species featuring greater complementarity of precursors than others (Yeoh et al., 2017).

Neuropeptides mediate their biological actions via interactions with specific receptors on the cell surface (Ma et al., 2017). Most of these receptors are G-protein-coupled receptors (GPCRs), which have a similar structure characterized by seven transmembrane domains that are highly conserved through evolution, and they constitute the largest superfamily of cell membrane-spanning proteins (Jastrzebska, 2017). Functionally, these proteins are able to recognize extracellular transmitter molecules (Bao et al., 2018).

*Habrobracon hebetor* is an ectoparasitoid distributed worldwide, with an extensive host range and a rapid life cycle (Ghimire and Phillips, 2010). It provides excellent biological control services in terrestrial agroecosystems and has become a specific agent for effectively managing multiple lepidopteran pests (Ghimire and Phillips, 2010). For example, *H. hebetor* is the natural enemy of *Heliocheilus albipunctella*, parasitizing the latter's larvae at a rate of 50–78% when released in pearl millet fields (Gahukar and Ba, 2019). With the development of next-generation sequencing technology, here we identified a complete set of neuropeptide genes and interrelated receptor genes via genomic and transcriptomic sequencing of *H. hebetor*. Building on this, we then compared them with those annotated in representative species of different insect orders, including the wasp parasitoids *Nasonia vitripennis* (Hauser et al., 2010) and *Pteromalus puparum* (Xu et al., 2020), the bee *Apis mellifera* (Hummon et al., 2006), the fly *Drosophila melanogaster* (Broeck, 2001), the moth *Bombyx mori* (Roller et al., 2008), the beetle *Tribolium castaneum* (Li et al., 2008), and an hemipteran insect: *Nilaparvata lugens* (Tanaka et al., 2014). In this study, we identified 34 neuropeptide candidate genes and 44 neuropeptide candidate receptor genes in *H. hebetor*. The expression profiles of neuropeptides and neuropeptide receptors at different developmental stages and various tissues were determined. These results allow us to compare the neuropeptidergic signaling systems across different insect species, to provide relevant information for further functional studies in *H. hebetor*. The findings also provide us novel practical insights for developing new, environmentally friendly insecticides with high effectiveness to against insect pests but special while remaining safety to for their specific parasitoid wasps.

## Materials and Methods

### Insects Rearing

In the laboratory, *H. hebetor* and one of its major host *Plodia interpunctella* (Hübner) were reared at 27 ± 1°C and 75% relative humidity (RH), under a 14-h: 10-h (light: dark) photoperiod (Phillips and Strand, 1994).

### Sample Collection and Sequencing

In a recent study, we carrier out the sequencing and assembly of the *H. hebetor* genomem, its assembled genome is 131.6 Mb in size with a contig N50 of 1.63 Mb (Ye et al., 2020). Here, we prepared embryos, larvae, and other samples from the wasps for RNA-Seq analysis. Embryos spawned by mating female wasps were obtained from the newly parasitized fifth-instar larvae of *P. interpunctella* within 2 h. Larvae spawned by mating female wasps were isolated from parasitized host larvae at the third instar. White pupae and newly emerged wasp adults of both sexes were collected. Moreover, we also prepared samples of salivary glands, venom glands, the carcass without salivary glands of larvae, and the carcass without the venom gland of female adults. All samples were collected, washed with phosphate buffer saline (10 mM of 1 × PBS; pH 7.4), and then ground with TRIzol reagent (Invitrogen, USA) for subsequent experiments. The construction of complementary DNA (cDNA) libraries was performed by Nextomics Biosciences (Nextomics, Wuhan, China), and then sequenced using an Illumina HiSeq^TM^ system (Illumina, NEB, United States). We sequenced two 100-bp paired-end lanes. We used the NGSQCToolKit (v2.3.3) for data filtering (Patel and Jain, 2012), and the FastQC (http://www.bioinformatics.babraham.ac.uk/projects/fastqc/) for quality control of data. The Q30 of all the samples were above 94%, and we then generated ~144.3 million clean reads, for a total of 21556.9 MB for all sequences. The two un-filtered paired-end lanes of each sequence have been deposited as a series, under the accession number PRJNA642006 at NCBI's GEO database or at the NCBI Short Read Archive under submission number SUB7674593.

### Identification of Neuropeptides and Neuropeptide Receptors

We used the already-reported neuropeptides and neuropeptide receptors of *P. puparum, N. vitripennis, D. melanogaster, A. mellifera, B. mori*, and other insects as queries to search for candidate sequences in *H. hebetor* genome database. TBLASTN were used to search for and acquire candidate sequences with the criteria of identity ≥30% and a threshold *E*-value of 10^−3^. We amplified 5000-bp upstream and downstream of the corresponding genome regions into high scoring segment pairs (HSPs). Gene predictions from the HSPs were carried out using the GeneWise tools (Birney and Durbin, 2000), and then confirmed through a BLASTP search with the non-redundant protein sequence (NR) at NCBI. After gene identification, signal peptides of putative neuropeptides were identified using the SignalP v4.0 software tool (http://www.cbs.dtu.dk/services/SignalP/). We identified candidate genes with signal peptides as precursor neuropeptide genes. The transmembrane domains of putative neuropeptides receptors were verified by HMMER (Finn et al., 2011) in the Pfam (http://pfam.xfam.org/) database. Identification of neuropeptides of the other seven wasps was conducted applying the same procedure.

### Sequence Alignments and Phylogenetic Analysis

The sequences of neuropeptide precursors and neuropeptide receptors from *H. hebetor* and other insects were aligned by ClustalX2 software (http://www.clustal.org/clustal2/) and edited with GeneDoc v2.7 software (https://genedoc.software.informer.com/). According to an earlier study, the location of the disulfide bond is distinguishable in the results of the multiple sequence alignment (Kono et al., 1990). A phylogenetic analysis of neuropeptide receptors of *H. hebetor* and other insect species was used to study their evolutionary relationships, for which neighbor-joining trees were constructed in MEGA 7.0 with *n* = 1,000 bootstrap replicates (Kumar et al., 2016). The circle trees were drawn with FigTree v1.4.3 software and all trees were rooted by the *D. melanogaster* metabotropic glutamate receptor (DmCG11144).

### Expression Profiling of the Neuropeptides and Neuropeptide Receptors

Gene expression levels for *H. hebetor* samples from different stages of both sexes were estimated via RNA-Seq using the Expectation Maximization (RSEM) (Li and Dewey, 2011), with expression levels calculated and conveyed as fragments per kilobase of transcript per million (FPKM) values. The expression profiles of each neuropeptide and neuropeptide receptor were visualized by using TBtools (Chen et al., 2020).

### Quantitative Real-Time Polymerase Chain Reaction (qPCR) Analyses

We performed qPCR using SYBR^R^Premix Ex Taq^TM^ II (Tli RNaseH Plus) (Takara, Japan) on the CFX Connect^TM^ Real Time Detection System (Bio-Rad). Total RNA was extracted with TRIzol and quantified by a NanoDrop (Thermo Scientific) spectrophotometer, after which the cDNA was synthesized using a PrimeScript™ One Step RT-PCR Kit (Takara, Japan). Then, in a 25-μL reaction volume, 1 ng cDNA was used as a template for which primers were designed by Primer3 web v4.1.0 and Primer-Blast (http://www.ncbi.nlm.nih.gov/tools/primer-blast/). Initially, we used the reverse transcription polymerase chain reaction (RT-PCR) and sequencing of its product to correct the nucleotide sequences of the selected genes for the qPCR verification. Multiple specific primers were designed for each tested gene. To determine the efficacy of the primers, we performed qPCR for the templates with serial dilutions that ranged from 10 to 10,000, respectively, and calculated their efficiency values. A dissociation curve was drawn spanning 60–95°C at the end of each qPCR reaction, to verify the specificity of the used qPCR primers. Based on the results from these specificity and efficiency verifications, the suitable primers for gene expression profile determination were selected. We chose glyceraldehyde-3-phosphate dehydrogenase (GAPDH) to sever the reference gene (Li et al., 2019). Statistical analyses were implemented in GraphPad Prism 6 (https://www.graphpad.com/). To quantify the mRNA expression levels the 2^−ΔΔ*Ct*^ method was utilized (Livak and Schmittgen, 2001). The correlation of *R*-values was calculated by using data processing system (DPS) software (Tang and Zhang, 2013).

## Results

### Comparison of Neuropeptide Precursor Gene Annotations in Different Insect Databases

Using both Nr-annotation and homology searches, we newly annotated 34 candidate neuropeptide genes in *H. hebetor* (Table 1, Supplementary Material File 1). When compared with other insects, such as *P. puparum* (Xu et al., 2020), *A. mellifera* (Hummon et al., 2006), *D. melanogaster* (Hewes and Taghert, 2001), *B. mori* (Roller et al., 2008), *T. castaneum* (Li et al., 2008), *N. lugens* (Tanaka et al., 2014), and *Chilo suppressalis* (Xu et al., 2016), evidently *H. hebetor* has the fewest neuropeptide precursor genes (Supplementary Table 1), and also slightly fewer types of neuropeptides. Then, by using the RNA-Seq and genomic data of *Cotesia vestalis* (Wei et al., 2010), *D. alloeum* (Lowe and Eddy, 1997), *Diadromus collaris* (Li et al., 2015), *Fopius arisanus* (Lowe and Eddy, 1997), *Macrocentrus cingulum* (Yin et al., 2018)*, Microplitis demolitor* (Burke et al., 2014), and *Trichogramma pretiosum* (Lowe and Eddy, 1997), we were able to identify their neuropeptide precursor genes using the same method (Table 2). In this respect, we annotated 36 neuropeptide precursor genes in *C. vestalis*, 35 in *Diadromus. alloeum*, 33 in *D. collaris*, 38 in *F. arisanus*, 24 in *M. cingulum*, 35 in *M. demolitor*, and 33 in *T. pretiosum* (Table 2). When compared with the other seven Braconidae wasps, the number of neuropeptide precursor genes in *H. hebetor* ranks in the middle.

**Table 1:** Description of neuropeptide genes in *H. hebetor*^a^.

**Peptide name**	**Gene ID**	**Acronym**	**Protein (AA)**	**Assigned receptor ID**
Adipokinetic hormone 1	Hheb05007	AKH1	137	Hheb006770.1
Allatostatin A	Hheb100640.1	AstA	197	Hheb116660.1
Allatostatin CC	Hheb092060.1	AstCC	140	Hheb023220.1, Hheb044860.1
Allatostatin CCC	Hheb092070.1	AstCCC	90	Hheb023220.1, Hheb044860.1
Bursicon alpha subunit	Hheb01190	Burα	108	Hheb00749
Bursicon beta subunit	Hheb109470.1	Burβ	137	Hheb00749
CAPA splicing variant a	Hheb025420.1	CAPA	175	Hheb109890.1, Hheb109910.1
CNMamide	Hheb092090.1	CNMa	106	Hheb08603, Hheb00794
Corazonin	Hheb012400.1	Crz	128	Hheb111350.1
Crustacean cardioactive peptide	Hheb024270.1	CCAP	94	Hheb011130.1
Diuretic hormone 31	Hheb005670.1	DH31	109	Hheb039610.1
Diuretic hormone 44	Hheb091920.1	DH44	182	Hheb104700.1
Ecdysis triggering hormone	Hheb087690.1	ETH	136	Hheb008350.1
Eclosion hormone	scaffold29	EH	82	Hheb03286
Elevenin	Hheb109520.1	Ele	136	nd
FMRFamide	Hheb049790.1	FMRF	181	Hheb077040.1
Insulin-like peptide1	Hheb091810.1	ILP1	633	Hheb003390.1, Hheb037010.1
Insulin-like peptide2	scaffold4	ILP2	95	Hheb003390.1, Hheb037010.1
Ion transport peptide	Hheb087940.1	ITP	329	nd
Leucokinin	Hheb035510.1	LK	239	Hheb039010.1
Myosuppressin	Hheb029860.1	MS	99	Hheb007010.1
Natalisin	Hheb035520.1	NTL	251	Hheb09984
Neuroparsin	Hheb096350.1	NP	127	nd
Neuropeptide-like precursor 1	Hheb000030.1	NPLP1	362	Hheb03289
NVP-like putative neuropeptide	Hheb068000.1	NVP	325	na
Orcokinin A	Hheb092110.1	OKA	135	na
Pheromone biosynthesis activating Neuropeptide/hugin-pyrokinin	Hheb048490.1	PBAN	187	Hheb073560.1
Proctolin	Hheb088460.1	Pro	237	nd
Prothoracicotropic hormone	Hheb092100.1	PTTH	165	nd
RYamide	Hheb04319	RY	111	Hheb033210.1
Short neuropeptide F	Hheb073990.1	sNPF	118	Hheb089310.1
SIFamide	Hheb087710.1	SIF	75	Hheb03416
Tachykinin	Hheb033240.1	TK	565	Hheb05423
Trissin	Hheb066920.1	Tris	90	Hheb035150.1

a *Coding sequences and amino acid sequences of neuropeptides are available in Supplementary Material Files 1, 2. na, not applicable (no receptor known in any insect); nd, not detected in H. hebetor*.

**Table 2:** Neuropeptide genes in Braconidae species^a^.

**Peptide**	**Hh**	**Cv**	**Da**	**Dc**	**Fa**	**Mc**	**Md**	**Tp**
AKH1	+	+	+	nd	+	+	nd	nd
AKH2	nd	nd	nd	nd	nd	nd	nd	nd
ACP	nd	nd	nd	nd	+	nd	nd	+
AstA	+	+	+	+	+	+	+	+
AstB	nd	nd	nd	nd	nd	nd	nd	nd
AstC	nd	nd	nd	nd	nd	nd	nd	nd
AstCC	+	+	+	nd	+	+	+	+
AstCCb	nd	nd	nd	nd	nd	nd	nd	nd
AstCCC	+	+	+	+	+	+	+	+
AT	nd	nd	+	+	+	nd	nd	+
Inotocin	nd	+	+	+	+	nd	+	+
Burα	nd	+	+	+	+	nd	+	+
Burβ	+	+	+	+	+	nd	+	nd
CAPA	+	+	+	nd	+	nd	+	+
CCHa 1	nd	nd	nd	+	nd	nd	nd	+
CCHa 2	nd	nd	nd	+	nd	nd	nd	+
CNMa	+	+	+	+	+	nd	+	+
Crz	+	+	+	+	+	nd	+	nd
CCAP	+	+	+	+	+	nd	+	+
DH31	+	+	+	+	+	nd	+	+
DH44	+	+	+	+	+	+	+	+
DH34	nd	nd	nd	nd	+	nd	nd	nd
DH45	nd	nd	nd	nd	nd	nd	nd	nd
Elv	+	+	+	nd	+	nd	+	nd
ETH	+	+	+	+	+	+	+	nd
EH	+	+	+	nd	+	+	+	nd
FMRF	+	+	+	+	+	+	+	nd
GPA2	nd	nd	nd	nd	nd	nd	nd	nd
GPB5	nd	nd	nd	nd	nd	nd	nd	nd
Hugin-PK2	+	+	+	+	+	+	+	+
IMF	nd	+	nd	nd	+	+	+	+
ILP	+	+	+	+	+	+	+	+
ITP	+	+	+	+	+	+	+	+
ITPL	nd	+	nd	+	+	+	+	nd
LK	+	nd	+	+	+	nd	nd	nd
MS	+	+	+	+	+	+	+	+
NTL	+	nd	+	nd	+	nd	+	nd
NP	+	+	+	+	+	nd	+	+
NPF1	nd	+	nd	nd	nd	nd	nd	+
NPF1b	nd	nd	nd	nd	nd	nd	nd	nd
NPF2	nd	nd	nd	nd	nd	nd	nd	+
NPLP1	+	+	+	+	+	+	+	nd
NPLP2	nd	nd	nd	nd	nd	nd	nd	nd
NPLP3	nd	nd	nd	nd	nd	nd	nd	nd
NPLP4	nd	nd	nd	nd	nd	nd	nd	nd
NVP	+	+	+	+	+	+	+	+
OKA	+	+	+	nd	+	+	+	+
OKB	nd	+	+	+	+	nd	nd	nd
PDF	nd	+	+	+	nd	+	+	+
Pro	+	+	nd	+	nd	+	nd	+
PTTH	+	+	+	+	+	+	+	nd
RY	+	+	+	+	+	+	+	+
sNPF	+	+	+	+	+	+	+	+
sNPFb	nd	nd	nd	nd	nd	nd	nd	nd
SIF	+	+	nd	nd	+	+	+	+
SK	nd	nd	nd	+	nd	nd	nd	nd
SP	nd	nd	nd	nd	nd	nd	nd	nd
TK	+	+	+	+	+	+	+	+
TR	nd	+	+	+	nd	nd	+	nd

nd, not identified; +, identified.

a *The data for other insects are taken from C. vestalis (Wei et al., 2010), D. alloeum (Lowe and Eddy, 1997), D. collaris (Li et al., 2015), F. arisanus (Lowe and Eddy, 1997), M. cingulum (Yin et al., 2018), M. demolitor (Burke et al., 2014), and T. pretiosum (Lowe and Eddy, 1997)*.

### Missing and Unique Neuropeptide Precursor Genes in *H. hebetor* in Comparison With Other Insects

Unlike *C. vestalis, D. alloeum, D. collaris, F. arisanus, M. cingulum, M. demolitor, T. pretiosum, N. vitripennis* (Hauser et al., 2010) and *P. puparum* (Xu et al., 2020), neither allatotropin (AT), inotocin, CCHamide (CCHa), neuropeptide F (NPF) nor pigment dispersing factor (PDF) were found in the transcriptome databases of *H. hebtor* (Tables 1, 2, Supplementary Table 1). Both allatostatin C (AstC) and allatostatin B (AstB) were missing in several wasp species analyzed in this study, namely *P. puparum, N. vitripennis, A. mellifera*, and *H. hebetor*, but the two were present in *D. melanogaster, B. mori*, and *T. castaneum* (Table 2, Supplementary Table 1). We also did not find the glycoprotein hormone alpha 2 (GPA2) and glycoprotein hormone beta 5 (GPB5) in Hymenoptera wasps analyzed (Table 2, Supplementary Table 1). Furthermore, sulfakinin (SK) was found in *A. mellifera* and *D. collaris*, yet it was absent in other wasps analyzed (Table 2, Supplementary Table 1). Taken together, these results indicate that some neuropeptides are generally missing in wasps. We did identify natalisin (NTL), FMRFamide (FMRF), and leucokinin (LK) in *H. hebetor* (Table 1), which had not been annotated in *N. vitripennis* (Hauser et al., 2010) or *P. puparum* (Xu et al., 2020), however, they were also found in other Braconidae wasps. For example, NTL was annotated in *D. alloeum, F. arisanus*, and *M. demolitor*. Moreover, we could only find an adipokinetic hormone 1 (AKH1) in the genome of *H. hebetor*, a result consistent with that for the braconid wasps, *M. cingulum* (Table 2).

In identifying the genes encoding neuropeptides and their receptors in insects, it became apparent that at least three neuropeptide genes seemed prevalent: those encoding for corazonin (Crz), LK, and allatostatin A (AstA) (Veenstra, 2019a). We also annotated these three genes in *H. hebetor* in the present study (Table 1). The number of neuropeptide precursor genes in *H. hebetor* was lower than that in non-parasitic insect species, such as *B. mori, C. suppressalis, D. melanogaster, N. lugens, T. castaneum*, and *A. mellifera*, probably due to its parasitic life cycle (Supplementary Table 1). Furthermore, we identified the lowest number of neuropeptides in *M. cingulum* (Table 2). With the exception of *F. arisanus*, all the braconid wasps lacked an adipokinetic hormone/corazonin-related peptide (ACP) (Table 2). In stark contrast, ACP, along with AT, inotocin, CCHa, and PDF, were absent in *H. hebetor* compared with *P. puparum* and *N. vitripennis* (Supplementary Table 1). This disparity likely exists because these three wasps are from two different families. Yet many differences were discerned in the comparison within Braconidae among *C. vestalis, D. alloeum, D. collaris, F. arisanus, M. cingulum, M. demolitor, T. pretiosum*, and *H. hebetor*. For example, we could not identify inotocin in *H. hebetor* and *M. cingulum*, but inotocin was found in the six other wasps we investigated (Table 2). We speculate such differences may also relate to the species' host specificity and parasitic mode. Apart from *H. hebetor*, the other seven wasps are endoparasitoids, and both *F. arisanus* and *T. pretiosum* parasitize host eggs, whereas the other species parasitize larvae. The hosts of *F. arisanus* and *D. alloeum* belong to Diptera, while the other wasps can only parasitize Lepidoptera.

### Multiple Sequence Alignments of Some Neuropeptides in *H. hebtor*

**Figure 1:**
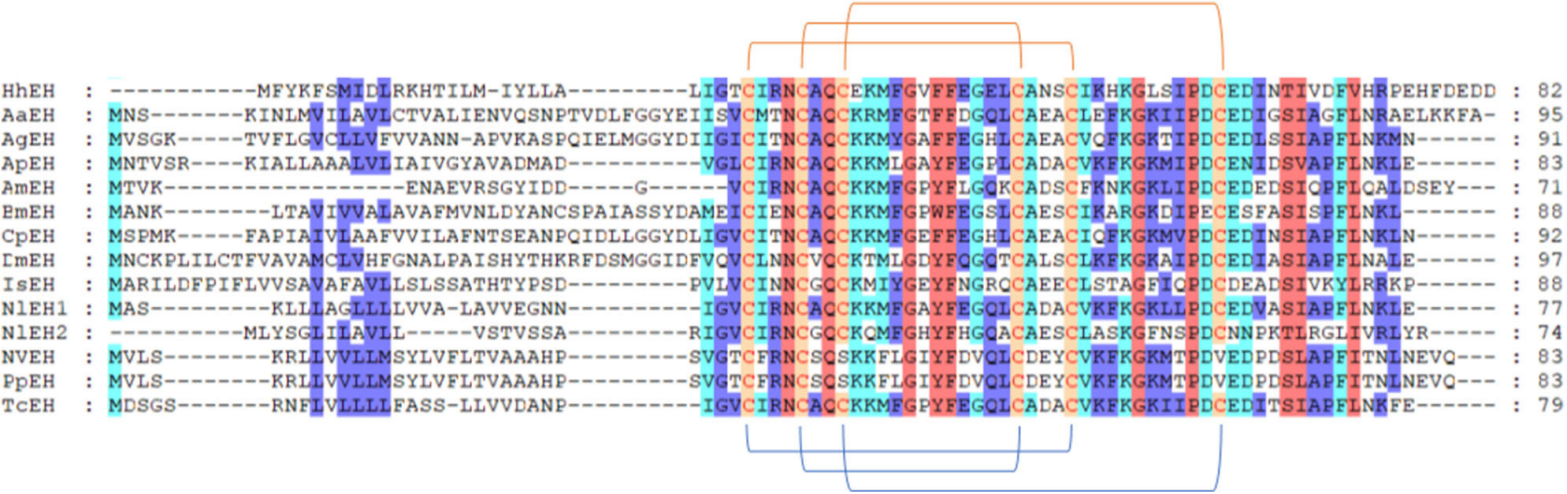
Multiple sequence alignment of the eclosion hormone (EH) from *H. hebetor* (HhEH), *Aedes aegypti* (AaEH, XP_001652203.1), *Anopheles gambiae* (AgEH, XP_001230805.1), *A. mellifera* (AmEH, XP_001122120.1), *Acyrthosiphon pisum* (ApEH, XP_001943617.1), *B. mori* (BmEH, NP_001037307.1), *Culex quinquefasciatus* (CqEH, XP_001864428.1), *D. melanogaster* (DmEH, NP_524386.1), *Ixodes scapularis* (IsEH, XP_002399271.1), *Nilaparvata lugens* (NlEH1, AB817253.1; NlEH2, BAO00951.1), *N. vitripennis* (NvEH, XP_001603337.1), and *T. castaneum* (TcEH, XP_969164.1). Amino acid residues common in at least four sequences are highlighted. The blue lines below the sequences denote positions of the three disulfide bonds usually present in EH. The orange lines above the sequences denote positions of the three disulfide bonds in HhEH.

**Figure 2:**
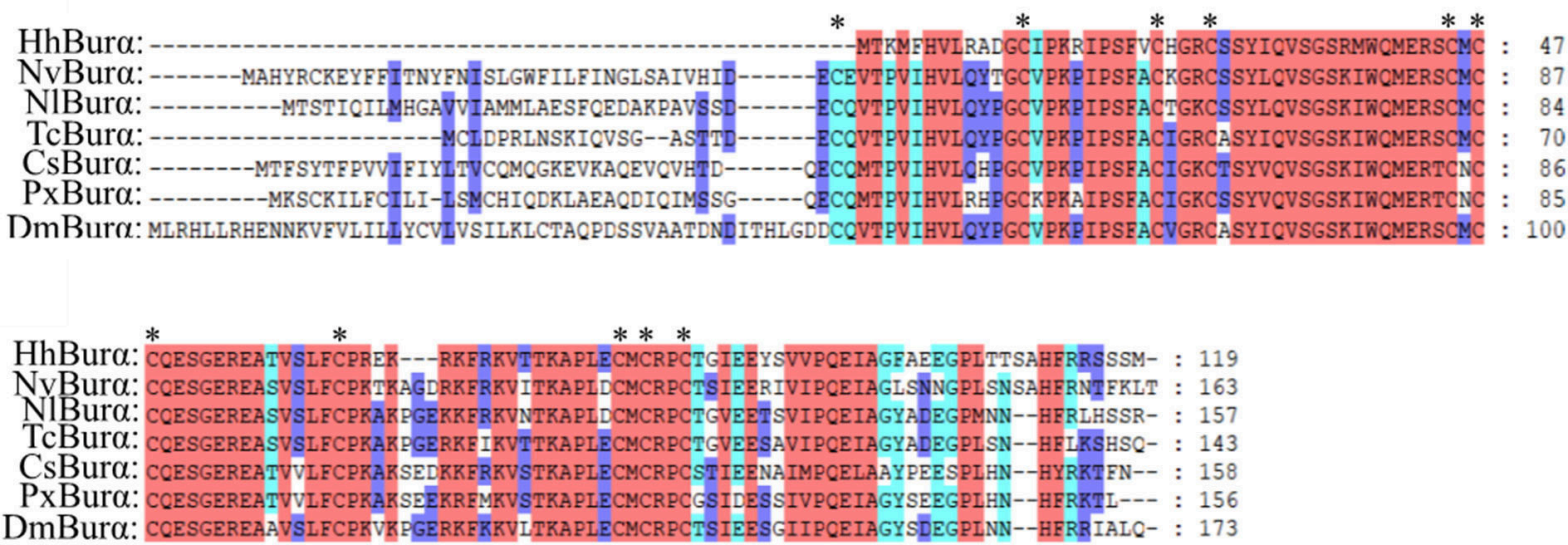
Multiple sequence alignment of the bursicon alpha subunit (Burα) from *H. hebetor* (HhBurα), *D. melanogaster* (DmBurα, CAH74223.1), *C. suppressalis* (CsBurα, ALM30306.1), *Nilaparvata lugens* (NlBurα, BAO00937.1), *N. vitripennis* (NvBurα, NP_001155852.1), *Plutella xylostella* (PxBurα, AJM76770.1), and *T. castaneum* (TcBurα, ABA40402.1). Amino acid residues common in at least four sequences are highlighted. Asterisks indicate those cysteine residues proposed to be involved in cycling bridges.

**Figure 3:**
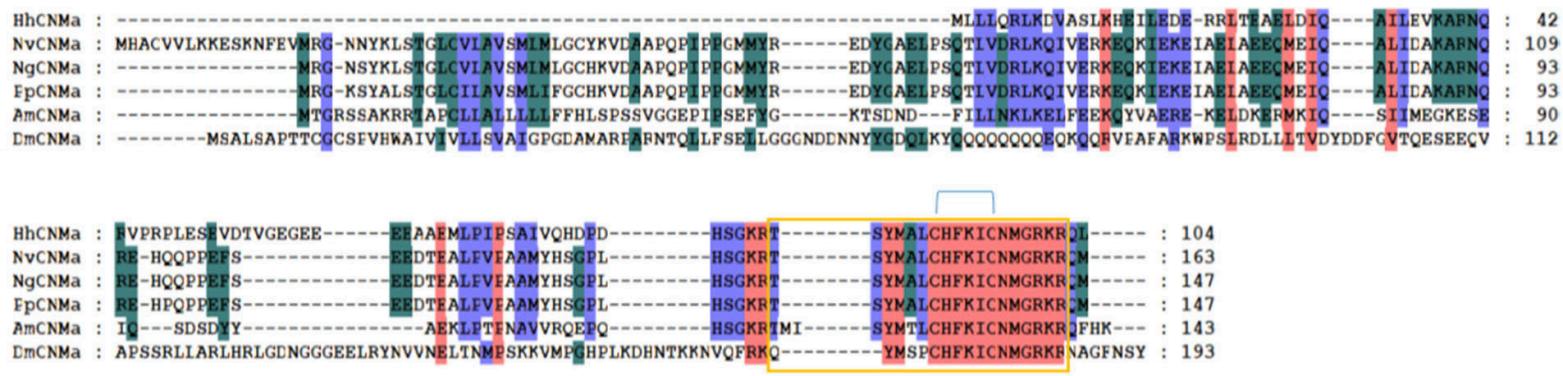
Multiple sequence alignment of the CNMamide (CNMa) from *H. hebetor* (HhCNMa), *A. mellifera* (AmCNMa, XP_001121373.1), *D. melanogaster* (DmCNMa, AAF47581.2), *N. vitripennis* (NvCNMa, XP_016840002.1), *Nasonia girault* (NvCNMa, GBEC01006871.1), and *P. puparum* (PpCNMa, PPU11419-RA). Amino acid residues common in at least four sequences are highlighted. The blue lines above the sequences denote positions of the three disulfide bonds in CNMa. The orange boxes indicate the mature peptides.

We annotated the eclosion hormone (EH) in *H. hebetor* (Table 1). The alignment of EH sequences from various insects showed that *H. hebetor* EH contains all three disulfide bridges usually present in EHs (Figure 1). Notably, the sequences of some candidate neuropeptide genes were found to be incomplete, in that they did not harbor signal peptide, as in the case of the bursicon alpha subunit (Burα) and CNMamide (CNMa). Nevertheless, those sequences were able to align with the same genes in other insects (Figures 2, 3). The complete genes of the bursicon beta subunit (Burβ) and a bursicon receptor were found in *H. hebetor*; hence, we deemed Hheb01190 candidate neuropeptide gene. CNMa is a member of a conserved insect neuropeptide family recently identified in the genomes of *D. melanogaster* and other species, including *Rhodnius prolixus* (Traverso et al., 2016) so named after its C-terminal consensus motif (Jung et al., 2014).

### Receptors for Neuropeptides in *H. hebetor*

#### G Protein-Coupled Receptors (GPCRs) for Neuropeptides

A total of 44 putative neuropeptide receptors were identified in the transcriptomes of *H. hebetor* (Supplementary Material File 2). Of these receptors, 32 GPCRs belong to the A-family and another five to the B-family, two are leucine-rich repeat-containing GPCRs (LGRs), three are receptor guanylyl cyclase (RGCs), two are receptor tyrosine kinases (RTKs). If a likely receptor gene is identified in *H. hebetor*, this gives additional support for the presence of these neuropeptides in *H. hebetor*. Those neuropeptides, such as CCHa, NPF, and PDF, which we could not identify, yet found a corresponding putative receptor in *H. hebetor*, are probably missing from this wasp. However, according to Roller et al. (2008), the genes encoding CCHamides and their receptors can be found in most sequenced insect genomes (Roller et al., 2008). Another reason for why some were missing in *H. hebetor* could be the large differences between their sequences in Braconidae vis-à-vis those in other species. Similarly, neuroparsin (NP) was annotated in *H. hebetor*, but its receptors were not found. These missing neuropeptides may require further experimental verification.

#### A-Family GPCRs

**Figure 4:**
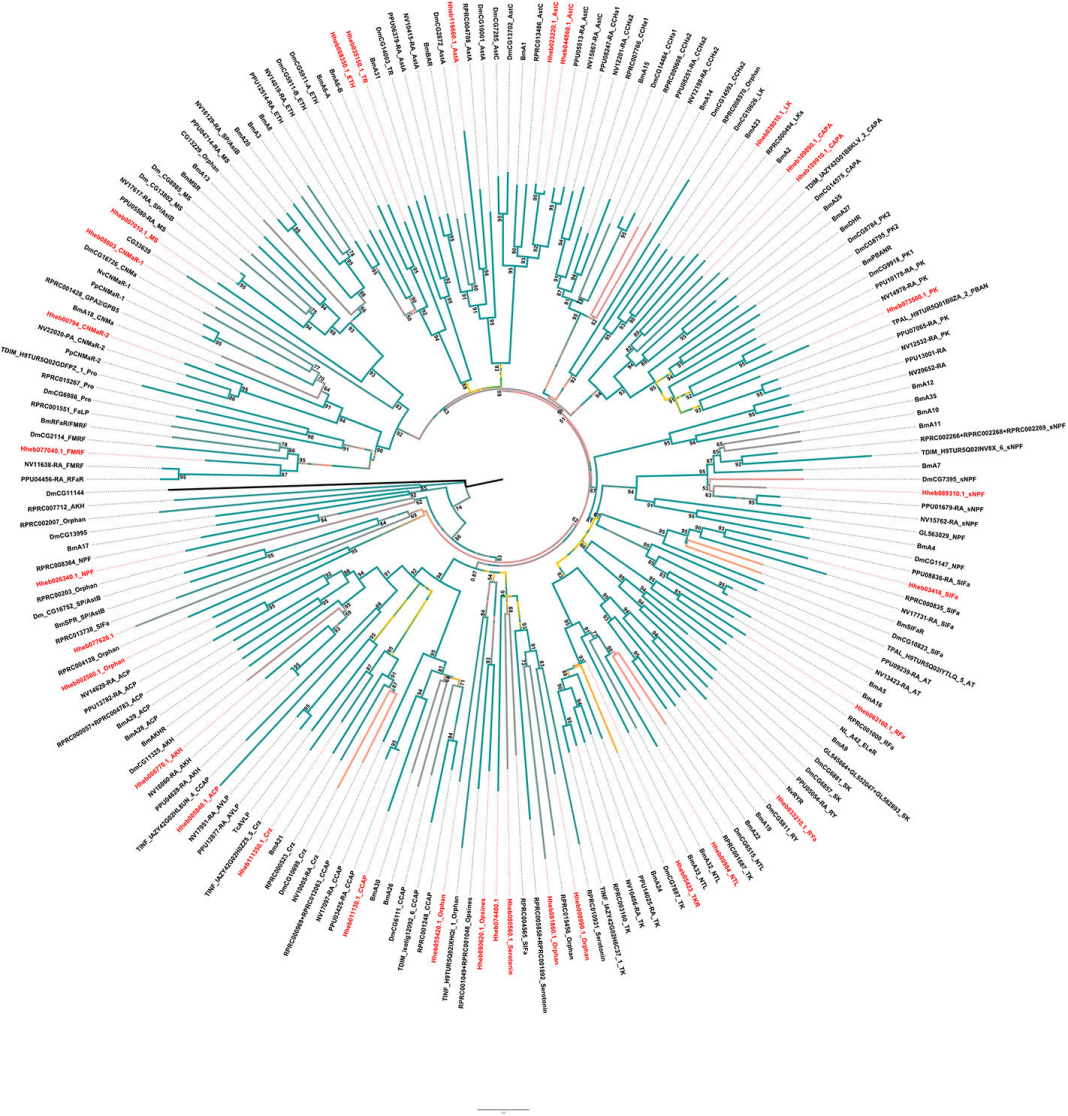
Phylogenetic tree analysis of the family-A neuropeptide GPCRs in the Cluster from *H. hebetor* (Hh), *B. mori* (Bm), *D. melanogaster* (Dm), *P. puparum* (Pp), *N. vitripennis* (Nv), *Rhodnius prolixus* (RPR), *Triatoma dimidiate* (TDIM), *Triatoma infestans* (TINF), *Triatoma pallidipennis* (TPAL), and *T. castaneum* (Tc). The numbers at the nodes of branches indicate the posterior probability for each branch. The GPCRs from *H. hebetor* are highlighted in red. The amino acid sequences used are listed in the Supplementary Material File 3.

The A-family GPCRs are also known as the rhodopsin family (Jung et al., 2014). Here we identified 32 A-family GPCRs in *H. hebetor* (Figure 4). For example, one subassemblage includes the receptors for AKH, ACP, and Crz. Since those three and their corresponding GPCRs do not always occur together in all insects (Li et al., 2008). We tried to annotate all of them, but the receptor of ACP found by BLAST in *H. hebetor* did not cluster with the ACPs of *B. mori, N. vitripennis*, and *P. puparum*, and being positioned instead on the outskirts of this phylogenetic branch (Figure 4).

There are two AstC receptors positioned in *H. hebetor* one of the Cluster (Figure 4), which differs from *P. puparum*, namely Hheb023220.1 and Hheb044860.1. We also found two CAPA receptors in *H. hebetor*, whereas neither CAPA or its receptor occurred in *N. vitripennis*, and the receptors of CAPA and pyrokinin were clustered together in the phylogenetic tree. There are other orthologs evident as well, including RPRC004565, Hheb55420.1, Hheb008990.1, Hheb008990.1, and Hheb07740.1 (Figure 4), all of which are orphan receptors. Some of these A-family GPCRs in *H. hebetor, N. vitripennis* and *P. puparum* were clustered together, such as Hheb077040.1, the receptor of FMRF.

#### B-Family GPCRs

**Figure 5:**
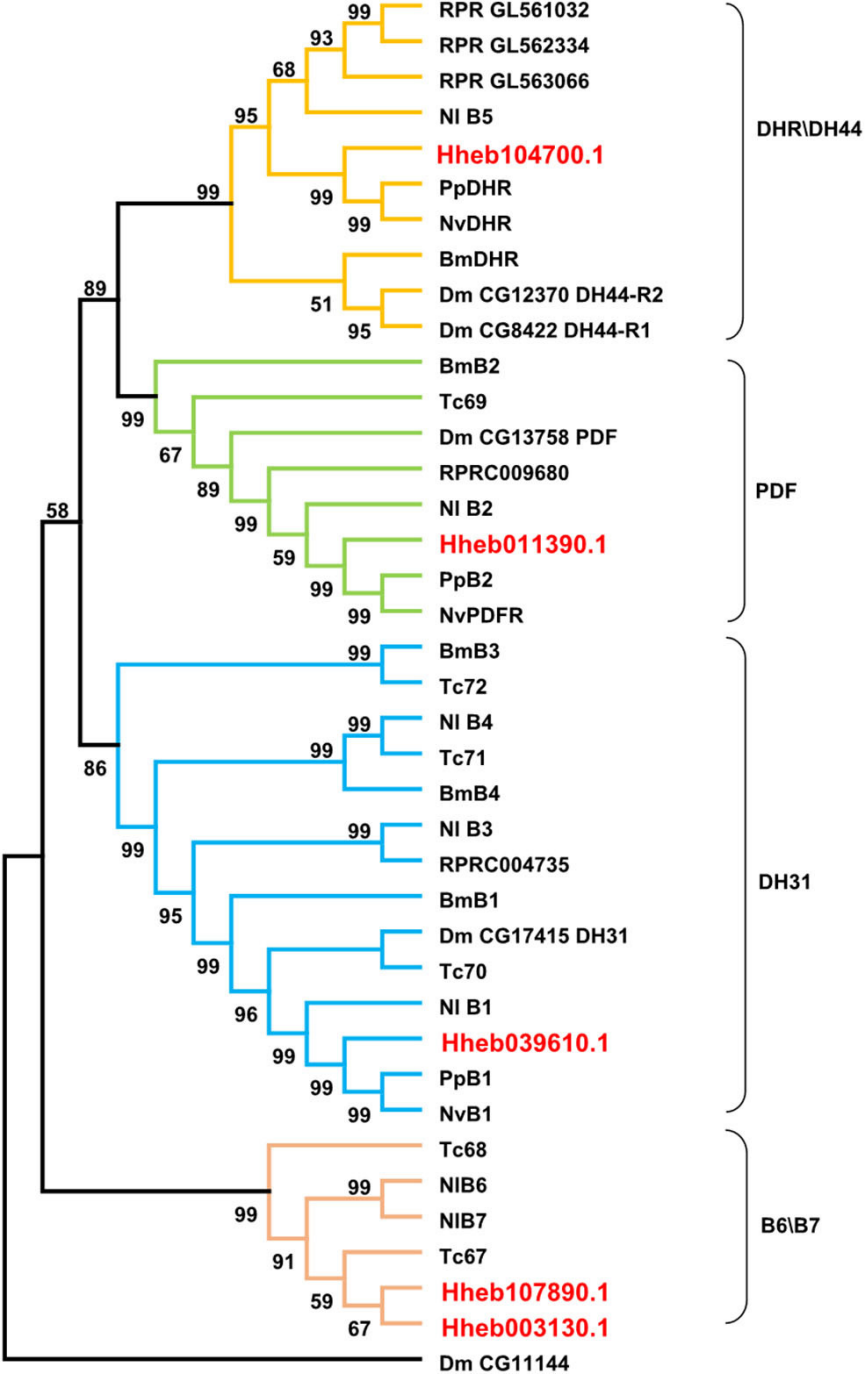
Phylogenetic tree analysis of the family B neuropeptide GPCRs from *H. hebetor* (Hh), *B. mori* (Bm), *D. melanogaster* (Dm), *P. puparum* (Pp), *N. vitripennis* (Nv), *Nilaparvata lugens* (Nl), *Rhodnius prolixus* (RPR), and *T. castaneum* (Tc). The numbers at the nodes of branches indicate the posterior probability for each branch. The red labels are from *H. hebetor*. The amino acid sequences used are listed in the Supplementary Material File 4.

Family B (secretin-like) GPCRs include the calcitonin-like receptor (CTR), CRF-like diuretic hormone receptor (DHR) and pigment dispersing factor receptor (PDFR) (Ons et al., 2016). We found 5 GPCRs belonging to B-family GPCRs in *H. hebetor*, including one diuretic hormone 44 (DH44) receptor, one PDF receptor, one diuretic hormone 31 (DH31) receptor and two other B-family GPCRs (Figure 5). In the phylogenetic tree, Hheb039610.1 is closely related to PpB1 and NvB1, while Hheb104700.1 is closely related to both PpDHR and NvDHR (Figure 5). The two GPCRs were orthologs of B6 B7, while the orthologs of B3 and B4 are not present in *H. hebetor* (Figure 5).

#### LGRs

**Figure 6:**
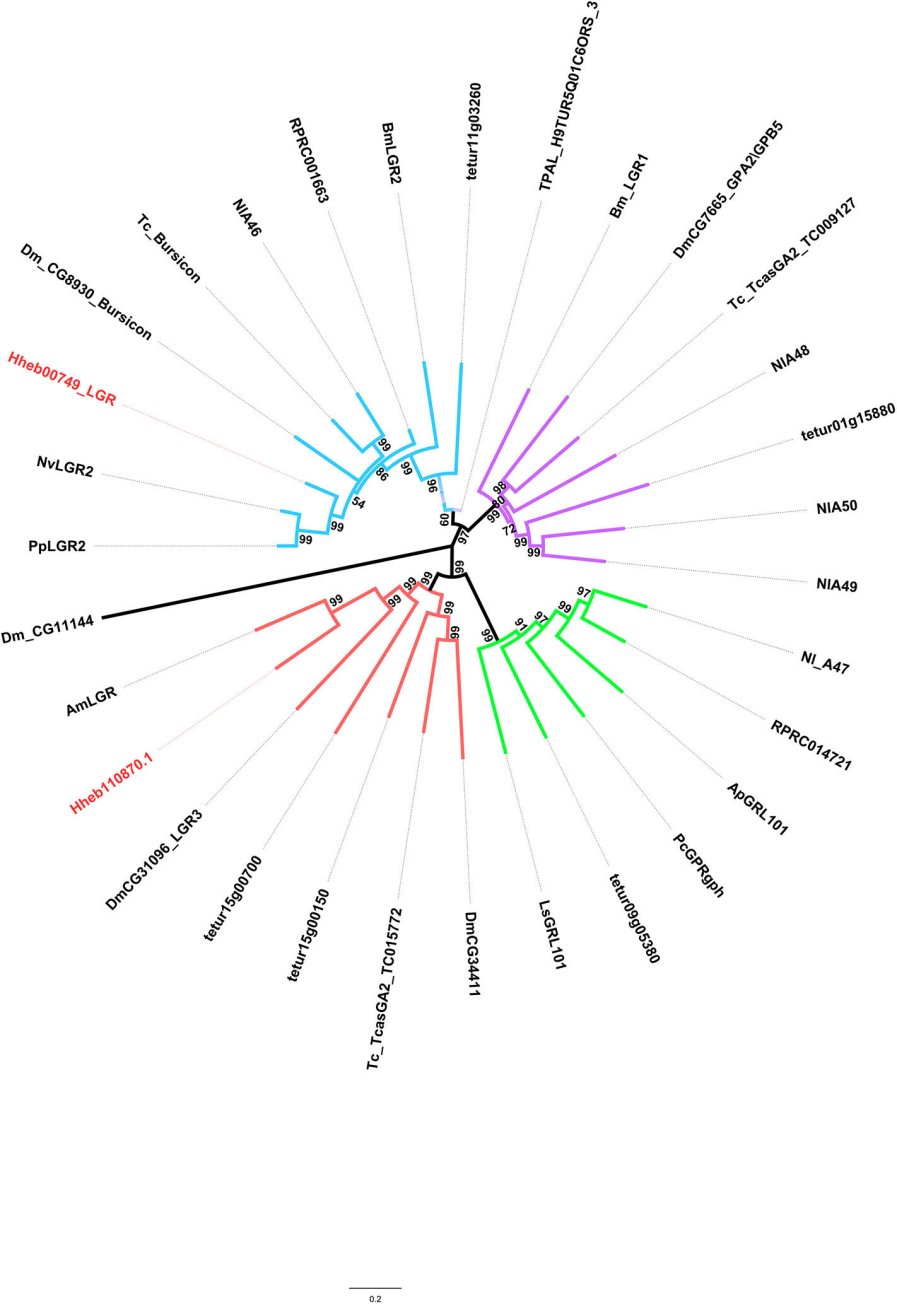
Phylogenetic tree analysis of the LGRs (leucine-rich repeat-containing GPCRs) from *H. hebetor* (Hh), *A. mellifera* (Am), *A. pisum* (Ap), *B. mori* (Bm), *D. melanogaster* Dm), *L|ymnaea stagnalis* (Ls), *Pediculus humanus corporis* (Pc), *P. puparum* (Pp), *Nilaparvata lugens* (Nl), *N. vitripennis* (Nv), *T. castaneum* (Tc), *Rhodnius prolixus* (RPR), *T. dimidiate* (TDIM), *T. pallidipennis* (TPAL), and *Tetranychus urticae* (tetur). The numbers at the nodes of branches indicate the posterior probability for each branch. The red labels are from *H. hebetor*. The amino acid sequences used are listed in the Supplementary Material File 5.

LGRs are receptors with key functions in organismal development and reproduction, a prime example being the bursicon receptor, which triggers the cuticle hardening and tanning in newly emerged insects (Van Hiel et al., 2012). Three distinct types of LGR (type A-C)are known to exist, distinguishable by their number of leucine-rich repeats (LRRs), their type-specific hinge region, and the presence or absence of a low density lipoprotein receptor domain class A (LDLa) motif (Van Hiel et al., 2012). In *H. hebetor*, a type C1 LRR was identified, it being an ortholog of the fruit fly's LGR3 and LGR4 (Figure 6); likewise, a type B LRR was also identified (Figure 6) this being receptor of bursicon. However, the orthologs of Type A and Type C2 were not present in *H. hebetor*.

#### RGCs

**Figure 7:**
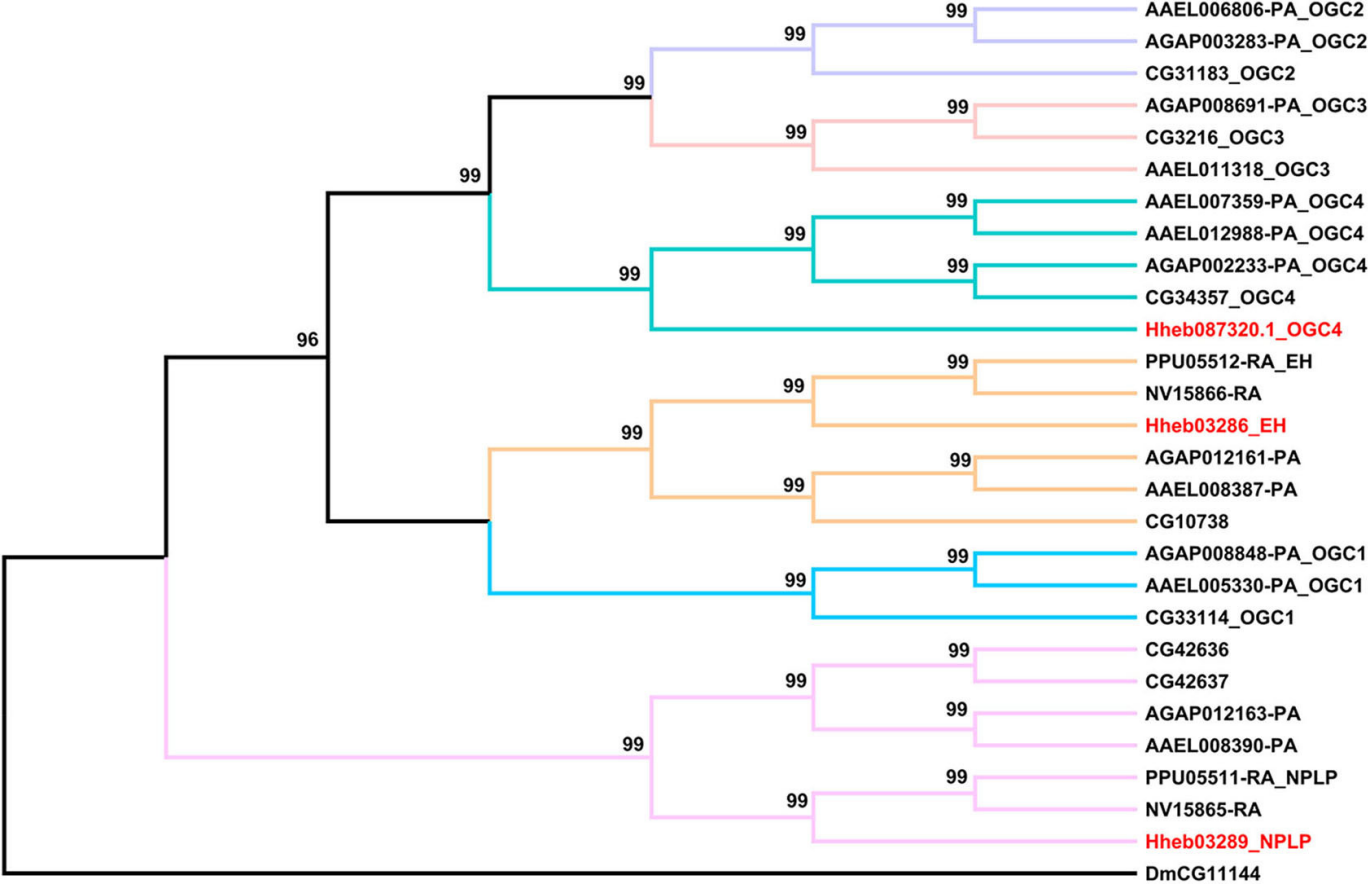
Phylogenetic tree analysis of RGCs (receptor guanylyl cyclase) from *H. hebetor* (Hh), *A. aegypti* (AAEL), *A. gambiae* (AGAP), *D. melanogaster* (Dm), *P. puparum* (Pp), and *N. vitripennis* (Nv). The numbers at the nodes of branches indicate the posterior probability for each branch. The red labels are from *H. hebetor*. The amino acid sequences used are listed in the Supplementary Material File 6.

RGCs are conserved homodimeric membrane proteins with an intracellular protein kinase and guanylyl cyclase domains that catalyze the formation of cGMP (Potter, 2011). Here we identified three neuropeptide receptors which belong to the RGC family. In addition, similar to the circumstances of *P. puparum* and *N. vitripennis*, we found an EH receptor and a NPLP receptor in *H. hebetor*. Notably, Hheb087320.1 is an ortholog of orphan receptor guanylyl cyclase 4 (OGC4) (Figure 7).

#### RTKs

**Figure 8:**
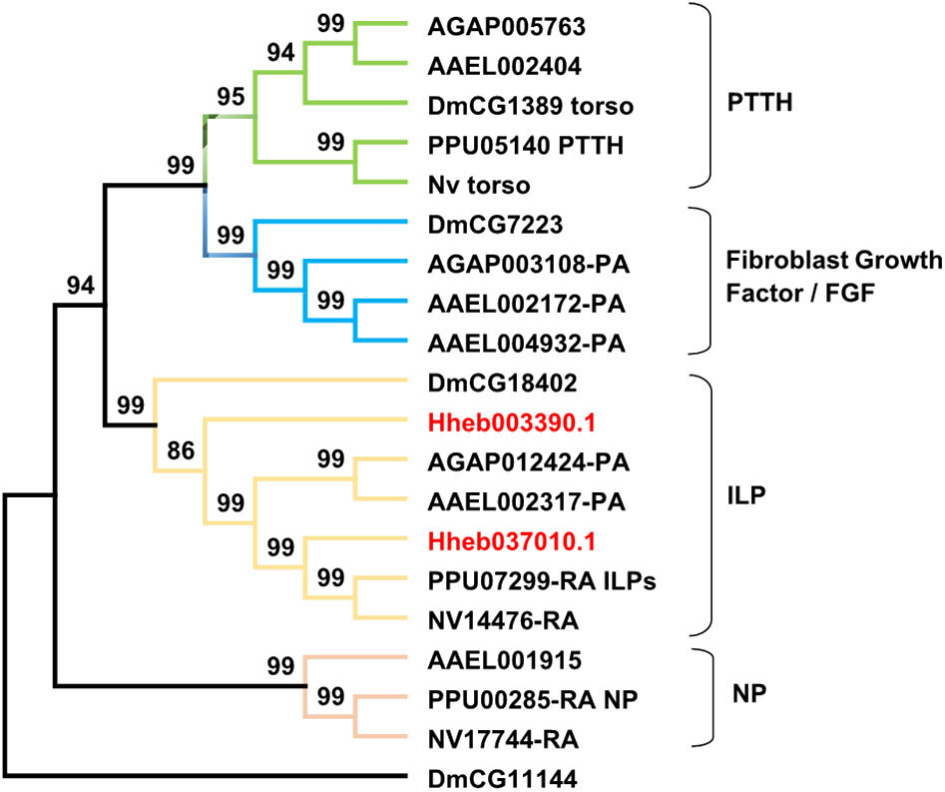
Phylogenetic tree of RTKs (receptor tyrosine kinases) from *H. hebetor* (Hh), *A. aegypti* (AAEL), *A. gambiae* (AGAP), *D. melanogaster* (Dm), *P. puparum* (Pp), and *N. vitripennis* (Nv). The numbers at the nodes of branches indicate the posterior probability for each branch. The red labels are from *H. hebetor*. The amino acid sequences used are listed in the Supplementary Material File 7.

The receptors of prothoracicotropic hormone (PTTH), ILP, and NP all belong to the family of RTKs. In this study, we identified two receptors in *H. hebetor* that belonged to the RTKs (Figure 8), both being orthologs of the ILP receptor (InR). A phylogenetic analysis indicated that the NP receptor and InR are closely related (Xu et al., 2020).

### Expression Profiles of Neuropeptides and Neuropeptide Receptors

**Figure 9:**
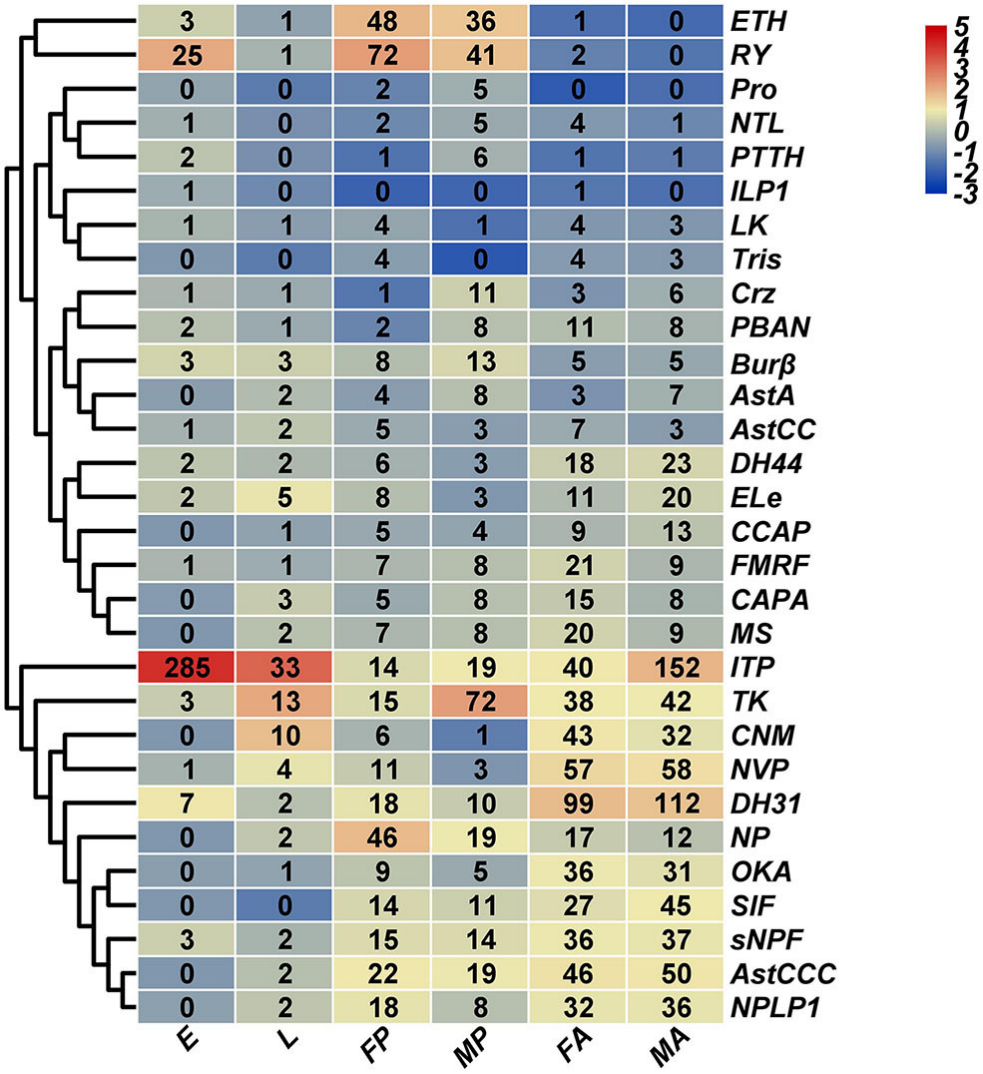
Expression profiles of *H. hebetor* neuropeptide precursor genes across different developmental stages. Log2 FPKM (fragments per kilobase of transcript per million) values for neuropeptide precursor genes are presented by bar colors where darker red represents higher expression values, and darker navy blue represents lower expression values. FA, female adults; MA, male adults; E, eggs; L, larvae; FP, female pupae; MP, male pupae.

**Figure 10:**
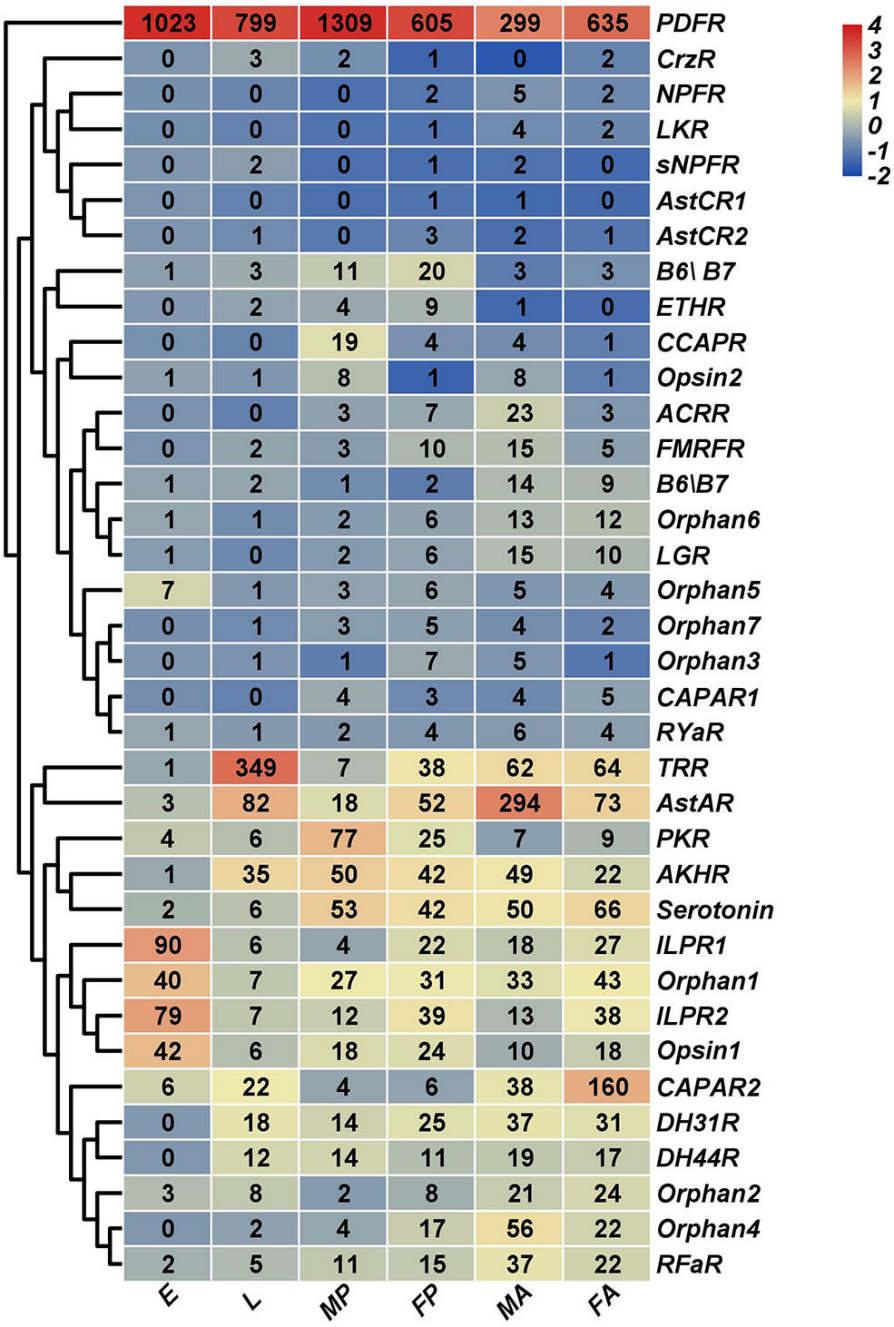
Expression profiles of *H. hebetor* neuropeptide receptor genes across different developmental stages. Log2 FPKM (fragments per kilobase of transcript per million) values for neuropeptide precursor genes are presented by bar colors where darker red represents higher expression values, and darker navy blue represents lower expression values. FA, female adults; MA, male adults; E, eggs; L, larvae; FP, female pupae; MP, male pupae.

Based on *H. hebetor*'s RNA-Seq data, the expression profiles of its neuropeptides and neuropeptide receptors at different developmental stages (embryo, larva, pupa, adult) were characterized (Figures 9, 10). The genes *AstA, AstCC, FMRF, Crz, NTL, ILP1*, and *LK* were expressed at low levels in nearly all stages, whereas the ion transport peptide (ITP) was highly expressed in every stage, especially the embryonic stage (Figure 9). Both ITG and tachykinin (TK) were highly expressed through development except in the embryonic stage (Figure 9). Expression of ecdysis triggering hormone (ETH) was greater in the pupal stage than other developmental stages (Figure 9). Stage-specific expression profiles of the neuropeptides indicated that mRNAs encoding *ITP* undergo a high level of expression in the embryos, in contrast to *NP, RY* and *ETH*, whose expression was higher in the pupal stage (Figure 9). Concerning *OKA, ITG* and *DH31*, they were expressed more in the adult stage (Figure 9). Finally, these expression profiles also revealed that *TK* was preferentially expressed in the male pupae of *H. hebetor* (Figure 9).

Using for inference the RNA-Seq of the wasp tissues salivary glands, venom glands, the carcass of larvae without salivary glands, and the carcass of female adults without the venom glands, we characterized their expression profiles. This showed that TK and ITP were expressed at higher levels in both the venom and salivary glands than in the corresponding residual tissue (Supplementary Figure 1). Most expressed in the venom gland tissues was ILP1, whereas it was barely expressed in salivary glands (Supplementary Figure 1). We also determined that PDFR was highly expressed in all developmental stages and tissues examined (Figure 10, Supplementary Figure 2), but we failed to annotate PDF in *H. hebetor*. The mRNAs encoding AstAR underwent a high level of expression in male adults (Figure 10); conversely, it was the mRNAs encoding CAPAR2 that were most expressed in female adults (Figure 10). Many receptors, however, were not at all expressed in the salivary and venom glands (Supplementary Figure 2). From the cluster perspective, females and males differed little, indicating negligible sex specificity, but the adults were significantly separate from the pre-eclosion period, suggesting the expression of these neuropeptides before and after the wasp's emergence is different.

**Figure 11:**
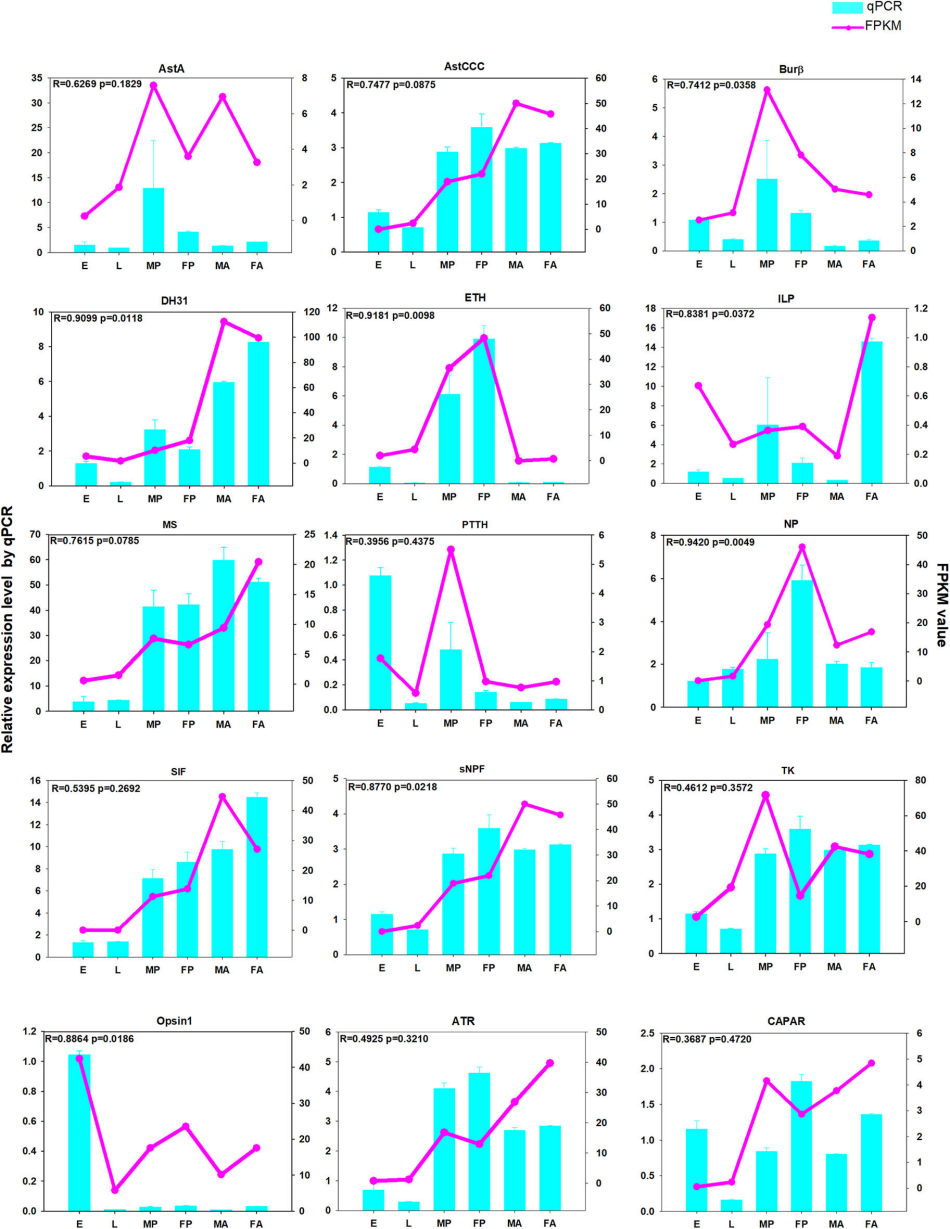
The qPCR analysis of relative expression levels of neuropeptide genes and neuropeptide receptor genes at different developmental stages. E, embryos; L, larvae; FA, female adults; FP, female pupae; MA, male adults; MP, male pupae. The pink-dotted line represents the FPKM (fragments per kilobase of transcript per million) value, and the relative expression levels from the qPCR are depicted in the light blue histogram.

To verify the reliability of our RNA-Seq data, we randomly selected some genes for a qPCR analysis. These results revealed that the expression patterns of those genes generally agreed with that registered by RNA-Seq. For example, the developmental expression profiles *ETH* and *NP* obtained by qPCR were consistent with those registered by RNA-Seq (Figure 11). Accordingly, the positive correlation was extremely strong (*R*-values > 0.80), for the four genes: *ETH* (*R* = 0.9181, *p* = 0.0098), *NP* (*R* = 0.9420, *p* = 0.0049), *DH31* (*R* = 0.9099, *p* = 0.0118), and *ILP1* (*R* = 0.8381, *p* = 0.0372). The other selected genes had nearly identical expression trends recorded via both analyses (*R*-values > 0.3) (Figure 11). In addition, the qPCR results for AstCCC, TK and AstCCR each had the same peak in the expression profiles as determined by the RNA-Seq at different developmental stages (Supplementary Figure 3). Overall, these validation results indicate that our RNA-Seq data were robust.

## Discussion

As ancient molecules, neuropeptides and peptide hormones mediate cell-to-cell communication in all multicellular animals (Li et al., 2008; Garczynski et al., 2019). To date, many neuropeptides have been identified and sequenced in several insect species (Yeoh et al., 2017). Insect neuropeptides show heterogeneous similarities to mammals, yet display clear similarity among insects taxa, despite the large phylogenetic distance across these huge and various classes (Yeoh et al., 2017). Indeed, it is well-established that the structure of the genes encoding neuropeptides and their receptors are highly conserved during evolutionary history, and this is not surprising because they are important regulators of various vital physiological processes (Li et al., 2008; Veenstra, 2019a,b). Neuropeptide precursors share few features in common, however. The only common feature is the presence of an amino-terminal signal peptide that directs the ribosomes synthesizing neuropeptide precursors to the endoplasmic reticulum (ER). Most neuropeptides can interact with GPCRs, thereby generating an intracellular response (Clynen et al., 2010). Neuropeptides and their receptors regulate fundamental events in the insect life cycle, hence, they have been proposed as potential insecticides or targets to replace or complement the use of neurotoxic compounds against pests (Verlinden et al., 2014). In addition, potential mutations to the neuropeptide precursor or receptor genes are usually harmful, so resistance is not easy to evolve (Ons, 2017). Insect neuropeptides are interesting because their receptors may be reasonable targets for a new generation of insecticides (Veenstra, 2019a), and we should also study these related genes in natural enemy insects (predators, parasitoids) to avoid harming them with insecticides.

In this study, we annotated 33 neuropeptide genes in *H. hebetor* by Nr-annotation and homology searches. Inotocin was first discovered from the central nervous system (CNS) of *Locusta migratoria*, and later also identified in *T. castaneum, N. vitripennis, P. puparum, C. vestalis, D. alloeum, D. collaris, F. arisanus, M. demolitor, T. pretiosum*, and *N. lugens* (Table 2, Supplementary Table 1). But it is apparently the lost from *A. mellifera* (Hummon et al., 2006) and *H. hebetor*. The genes encoding two well-known neuropeptides, PDF and NPF were not found in the *H. hebetor* genome (Table 1). PDF was first isolated from crustaceans, earning its name because of its pigment cell-dispersing activity (Hauser et al., 2010). Similar peptides were later found in insects (Rao et al., 1987), and these participate in the regulation of circadian rhythm (Renn et al., 1999). In *D. melanogaster*, PDF is involved in maintaining behavioral rhythms (Lin et al., 2004), while NPF has the function of regulating olfactory responses to food odor (Lee et al., 2020). In *D. melanogaster*, NPF is a 36 amino acid peptide whose C-terminal sequence is RVRFamide (Hauser et al., 2010), it is structurally related to the vertebrate neuropeptide Y family and affects food intake and feeding behavior (Wu et al., 2003). Interestingly, we found the highly expressed PDF receptor in *H. hebetor* (Figure 10 and Supplementary Figure 2) but could not identify the PDF neuropeptide. We speculate this absence arose because we failed to annotate it in the genomic and transcriptome data, rather than it not existing *per se* in *H. hebetor*. This would fit with the fact that we did not obtain the transcriptome of the neural tissue and so could have easily missed those genes represented by small fragments and low expression when we annotated them. In this context, we also could not find NPF in *H. hebetor*, however, it is a very conservative neuropeptide, and it also went unfound in *D. alloeum, D. collaris, F. arisanus, M. cingulum*, and *M. demolitor* in this study. This result is similar to findings reported by Chang et al. (2018), who also did not find NPF in *F. arisanus, D. collaris*, and *D. alloeum* (Chang et al., 2018). Thus, we speculate there are the large differences between the sequences in Braconidae and those in other species. This would explain the failed annotations made by using BLASTP and TBLASTN. Nonetheless, we did succeed in identifying the receptor of NPF, but at a low expression level (Figure 10, Supplementary Figure 2), the receptor of inotocin was never found. Therefore, we speculate that inotocin neuropeptides may have been lost from *H. hebetor*.

CCHa members and their receptors were also lost from all braconids analyzed in this study, except *D. collaris* and *T. pretiosum* (Table 2). More than 10 years ago, Roller et al. (2008) discovered a novel neuropeptide CCHa in *B. mori*, and recently, two CCHa genes (*CCHa1* and *CCHa2*) were identified in the tsetse fly *Glossina morsitans* (Wang Z. et al., 2018). This type of neuropeptides contains two highly conserved cysteines and an amidated histidine residue at the C-terminus (Roller et al., 2008). The CCHa pre-prohormone is expressed in several small neurons in the CNS and in the midgut endocrine cells of *B. mori* larvae, but the biological function of the peptide remains unknown (Roller et al., 2008).

**Figure 12:**
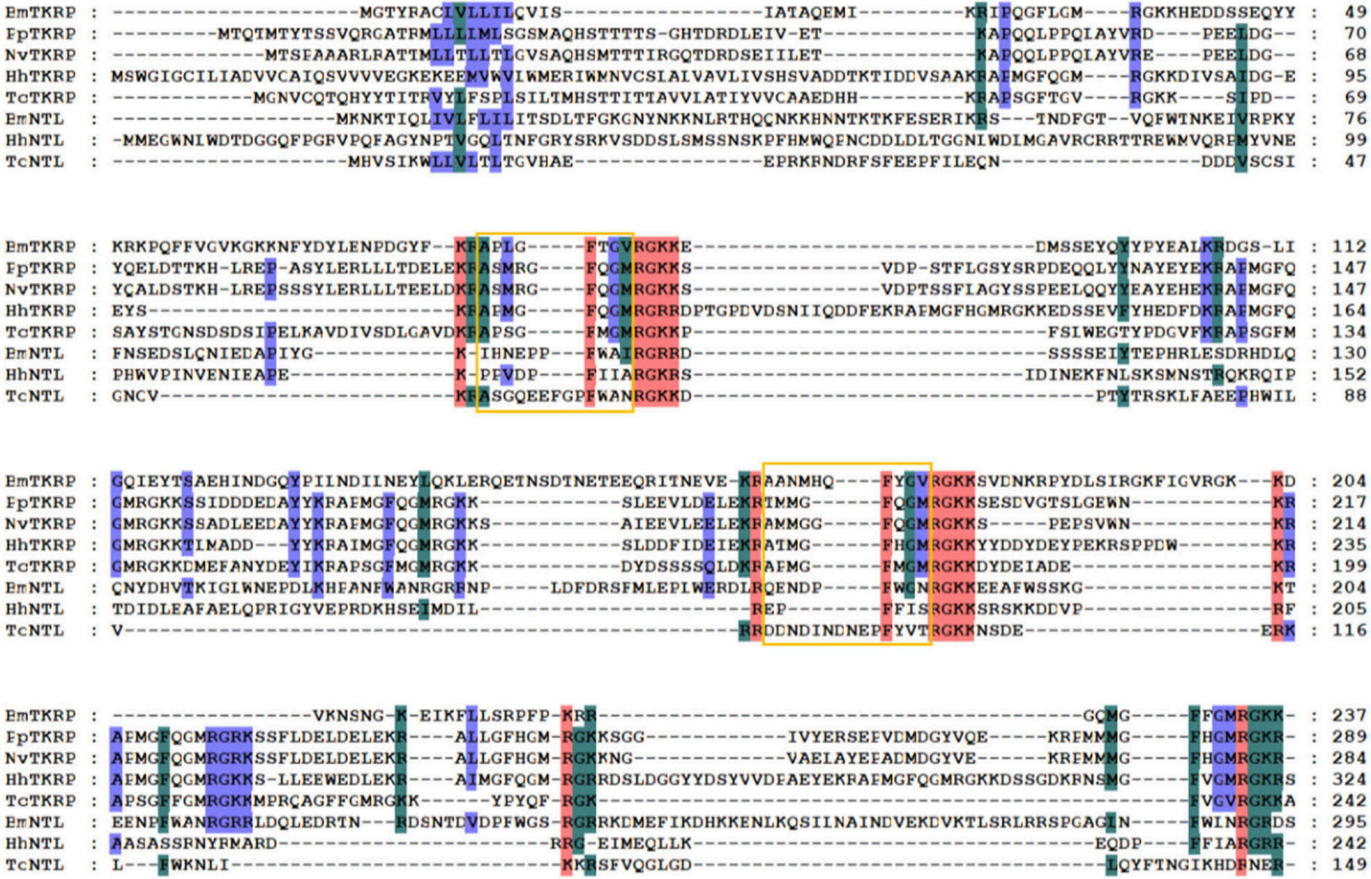
Multiple sequence alignment of the natalisin (NTL) and tachykinin-related peptides (TKRPs) from *H. hebetor* (HhNTL, HhTKRP), *B. mori* (BmNTL, XP_021207121.1, BmTKRP, BAG50368.1), *D. melanogaster* (DmNTL, NP_001163608.1; DmTKRPs, NP_650141.2), *N. vitripennis* (NvTKRP, XP_016836900.1), *P. puparum* (PpTKRP, PPU12373-RA), and *T. castaneum* (TcNTL, XP_008200697.1; TcTKRP, KYB25859.1). Amino acid residues common in at least four sequences are highlighted. The orange boxes indicate the mature peptides.

In contrast to *N. vitripennis* (Hauser et al., 2010) and *P. puparum* (Xu et al., 2020), we identified NTL in *H. hebetor* (Table 1). NTL is named for its function in promoting reproduction and modulating sexual activity. It was recently discovered and characterized in *D. melanogaster, T. castaneum*, and *B. mori* (Jiang et al., 2013). Being closely related to motif of tachykinin-related peptides (TKRPs), NTL is an arthropod-specific neuropeptide (Figure 12), that has a tachykinin-like signaling system (Jiang et al., 2013; Xu et al., 2016).

In our study, both allatostatin CC (AstCC) and allatostatin CCC (AstCCC) were located to the same genomic scaffold. Among various insects, AstA inhibits the corpora allata to produce juvenile hormones or block muscle contraction in the gut (Hauser et al., 2010). The neuropeptides from insects' allatostatin family C are myoinhibitory in nature (Pandit et al., 2018). Allatostatins were discovered as rapid and reversible inhibitors of juvenile hormone synthesis in insects, though AstC is a cyclic neuropeptide that is structurally unrelated to AstA (Stay and Tobe, 2007). AstCC is a paralog of AstC, and AstCCC was also regarded as an AtsC in previous studies (Veenstra, 2009; Xu et al., 2016). AstCC and AstCCC are likely generated by gene duplication, and the similarity between the precursors and receptors of these genes suggests they have a common ancestor (Xu et al., 2016).

Most insect neuropeptides are peptide hormones involved in modulating physiology and behavior, such as the AKHs, EH, and ETH. In *H. hebetor*, we annotated an AKH (Table 1). The AKHs are insect neuropeptides with 8–10 amino acid residues that are expressed in endocrine cells from the two neurohemal organs, corpora cardiaca, that are often fused and closely situated to the insect brain (Hauser et al., 2010; Li et al., 2016). AKH is a well-studied neuropeptide, thought to be similar to mammalian glucagon, which acts antagonistically to insulin by activating glycogen phosphorylase and mobilizing carbohydrates (Bacci et al., 2004). Some AKHs mobilize lipids and sugar mobilization from insect fat body during energy-consuming activities such as flight and intense locomotion (Li et al., 2008; Hauser et al., 2010). One axon branch from a pair of glucose-sensing neurons projects toward insulin-producing cells, thereby triggering the release of *Drosophila* insulin-like peptide 2 (dilp2), while the other branch extends to AKH-producing cells to inhibit the secretion of AKH, the analog of glucagon in flies (Oh et al., 2019). EH is known for its involvement in ecdysis behavior and is produced by a pair of brain neurons every time an insect molts in its lifetime (Zitnan et al., 2007; Li et al., 2008). In the CNS, EH induces the release of crustacean cardio-active peptide (CCAP), though it can also act peripherally on Inka cells in the epitracheal glands to induce the release of ETH (Hauser et al., 2010). A membrane-bound guanylyl cyclase was shown to function as an EH receptor in Inka cells (Chang et al., 2009). Respectively, located in different scaffolds in *H. hebetor*, ETH, EH and CCAP are the main players in the peptidergic circuit that controls ecdysis in insects (Wang Z. et al., 2018). EH is expressed in CNS, ETH production occurs in endocrine cells, and the CCAP is expressed in neurons (Kim et al., 2006). In insects, development and metamorphosis are coordinated by a class of ecdysteroid hormones, of which 20-hydroxyecdysone (20E) is the main one (Gilbert et al., 2002). Both the production and release of 20E from prothoracic glands are regulated by the brain-derived PTTH (Gilbert et al., 2002). We annotated PTTH in *H. hebetor*'s genome. PTTH is secreted by the brain's neurosecretory cells and released into hemolymph at a specific time and in particular developmental stages (Yamanaka et al., 2010). The amino acid sequences of various insect PTTHs are not very well-conserved, but they are all believed to operate by forming homodimers (Hauser et al., 2010).

GPCRs mediate almost all physiological and behavioral processes, by delivering signals from a series of extracellular ligands, including neuropeptide hormones and biogenic amines, across the cell membrane to signal pathways that elicit specific responses (Audsley and Down, 2015). Some GPCRs can mediate extracellular ligand signals to intracellular signal transduction proteins (Ja et al., 2007), while others recognize various ligands, including odorants, photons, neurotransmitters, lipids, hormones, peptides, and other small molecules, enabling the regulation of intracellular responses when adapting to a changing environment (Zhu and Roth, 2014). In our study, we annotated 44 GPCRs. From the phylogenetic analysis, evidently some GPCRs in *H. hebetor, P. puparum, N. vitripennis* are clustered together, but some are not. This evolutionary divergence could be due to different taxonomic families that *H. hebetor* and the other two wasps belong to. If a neuropeptide gene is genuinely missing from a species, one should expect its receptor to be disabled and no longer affected by positive selection (Veenstra, 2019a). Hence, when *both* neuropeptide and its unique receptor are absent in a genome assembly, it is a reliable indication that the particular neuropeptide signaling system has been lost from the species in question (Veenstra, 2019a). As mentioned above, inotocin may fit this situation.

In our research, we performed RNA-Seq at different developmental stages in different organs including the venom and salivary glands of wasps. This expression data set provides us with basic characteristic information on the neuropeptides and their receptors, which is required to functionally address their biological significance in ectoparasitoid wasps. As revealed by the expression profile analysis, ETH is expressed higher in the pupal than other developmental stages (Figure 9), which is likely related to its function. Both ETH and EH regulate the release of CCAP from central CCAP neurons, thus inhibiting pre-ecdysis (Wang L. et al., 2018). Accordingly, it should be expressed more at the pupal stage. In parallel, we found that the peak expression of *ATR* and *SIF* differ (Figure 11). SIFamide (SIFa) influences sexual behavior, feeding, pupal mortality and sleep regulation in holometabolous insects (Ayub et al., 2020), and AT has multiple functions, including the regulation of juvenile hormone synthesis, growth, development, and reproduction (Zhang et al., 2019). We speculate that this variation in the expression level of SIFa and AT receptor could drive from differences between individual insects such as in their body size.

To conclude, through this comprehensive study we gained some timely insight into the neuropeptide precursor and neuropeptide receptor genes harbored by *H. hebetor*. We anticipate that our data will provide some basic yet practically useful information applicable to future pesticide development and other research.

## Data Availability Statement

The transcriptome data has been deposited in the Gene Expression Omnibus (accession: PRJNA642006).

## Author Contributions

KY, SX, GX, XY, HY, FW, QF, QS, and GY conceived and designed the experimental plan, and KY performed the experiments. KY and SX analyzed and interpreted the sequence and experimental data. GX, XY, HY, FW, QF, QS, and GY contributed the reagents, materials, and analysis tools. KY and SX prepared all the figures and tables. GX, QF, QS, and GY authored or reviewed drafts of the paper. GY revised the final draft. All authors read and approved the final manuscript.

## Conflict of Interest

The authors declare that the research was conducted in the absence of any commercial or financial relationships that could be construed as a potential conflict of interest.

## Supplementary Material

Supplementary Figure 1 Expression profiles of neuropeptide precursor genes in *H. hebetor* across its different tissues. Log2 FPKM (fragments per kilobase of transcript per million) values for neuropeptide precursor genes are presented by in colored bars, where darker red denotes higher expression values, and darker blue denotes lower expression values. VG, venom glands; SG, salivary glands; VGC, carcass of female adults without their venom gland; SGC, carcass of larvae without salivary glands.

Supplementary Figure 2 Expression profiles of neuropeptide precursor receptor genes in H. Hebetor across its different tissues. Log2 FPKM (fragments per kilobase of transcript per million) values for neuropeptide precursor genes are presented by bar colors where darker red denotes higher expression values, and darker blue denotes lower expression values. VG, venom glands; SG, salivary glands; VGC, carcass of female adults without their venom gland; SGC, carcass of larvae without salivary glands.

Supplementary Figure 3 The qPCR analysis of relative expression levels of neuropeptide genes and neuropeptide receptor genes in different tissues. VG, venom glands; SG, salivary glands; VGC, carcass of female adults without their venom gland; SGC, carcass of larvae without salivary glands. The pink dotted line represents the FPKM (fragments per kilobase of transcript per million) value, and the relative expression levels from the qPCR are depicted in represented by the light blue histogram.
