# Supplementary figures and images for: Identification of Neuropeptides and Their Receptors in the Ectoparasitoid, Habrobracon hebetor
Source: Front Physiol. 2020 Oct 16;11:575655. doi: 10.3389/fphys.2020.575655 (PMC7596734; doi:10.3389/fphys.2020.575655)

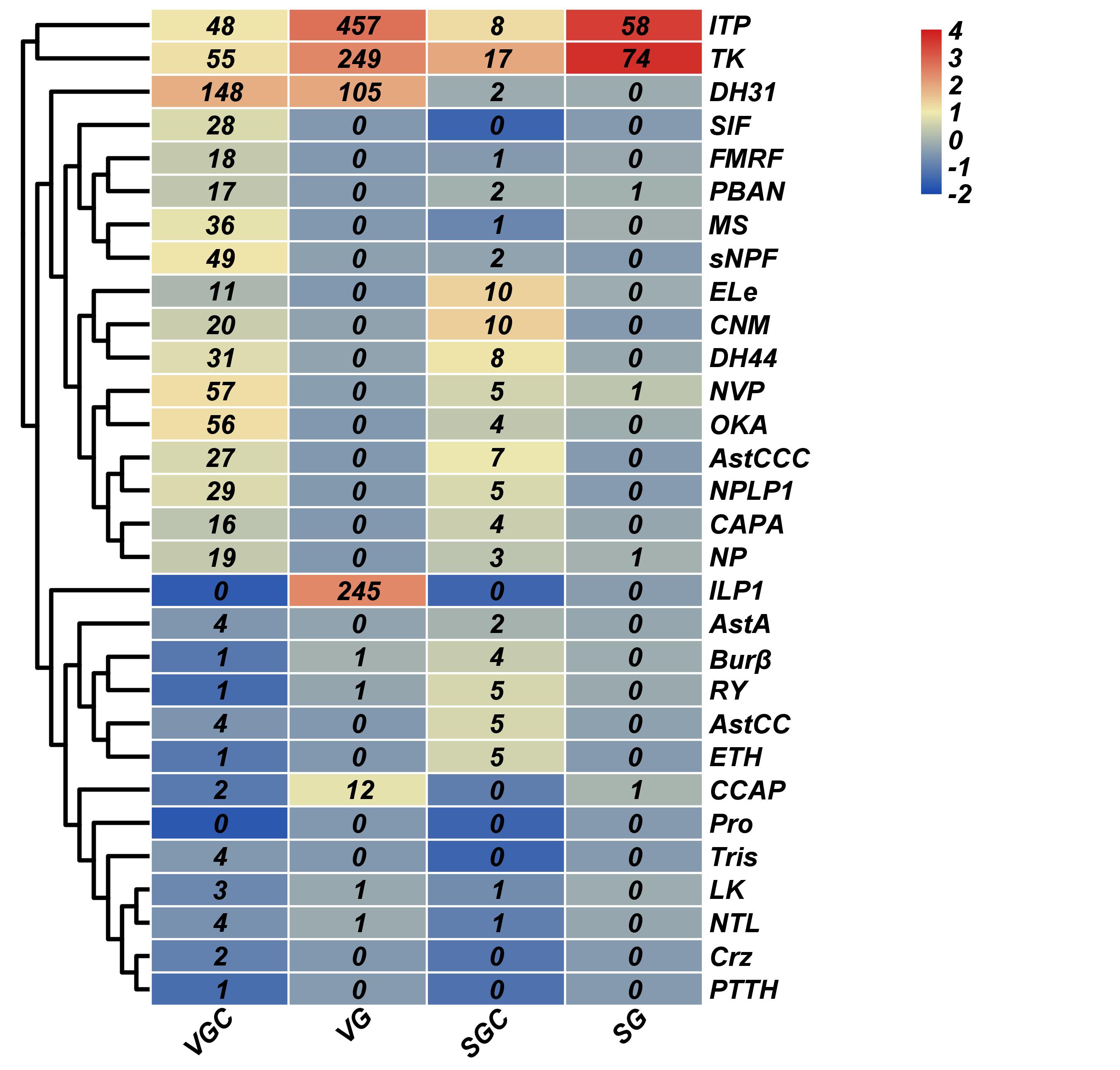

Supplement: Supplementary Figure 1 — Expression profiles of neuropeptide precursor genes in H. hebetor across its different tissues. Log2 FPKM (fragments per kilobase of transcript per million) values for neuropeptide precursor genes are presented by in colored bars, where darker red denotes higher expression values, and darker blue denotes lower expression values. VG, venom glands; SG, salivary glands; VGC, carcass of female adults without their venom gland; SGC, carcass of larvae without salivary glands. [file Image_1.JPEG]

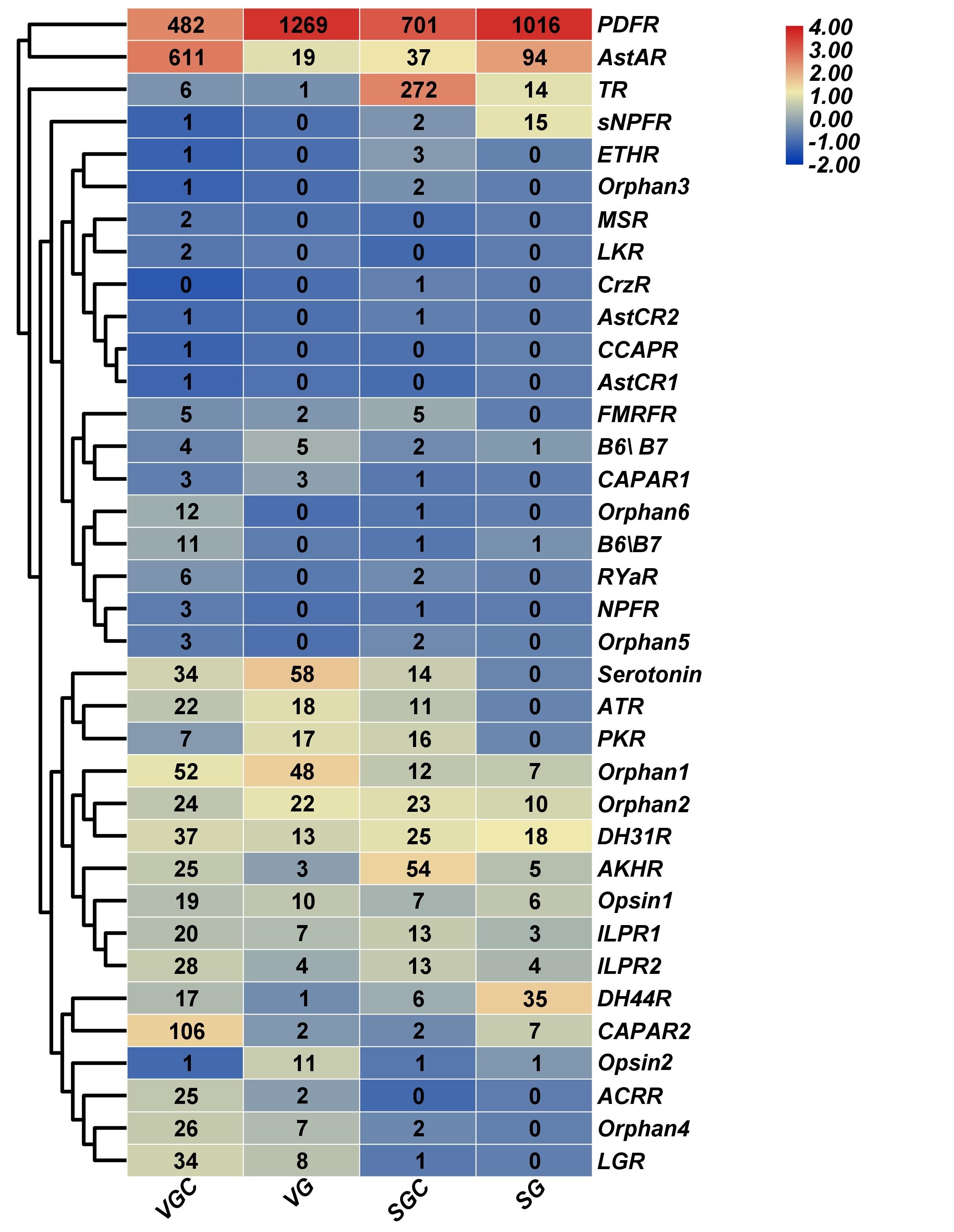

Supplement: Supplementary Figure 2 — Expression profiles of neuropeptide precursor receptor genes in H. Hebetor across its different tissues. Log2 FPKM (fragments per kilobase of transcript per million) values for neuropeptide precursor genes are presented by bar colors where darker red denotes higher expression values, and darker blue denotes lower expression values. VG, venom glands; SG, salivary glands; VGC, carcass of female adults without their venom gland; SGC, carcass of larvae without salivary glands. [file Image_2.JPEG]
