# Supplementary material for: Identification of Neuropeptides and Their Receptors in the Ectoparasitoid, Habrobracon hebetor
Source: Front Physiol. 2020 Oct 16;11:575655. doi: 10.3389/fphys.2020.575655 (PMC7596734; doi:10.3389/fphys.2020.575655)
Supplement: Supplementary file 4 [file Table_1.DOC]

The putative signal peptides (predicted by SignalP) are marked in gray, the putative active neuropeptides or protein hormones (based on similarity to neuropeptides or protein hormones identified in other insects and the peptides found with mass spectrometry) are marked in yellow. Green indicates putative basic cleavage sites; glycines used for amidation are shown in blue. Dots indicate missing N- or C-termini. Underlined cysteine residues are proposed to be involved in cyctine bridges. ITG and IDLSRF-like peptides are (together with the NVP) still very elusive, putative “neuropeptides” with no biological function or proven receptor.

>Hheb05007 AKH1

..MSIFPFQLPNRNIPHALRDTLPTHTHTPAPSTALPIENKPGPVNKQTATIKSSLLVQKNLIVMYYKNFPRILSIVLIISTILLLTTKLTEAQLNFSTGWGKRNKIFGIERENTNCPTTNSQNKPTLDALLNLYNLVR

>Hheb100640.1 AstA

MRYNGIKSSLQQRRMKRILLEIVVMLMLSLTIDTASSVMDDAMARLPVRGIHQRGCGAPKKDYNFITEYKRLPQYRYAAAYKRFPDYLYSFGIGKRSDEQSKRFSPYSFGVGKRNPLDKLILPQLLEAKNYDADFLIDNDPSQQTKRNAPWHAYGFGIGKRAAQYFNHQIENDNHQDLNNDMKINENNPLWDQY

>Hheb092060.1 AstCC

MGVSLVTLILMVGLTITTTVARVLPKRSIDGLSESYDYEDYSANNEPYPVANKRAALLLDRLMVALQKAVDDQGFGGDSSSIKGGGVIRTKPRLQTSNIGPLRSGGSKSGMDLQRRGHANGSVYWRCYFNAVTCFKRK

>Hheb092070.1 AstCCC

MASTRQLIFAIVMVYLVGVTIALPAGKDAFLTDLDLVDDDGSIDNAIINYLFTKQMVKRLRNSMDVGDLQRKRSYWKQCAFNAVSCFGK

>Hheb097870.1 ITG

MMSKSAVFFALAVAGCLLQMNGRVDAWGGLFNRFTPEMLSNLGYGGHGGYKSNYMQRPLTGHYSNNFAEGYQFAGDDDPCENRRCSINDHCCPAQICVDMDGAPEGTCIFAFGLKQGELCRKDNDCETGLMCAEVAGGDTRSCQPPVTSNKQYSEECLMSSECDITRGLCCQIQRRHRQATRKVCSYFKDPLVCIGPVATDQIKSIIQYTSGEKRITQGQHNRNSLLYKRGLFI

>Hheb01190 Burα

..MTKMFHVLRADGCIPKRIPSFVCHGRCSSYIQVSGSRMWQMERSCMCCQESGEREATVSLFCPREKRKFRKVTTKAPLECMCRPCTGIEEYSVVPQEIAGFAEEGPLTTSAHFRRSSSM

>Hheb109470.1 Burβ

MFWIFVVFMITGTQGTIISQDVDDNCETLMTDIHITKEEYDEEGILLRTCSDDLQVTKCEGYCASAVQPSIMTATGFLKDCHCCRESYLKERSVTLHHCYNPDGIKLTEPETSTMDIKIREPADCKCFKCGDFSR

>Hheb025420.1 CAPA

MKDHPILTALIFLTFATSFNRGEKINELIAKFNERRAAGILAQPRIGRNSGMGGFIRPDGAAGLVHYPRVGRSGMNDDYKYSQEFETDPKNHREIIDESHEVDDHKNFDDDFRKRLLNAIYLDNARLGGKKEHRPPCDNQGETGGVLFFTPRDMQKMERQLLHYYAPRSSSR

>Hheb092090.1 CNM

..MLLLQRLKDVASLKHEILEDERRLTEAELDIQAILEVKARNQRVPRPLESEVDTVGEGEEEEAAEMLPIPSAIVQHDPDHSGKRTSYMALCHFKICNMGRKRQL

>Hheb012400.1 Crz

..MFCTITCQTFQYSRGWTTGKRSDPSELGLPSDMRMLFPRHDDIAHCGLTKLRMLLRGNVNDRVYQLPCDLLRQLESSNEPRKTERQRFSEDYNNNNLINNNDNUNULINLKNULNLTRENGDGQIQ

>Hheb024270.1 CCAP

..MELPPKPKRPFCNAFTGCGKKRNYDENSLGSNSQLKFDLPPQIYKALIRALTDELRSAVARRLNEEERNSEISLQDYLAFLNMKRPVRSQMQS

>Hheb005670.1 DH31

MKKVTAIAWTILAVLVVGFGLVADAAPYPEERPLYLDQYEKDRERLYEMMSNLDRFIQLQDNEKRGFGLDFGLNRGFSGAQAAKHLMGMAAANYAGGPGRRRRSDRA

>Hheb091920.1 DH44

MLLVSLLVVSTLISLSKSTPYRRRDPDDILIDIPYIKGGSWDPAEYYGYSLQNEQTEVPKVDNNSDGTTARRKKMGSLSITNPMDVLRQRFLLELQRRRQMQQQEQAKANREILNDIGKRSARRLGMIYPRIQTRNKQVYQPAGPSSVHVASDNPLWSRLERFDWYNVENSGEHLNDAEQSH

>Hheb087690.1 ETH

MAKFARSRNDQLLIGVCLMFILSIGLILVEAEEEVPAFFLKIAKNVPRLGRSSGEFHDYLLKTTKDDKQSDYYNQDEPFVERQKRKINFHGSQEVTSDLDSSQWNHFPLAIEGPPDLWRTLARYANQKYGTSNE

>Hheb049790.1 FMRF

MVNCTIICGLLVTVNLIVAKGTVLTPLKLETSPVHVYKTMNGLENEFEYVLKRTGSQNADESPDSKERRSQMGTSFIRFGRNHHQGNLESPDDSGNLDAVSSRMARGRSDVIIRFGRAEFPSTFDARRGQFVNIPSDLRSLAFICPKISNIPQTLPHNDIIRICSLFASGSLGEDINY

>Hheb048490.1 PBAN

MFTISSACVIIIATIVANVSCDYDGIQGSSLGVKSLDGNGLCPGGRCVDQSGNGISGAMWFGPRLGRKRRSGERIETTDEDIGAIADVINSGPWSFVQYQVPSDKRHTTQFTPRLGRELPDDFLQRYLNYNLDSNHHYLDIADIENQRQLPPPPPPPFAPRLGRHLTFNPSPRLGRRVRFARKA

>Hheb05986 IDLSRF

MVGALSTSLAIGLALTACAAFPHSAMAIDISRFYGHLHSKRSGDACHPYEPFKCPGDGTCISIQYLCDGAPDCTDGYDEDSRLCTAAKRPPVEETASFLQSLLASHGPNYLEKLFGSKARDALAPLGGVNMVAIALSESQTIEDFGAALHLMRSDLEHLRSVFMAVENGDLGMLKSLGIKDSELGDVKFFLEKLVNTGFLD

>Hheb091810.1 ILP1

MNLTIHALLAHLIVCTVVTHGQKAAKKWPIGVVEKLHTFGSRLMKNRARDKSEIAAKIKEASTQIKELQGSLKMVERTILNEIDRLLNYHQIMHELKTSGTYIDNMFRRFELYDDDTTYEDSTLKDFAQSSTSHSKGHLLSMMNLIYRLVNPVGNDNKGLFDLIMKDKTTKLLQITGICNKTESPQQTAFLLYNLVSFYMIKGSVIRLYSYEMLKELGLGHYNTEIAGVRLEYDKFMKNIGLRVKETMKTTSRDIRPCDAKIHKEGETYLQLKWLFGSMYTNEIFLSVDKNCGKSCSDYTKADFHPNNFPGRPERCYGSLYRCFSEDSDYYCEATVPYRRYSGIPAQTLYGMYGEKTIAARGNVTDCKKKELVPDEQRVPPQKVGDSYDYCNECVCSCDDYNHPETVRTFSLREVSSDTDENMVITGVRFVEAQKVFHIQIQQGKLLPDGTIDVKTITWKPVDEFDYRDPSVPTSDYYTVTALNNAVALARFEMVNGKVMAGVRLAKRHSMLTVEMKTCSMHNDYLSGCGHWLISRRHSNPPVFTRSFNWDSKVQLCGTKVHNMLRLICGERGFYGSVSDFNRRRRHVKRGIVDECCTQPCTLANLELYCKTEEDEGNYDA

>Hheb087940.1 ITP

MIVTSICLVSLVITIEVIPPDILEILEQEWIFSYYNFQKLSCKGNYDTQIFAHLEKKCDDCMNVLNLTPRAYVHCRWRCYSTEWFTACLKGLQYADEYEKYAGYVNELSGRELAPLVALADQQCKSSPCITIDVYYESLCPDSIRFITRQLATTYNGLKDHLNVNLIPYGKANHYQDTNKNWVFTCQHGPSECRGNKAQACGLDAISSLNGASFEKKQSLSVAFVNCVMDAINPASAVPKCAETVGLDTDTRTSINECIRSDHGNALLASLGDKTHALRPTLSFVPTIVLDNVYSQENQNKALYQFKKLICDRIPLDNKPAVC

>Hheb035510.1 LK

..MIRQCTDCARSVLQSGLIRQILSMRRQENSIIQHNSLSYLRRLLLLYINFYFFYIYTIQFIRPVGQRRNHALVIKERRYPSFSPWGGKRDSSVYKPGLKIRRPVRVPFNSWGGKRNGGASVIKTPFNSWGGKRSSRLIDHILTTATGMDEPLRGDWRYSVINNPELVGIDPMNLAMSNLDEIENHQDYSNLEDPSANFNSHLPRIPFGPWGAENNENNEESDEGDVSSTSVFDSE

>Hheb029860.1 MS

MSSRVCFLFTLTLALLAVLPGQTYTMPPVQCKPGLLEGVPPRIRKVCDALTTIYELGSAMENYIEDKVPILHDNIPLPDSGVKRQDVDHVFLRFGRRR

>Hheb035520.1 NTL

..MMEGWNLWDTDGGQFPGRVPQFAGYNPTVGQLTNFGRYSRKVSDDSLSMSSNSKPFHMWQPNCDDLDLTGGNLWDLMGAVRCRRTTREWMVQRPMYVNEPHWVPINVENIEAPEKPPVDPFIIARGKRSIDINEKFNLSKSMNSTRQKRQIPTDIDLEAFAELQPRIGYVEPRDKHSEIMDILREPFFISRGKKSRSKKDDVPRFAASASSRNYRMARDRRGEIMEQLLKEQDPFFIARGRRAINSF

>Hheb096350.1 NP

MLTIQSLGIITLLAILPMEIMSHPGIRIRDVEQSTCRPCGDDCDYCEHGVTISPICGVPECKRGPDEHCGHNSGVCGEGMMCMCNKCIGCSLYTLKCSVTSPEHPCLNRERRFHNYEHSHTFTLV

>Hheb000030.1 NPLP1

..MMENTDQSNESVVDQEKRSIATLAKNGDLPASIQEREQGRREEGEKRSRTSITPEEVLKEYLGEASDNIDSQTAAQILAKYTLPSGELDIQALRRDFGTTKRNIATLARDYALPSNRRTLATMSRDFTYPYLKRNIGSLARSSLLPAGKRSQYTMSRFYVIPWLEAKRNIGSLARDSAIPRYGKRGIANLAKNGDFPMPKKNVGTLARDWSLPQTRSERSISNNPFVAELNDIKNRSRDKRQVDYSDEYPLPVMQNTNVLDYEDVIEALANGFPSTAKRFMETGGLQNKWQDIDDERVRLSHEVPYQPSKRHIGALARQGWLPSFRAARFSRSPREDSSDGPTPNDTINTSFRPL

>Hheb068000.1 NVP

..MATGEEVLAHFWIVQCREKTLQHDSSSFSIDFVCKVKFGTSYGRVVVDEMREELPRNWDILPYSRYFGMEEDRRKRSEAVNKDAESGVVTSDSPLAPPTAASSSTSSFSPMIVPASGVQLTATARPFIQAKRSMQTLYPEPRYKRALDRDDFLALLRLWENQPRAGARSWRNLMNNEEYDNMEDEANYIGQDLDEDARVGADWLDSGSLYAPSRHMNLGSEISGPSEFGIPRTHPFNSYDQYGVQYNTPTYTDNSQYGTLLYPHATYFAPEKRFIVSRKRSQAYDTYGARNINNIIGFSQLMNSQAQGYPNNLPQRLFY

>Hheb092110.1 OKA

..MHDEATGLTEAVYTEPELIEALARSYAAMGSIRSGSGNMRDSRIVRQSRRGLDSLSGATFGESKRFDPRRKPELSNVIPIEYYNEGIKRNMDEIDRAGFDSFSKRNFDEIDRTGWDSFVKRRFVDAYLRQQQH

>Hheb088460.1 Pro

MSTIFLVLWLFSFQQGIGVRADKPEGILERIKNGLQYAKNYLDTAKQIADLVSESLGHNNYKQQNSDNDKLNKSSKRLDPGLGSAFFRLLGLDSTKIAAIAIGSLFNIQPDPKKVGSGRDFINEFDPLNFIKNTKNEKFNDLLKQAQSPDLPRRLIDNIDGFDSECIKLLICKSSPVISAVQASLKNKTGDKRSQMTSWLPRREIFERNSDSCEEKHRDCKIFPEMDMEKGDW

>Hheb092100.1 PTTH

MRIIMIHITLISLVLRGDGKEFNYARWPTIIDPDSLSLNGDCTGPGCNSLYTQKRSGNFDAMEQMIPWMCPCETRYGVLDLGPENYPRYLAHANCVPKPCHTKFNQCKLMHYKVHVLREREDNDANDPSDAQYIEQSILPESLRVKWQLKPMKVAVACVASA

>Hheb04319 RY

MDMFACFWVILVLLANVAFAENNFYTGGRYGKRDDAPAVHSAGAMPFFSGSRYGRSNTNTGSDTIKNVEISPRPDRFFLGSRYGKRNQQQSPESAAGDAAASLRRLEAVLNYLSRVRRLDEPQQQHRQPHNYEIEQTYYDNGYDDNDQKDDTDNQVLYGK

>Hheb073990.1 sNPF

MKNYALTTIALFLIFGVALGTENYMDYIADENANRDLENLREFYTFLFRRNQMDNNYPGGNTANGLVGIPYEHLMIRKSQRSPSLRLRFGRSGPVMPPPEGVLTRMRSSSPGTEDN

>Hheb087710.1 SIF

MVSPRVFLAIFFIVVVIVSLEVDAAYRKPPFNGSIFGKRSNTVTDNEMINRALSNMCEAASEACNTWFMHQDSN

>Hheb033240.1 TK

MSWGIGCILIADVVCAIQSVVVVEGKEKEEMVWVLWMERIWMNVCSLALVAVLIVSHSVADDTKTIDDVSAAKRAPMGFQGMRGKKDIVSAIDGEEYSKRAPMGFQGMRGRRDPTGPDVDSNIIQDDFEKRAPMGFHGMRGKKEDSSEVFYHEDFDKRAPMGFQGMRGKKTLMADDYYKRAIMGFQGMRGKKSLDDFIDEIEKRATMGFHGMRGKKYYDDYDEYPEKRSPPDWKRAPMGFQGMRGKKSLLEEWEDLEKRAIMGFQGMRGRRDSLDGGYYDSYVVDPAEYEKRAPMGFQGMRGKKDSSGDKRNSMGFVGMRGKRSIYDSDEEEYYKRVPMDFLDSRYVNDATHFDKRASYGFFGTRGKKIPWEIRGKFIGVRGKKWTPEALASSNDNLINEVLLDDIERIGAGSTTSFGFFAVLSVPTHARPQFQNDMSQADGFIFDGPADRPNNFPPNGISTTSRPWQPTQPIDVPPTPNPGNNNNNNQFTTSTTQSPQVARCIRDCPVTSEYNPICGTDQVTYSNPGRLTCAQACGAPV

>Hheb066920.1 Tris

..MRIKRLDGDHLDPSRSIRRLDLELAVCVVLALGGSLSCEDCGRECAHTCGTRAFRACCFNAQKRAPNVGLRVWFTPPEEEERLRYVYDS

>scaffold4 ILP2

MFIINRCYSLLLLVVIINLLIDIGDAAPHQTSFRLCSKSLSDALYLVCNGIYNEPFPDSGSADTGPQSGTGIVDECCHRSCTYEQLKMYCKPSAS

>scaffold29 EH

MFYKFSMIDLRKHTILMIYLLALIGTCIRNCAQCEKMFGVFFEGELCANSCIKHKGLSIPDCEDINTIVDFVHRPEHFDEDD

>Hheb109520.1 Ele MSLRQFIIILSIFAWCAYVEAARAARPLDCEKYVFHPHCRGSQARKRFITTMKSDNNEQPCYCAGKDDRVAITNAKILEAILSNGFDVNTIYDAYATSSDRHRDYNDNSRDRPGQRRRSSLDSPVDGLNIDSEVDY
