# Supplementary material for: Identification of Neuropeptides and Their Receptors in the Ectoparasitoid, Habrobracon hebetor
Source: Front Physiol. 2020 Oct 16;11:575655. doi: 10.3389/fphys.2020.575655 (PMC7596734; doi:10.3389/fphys.2020.575655)
Supplement: Supplementary file 5 [file Table_2.DOC]

>Hheb026340.1 NPF

MDETRRQLEALPDKYFEKIAHILMDLRNDTVDLSKPHLRISFANNYAFFIFLYVVMITMGVAMNIGMIYHIVRHKLYHDPTYAYLINLAISDVVKCIFVLPITLAVLMIHNWIFGKFLCFFLPMLQDIPLHVSMMTYLLIASDRYRLVSDPGKPRIPAFVVALGAWFFAVCIVLPYAIYTSYLDLTMYKKPTLHGFGICMVNLYDDIQQYMRCLFLFTYIAPLTITAYLYVKASRELQNQEEPMAVAMFEARRKNSYSRHGSNTSNDITSFRDGKRESGSVMTGGTVGLSGLSANYDLYDAELDVRKEQRTQKYLIFMVSVFAILLCPLMVLRLAKPALLETYENTGHFDITFIMFVWMAFASTVTTPLFYASWQMSRPAKERLKGYFQFSTKRLPPVLEKGLRHHGNRHQSGLANVAYTPQARNGSLSGSNGGEDYSRGNSTFHSPDLGNNVHRMNLVQ

>Hheb109890.1 CAPA

MKSKDLLSLNNTTTMEPVDTGGTEIFNFSFFFNSSEEEYLRLLLGPKHLPMRLVIPITVVYVFIFVMGIFGNVVTCWVILRNPVMQTATNYYLFSLAVSDLMLLILGLPFELRVFWQQYPWELGWGLCKIRAYVSETSSYVSVLTIVAFSMERYLAICHPLHLYAMSGLKRPLRFIFAAWLLAMIAALPFAAYTTVNYVEYPPGSGRNSEESAFCAMLLHNMPGFPLYELSCLIFFLVPLILIMVLYIRMGLRIQNTTLGGSIEGTVHGETRQAQSRKIIIRMLSAVVVTFFICWAPFHAQRLLYVYDRTSSFGDVNEWLYFLGGCLYYISTAINPILYNVMSVKYRSAFMETLCCTHGGNSLNRDDQSSMKETTIYRCGSCKSSQIARGRSKSVRYHSENFRDLLHTKPPDNTNDNFRPERHLMNNSNSLINNHETCLLESRKLSTVVHTSNGRAKCRTTDINGSPDETHI

>Hheb109910.1 CAPA

MDRMNLELLTSEEEREFEEFWDNLNNQNLTEEEYLTRVLGPKHLPAKLVIPLTLAYVTIFVSGVVGNVATCFVIIKNSTMHSATNYYLFSLAISDLILLCLGLPNELSSIWQQYPWPLGLGLCKIRAYVSEMSSYVSVLTIVAFSMERYLAICHPLRAYAINGPRRPIIIILAAWMIAIVSAIPFAIYMKINYVEYPPGSDKNSADSAICAMLLPDMPHFPLYELSCVVFFFIPMLIILVVYTRMGLKIRASTRNNAATRPGESSAHWDSRQVQSRKSIIRMLSEYLLMH

>Hheb023220.1 AstC

MNSTDFLDNPFNLTNTSCGVNVPIVAAVNQVLYSIVCIVGLLGNTLVIYVVLRFSNMKTVTNIYIVNLAIADECFLIGIPFLVTTISLGYWPFGQTTFTLYSFIFGFAIPLTLILIFYVLVLRKLRTVGPKNKSKERRRSHKKVTKLVLTVIAVYIACWLPYWVTQVTLIFTPPMQCQSMLTITIFLLAGCLSYSNSAMNPILYAFLSENFKKSFLKACTCAAGNDINAALQIENSVFPKKNKQRGERGQFNKMTTSVTSKPEMDDEEGERGLLICKSSTTNITMTSRSSIPMACDKDRNGGVENGIQETLLSSGGERGLIICKRPNTDIIITSKSGITIAGDKESEIKNDSQEQLMSSTDKQNFIICQSSTMTNKSGMPIEEREGGVENDNQEILLTEVURERERAYQS

>Hheb002560.1 Orphan

MTDSMSMAARIAEAANRTVIEGEMSRFPKPLRTFAAVVAILIMIVGLAGNLLTIVALCKYPKVRNVAAAFIIRFIDSGWTDVRFLCVLVPFLRYGNVGVSLLCVAAITVNRYIMITHHNLYGRVYKKHWIAAMILFCYVFSYGMQVPTLIGAWGKFDYDPNLETCSIIKDSNNRSSKTFLFVMGFIVPCIVIVGCYAKIFWVVHSSESRMRKHASPTVKSPHTPGRDTREIKQRRSEWRITKMVLAIFLSFVVCYLPITIVKVADPKVQYPAAHVMGYLLLYFASCVNPIIYVIMNKQYRQAYAGVISCSRIRATLTPHGSSVPGQNNYGQGNHIVTVTKL

>Hheb005840.1 ACP

MKPKLAASANDPLDPKNEARLKDRAVLTPIIKYVIPTFLIATSVLAGVYVTMLFLSFNENEPGITTTCDIQLAFRIDCLPGLEAIYGDCMDAGCCWDESDKFCYHTLPSVHTYQAVKHENYWALHTKNKLSPLKSYNKPSLQATINKIDNGYVEIKLTTTPSIQSMRHEIEKGAPAASPSNGNSTGHDSILDDLINVMIHEPTFGVTLTRVDNLTKAFPILTTSRGPLIITDHYWELSLYLGNSNTTLYGLNSGELNSSINWIYNNKGGRVMSNILGITSKSWIVGCYIDSQGPMEIEVLPSNLIIVRGLALPKDLSLHVFIGKEPEEITKRFVALHNNQLEREPPIPESFGLHICPDDTPKEMLSDLDKVIKTMDDYRAPWDTHCIYKKFRSTLDQKMSISDAEDLEDIRKKLEGIGRKIIHHISSMCSYGKSSLPFELANATMLLENTYGPYVGSIDSESFVYPEWRDERIEEVYNSVIEEYLHEAPVSKSLYVRDSWPRDDSNYTTAAFEKFDYLPKELRALMSSGTIPIDVHSNITSFHYKHHNEYAEKFDAFVGKYAYPMNVEEPNGESIGGWAALKNTLKRGIASGPIGQLPPAIYVCNVTTMDEGNLCTRWYGLAVAFPHILARPQNIPGGELLNPGTSKYVAQLLRLRTSFTLYQQSNIMAYFHNGASILSPTHYHYPEDTATRYTPDQFMWGPSVLVGLVTSPNIYQLQMAIPGEEPWRHILGGLIVHPSKVSISVLEGEIIALLRPGHIIPFHEETALTSMATSQRPLKLICNLACFDDKCNAQGKIFYHPNLYIQVNVSKTEIYLKTYNDGLVDCNIQENITIDTVRFLGIVISKSSSGTYVSNGASISGSRLRLRRSDMSSIERARSRTLKMTITIVAVFILCWTPYVAMLMWYTFDRQSAENVDPRLQDAFFIMAVGNSCANPLVYGSYAIDFRKECCRCFLPYPTAPKIDALELTQRNTGKKVQVTKIPSPGVSSLLVRSIRHAVPTYLRVGSLRTRSTGVSCNEVIPSSSPKLISESFSSKSLPLHEMKHEKSHNFLSVPGDDVLKMSTSSGIVSVGQSA

>Hheb011130.1 CCAP

MTRILDNLYSNKTDGNFTQLVGDFNLSDFIIRNELPNGSLADVNATDIDPFYFYETEQFTVLWLLFAVIVAGNVAVLAGLLLGKRRKSRMDFFIKQLALADLLVGLISVLTDIVWRSTVTWYAGNIACKLIRFSQVVVTYSSTYVLVALSIDRYDAITRPMNFSGSWWRARVLIAAAWGLSVLFSIPIIFLYEETVVEGKNQCWIELSSPVKWRVYMTLVSFTLFIAPTIIIGGCYTVIVATIWSQSSVLRQGPLRDRRASSRGLIPRAKIKTVKMTFVIVFARSENFPSIASLPSNDALHDKDFVTVLQDQIKVMKARTTALYHENVHLKTQIHQERMTSKNLEERLDQMKEYKNIGECKNEWTNTEMRPEEMTIKVPFTCHACSKTIVNDNDDDDDGDPMVFITKTELNNLEKDIKELRESLQLRENSWDGMVEREQNYSRQLTRLAQEIMTVNQLVENQSNDIENLSILLQAREGELKSAQKDIIGLQKLVVRLEKRNKALKENGGEKTMTEMNERDRKWIETIVRQVSTPRGRQKSKESYYSTPRSVKNSGRDETTTIIDT

>Hheb035150.1 TR

MRGTTNFFLANLAAADLCVGVFCVYQTLTNYLMNSWQLGDFLCKTYMFVHALSYTASIMILVVVCIERYLAIVHPIRCRSMLTRGRLRAAVVIVWILAAVYASPRFIYVETINHKLNSGSVDIICIANIRKHNKNVLDAVNLILLYLVPLFLMCCLYTRIALGLWKSGEAFGGPGLVARTRNGRVHHIHASSKNVLRARRGVIRMLIAVVLMFAVCNLPQQARILWLHVDPNYDRGSDFSTIFTVSTFLISYTNSCLNPLLYAFLSRNFRRAMRELFTCHNHNPSRAFGMGYVPGDAARLENGHTANMPHSSVIRLSSVHDSPCTTHTIARQGTLVKTSNENKMSINLRSVLISDPVDESCGALLASHGVPVTTKYKLSKEELIREIQHHDGLIVRSETKVTGEIIAAATNLRVVGRAGTGVDNIDLLAATRSGIVVLNTPGGNSISACELTCALISALARNVAQAAQSMKEGRWDRKLYSGFELSGKTLAVLGFGRIGREVALRMQSFGMKIVCFDPMLDPEVAASLGATKLTLDEIWPIADYITVHTPLIPQTRNLINATSLGKCKRGVRIINVARGGIVDELALLDALKSGQCGGAGLDVFAEEPPKNPTTLELIQHPKVIATPHLGASTAEAQQRVAVEIAEQFLAISGITDKYTVTGIVNAPILSAAMTVENGPWIELSKKLGQLAARFLKKNMNAPIESHTVGAGLQNKKFIHTAVLVGILSGQTKNGLNLINAATLAKDIGINVKEAHVDGEVDAVIIKIGNHQIKGTVRNNEALLLSVDDAMFNNGIVLRDFISLYHANGPQDLVTIVNAFSSKGITINSLNANGNWLVIETDQNVTIPIQGIEAF

>Hheb039010.1 LK

GAQPRMNTSWTAGNGESDLIWESDSNYSDVYNDSSIFEDSDELYNVPTGIIFLLSLLYGSISILAVAGNSLVMWIVATSRRMQSVTNFFIANLALADIVIGILAIPFQFQAALLQRWNLPYFMCAFCPFIQVLSVNVSVFTLTAIAVDRHRAILKPLSARPSKFCAKIIIACIWFLSGALAAPMAIALRVVLVPESSTGGRMHLKPFCQNVNLSEGSMITYRGLLGFFQYLTPLAIISCVYARMAFRLWGSQAPGNAQYSRDANLMRNKKKVIKMLVIVVTLFAVCWLPLQTYNVLQSTCHRINEYKYINIIWFCCDWLAMSNSCYNPFIYGFYNEKFKREFQQRYPFKSRKWSTSPPPGSLDIEKTMSTRTSLSVANIITTQNPNNTFKYSVNIAEGIERSSFNN

>Hheb111350.1 Crz

MELEHAPTLTQHAIIKAIVLCVLVADLFVSVFCIMGDAMWSYTVNWPWGNVACKFFKFSQMFSLYLSTFVLVLIGVDRFVAVRYPLTSFNYPGRCQQFVAIAWILAFILSIPQAKYRMQILGEQYY

>Hheb063160.1 RFa

MADEKYFDNFTNDTNTTYDPYADYDIEDSFNHFDWEELAPVVVIYSITFCLGLVGNLVIITSTLCPKLRPLPSTPTNIFLGGLASADLILILFCIPVKVAKLFSYSWTMGWFLCKGVHYMQSVSAICSVLTLTAMSVERYYAIVHPMRAQYTCTISQARRIVVITWISSFFLAIPIIFVQRHKPVGWRYPAFYCVRDDSQPTYWRAHELYMLLLVLVVPLVVMAFCYTAICWEIWLVMKRRYHMTSRHALNPSMNNNNVTNGECIPMTDRRRSTERSRRARSRREDTTTDGESRTMKQVVKMLVAVVVLFAICWSPMLIDNVITSYGILSQSKQGTVKHLNTAFQLMAYFNSCINPIIYGFMSKHFRESFLAAACGGWWCCFRRRVYTPPVKRHPSLSQTRTTSVSHFYLDNLLFSSQVRLIAPSFSENSKNFIYHCLTTIQIKGGLDAALPVNIDHPFPLSLIVSNSLQKIKEQQQQKISSPNLNN

>Hheb073560.1 PK

MRQGLREDRSAIDTFPIGINKDLGGDSLNEISQSIHENYTSQLAQLMTDDEFNQTTPKRDALYIVVPITIIYFAIFLSGLIDLLLLVSGLPPEMYYIWSHFPYIFGEIFCIIQSFAAETSANATVLTITAFTVERYVAICHPFRSRTIPQLSRVVKYIIVIWVLALCLAIPQAIQFGITFSKTLNGTIIPDTATCSVKWVIIKHAFEISTILFFVVPMTIITVLYGLIGIKLISSRMPGADKRKRPEQSNSQDSSRSGVPNEKNVLRMLVAVVVAFFICWAPFHAQRLLAVYAKSLGDGGSSLVTVYTTLTYISGIFYYLSTTINPLLYNIMSNRFREAFKRMLAEHCGGRRSIEPASPRKRTYSDLSHGRGPVGKRPEQNSGSFSASDETQHLTPLVRNDEIEQASSHQESSVLIAKDSIGRKQLSSDVTPKSRSNDSSDSSQIIIVTSLAKGLDEACHNKGGLSHSTVNKCLKVQRPIKAVTLGLLAERLRSGTKGLFAHQQRQTRSSKMRVDSTIQRVQQKMQSHPSIESANTISNSSLQDLDETEFTGSELAKYMGEINFELVT

>Hheb044860.1 AstC

MNSTDFLDNPFNLTNTSCGVNVPIVAAVNQVLYSIVCIVGLLGNTLVIYVVLRFSNMKTVTNIYIVNLAIADECFLIGIPFLVTTISLGYWPFADRYIAVCHPITSPKMRTQCISLLVSVTAWFTSALFMVPIVLYAETKDFPNGGNCNIFWPNNYGGQTTFTLYSFIFGFAIPLTLILIFYVLVLRKLRTVGPKNKSKERRRSHKKVTKLVLTVIAVYIACWLPYWVTQVTLIFTPPMQCQSMLTITIFLLAGCLSYSNSAMNPILYAFLSENFKKSFLKACTCAAGNDINAALQIENSVFPKKNKQRGERGQFNKMTTSVTSKPEMDDEEGERGLLICKSSTTNITMTSRSSIPMACDKDRNGGVENGIQETLLSSGGERGLIICKRPNTDIIITSKSGITIAGDKESEIKNDSQEQLMSSTDKQNFIICQSSTMTNKSGMPIEEREGGVENDKQEILLTEVURERERAYQS

>Hheb116660.1 AstA

MDEKISFNATRWIDWMDQIGQNSTNCTINESNCPDYDELLIKRVVKVVVPLFFSPIGILGLVGNSLVVIVIALNPGMRSTTNILIINLAVADLLFVIFCIPFTAADFVLPYWPFGDLWCRMVQYLIIVTACASVYTLVLMSLDRYLAVVHPVASMTVRTEHHAFLAICIVWFVILTASIPVLLIHGELTGAPEEEEGEKQKVVNAPKSPLLCSCDKGVGTWAIFDNDIADPFNVKILYSGLIQFVHEFGRGEKSAWNCTPNENDKKKSKYMAIMFLKYIIYLPDRSVESIDVNSINMLRLHLFLFTTLLAGFADDAHAASIFTHYNDEEHKGTDLCHSLVFCNSELLKTVQLANIFNDSKTFVDHYQLNDPSVTLANFERLMSETNRKPSKDQIAKFVAENFANTNEVLPWNPPDWQPNPPILERIEDPNIRDWVKQLNGIWKNLSRQMSPDVLKHPERHSFIPVEHGYIVPGGRFQELYYWDSYWGVEGLLLSGMTQTARGIILNLLSMVERFGFVPNGGRIYYLMRSQPPLLIPMIEKYVEATGDIEFLADNLLTMEKEFAYFQREKTVDIVKDGKTYRMARYIVSSQGPRPESYREDYKLAQFFPEAQRNTLYEDLKAGAESGWDFSSRWFVTDGDKVGNLSNTSTRSIIAVDLNAFLQRNARLLAEFNKLLGNRVKAREWMDVANAYQEAIDEVLWNEQFGIWLDYNIKNGQQRHHFYATNLTPLYTKSFNASRAAYYAKRTVEYLKSQGIDDFMGGTPSSLSETGEQWDAPNAWAPLQSIIIQGLYNTNAEPALSASKELATRWLRSNYLGFERYNQMFEKYDSGNPGHYGGGGEYIVQPGFEFTNGVVFEFLDINAKKLSSTIPIFSE

>Hheb090620.1 Opsines

MSNFLSGGGARKRELKVTKMVALMVTAFLIAWTPYAAVAIATQYFHWQPPNSLGVLPSILAKSSICYNPIIYAGLNSQFPQSLRKLLGMKQSRTRSQGPASDVTMGINREKVFE

>Hheb007010.1 Orphan

MAVAPINKILTALATADMFVMIEYIPFSIYYYIIFPNRATFPYFGAVFVLFHMHFAQLLHTISIALTLSLAVWRYLAIRFPQHNHAWCSDARCKMALWCSLAVSAFACSPSYFVFEIHEQPVQENGITEILYYVNADSYSGSGIAYQINFWVLAVVVKLLPCLILTVISCWLIKELYSYALFLLALFRAIYSKEPGEENKGEIME

>Hheb077040.1 FMRF

MKSSINYLLIGLARCDTVLIITSVLIHGLPAIYAYTGLLFDYKFGVFPQIVRYLYPLSCMAQMVTVYLTLTVTMERYVAVCHPLRARAFCTYGRARLAVLSIVIVSIIYNMPKFWEVDLEKEIHWKYNVTVYCVVPAILRSSDLYITIYVNWMYFFVYYAFPFVALVVFNVAIYRR

>Hheb089310.1 sNPF

MEDPSIMNTEIYDESMFSGNLTVPEDYVTSLIWVQAIFYFLYGLIFVVGIFGNALVCFVVVRNTQMQTVTNLFITNLALSDILLCVLAVPFTPLYTFLGRWVFGKTLCRLVPYAQGVSIYISTLTLSSIAVDRFLVIIYPFHPRMKIKVCLSVILGIWVVALFLTLPYGLYMSLEEPQGRVPLCEEHWPDPTFRQIFSSFTSILQFVIPVLVIGFCYVCVSIRLNDRARHKPGTKTSRREEADRERKRRTNRMLIAMVGIFTICWLPMNILNIVDDFNVDISNWAYFRLCFFITHALAMSSTCYNPFVYAWLNDNFRKEFMHVYNFDYIY

>Hheb077620.1

MVRLLLLCFLAGALGYVDGHGCPAPCICKSVGPQNERLRVKCNKDIQDIKEINVNSVSIELYHLDLSKNSIYIIEPGIFQNLTNLRRLDLSINKITALEEGCFSGLENIERLDLSKNRIASIDALVFRQLKNLKKLDLSGNKITTVETNLFHDLLALERLKLNGNLLKTLSEGTFHGLKLLRQVDLTNNPWDCDCYLYWLSNWKNTSLFKLIPAPTCASPPPLHGHSLLDLRFSDELQCQFTSPIIDLQPDQNQVVFAGDSMTLHCSVPSITDDRSARLKWYWNPSIFEEAGAFVDPQDTLSNIKVENRYLSDSGAIDSSITIFPVTKEHNGQWNCELTSVYGNRSKTISMIVISDETKYCPLVITRNNKGMYAWPRTVVGWRVELPCEGLGLSGLVPIPLRASYHCNATGSWIDLNTEACPFISPITKALEQYSKVNLSLTKGNLLETAIRFKNHTSDSTKITDPIEIHFITKTIENYLNFLVEEKELGAMLIDIVSSIMNLPKDMLKFAETSYNACTRLIKAVELITEFTPSIQLHKNNMALEEFRVKRENFGGLTCTWYSETSGDKEVKLLHCATNNKTSILSTRDKAIEASIQLPPSLLRRLDLTVAHQLMISMYTDNSLFPVTSGFSPKIEVTSGVIGAKLIGLQVANLTEPVYVMLKVPEFLSRRPKPVIWDTVGNSSEWSTTGCQLVNLINDLVIFHCDRLGYYGLLEDTSHLEAVMAPVGEKFRYSNPAIYIGTFIIISCLTITSVTYIICHASIVMPKRAKHCVVNTWVSITLLCFLYTAGIQQTDNLEICQSVGLVQHYLSLCSLLWMAVTASTMYKRLAKPDITQVPDDEIPEQPIQKPLLGLYLVGWGIALIVCGISGAINLREYASYSFCFLSSGAALAALFVPAVILIFYLTIFYLLVRCAIRSGDHNGQLSEGTQATENMDLELLEPNDNRADQNSVHSTQTVSSEVEDVEHSQITQLKGHIVVLVLYLIMWCAAAAATSRPFNPHLPHEETIFAVLYAISASSLGFFVLLFYGIARSDVRSQWTIMRCWLRRKKNRCCRTRSVTDANPSLPAQPLVQNLTMPVQGTPQIVSDTNSLSSSRITSASRACNALKISDGGSDTPSITKKGPNMNLVVLHRQQYRSNNSVTTFTEATHSVEMFYNPHQSGVARKFFKKQRRHTKHNNLGPRKQGDGGATSDGGSCVSIPRPAKIQDSEIERSIFGSSAKVNNTNIHVELNPVTATKNPNILSDSGGSISEDRTMPLRYVIGQEHTRISRKINNDDIRMIHNHPPERIRINPNAIECIRIDPNLASESVRMSQNVADCIRMSPNESDLETRTEEEKHLRNVSQQCSLEYSSEMDSGGQMLSERSDHDLPEIDETPETPDKITEGDFKCSSLHELTQMVDDKALARSEHSSSLYCLSEDRFEEPPLRTSYRSSYNDVTSLGTSSRNCEDFESEGKPTTDDNSRCSSFSNVNRMIPSDEELELLNELTLPNLNERAIEPSDYEKEYNSMTDLTAIDITLGPTRHLDMNASIGVDYEDANYENSQHFSEDGVPLDDAIIDANHGKKETSV

>Hheb081860.1 Orphan

MKAQLPLGLSEDIGYVLYSALGSFYIPSCIMVFVYIRIYFAAKKRANRNIRKAPRPRPAIAIPPESPDIRQTSFTQLTPATDSRLSSGINTMDNVATIESPQIQIPIVTCDYASDVSTSEADPGANYNNMEEKDTLKSLVNLQVNAPMPMQKLNLKATLSVNGNEGQSTSPKLPARCRAPSVGIDVDMVSEFDPSSSDSGVVSKCAVVKPLKLRICRPIFGKKAINKVKKEHSSDGKHINRDGVVVDNSTPRPRDPEREKKRIARKKEKRATLILGLIMGSFIACWLPFFFLYILKPLYANLNTPGAAFAVAFWLGYMNSAFNPVIYTVFNKDFRRAFRRLLYK

>Hheb006770.1 AKH

MNATRELPIDMRFNDGHVVSIIMYPILMVISIIGNVTVLYLILQRRRSNRSRINTMLLHLAVADLLVTVLVMPLEIGWAITVDWRAGDAMCRIMSFFRLFGIFLSGFILICISIDRYYAVLKPLQLMDVDRRGKIMLVSAWVGAFICSAPQVVVFQQKSHPEFTWYNQCISLGSFPSYAHELTYFIFGMTMMYWLPLSVIIFTYSSILLEIYRKSKEAGDKIRRSSVGFLGRAKIRTLKMTITIVVVFFVCWTPYNIMSVWYFLDRESAREVDQRVQKFLFLFACTNSCMNPIVYGVFNIRRDTRGSTSQQAVTIGKKTSIENVVCRVSWRRQETGSIRDNNDQNGQDTDVCLDIANQLSYNIGKNHENLTKSFGEAQYDDLFAIAVKHPKEKKLNHHSGKQTLIATKYGSSDTKFLFMFDSTSRRDCQFGGEIPLNTTFREAMENGLTESAFQVFSDKKYHPRVYKNSRIQEGWKHANCMSNSLLDVDYLRDLNVTNMRVRRRGDQPSIHKLDELNCIGEQKLVSTLNTLIEYTNQILEELRRNTQETRYVRKLLEECQGCKAPLPPLPPLRPSCDYNAPQCYPGGQCRDTASGPICVCPPGYRGNGIQCERVSACATAKCYPGGQCRETERGPVCSCPPGHIGNGITCERIRTCDDRPCFPGVRCENTPRGYRCGPCPPGHEGNGETCQTIRITCEMSPCGRGVTCHPIHEPPHHRCGGCEPGWKQHGSECRDIDECDLENPCRPNEECKNTLGSYRCIPCAPGYRGSRTGCVDIDECSTNNGGCVPNSECINTPGSYRCGQCIQGFTGNQTSGCYQVGNLCPDRVTVCHERATCNCIVPNVEYTCQCQVGWAGDGFACGIDSDNDRHPDEELNTDGDIIPADNCPHIPNSGQEDVDGDGVGDACDDDADNDGVLNSSDNCPYDANVAQEDTDRDRKDGVGDVCDNCPSIYNPHQEDTDDDGIGDACDRDIDNDGIINENDNCRFVKNEDQYDSDGDGVGDVCDNCRSVPNSNQSDSDRDGVGDACDTGRDRDRDGIQDDVDNCPDVPNADQLDTDNDNIGNACDDDIDGDGVPNLIDNCPYVYNPRQEKSHPGISGDACWNDFDNDTIANPYDNCPNNSQIWSTDFRQYERIPLDPVGDAQLDPKWYTHDDGAEIEQTVNSDPGIAIGFDHFGGVNYEGTMFVNTDIDDDYIGFVFAYQNTHKFYAVMWKKNSQTYWRSEPFRAVALPGIQIKLVDSETGPGKEMRNSLWHTEDTPKQVKLLWRDPKNVGWKEKTSYRWELLHRPKIGLIRLWIHQGDKVVVDSGNIFNSALKGGRLGVLCFSQEMIRWSNLQYSCRETVPQIVYDELPADLQAKVGVDSPKY

>Hheb008350.1 ETH

MKMLTTMSSTAFELNSSYYTTAIGSSSTIDGFSVSVLPPATNATSIPYVLPAYIRITSMVVVIIVMVLGIVGNLMVPLVVLRGKDMRNSTNIFLVNLSAADLCVLLVCAPTVLVEVNSGPQVWPLGEHMCK

>Hheb008990.1 Orphan

MHSRMGNAILLSHRPSRDLNNLNGELNSGGSSKTLTLNEVNQDHHLHTPTKDKNLMKMKREHKAARTLGIIMGTFILCWLPFFLWYVSTSLCGAHCHCPEIVVHIVFWIGYTNSALNPLIYAYFNRDFREAFKNTLQCAFCSLCRREPFDLEALDIRRPSLR

>Hheb055420.1 Orphan

MEEPSTEALMQAGFIFVVSIAIILSNLLIIATYLNFRGPSEVINCYLLSLATADLLCGLLVVPLSVYPALMKRWVYGDIVCRLVGYLEVTLWAVSVYTFMWMSVDRYLAVRKPLRYETVQTKTRCQCWMAFTWISVAMMCCPPLLGFNKPIFDEQAFICMLDWGNMAAYTITLSILILGPSVITIVYTYFYIFSMKLKLRSGVPIHDKEYATALSENLSNPSHYMSFALIMTFWLSWAPYALLRIYISIQGAAEIPLLHFAVVWLGITNSFWKAFILGTMSPQFRLAARVLCLTLCCRHRRLPPELLGLDDDD

>Hheb074400.1

MSSDYLNVTNKIYPGTTKAELNLPYAVCEIVVAVCAVLGNGLVIIVFSREKKLRRRTNYYIISLASADLMVGLFAIPFAILASIGLPTNFYACLFTVSVLVVLCTISIFCLVAVSIDRYWAILYPMGYSRNVRTKTAIAIICVCWITGTLVGFLPLLGWNAGFKKTDEKCIFVEVMDYNYLVFLYFATIIFPAFLIAAFYAHIYRVVIQQQLMSRKPINIEGKRRKRKGNNNNNEPSSGTMLRLLGAAQKREVKATQNLSRIVIFFIICWFPLYTINCVQAFCPDCTVSEFVLNATIILSHLNSVGNPILYAYHLKDFRAALKNFILRILNPGRCRRDNNAMGMNEIQNKIISQRNLAKKSLNTAIKHTNSNLSEKGVTLALPEPNNIDSSPSSTPTINRSPTICIKYEFPEEDVGYVDESLSSNVKTSDNSENYNNSNNYDLIEDSRDFKDDSVLQLREHQITIEESQLKEIE

>Hheb080560.1 Serotonin

MMRDLNASACNELYEAVEWSGPGIVGTLVVLAIVDVMVILGNVLVILAVYHTSKLRNVTNMFIVSLAVADLLVGVAVLPFSATWEVFKVWIFGDIWCSVWLAVDVWMCTASILNLCAISLDRYLAVTRPVNYPQIMSPKRARLLVAAVWVLSFVICFPPLVGWKDQMSHPTKTDPVPEKNGPFNTTIILVPVKPCPWICELTNDAGYVVYSALGSFYIPMLVMMFFYWRIYNAAVSTTKAINQGFRTTKGSKMFGSRFDEQRLTLRIHRGRGSVHNGANNNSTSSPRSPESSRSASVRRDKIKISVSYPSTETLNTKCNTLERTPSKCSQISVHYTNGQTQNQLCTTSRNTHLKVGGINRVGSARRPSRRSSCESQVTGDEVSLRELATSSEDKPPRVMKMGKRNIKAQVKRFRMETKAAKTLGIIVGGFILCWLPFFTMYLVRAFCPNCIHSTVFSVLFWLGYCNSAINPCIYALFKATEAQVIQYVCHSRAYLSRTLIQIQALSIMSTHRVTLDDRRVKYGERLV

>Hheb033210.1 RYa

AVSVLVSAYTLVAISIDRYIAIMWPLKPRMSKKQAKLLILAVWLVALTVSSPIAFVSQLLQPNERYKKCNQFICQEYWPSAHQRYYYSIALLVLQYLVPIVVLMFTYTSIAIRVWGKRPPGEAENTRDLRMAKSKRKMIKMMMTVVIAFTVCWLPFNVLTLILDNNESINSWRGLPFAWTALHWLSMSHSCYNPVIYCWMNARFRSGFISALAGIPCFRKFWPERRTPPYNTSTAGGIALTGNVIYSVPKEQLKNPQQPLKKNQTKIQHCVDTKTL

>Hheb00794 CNMaR-2

MYTIQSYCAGSRLRRSLLQQEASLSSNDSYENGYDYEEEPLPCTMTDFTFLIALLFVWLNSELGWKVFNVAGWCEILVYVSAVCSSLSVWLIVAFTVERFIAVQYPLHRPQMCTISRAKTIICALVVLSLVCHSYAFITAGVVTVGDSDYCDLKVEYMDVMKIISTVDSIASLIVPIVLIVVMNTMIMRNLLKFSRRFKQTPMNSLITSNQCPSRERSDINLNQIPNKSSSQSGIMLATIVGKRGGSQQSFHSSRNSHSHHSSSGSGGPVIITTTQTANVTSPAPPSSSGARNISYQVPEVGGAKCISLYHHTKSIGTSSKSVVSTRNQQSITKMLLLISTVFILLNLPSYVIRLCIFFFTLAKRDSPDLLWCLQQFFMLLYYTNFSINFLLYAMCGMTFRRCLEQLVQKAFKGVTR

>Hheb09984 NTL

MTSCCLENESNITMPAYMAIMRPLRYHLSRRRTIGALVLIWLASVLLAIPGLLYSTTMTRRYSNGKTRIVCYIMFPDGDYLNSRIEYIYNLIFLGVTYLIPMTVMAVCYSLMGRELWGSKSIGEHTKHQKESMKSKRKVVKMFIIVVMIFAICWLPYQGFSIYLYHHSDISSSSYIQHVYLSFYWLAMANAMVNPLIYYWMNNRFRVYFQKIICSCCVIGRSNAGSCQMHELADFHRSDTARSNSGRLKFTTIRWRQSTAESHVHPYKIKSRSICEKIHTSRQDVAVI

>Hheb08603 CNMaR-1

MEERVITSNKTNQSDTEDWIRMGSTTDWVETTMIAIQMYYTPVLVCLGTLGNCLSVYVFFRTKMRRASSSWYLSALVVSDTGFLISLFFAWLDMVGIGIFNLSGYCQFFVYLTTLCSFLSVWFVVSFTVERFIAVQYPLRRQSMCTVARAKMILIGLTCVGLVLCSPVLWFSSPRPIDKKPNVTGCRLVEEWEAWASAFNVADTILTFVLPFTVIVILNGLIARTVYRLARVRRTLTTNGRNRRDQKCNGIGVSQTKVTKMLLIVSTVFLCFNLPAYVMRVRAFLEVHDLRSTIIAQQICNIFFETNFGINFVLYCASGQNFRKAVVRLIFRRPRRWHSGTPVSNHVSDFRRSSSTMGRQRTIVYEVPWTEAYEMRGLDSRVSRQLSQQSNQGKL

>Hheb03416 SIFa

MVGGLYTAKTLNSFVKSYYESILKITGAQRVHTWVLGRFMCKTVSYIQGVSVAASVYSLVAVSLDRLYLLPFWLSLLPPQVVSPVYPLTTDLATFTHPMLLLTHQSIMLPMLPMSMLSTSPLLQSYTLHQLFLAIWWPLKCQITKRRARLLIVIIWCVALGITVPWLLFFDIKIIPSSVDQAFCVESWPRKEGERLFFLIGNLMFCYVLPMILISLCYIAIWVKVSRRNIPTDTKDAQMERIQQKSKVKVVKMLVVVVILFVLSWLPLYVIFARIKLGSSPTIWEEDVLNIATPIAQWLGSSNSCINPILYAFFNKKYRRGFMAILKSGRCCGKLRYYETVAIMSSSTSMRKSSYYVNNNNSSTRRTCHGPPVHQDSNVSYIFNHTGV

>Hheb05423 TKR

MLLTYFLPIGSMTFTYARVGLELWGSQSIGEATQRQLDNIRSKRRVVKMMIVVVVIFAVCWLPFHVYFIVTSYRPEITNQPYIQDVFLAIYWLAMSNSMYNPIIYCWMNSRFRRGFAQFFSWCPFVKVTPEPGLSRSEAVTSRYSCTGSPDGHTRIARNGTSTCSSSDQSLSTIASGTTRRDTILHERVDIDLCEFTSP

>Hheb107890.1

MAWSDIDFNSPIIQQQMLEMGALRCRNGSAPEPGWCPETWDSILCWPPTPAGALALLPCPRYIAGFDLQANATRQCMPNGQWYENPETNYTWTDYSQCYNSELVTVLMDLPEVEERNKTLIKTYLPIVSKMSKIGYAVSFSTLLVALVILATLKRLKCSRNKLHMHLFVSFMLRAFMAMLKDILFVSDLGLASDVITKNGESFWVVDDVVHNNWQCKMFTSFWQYFILANYSWIFMEGLYLHNLVFFAMSADSNASIKGHVVSGWGLPVIFVIPWVVARALYDDTYCWTTNNYQSLFLIIRVPTITTIVMNFLLFINIVRILYVKLRNSVAEELERYKRLARSTLVLMPLFGVHYAIFLTISFSGLNEKVELVWLFGDQLFASFQGSFVAVLYCFLNGEVKTEVLRELRSKKWLGSRWGLGSGPRHSTRSNSTCSCNGKSGKLRKPRWKRPWFGLFHRRTIHRSSHSMASTQDVTTRGGSLTSSHGYLDSVVVEGNKTYNTDLHDRSVTQMSPLCSKFTDQSMLSFCSNYLPANFRLTR

>Hheb003130.1

MTNGDLERFLQDQQDLCDLHIAEQAGLNNGLCPPSFDGLLCWPATLANTTAQLACPPRVVLGYENPNNGTATKRCLASAVWYNNSEGIAWSNYTACTPPQGYYITAIENIEITHAEYAKANNSTLLMKWLPIIRNVSKVGYATSMISLIIALIIFSLLRKLKNHRNRLHMHLFVSFVMRAFMALLKDWLFVDGIGLAWDIVLVDGKSAFIKEKNIWVCKAITSAWQYFIVANYTWILMEGLYLHNLVFLAFCADTSAITLYVVFGWGLPAVIVGLWIIIRILMEDTLCWTTHTNPSLFLIIRIPIMVSLLFNFMLFLNIVRVLLVKMKTSDHLQRKKMRYGRWARSTMVLMPLFGAHYTIFMGLSYHEDHQIELIWLFCDQLFASFQGCFVALLYCLMNSEVLSEMRRAWRAQCARRGGVWLDLTQRNFGKNSERGHGNKGTNNVTSNVSML

>Hheb011390.1

MEVEGKDDEVWCRWAWDSILCWPPTKASTTARLSCPLENGIDTTKFAERRCSDDGRWEGKGGVINEQTDAPSGWTNYTPCFTPEMLLLLQKLYSGNEEDAKVKLNIAQRTRTLEFVGFSLSLAALLISLAIFCRFRTLRNTRTRIHKNLFVAMVIQVVIRLTLYIDQALDKTEVYNAQQGINNTPVLCEASYVLLEYARTAMFMWMFIEGFFLHNMVTVTVFHETSYYRMYRLVGWGFPAIMTITWATITAIYYHPSKCWWGYNLTGYFWILEGPRMAVVLLNFLFLMNIVRVLIVKLRQSRSSEMEQVRKAVKAAVVLLPLLGITNLISMAAAPLEVTIWFALWSYSTHFLTSFQGVFIATLYCFLNGEVRLALNKSVSLYMSVRTTERQARRQSTFSACQPQRIESENETAALGVNAINAPTIEHTSWLQVCWHGKNIQDQKQPADYRMKQIVTNQTVKIPEGLTVTVKSRRVTVTGPRGTLKRCFKHLAVDIHMVSPRLLKVEKWFGTKKELAAVRTVCSHISNMLKGVTKGYQYKMRAVYAHFPINCVTTENNSVIEIRNFLGEKFIRRVKMAPGVTVCNSAKQKDELILEGNSLEDVSRSAALIQQSTTVKNKDIRKFLDGLYVSEKTTVVQDDE

>Hheb104700.1

MALNASDQSEEVNYIHGTLNYINELNDSLLIKEFECLKAQHFEALEGRKTESTFCQVDWDRLLCWPTSPPGTLVKQPCFEQLHGIHYDSSQNASRWCWQNGTWDSYSNYSQCQELRMNVIESGIEITTTLYFIGYTISLSTLLVAVAIFAYFKELKCLRNNIHTHLMLTYIFADLMWIVTTIMQVSMQTGMPTCIIFFSLLHYFHLTNFFWMFVEGLYLYQLVVKTFTGDNIKLRSCLAIGWGAPMVIVIIWTALKSWAISADQSSQNVALYRHCPWMVHHNFDWIYQIPAIAVLAINVMFLFMIMWVLITKLRSANTVETQQYRKASKALLVLIPLLGVTYVLVLTGPESGQVADIFTYIRAILLSSQGLLVALFYCFLNSEVQTAVRHRLSRWSTARNLRSDRKFYSNCSPRSRTESI

>Hheb039610.1

TPGNLTTTVGSTVQEEELRRIILERKQQCMEMLALNTTLPAEPYCPGIFDGWSCWPNTPAGQKAYTKCPPFVTGFDTSFYAHKVCEADGSWFRHPVSNQIWSNYTTCVNVEDLNWQQGINVIYETGYTISLIALVLSLAILTYFRSLRCARITIHMNLFASFAVNNTLWLMWYGLVLPNTEVLVENGITCRFLHVILHYFLLTNYAWMLCEGFYLHTLLVSAFTSEHNLVKWLMAIGWPTPAIIVLIYAILRGTSDDPDDNSQCWIDEGNYINVLVYPVCVSTLLNLLFLFNIVRVLLTKLRAGPAIGSRPSRSMLQAFRATLLLVPLLGLHYLLTPLRPPKNHPWSQSYEIISAITASFQGLCVAILFCFCNGEVIAQFKRKWEGSALMRKRANSCTATTVS

>Hheb110870.1

MRGRRIVITAVTLITMVIGLAVMMYYFNQDKCPMGTFLCQNSTECKPQRYWCNGRIDCPEGDDESFTNCFDASGNWEWFFKKRPQPPVLICDPRDCPSPECSCQGCRANCKGFTEHLPNLSPNITSMTLFNASVKQWRAHNLERYAEIRSLYLASNKIETLEEEAFSKQAKLYWLVLSHNKIKQIKRGHFKGLSSLETLLADDNRIAKADFSDFAESTSLEMIDLANNSLTEMTLIFPNLPAMKELILDNNNIKSINRDFLAGMPQLRSLSIERNKLITIDSGVFRNLGELTELNLADNRIRVIEEHLFDPLVNLTQLMIGYNPIENLPVSSFNELTNLRSLGLEDVDMENFDTNAFVPFAQLEFVYFKKFHYCTTYAPNVRKCRPASDGVSSLSHLLGKPLLKVAVWSISSVTCLGNALVLWGRFTAKDENRVLSILIKNLAVSDMLMGVYLLIIGLVDVQFRDTYYQQASSWMSSWSCTLLGILAMISSEVSVLILSFMSVERFILIAQPLRGQQRALTPQAAAFSMTFIWICGIILAFIPVIHWRSSTRFYGLNGLCFPLHIDDPFLVGWEYSAFIFLGLNLVGLVTIGYVYAGMFTSIWRTRHNTPLFVGDSEFALRFFFIVLTDAACWAPIIALKIVAFWNYPVPPDLHAWVVIFVLPVNSAVNPLLYTFTTPKFRERLNEGWMGQLKNYVFDRRATQDSQASAGSSQDVVPNGFLSLAAIGKWAEFDRKPSTHKQN

>Hheb00749 LGR

MERIDSRAFTNLSKLRVLELDDNLLSEIPEAIETISTLEDLSISNNRIDRIPANAFRGNKNLMSLDLRGNPIKVIDEGALQNHRKLRKLIISNVRSLADFPNLNGTRSLEVLRLDRASLKTVPEDLCKQCPKLKSLDLKSNYLESIPNLTECSDLKVFNMITSLANKPFSGQRFLHDLLLSNNNIKTIPEEAFAGLVRLQVLNLENNLIEYIHPAAFEAIKQLEDLNLGNNVFPDLPTKGLGNVLHLKTFNNPALREFPPPHLFPRVQTMILSYAYHCCPFLTAEYNEEVTKSSLQESVVFPDENDLELDPWNSSLTDSWSSSENSTNKFGAELKNLWDSYEGEYTYPGNVPTYIEDYFEDQEGRVSAPSGSGFPAHVQCLPQPGPFLPCQDLFDWWTLRCGVWVVFLLAMLGNGTVVFVIVFSRSKLDVPRFLVCNLAAADFFMGIYLGFLAVVDASTLGEFRKYAIPWQMSLGCQIAGFLGVLSSELSVYTLAVITLERNYAITHAMHLNKRLSLKHATYIMIVGWSFAFSMAILPLFGVSDYRKFAICLPFETNGIASLTYVVFLMLINGIAFLILMGCYLKMYCAIRGSQAWNSNDSRIAKRMALLVFTDFLCWSPIAFFSLTATFGLQLVTLEQAKVFAVFILPLNSCCNPFLYAILTKQFKKDCVLICKAIEESRVTRGIGRCRHSSNFSNRPTAANTNSLVDRSSRENHQPQCACNARLLEVNECRKTWWNSRILWSCRREMRSRPYNRSDAYAYQIAEIQQKQNKRASSMSSSENYSSSRSDSWRQAHHCGIPLRLLDPTRRASSWLITRKTSQDSNLSSSRNDSSGSATTASTSTWRMSRSSASLEFNSRTTPRPARSKPRLIRQFAIQEPEPPGSPSRLAVRLLATIPSAAEMSEQQEEESNADKE

>Hheb03289 NPLP

MQSIENQQLEQIGQNSIALIGNDRIKNLTIGYLTAIKGGLKDRQGLAISGAISMALDEINNNPRLLPNVQLVMRWSDTRGETVEATKAMIDMICDGVAAFFGPEGSCYVEAIVAQSRNIPMISYVPTFARTEPPDTQVTKSVIALLLHYGWNKFTIITEMAWISVAKSLENQAARNNLTVNHYKTVEDRHTCCEERLPCCQVSVWFQLIQETKNMTRIYIFLGTAMSLIDMMNSMQNQRLFDNGEYMVIYVDMMTYSQKEAQKYLWKPEHFDNLKNCLEPKDFLKRARSLMVVASTPPTQSYEEFTKKVRNYTSNEPFNFTVPDLLMNMKFDKYVSIHAAYLYDSVMLYAMALDQLIRERPESTIDELASNGTLIIETIIKKHTYLSVSGQTIKLDKSGDSEGNFSVLALKKEFFQRNNFSCDFQMKPVGQFQQGETLVYRPSESMDWPGKNKPEAEPGCGFLNEHCPKDDTHMRGVVVAGLLAVTLFCAAVITMSIYRRWKIEQEIEGLLWKIDPNDIAGYPVNDKIMASPSRLSLASAMSMESRVGGQVFAQTGQYHGVVVRIKELKFSKKKDVSRDVMKEMRALREIRHGNLNSFIGACVEPMRILLITEYCAKGSLYDIIENEDIKLDIMFIASLVHDLIKGMLYIHESSVLVCHGNLKSSNCIVTSRWVLQVSDFGLHDMRHCAESDSIGEHQYYRNLFWKAPELLRNPHASIKGTQEGDIYSFAIILFEIIGRKGPWGGVNLEPKAIGEPDKECPEYIVSTITDCWSESPELRPDFKSIRTRLKKMKAGRHRNIMDQMMDMMEKYADNLEELVSERTRLLFEEKQKTEDLLHRMLPEPVAHCLTNGIGVEPEAFDICTIYFSDIVGFTAMSAESTPFQVVNFLNDLYTVFDRIIKGYDVYKVETIGDAYMVVSGLPIKNGNRHAGEIASMSLDLLNAVKHYPIAHRPKDTLKLRIGIHTGPVVAGVVGLTMPRYCLFGDTVNTASRMESNGEPLRIHISAQCKEALDKIGGYIVEERGLVQMKGKGEVKTYWLVGANEKAIQKREVDVTDLPPLFCRPRRSPKLNPDSRQASLLTGLAAGSRRTSCVPRPSPDDSASQCGNSSPAPARQMRLSKLERNQLHLVDSKTTLDNVTVCEDAEIRAATIKQVLDGVFPENQQEAQRVLSTIASSSTTSDHPSAIIRESKSLDPFPSELHRDSPDVQRKEPKRSFRSLENVDNYGSKGDLRILNNNHPNGDIILKDNFVQDEEVNAPLLGDHNEAEMGIIKKWRSLDQVLVNNVSGDMVPEKKSSARNSIRSWLVNLFNGNTIRSSNVSIRRTVITGYDLQGERESIV

>Hheb03286

MQLINLERACLLVSLLSGFVGAETFTLGYITGSKRRINDLEYERPGIRISGAINLAVEEVNSGELGKLGHKLDFIVAETYGEEDTSILVTADLWTKNISGYIGPQETCVHEGKMAAAFNLPMISYFCTHHETSNKAEYPTFARTRPPDTQISKSVVAVLLAFNWTKVTFMYMNSTVTEFNSNWASVASTILELFQSSGITVTHERCWDEPYHTINVSNPFYRLVETSYKETRIYVILGNYDEHRGLLMALDEKKLLDNGEYWVVGVDIEQYKAKYPDEYLRGLLQDRTGPSLLRAYRSYFSIVASAPNISKKFTKIINEYRTKPPFNFKNPLQRFGGIVEVVPETAYLYDAVHLYARSLLSALKEGRDPRDGRKMVEMLHGVHYRSAMGYMVYMDRNGDAEGNYTLIALENHPDKGHGLYPIGHFVGKEESSNLPKLHLTRNITWLAGGPPVAEPACAHTGEIVGGIAGGILLILLAIVLVLYRNWRYEQELDSLLWKPVVRTSQVSLSSNPDADFRYSMIYTQIGFYRGRMFAIKKIRKKSIEITREMKKELKVMRDLRHDNLNAFIGACTEPPNICIVVEYCPRGSLKDIIENEDMKLDNMFMASLVGDIIRGMMYLHESVIRYHGNLNTSNCLVDARWVVKIADFGLREFKRDAECDSQDILKKYQSLLYRAPELLRSRQLPPSIRDFQKADTYSFSIVLYELHGRQGPFGPTHLSPADILAHLCNPTPSTPPLRPALDDLENCFDFVRDCLEECWSEDAELRPDFKTIRNKLRPLRKGMKQNIFDNMMAMMEKYANNLEALVDERTDQLTEEKKKTDALLYEMLPKYVAEQLKKGHRVEAEGFDCVTIYFSDIVGFTHMSAESTPLEVVNFLNDLYTCFDATIENYDVYKVETIGDAYMVVSGLPIRNGIQHAAEIASMSLCLLDKIKEFTIRHRPCEKLQLRIGIHSGPVCAGVVGLKMPRYCLFGDTVNTASRMESTGLPLKIHCSQETKELLDKIGGFYLEERGIVNMKGKGERLTYWLYGEHRGIRDARYQQIEQSLVPKSSLKNKTIRMSFLRCCSESPKRLRFASSDQLDGGNIENDEWSPCKGCMEGSKSASSSCPCVEKIEFGGSCFKDACKSVPASPNFLGKGKFCEILEGEADPLI

>Hheb087320.1

MARSGLHQDWWWLRMTIFICLLTGQYALPQRPIRTGLPIPMPPIDRDQSDVVIFLRNNCNTDKTSTISDEANEIGQFLSKYNEDGQLSVNILEKYLACGTKSWGLQVLIDVLGQASTKALVAALDVNICEVAEKLAHLWNKPLLTWTCPARMEDTNERTSTIRLSPSLPAVAQALGEIFLHFKWKSVSIISLDEEPWLSLDRAVIGVLRSVDIIPRHHVLVSRNARSEQIHQSLSPLESVIPRGIVLCLPIEDEKLMSRVMGELKAIRTSRTDNSLFLLVDPEGPGFFLPSPLTGEEEKSVESLADNSTGRPASMTSRWWHTQRQNFDQIPKRLGGRTTMNLLAFAPFDKRYDLLKIYNGTQEYSEPRMLDHLNESLTILTRDNSTLNTNNNKMFTYGLLDWRSSGSGANDGHWQPIAEVIESRDRLDVRELIENDLIDEETLDLALCLSGNDCGGHNNNVEGDEKETEEREDHQLPLKTSHIIVIILICFLLIVVLFIITLLVRRHIMTKRVAKGPFKIILTASDFVFPQPADNRRVDEGIETMLCCWLQQLQEFGGPEVEKPDLLQGSVGSLKPHLKASTGSLAWHTILKDPRARYNGDLVQLKELPCQSNFELKSKAMDVLVMIHGLRHENLNPFIGCLTEPARPCLVSEYCARGSLEDVLVQDEIKLDWSFRLSLLTDLVRGMKYLHSSPIRVHGYLTSRNCVIDARWVLKVADYGLPAFYEAQNIVPPPKSARDLLWTAPELLRHTGLRRKGTQPGDVYSFGIIMQEVVVRGEPFCMLALSPEDIIEKVKKPPPLIRPSVSKGAAPPEAINIMRQCWAEAADMRPDFNAVHDLFKKLNHGRKVNFVDTMFQMLEKYSNNLEELIRERTEQLDMEKKKTEQLLNRMLPSSVAEKLKLGMPVDPEEFAEVTIYFSDIVGFTTISAHSTPFQVVDLLNDLYTCFDDTINAYNVYKVETIGDAYMVVGGCPVRIQDHPSQIATMALDLLHQSGKFKVRHLPRTQLRLRIGLHTGPCCAGVVGLTMPRYCLFGDTVNTASRMESTGAPWRIHLSQATRDRLCQVGGYHIEYRGCTDVKGKGKMPTYWLLGKQGFDKQLPTPPPLGDDHGLEESLEGFKLEGIDDPKSECSEDSPTTTTTTEAHVLHKQATDERTTSSLKDEDDNSSGQSVCSFTTTCTSSKFSNAPTSLTRKVAVSVHDEREPTAAPSNLSAATGPLLGPAASTTSLSSISSSAFRPATSAPSRHRRVGHIDEDDLSTPYNHYRCLSPNEHHTKSSSRLLKRQFSLDRADEPVSIISESSMSLISTRPPPRLYKQNSAGAANDLEKIEEVPSLPPPHQTYRHAASMSLSVESLTLH

>Hheb037010.1

MRREMCQSIDIRNRVDQFSKLRGCRVVEGFVQILLIDHANETAYINQSFPELVEITGYLVLYRVSGLKSIGRLFPNLRVIRGHSLFINYALVAFEMMHLQEIGLHSLTDILRGSGNKPQNGCPVCDKKCPMRSMTHLKRYEERLCWNQEHCQLVCGTSSCLAKNTSSCCHESCLGSCEGTSAGHCLVCKDVSVDNMCVNKCPDNTYEFMNRRCVQEGECRRMRKPREALNTVKEYPYKPFNGSCIMECPAGYMEEEIDGKASCKKCKGICLKECSGINVESIATAQKLRGCTHIRGSLEIQIRGGKNIVKELEDNLNMIEVIDGYLKIVPVLDNQNLQELWDWSTHPKITIGAEGTEPKLFFHFNPKLCLQKIEELRIKAGLQPFTDLEVAPNSNGDKVACNVSKLHVRIHKRTAEAVLVGWEPFEHHDSRSLLGYVVYSIEAPEQNIAMYDGRDACGGDRWHVDDVASNEQKNDGNETRLQNHILTLLKPYTQYAFYVKTYTIATERSGAQSDLMYVTTMPGTPTPPRALTMWSNGSSELVIQWQPPMRANGELTHYNVYGRMEPDDAEFLQQRNYCNEPIHLPEKKSIATMAAEERERAEVERQLAKQPENPTCQCSGDKNFNEEDDREKEVSSSIAFEDALHNQVYIKRTINHRRKRHIDLSIPTDFETLYQQIFGDADKNEQCNDDDTCLAFKNKRETLDSQDNIEKELTDETIGNNTYMSFVKRVMVQNNSKDELPSITITGLRHFAAYNIEVQACRQRADGGKDLDLNPECSTKTMRTYHTLPLDGADDIPAGTFKLEMTGNSSQPVVQLQWAEPPEPNGIIVTYQIEYKRVDIQNIQAIPICITRTDFLRANKYYYLRGMTSGNYSIKVRATSLAGPGAYSEVKYFIIDASTTSSYVWIILGVFTSIILFSIVAFAFFCRRKIMGNVPNMRLIASVNPEYVSPTYVPDEWEVPREQIEVVRELGIGSFGMVYEGIIKDADKEDAEELRVAVKTVRKKASDHAKAEFLNEATVMKAFHTNHVVKLLGVVSCSQPTLVIMELMRNGDLKSYLRSHRPDNKDPKNQPTTEPPTLKRLMRMAVEIADGMAYLTSKKFVHRDLAARNVMIAEDMTVKIGDFGMTRDIYETDYYRKGTRGFLPVRWMAPESLKDGVFTNSSDVWSYGVVLWEMVTLASQPYPGLGNDMVLKYVISGGLMECPDNCPNSLYLLMRKTWHQKAHRRPTFIDITKILINNIHYEDFSQVSFYHSPGGIESRYLNVQHSTVKAKDLEISFDDLWEDYIEEEEQTENSPLRRDFGDFACIEPGRMPDCGDSHNGSEPCGESSKLVTNFHDLNSSEAPRKTVPIDSNGRSLNSSRDTLDCPFADSLRSSKNSPSIRHNNVSTTPHISPASASNSIPLTKRNNTSMGNFANNSSLSKKTHDYENHTPEGVATIENKDTVSIRITCPSVEDIEVDVINGDDRGEPSTVDHNNGTETLNGYIGNTAT

>Hheb003390.1

MGKNLMKTVVLGVLGIWWFLVICADGNSRLLRINNELATDKDTRKNESDRDLERRVERAIGDSSSNDGNLLMDSVPSQPGYTELTPIPETNSSEIKAEKKKIKGTKRFKDITVAPGGICRTVDVRNYLADLEILRPCRVIEGTLQVVLMENVDGNKTIEPFDQLREITGYLLFYRVANLKSIGQLFPNLEVIRGQQLLADNAFMVYEMATLQELALPNLVEISRGGVMIQKNPSLCYAETIDWDRIVRAGENSIRENNNNPSQCPVCTLNCPEGHCWSTRECQKMEKEECHPQCLGHCFGPTDRDCHVCRNFKLDGQCIPECPKHLYNYLERRCITAEECWAIEEPDINSLETVHLTPFAGMCKQTCPHGYEIGRDAKNMSTCVECKGKCQRSSLGVTIRRASEAQRLRGVVVIKGSLELQIRTGNSNLIMSELAASLGQLEEIMGYLKVTHSFPITSLSFLKNLRLIHGTKTDTNNASLIVLDNPNLSQLFPEDQEVKILSGKLFMHYNPKLCMSHIMRVVEKSGITNITNMEVEPESNGDKVACDIVDINITVSEKGPTYANLEWTAYKPATGQTLLTYLLNYVDTKYENITHDTNSCSGKQWQIIDVESDPESLDPVIKLKITDLKPYTKYAVYVKTMMTKDKNRAGSGTGQSRIIFFMTEPDTPDVPIDVTSFSVSESEIMVNWLPPERPNGPLGYYKIAGFLRPENPSVLHNRNYCDFPPELYVPDDVSEVTIKTPIVPKSCCDKDTSPSLSSHKFHIFCYDNMAISALPLNGRKQCDTQNHYGVNLMSPMSSIGKQSSLDYTHDVLDYQMTRMVNDTYYSFVFDVPSRNTSYLLKKLRHYSLYTIAVAACAEKRSNSGEMCSMFELTSAMTNKKDAADNIRKLEAQESNDTIVMLTWEPPIDPNGLTVAYTIEWVNLMIKDAKSTSECLPASLFHGYKIISNLSPGRYSARVRAISLAGEGPWSEGVTFTVGMDSSNISTIAMGITFFSIFGIVVFVFFLFRNHQKRKKQQRLIASVNPDYIESKYVKDSWEVPRENVMIIRQFGEGNFGTVFWGILNGEKPVAIKTPPRGSSEEGKNEFLNEASVMKKFSSHHIVRLVGVVSDGIPPYVIMELMENGDLKTYLRKLREAGQLSLDVPRIIRMASEIADGMAYLESKKFVHRDLAARNCMVSKDIVCKIGDFGMTRDIYETDYYKVGQKSMLPIRWMAPESLSDGVFTSDSDVWSYGIVLYEILSLAELPYQGLSNDEVMHHVMRKGTIDIDRDCPEVIQRVMEKCFKWRPFERPTFMEIISELEPFIGQDFCEKSFYHSEQGVEIRNSGAKKVYHQAAQIRFHWGNETARWIREFEDSAALLEPDKASTSRGKIFKNGFQQLGTEPIMEDVPLNR
