# Supplementary material for: Identification of Neuropeptides and Their Receptors in the Ectoparasitoid, Habrobracon hebetor
Source: Front Physiol. 2020 Oct 16;11:575655. doi: 10.3389/fphys.2020.575655 (PMC7596734; doi:10.3389/fphys.2020.575655)
Supplement: Supplementary file 6 [file Table_3.DOC]

>Hheb026340.1 NPF

MDETRRQLEALPDKYFEKIAHILMDLRNDTVDLSKPHLRISFANNYAFFIFLYVVMITMGVAMNIGMIYHIVRHKLYHDPTYAYLINLAISDVVKCIFVLPITLAVLMIHNWIFGKFLCFFLPMLQDIPLHVSMMTYLLIASDRYRLVSDPGKPRIPAFVVALGAWFFAVCIVLPYAIYTSYLDLTMYKKPTLHGFGICMVNLYDDIQQYMRCLFLFTYIAPLTITAYLYVKASRELQNQEEPMAVAMFEARRKNSYSRHGSNTSNDITSFRDGKRESGSVMTGGTVGLSGLSANYDLYDAELDVRKEQRTQKYLIFMVSVFAILLCPLMVLRLAKPALLETYENTGHFDITFIMFVWMAFASTVTTPLFYASWQMSRPAKERLKGYFQFSTKRLPPVLEKGLRHHGNRHQSGLANVAYTPQARNGSLSGSNGGEDYSRGNSTFHSPDLGNNVHRMNLVQ

>Hheb109890.1 CAPA

MKSKDLLSLNNTTTMEPVDTGGTEIFNFSFFFNSSEEEYLRLLLGPKHLPMRLVIPITVVYVFIFVMGIFGNVVTCWVILRNPVMQTATNYYLFSLAVSDLMLLILGLPFELRVFWQQYPWELGWGLCKIRAYVSETSSYVSVLTIVAFSMERYLAICHPLHLYAMSGLKRPLRFIFAAWLLAMIAALPFAAYTTVNYVEYPPGSGRNSEESAFCAMLLHNMPGFPLYELSCLIFFLVPLILIMVLYIRMGLRIQNTTLGGSIEGTVHGETRQAQSRKIIIRMLSAVVVTFFICWAPFHAQRLLYVYDRTSSFGDVNEWLYFLGGCLYYISTAINPILYNVMSVKYRSAFMETLCCTHGGNSLNRDDQSSMKETTIYRCGSCKSSQIARGRSKSVRYHSENFRDLLHTKPPDNTNDNFRPERHLMNNSNSLINNHETCLLESRKLSTVVHTSNGRAKCRTTDINGSPDETHI

>Hheb109910.1 CAPA

MDRMNLELLTSEEEREFEEFWDNLNNQNLTEEEYLTRVLGPKHLPAKLVIPLTLAYVTIFVSGVVGNVATCFVIIKNSTMHSATNYYLFSLAISDLILLCLGLPNELSSIWQQYPWPLGLGLCKIRAYVSEMSSYVSVLTIVAFSMERYLAICHPLRAYAINGPRRPIIIILAAWMIAIVSAIPFAIYMKINYVEYPPGSDKNSADSAICAMLLPDMPHFPLYELSCVVFFFIPMLIILVVYTRMGLKIRASTRNNAATRPGESSAHWDSRQVQSRKSIIRMLSEYLLMH

>Hheb023220.1 AstC

MNSTDFLDNPFNLTNTSCGVNVPIVAAVNQVLYSIVCIVGLLGNTLVIYVVLRFSNMKTVTNIYIVNLAIADECFLIGIPFLVTTISLGYWPFGQTTFTLYSFIFGFAIPLTLILIFYVLVLRKLRTVGPKNKSKERRRSHKKVTKLVLTVIAVYIACWLPYWVTQVTLIFTPPMQCQSMLTITIFLLAGCLSYSNSAMNPILYAFLSENFKKSFLKACTCAAGNDINAALQIENSVFPKKNKQRGERGQFNKMTTSVTSKPEMDDEEGERGLLICKSSTTNITMTSRSSIPMACDKDRNGGVENGIQETLLSSGGERGLIICKRPNTDIIITSKSGITIAGDKESEIKNDSQEQLMSSTDKQNFIICQSSTMTNKSGMPIEEREGGVENDNQEILLTEVURERERAYQS

>Hheb002560.1 Orphan

MTDSMSMAARIAEAANRTVIEGEMSRFPKPLRTFAAVVAILIMIVGLAGNLLTIVALCKYPKVRNVAAAFIIRFIDSGWTDVRFLCVLVPFLRYGNVGVSLLCVAAITVNRYIMITHHNLYGRVYKKHWIAAMILFCYVFSYGMQVPTLIGAWGKFDYDPNLETCSIIKDSNNRSSKTFLFVMGFIVPCIVIVGCYAKIFWVVHSSESRMRKHASPTVKSPHTPGRDTREIKQRRSEWRITKMVLAIFLSFVVCYLPITIVKVADPKVQYPAAHVMGYLLLYFASCVNPIIYVIMNKQYRQAYAGVISCSRIRATLTPHGSSVPGQNNYGQGNHIVTVTKL

>Hheb005840.1 ACP

MKPKLAASANDPLDPKNEARLKDRAVLTPIIKYVIPTFLIATSVLAGVYVTMLFLSFNENEPGITTTCDIQLAFRIDCLPGLEAIYGDCMDAGCCWDESDKFCYHTLPSVHTYQAVKHENYWALHTKNKLSPLKSYNKPSLQATINKIDNGYVEIKLTTTPSIQSMRHEIEKGAPAASPSNGNSTGHDSILDDLINVMIHEPTFGVTLTRVDNLTKAFPILTTSRGPLIITDHYWELSLYLGNSNTTLYGLNSGELNSSINWIYNNKGGRVMSNILGITSKSWIVGCYIDSQGPMEIEVLPSNLIIVRGLALPKDLSLHVFIGKEPEEITKRFVALHNNQLEREPPIPESFGLHICPDDTPKEMLSDLDKVIKTMDDYRAPWDTHCIYKKFRSTLDQKMSISDAEDLEDIRKKLEGIGRKIIHHISSMCSYGKSSLPFELANATMLLENTYGPYVGSIDSESFVYPEWRDERIEEVYNSVIEEYLHEAPVSKSLYVRDSWPRDDSNYTTAAFEKFDYLPKELRALMSSGTIPIDVHSNITSFHYKHHNEYAEKFDAFVGKYAYPMNVEEPNGESIGGWAALKNTLKRGIASGPIGQLPPAIYVCNVTTMDEGNLCTRWYGLAVAFPHILARPQNIPGGELLNPGTSKYVAQLLRLRTSFTLYQQSNIMAYFHNGASILSPTHYHYPEDTATRYTPDQFMWGPSVLVGLVTSPNIYQLQMAIPGEEPWRHILGGLIVHPSKVSISVLEGEIIALLRPGHIIPFHEETALTSMATSQRPLKLICNLACFDDKCNAQGKIFYHPNLYIQVNVSKTEIYLKTYNDGLVDCNIQENITIDTVRFLGIVISKSSSGTYVSNGASISGSRLRLRRSDMSSIERARSRTLKMTITIVAVFILCWTPYVAMLMWYTFDRQSAENVDPRLQDAFFIMAVGNSCANPLVYGSYAIDFRKECCRCFLPYPTAPKIDALELTQRNTGKKVQVTKIPSPGVSSLLVRSIRHAVPTYLRVGSLRTRSTGVSCNEVIPSSSPKLISESFSSKSLPLHEMKHEKSHNFLSVPGDDVLKMSTSSGIVSVGQSA

>Hheb011130.1 CCAP

MTRILDNLYSNKTDGNFTQLVGDFNLSDFIIRNELPNGSLADVNATDIDPFYFYETEQFTVLWLLFAVIVAGNVAVLAGLLLGKRRKSRMDFFIKQLALADLLVGLISVLTDIVWRSTVTWYAGNIACKLIRFSQVVVTYSSTYVLVALSIDRYDAITRPMNFSGSWWRARVLIAAAWGLSVLFSIPIIFLYEETVVEGKNQCWIELSSPVKWRVYMTLVSFTLFIAPTIIIGGCYTVIVATIWSQSSVLRQGPLRDRRASSRGLIPRAKIKTVKMTFVIVFARSENFPSIASLPSNDALHDKDFVTVLQDQIKVMKARTTALYHENVHLKTQIHQERMTSKNLEERLDQMKEYKNIGECKNEWTNTEMRPEEMTIKVPFTCHACSKTIVNDNDDDDDGDPMVFITKTELNNLEKDIKELRESLQLRENSWDGMVEREQNYSRQLTRLAQEIMTVNQLVENQSNDIENLSILLQAREGELKSAQKDIIGLQKLVVRLEKRNKALKENGGEKTMTEMNERDRKWIETIVRQVSTPRGRQKSKESYYSTPRSVKNSGRDETTTIIDT

>Hheb035150.1 TR

MRGTTNFFLANLAAADLCVGVFCVYQTLTNYLMNSWQLGDFLCKTYMFVHALSYTASIMILVVVCIERYLAIVHPIRCRSMLTRGRLRAAVVIVWILAAVYASPRFIYVETINHKLNSGSVDIICIANIRKHNKNVLDAVNLILLYLVPLFLMCCLYTRIALGLWKSGEAFGGPGLVARTRNGRVHHIHASSKNVLRARRGVIRMLIAVVLMFAVCNLPQQARILWLHVDPNYDRGSDFSTIFTVSTFLISYTNSCLNPLLYAFLSRNFRRAMRELFTCHNHNPSRAFGMGYVPGDAARLENGHTANMPHSSVIRLSSVHDSPCTTHTIARQGTLVKTSNENKMSINLRSVLISDPVDESCGALLASHGVPVTTKYKLSKEELIREIQHHDGLIVRSETKVTGEIIAAATNLRVVGRAGTGVDNIDLLAATRSGIVVLNTPGGNSISACELTCALISALARNVAQAAQSMKEGRWDRKLYSGFELSGKTLAVLGFGRIGREVALRMQSFGMKIVCFDPMLDPEVAASLGATKLTLDEIWPIADYITVHTPLIPQTRNLINATSLGKCKRGVRIINVARGGIVDELALLDALKSGQCGGAGLDVFAEEPPKNPTTLELIQHPKVIATPHLGASTAEAQQRVAVEIAEQFLAISGITDKYTVTGIVNAPILSAAMTVENGPWIELSKKLGQLAARFLKKNMNAPIESHTVGAGLQNKKFIHTAVLVGILSGQTKNGLNLINAATLAKDIGINVKEAHVDGEVDAVIIKIGNHQIKGTVRNNEALLLSVDDAMFNNGIVLRDFISLYHANGPQDLVTIVNAFSSKGITINSLNANGNWLVIETDQNVTIPIQGIEAF

>Hheb039010.1 LK

GAQPRMNTSWTAGNGESDLIWESDSNYSDVYNDSSIFEDSDELYNVPTGIIFLLSLLYGSISILAVAGNSLVMWIVATSRRMQSVTNFFIANLALADIVIGILAIPFQFQAALLQRWNLPYFMCAFCPFIQVLSVNVSVFTLTAIAVDRHRAILKPLSARPSKFCAKIIIACIWFLSGALAAPMAIALRVVLVPESSTGGRMHLKPFCQNVNLSEGSMITYRGLLGFFQYLTPLAIISCVYARMAFRLWGSQAPGNAQYSRDANLMRNKKKVIKMLVIVVTLFAVCWLPLQTYNVLQSTCHRINEYKYINIIWFCCDWLAMSNSCYNPFIYGFYNEKFKREFQQRYPFKSRKWSTSPPPGSLDIEKTMSTRTSLSVANIITTQNPNNTFKYSVNIAEGIERSSFNN

>Hheb111350.1 Crz

MELEHAPTLTQHAIIKAIVLCVLVADLFVSVFCIMGDAMWSYTVNWPWGNVACKFFKFSQMFSLYLSTFVLVLIGVDRFVAVRYPLTSFNYPGRCQQFVAIAWILAFILSIPQAKYRMQILGEQYY

>Hheb063160.1 RFa

MADEKYFDNFTNDTNTTYDPYADYDIEDSFNHFDWEELAPVVVIYSITFCLGLVGNLVIITSTLCPKLRPLPSTPTNIFLGGLASADLILILFCIPVKVAKLFSYSWTMGWFLCKGVHYMQSVSAICSVLTLTAMSVERYYAIVHPMRAQYTCTISQARRIVVITWISSFFLAIPIIFVQRHKPVGWRYPAFYCVRDDSQPTYWRAHELYMLLLVLVVPLVVMAFCYTAICWEIWLVMKRRYHMTSRHALNPSMNNNNVTNGECIPMTDRRRSTERSRRARSRREDTTTDGESRTMKQVVKMLVAVVVLFAICWSPMLIDNVITSYGILSQSKQGTVKHLNTAFQLMAYFNSCINPIIYGFMSKHFRESFLAAACGGWWCCFRRRVYTPPVKRHPSLSQTRTTSVSHFYLDNLLFSSQVRLIAPSFSENSKNFIYHCLTTIQIKGGLDAALPVNIDHPFPLSLIVSNSLQKIKEQQQQKISSPNLNN

>Hheb073560.1 PK

MRQGLREDRSAIDTFPIGINKDLGGDSLNEISQSIHENYTSQLAQLMTDDEFNQTTPKRDALYIVVPITIIYFAIFLSGLIDLLLLVSGLPPEMYYIWSHFPYIFGEIFCIIQSFAAETSANATVLTITAFTVERYVAICHPFRSRTIPQLSRVVKYIIVIWVLALCLAIPQAIQFGITFSKTLNGTIIPDTATCSVKWVIIKHAFEISTILFFVVPMTIITVLYGLIGIKLISSRMPGADKRKRPEQSNSQDSSRSGVPNEKNVLRMLVAVVVAFFICWAPFHAQRLLAVYAKSLGDGGSSLVTVYTTLTYISGIFYYLSTTINPLLYNIMSNRFREAFKRMLAEHCGGRRSIEPASPRKRTYSDLSHGRGPVGKRPEQNSGSFSASDETQHLTPLVRNDEIEQASSHQESSVLIAKDSIGRKQLSSDVTPKSRSNDSSDSSQIIIVTSLAKGLDEACHNKGGLSHSTVNKCLKVQRPIKAVTLGLLAERLRSGTKGLFAHQQRQTRSSKMRVDSTIQRVQQKMQSHPSIESANTISNSSLQDLDETEFTGSELAKYMGEINFELVT

>Hheb044860.1 AstC

MNSTDFLDNPFNLTNTSCGVNVPIVAAVNQVLYSIVCIVGLLGNTLVIYVVLRFSNMKTVTNIYIVNLAIADECFLIGIPFLVTTISLGYWPFADRYIAVCHPITSPKMRTQCISLLVSVTAWFTSALFMVPIVLYAETKDFPNGGNCNIFWPNNYGGQTTFTLYSFIFGFAIPLTLILIFYVLVLRKLRTVGPKNKSKERRRSHKKVTKLVLTVIAVYIACWLPYWVTQVTLIFTPPMQCQSMLTITIFLLAGCLSYSNSAMNPILYAFLSENFKKSFLKACTCAAGNDINAALQIENSVFPKKNKQRGERGQFNKMTTSVTSKPEMDDEEGERGLLICKSSTTNITMTSRSSIPMACDKDRNGGVENGIQETLLSSGGERGLIICKRPNTDIIITSKSGITIAGDKESEIKNDSQEQLMSSTDKQNFIICQSSTMTNKSGMPIEEREGGVENDKQEILLTEVURERERAYQS

>Hheb116660.1 AstA

MDEKISFNATRWIDWMDQIGQNSTNCTINESNCPDYDELLIKRVVKVVVPLFFSPIGILGLVGNSLVVIVIALNPGMRSTTNILIINLAVADLLFVIFCIPFTAADFVLPYWPFGDLWCRMVQYLIIVTACASVYTLVLMSLDRYLAVVHPVASMTVRTEHHAFLAICIVWFVILTASIPVLLIHGELTGAPEEEEGEKQKVVNAPKSPLLCSCDKGVGTWAIFDNDIADPFNVKILYSGLIQFVHEFGRGEKSAWNCTPNENDKKKSKYMAIMFLKYIIYLPDRSVESIDVNSINMLRLHLFLFTTLLAGFADDAHAASIFTHYNDEEHKGTDLCHSLVFCNSELLKTVQLANIFNDSKTFVDHYQLNDPSVTLANFERLMSETNRKPSKDQIAKFVAENFANTNEVLPWNPPDWQPNPPILERIEDPNIRDWVKQLNGIWKNLSRQMSPDVLKHPERHSFIPVEHGYIVPGGRFQELYYWDSYWGVEGLLLSGMTQTARGIILNLLSMVERFGFVPNGGRIYYLMRSQPPLLIPMIEKYVEATGDIEFLADNLLTMEKEFAYFQREKTVDIVKDGKTYRMARYIVSSQGPRPESYREDYKLAQFFPEAQRNTLYEDLKAGAESGWDFSSRWFVTDGDKVGNLSNTSTRSIIAVDLNAFLQRNARLLAEFNKLLGNRVKAREWMDVANAYQEAIDEVLWNEQFGIWLDYNIKNGQQRHHFYATNLTPLYTKSFNASRAAYYAKRTVEYLKSQGIDDFMGGTPSSLSETGEQWDAPNAWAPLQSIIIQGLYNTNAEPALSASKELATRWLRSNYLGFERYNQMFEKYDSGNPGHYGGGGEYIVQPGFEFTNGVVFEFLDINAKKLSSTIPIFSE

>Hheb090620.1 Opsines

MSNFLSGGGARKRELKVTKMVALMVTAFLIAWTPYAAVAIATQYFHWQPPNSLGVLPSILAKSSICYNPIIYAGLNSQFPQSLRKLLGMKQSRTRSQGPASDVTMGINREKVFE

>PPU05513-RA AstC

MMSNVSRGLFDFADNASQQNITMPLDCDANMPIISLLHQILYSIICIVGLLGNTLVIYVVLRFSKMQTVTNTYIVNLAIADECFLVGIPFLVTTMSLGVWTFGKFMCKAYMTSTSINQFTSSIFLFIMSADRYIAVCHPISSPKLRTPFISKMVSLTAWVTSAIFMIPVFLYANTMETKDGQMSCNIYWPDDHGGQTTFTLYSFVLGFAIPLTLIFIFYFLVIRKLQTVGPKNKSKEKKRSHRKVTRLVLTVITVYIICWFPYWLTQMALIYTEPNQCQSRITITTFLLAGFLSYSNSAMNPILYAFLSDNFKKSFLKACTCAAGKDVNATLHIENSVFPRRNKANAERIQSNKMVTSGNSKNDGEDEENERGLLISKTSTTTVTMTSRSNITVTSEVRDPTSQQQREKDALKNGTQLTLLTQV

>NV15867-RA AstC

MMSNISSGLFVFADNASQQNITMPLDCDANMPIISLLHQILYSIICIVGLLGNTLVIYVVLRFSKMQTVTNTYIVNLAIADECFLVGIPFLVTTMSLGVWTFGKFMCKAYMTSTSINQFTSSIFLFIMSADRYIAVCHPISSPKLRTPFISKMVSLTAWVTSAIFMIPVFLYANTMETKDGQMSCNIYWPDDHGGQTTFTLYSFVLGFAIPLTLIFIFYFLVIRKLQTVGPKNKSKEKKRSHRKVTRLVLTVITVYIICWFPYWLTQMALIYTEPNQCQSRITITTFLLAGFLSYSNSAMNPILYAFLSDNFKKSFLKACTCAAGKDVNATLHIENSVFPRRNKANAERIQSNKMLTSGNSKNDGEDEENERGLLISKTSTTTVTMTSRSNITVTSEVRDPTSQQQREKDALKNGTQLTLLTQV

>BmA1

MEIEEIELYRQMNYSYDFNGTFNGTMGTCPIVNLPYVSIVTQVLYALVCIVGLLGNTLVIYVVLRYSKMQTVTNMYIVNLAIADECFLIGIPFLITTMSLNKWPFGDYMCKTYMISTGINQFTSSIFLCIMSADRYIAVCHPIAAPRLRTPCVSRIVSAAAWTASAAIMTPIFMYAKLVRIGNKLSCNIVWPEQDFSQGQITFTLYSFALGFAAPLTLIFIFYCLVIRKLKTVGPKNKSKEKKRSHRKVTKLVLTVIAVYVLCWLPYWAFQMALIYSPPSQCVNHITITVFLVAACFSYSNSAMNPILYAFLSDNFKKSFLKACTCAAGKDVNATLHVENSVIPRRRARAQARAAEARGGFAAAVGGSRSEASTAMTSRSMAASEVLPLEARPPTLTPLIAHNGLSHSRL

>DmCG7285 AstC

MTLTSLITPTEQLAVAPNGTTLHQLESVESESYPSINGTQNETMVTSVRPHLDHRNRPTQQNGSHYLEYDDDGPDCSYSYNFILKLITMILYALVCIIGLFGNTLVIYVVMRFSKMQTVTNIYILNLAIADECFLIGIPFLLYTMQVGNWPFGNYMCKAYMVSTSITSFTSSIFLLIMSADRYIAVCHPISSPRYRTPFVSKLVSAFAWMTSVLLMLPVILFASTVQSSNGNVSCNIEWPDTQNSHTDSTFILYSLVLGFATPLTFILVFYCLVIRKLHTVGPKHKSKEKKRSHRKVTKLVLTVISAYIFCWLPHWISQVALISSAPQRCASRLELAVFLACGCLSYSNSAMNPILYAFLSDNFKKSFMKACTCAARKDVNAQLQLENSFFPKFGKGRQSERLLGGNGKGGAQRGALTKKKCLATRNNNAPMATTTTTTTTTTGTDAVTCLQPPVHQVPAEIQVGNPATVLVVNAETNNCKPPVLHTDL

>DmCG13702 AstC

MEGGWWRGGGGGGRLGGKAIMEGHSTPNGAAASHRNNSTRTNIATNGCAHSGILLFVLTAMTLTSLITPTEQLAVAPNGTTLHQLESVESESYPSINGTQNETMVTSVRPHLDHRNRPTQQNGSHYLEYDDDGPDCSYSYNFILKLITMILYALVCIIGLFGNTLVIYVVMRFSKMQTVTNIYILNLAIADECFLIGIPFLLYTMQVGNWPFGNYMCKAYMVSTSITSFTSSIFLLIMSADRYIAVCHPISSPRYRTPFVSKLVSAFAWMTSVLLMLPVILFASTVQSSNGNVSCNIEWPDTQNSHTDSTFILYSLVLGFATPLTFILVFYCLVIRKLHTVGPKHKSKEKKRSHRKVTKLVLTVISAYIFCWLPHWISQVALISSAPQRCASRLELAVFLACGCLSYSNSAMNPILYAFLSDNFKKSFMKACTCAARKDVNAQLQLENSFFPKFGKGRQSERLLGGNGKGGAQRGALTKKKCLATRNNNAPMATTTTTTTTTTGTDAVTCLQPPVHQVPAEIQVGNPATVLVVNAETNNCKPPVLHTDLXDRAPSMPLETVVFIARR

>PPU06379-RA AstA

MLLTANLTSTALQQQPPIGCYEDELMEEELDFDQVLVQSIVQVVVPIFFGLIGILGLLGNSLVVIVVAANPGMRSTTNILIINLAVADLLFVLFCIPFTATDFVLPYWPFGNAWCKVVQYLIIVTACASVYTLVLMSLDRYLAVVHPIASMSVRTESHAFIAIWIVWIVILTSSIPVLIIHGEWDENQMLNRSEACALHLGIVEQHRQQLQQHESKDEEPPLELTPQIACRILPDANWPLFQVSFFLASYVVPLTLICGLYVCMLLRLWKGARVSAESRRGRKRVTRLVLVVVGVFAVSWCPIQVILVIKSLDMYPLSSATIALQIASHVLAYTNSCVNPILYAFLSDNFRKAFRKIIYCRSRQDQHNRLGPLTKTTRAGSSGDIFCRTTTLVVDDESDDEVVPADNTSSQGQSARIIES

>NV10415-RA AstA

MTELGWSANSSVSQAILLTANLTSTALQQQPPIGCYEDELMEEELDFDQVLVQSIVQVVVPIFFGLIGILGLLGNSLVVIVVAANPGMRSTTNILIINLAVADLLFVLFCIPFTATDFVLPYWPFGNAWCKVVQYLIIVTACASVYTLVLMSLDRYLAVVHPIASMSVRTESHAFIAIWIVWIVILTSSIPVLIIHGEWDENQMLNRSEACALHLGIVEQHRQQLQQHESKNEEPPLELTPQIACRILPDANWPLFQVSFFLASYVVPLTLICGLYVCMLLRLWKGARVSAESRRGRKRVTRLVLVVVGVFAVSWCPIQVILVIKSLDMYPLSSATIALQIASHVLAYTNSCVNPILYAFLSDNFRKAFRKIIYCRSRQDQHNRLGPLTKTTRAGSSGDIFCRTTTLVVDESDEEVPSDNKSSQGQSARIIES

>BmBAR

MESTEDEFYTICLNLTAEDPSFGNCNYTTDFENGELLEKVVSRVVPIFFGFIGIVGLVGNALVVLVVAANPGMRSTTNLLIINLAVADLLFVIFCVPFTATDYVMPRWPFGDWWCKVVQYFIVVTAHASVYTLVLMSLDRFMAVVHPIASMSIRTEKNALLAIACIWVVILTTAIPVGICHGEREYSYFNRNHSSCVFLEERGYSKLGFQMSFFLSSYVIPLALISVLYMCMLTRLWKSAPGGRVSAESRRGRKKVTRMVVVVVVVFAVCWCPIQIILLVKALNKYHITYFTVTAQIVSHVLAYMNSCVNPVLYAFLSENFRVAFRKVMYCPPPYNDGFSGRPQATKTTRTGNGNSCHDIV

>DmCG2872 AstA

MAGHQSLALLLATLISSWPKASWGATGNGSIISVSNSSGNNYAFTSEHTDHSDHNANDSMEYDAESVALERIVSTIVPVFFGIIGFAGLLGNGLVILVVVANQQMRSTTNLLIINLAVSDILFVIFCVPFTATDYVLPEWPFGNVWCKFVQYMIVVTCHCSVYTLVLMSFDRFLAVVHPVTSMSLRTERNATLAIMCAWITIVTTAIPVALSHSVRIYQYHGNAGTACVFSTEEEIWSLVGFQVSFFLSSYVAPLTLICFLYMGMLARLWKSAPGCKPSAESRKGKRRVTRMVVVVVLAFAICWLPIHVILVLKALNLYGGSHLSVIIQIISHVVAYTNSCINPILYAFLSDNFRKAFRKVVWCGSPPPLMTNQQVTKTTRTATGNGTSNIEML

>DmCG10001 AstA

MENTTMLANISLNATRNEENITSFFTDEEWLAINGTLPWIVGFFFGVIAITGFFGNLLVILVVVFNNNMRSTTNLMIVNLAAADLMFVILCIPFTATDYMVYYWPYGRFWCRSVQYLIVVTAFASIYTLVLMSIDRFLAVVHPIRSRMMRTENITLIAIVTLWIVVLVVSVPVAFTHDVVVDYDAKKNITYGMCTFTTNDFLGPRTYQVTFFISSYLLPLMIISGLYMRMIMRLWRQGTGVRMSKESQRGRKRVTRLVVVVVIAFASLWLPVQLILLLKSLDVIETNTLTKLVIQVTAQTLAYSSSCINPLLYAFLSENFRKAFYKGLQSNRLGMWTTTHQDVSSEKTTY

>PPU10178-RA PK

MLNESFVASEYRRELDWLNLTATLLQEPLVDSNEDDDSIARFLERDPLYIVLPISILYTLIFITGLVGNVSTCVVIARNKCMHTATNYYLFSLAISDLLLLISGLPPEIYYIWSNIYIFGETFCIIQSFAAETSANATVLTITAFTVERYVAICHPFISHTMSKLSRAVKYVIVIWLLALCLAIPQAIQFGIVYSKLANGTLLKDSAMCSVKWPFIHHAFEISTILFFVVPMTLITALYILIGVKLRTSRLLSTVKRIPSGQGFGQSDSRSKNCSQRNVIRMLVAVVVAFFICWAPFHSQRLLAVYAENNKKDKHKLAIVMPVYTALTYISGIFYYLSTTINPLLYNIMSNKFREAFKAMLSKHCGSSLQKSNPGRPTYSSLSRYPRSTIHRVDPHQVSASLSVSEETQKLSPTAAECPVEIISCARLGYCSPADKNESNEIVTENILCREFLNNEPVQSKCIKPECWLIT

>PPU07065-RA PK

MEEPFGNSSNSSNSSYLGEEYLVDPLKATFGPVRDQLYVVIPISIIYASIFVTGTVGNISTCIVIARNKSMHTATNYYLFSLAVSDLLLLIFGLPSEIYQVWYKYPYVFGEAFCILRGLAAETSTNASVLTITAFTAERYVAICHPFLSQTMSKLSRAVKLILFIWLIALVCAVPQALQFGIVSYGTPDALMCQYKRQILQFSFELSTFLFFIIPMTLIMVLYILIGLKLKKSTLMKRNCRQHHRRSESTRVKVGSSRMDRHCRHSRSTRRVLKMLVAVVIAFFICWAPFHMQRLIAIYGKKNVYTLDRHYWMEQIYLILTYVSGVLYYVSTTINPILYNIMSNKFREAFMETLARSCRMSRFVMPRERRSYSSLSRSQQRNPATYPSRTTAVSGGTALAQDSTDCSGNSFREEHQDLRAGANIAEYSSSETPPPLPPLIYGNSKQSSRKSVITIEIGASDTSLSIMSVGNAPGQPRGSGSAGMGSHFVELNRQTGRNHSIGRELPSPGYDRCIRMTASPAQPLQQSTVVNCRNKPNQTTSSKKKWWRVLDWLPGLKSIRTSRALTRPTQDNIIAELPKQPDDYFMQLHTFKAKDESCRPV

>NV14978-RA PK

MLNESFVASEYRRELGWWNVTATSLQEPLVDRREDDDDDSIARLLRRDPLYIVLPISILYTLIFITGLVGNVSTCVVIARNKCMHTATNYYLFSLAISDLLLLISGLPPEIYYIWSNIYVFGETFCVVQSFAAETSANATVLTITAFTVERYVAICHPFISHTMSKLSRAVKYVIAIWLLALCLAIPQAIQFGIVYNKLPNGTLLKDSAMCSVKWRFIHHAFEISTILFFVVPMTLITALYILIGVKLRTSRLLSTVKRIPSGQGLGQSDSRSKSCSQRNVIRMLVAVVVAFFICWAPFHSQRLLAVYAENNKKDEDKLAIVKPVYTALTYISGIFYYLSTTINPLLYNIMSNKFREAFKAMLSKHCGSRLQKSNPGRPTYSSLSRYPRSTIHRVDPQVSASLSVSEETQKLSPTAAECPVEIISCSRLGYCGSPAGKNEPNEIVTENILCREFLNNEPVQSKCIKPECWLIT

>NV12532-RA PK

MEEPFGNSSNSSNFVGEEYLVDPLKATFGPVRDQLYVVIPISIIYASIFVTGTVGNISTCIVIARNKSMHTATNYYLFSLAVSDLLLLIFGLPSEIYQVWYKYPYVFGEAFCILRGLAAETSTNASVLTITAFTAERYVAICHPFLSQTMSKLSRAVKLILFIWLVALVCAVPQALQFGIVSYGTPEALMCQYKRQILQFSFELSTFLFFIIPMTLIMVLYILIGLKLKKSTLMKRNCRQHHRRSESTRVKVGSSRMDRHCRHSRSTRRVLKMLVAVVIAFFICWAPFHMQRLIAIYGKKNVYTLDRHYWMEQIYLILTYVSGVLYYVSTTINPILYNIMSNKFREAFMETLARSCRMSRFVMPRERRSYSSLSRSQQRNPATCPSRTTAVSGGTALAQDSTDCSGNSFREEHQDLRAGANIAEYSSSETPPPLPPLIYGSSKQSSRKSVITIEIGASDTSLSIMSVGNAPGQPRGSGNAGMGSHFVELNRQTGRNHSIGKELPSPGYDRCIRMTGSPARPLQQSSVVNCRSKPSQTANSKKKWWRLLDWLPGLKSIRTSRALTRPNQDNIIAELDQRLPKQPDDYFMQLHTFKAKDESCRPV

>BmA2

MMEPNNSEYENNSYITFFGPVGMAFYDNGQVLEDSLTVKIILSIILAVIMILSLIGNGCTCAVIARNRSMRTPTNCYLFNLAITDLFMALFVPIDIYIIWIPEFYPLGEVGCRLHFVLWDCLSNCSLLIITAFTVERYLVITRPFLRQKLSLNSRVFKLVGVIWFVSCSFCIPDLLYIDMIEEKKYVFCYVAMSHIVSVFVAAEIFVFYVIPMTIIIILYILITIELKFKKKLRSSPASNGQQNRDKAVIMLAAVALSFFLFWSPYCYLRIMLIWPGVYEKHYNAWKIVNYLCYNSYASSALNPILYSLMSRKFRRAFKDFFTRRKPDSTNRNDIAKASLSKNETKLELKM

>DmCG8784 PK2

MLQGVAITIANDSNDDGINQSFMAHVSPSPNQSPSIGVGIGIASSTMANPSESPEMLLLKNDKFLTHVAHLLNITTENLSNLLGSTNGTNASTMAADSPVDESLTLRTALTVCYALIFVAGVLGNLITCIVISRNNFMHTATNFYLFNLAVSDLILLVSGIPQELYNLWYPDMYPFTDAMCIMGSVLSEMAANATVLTITAFTVERYIAICHPFRQHTMSKLSRAIKFIFAIWLAAFLLALPQAMQFSVVYQNEGYSCTMENDFYAHVFAVSGFIFFGGPMTAICVLYVLIGVKLKRSRLLQSLPRRTFDANRGLNAQGRVIRMLVAVAVAFFLCWAPFHAQRLMAVYGLNLINIGISRDAFNDYFRILDYTSGVLYFLSTCINPLLYNIMSHKFREAFKITLTRQFGLARNHHHQQSQHHQHNYSALLRQNGSMRLQPASCSVNNNALEPYGSYRVVQFRCRDANHQLSLQDSIRTTTTTTTINSNSMAAGNGVGGGAGGGGGGRRLRKQELYGPGPGTAVPHRMLQAQVSQLSSLGDANSLLEAEVVDRHYASGRAKRALLATKSGALLVTPPQSGDPSEVSQPATRLKLTRVISRRDEVANTSTPPFCGSHSLPDPETCQSASVAGRSSRKFPWRKRRQKTEDPSSEGLTYGSPKSQ

>BmPBANR

MMADETVNMEMLENNLLNVTNVTDQSSAYSESYPLHLLVPLSVTYAVIFIVGILGNTSTCVVIARNRSMHTATNFYLFSLAISDIILLVCGLPLELYRLWNPFTYPLGEAQCITIGLASETSANATVLTITAFTMERYIAICRPFMSHTMSKLSRAVRFIIAIWVFALCTAVPQAMQFGIVSYVENGQSMSACTVKGPGVHQVFVISSFVFFVVPMSVISVLYALIGLKLRTSRILHPVKKLSLDSNERPGAHTPYRNGSSQRRVIRMLVAVALSFFICWAPFHVQRLLAIYGKSLEHPSDTFYLVYIVLTFLSGVLYFLSTAINPFLYNIMSNKFRNAFKMTLAAWCGRRGGPRMGRSYSALLASQRQRAANGLTDPVRGPRRLRRLSTATTHLCDAPPRAQVSATKIAISP

>DmCG8795 PK2

MAVKMLPTNSSGVLATDLQLFHNEKFLLNLTQVLNISADNLTSLLQGLEPEELLPTVTPMTPLSLLATLSVGYALIFIAGVLGNLITCIVISRNNFMHTATNFYLFNLAISDMILLCSGMPQDLYNLWHPDNYPFSDSICILESVLSETAANATVLTITAFTVERYIAICHPFRQHTMSKLSRAVKFIFAIWIAALLLALPQAIQFSVVMQGMGTSCTMKNDFFAHVFAVSGFLFFGGPMTAICVLYVLIGVKLKRSRLLQALPRRCYDVNRGISAQTRVIRMLVAVAVAFFICWAPFHAQRLMAVYGSTSGIESQWFNDVFSILDYTSGVLYFLSTCINPLLYNIMSHKFREAFKVTLARHFGLGGKNQGRGLPHTYSALRRNQTGSLRLHTTDSVRTTMTSMATTTTGLNGSANGSGNGTTTGQSVRLNRVSLDSVQMQGQNRSRQDLFDNPRRMLQTQISQLSSVGDAHSLLEEDLQFPGEPLQRQPTMCSIDELTDDLAISRSRLKLTRITRPPGGVTGGVAGGSTTGAAGSGGVSGDESSGKVRKAKVKVLKSSSPFKGLRTKFNWRARRKGSHKPHEKGATVNGGDTEERAAF

>BmDHR

MNSETINDTANASRPVDSTRVFGPQRDTLYIVLPITIIYTFIFVSGLLGNIFTCIVIVRNKNLHTATNYYLFSLAISDLLLLVSGMPQEMYSIWSKWPYVFGHTFCVIRGLAAETSTNASVLTITLFTIERYLAICHPFVSHKMSKLSRAVKHVVLLWVAALALALPQALQFGIRQYQGVIMCLQTRVIIEHSFEISTFLFFLAPMVLITVLYSFIGLKLREKSNVKEQNQNDFESSIRYSHKMCRKPSQSTRRVIKMLVAVVVAFFICWAPFHAQRLVAIYGTNENHLAKSPILFSVYLFLTYISGIFYYMSTCINPILYHIMSNKFRDAFKMTLCCCGTRNDTAVKRSSYTAMAFVRHPTSSGTSNSGNSIRNETNLQSKTRRTNGRDKILNDAHVCRNGTLSSAAVGKPDSRTNGDRPLDRNLINETYFNTNC

>DmCG9918 PK1

MSAGNMSHDLGPPRDPLAIVIPVTVVYSLIFITGVVGNISTCIVIKKNRSMHTATNYYLFSLAISDFLLLLSGVPQEVSYIWSKYPYVFGEYICIGRGLLAETSANATVLTITAFTVERYIAICHPFLGQAMSKLSRAIRIIVLVWIMAIVTAIPQAAQFGIEHYSGVEQCGIVRVIVKHSFQLSTFIFFLAPMSIILVLYLLIGVHLYRSTLVEGPASVARRQQLKSVPSDTILYRYGGSGTAMSFNGGGSGAGTAGLMGGSGAQLSSVRGRLNHYGTRRVLRMLVAVVVCFFLCWAPFHAQRLIAIYAPARGAKLRDQHEFVYTVMTYVSGVLYYLSTCINPLLYNIMSHKFREAFKAVLFGKKVSKGSLNSRNNIESRRLRRALTNSSQTQRFSIESAEQPKPSIMQNPTNKPPVAAQYAMIGVQVN

>BmA25

MDTGSFLRGNETYDEFFERCNNLSRFDCTEEEMLWWLMGSRRLPLREIIPISIVLVVIFLTGVIGNVCVCVVIVKHPGLHTATNYYLFSLAISDLLLLMFGLPNDLSVYWHQYPYSLGLVFCKLRALISEAATYVSVLTISAFSLERYLAICHPLHVYAMAGLTRASRIILILWIISIVCASPFAVYTDITYRDYPPNSGNISVDSAFCALMASSPLLELSSIFFFFIPAVLILCLYVRMGLHIRSTRLTEKTKLGLLNGHVHGETRQAKSRKAIIRMLAAVVIAFFVCWAPFHVQRVFYVYGYSLPHYHVINEHLFNVAGALYYVSATVNPILYNVMSGR

>BmA27

MVEFTRENETVSELISRCTNISHFDCTEDAMLWVMMGPRRLPLQKIVPISVLLLVIFVTGVVGNLTVCVVIVRHPTMHTATNYYLFSLALSDLLLLLFGLPNDLSVSWHQYPYSLGIVFCKLRALISEAASYGSVLTIVAFSLERYLAICHPLHLYAMAGLRRALRVVAALWLLSFVAAAPFASYTTVSYHDYPPGSGNSSLESAFCAMLEVPSWYLYELSSLLFFILPGLIILCLYVRMGLRIRSTHTSKPGSPGTLNGVNGSVHGEARQAQSKKTIIRMLAAVVIAFFVCWAPFHFQRLFYIYGTGASHYHIINEYLFYVAGAFYYVSATVNPILYNVMSHRYRIAFKETLFCKKATRIRSKYIEQSSTRETVVHNGRRTRSKYRNERKNCSYYVTETSLCSEWKKDFYQQKKMHVLYKERSGSELCSENEASQLMFGYLPGEENDDT

>DmCG14575 CAPA

MNSSTDPTFSELNASFTNTPDTLFATSVSSDPSHGFGEEDYACGTFNCSPKEFVAFVLGPQTLPLYKAVLITIIFGGIFITGVVGNLLVCIVIIRHSAMHTATNYYLFSLAVSDLLYLLFGLPTEVFLYWHQYPDLFGMPFCKIRAFISEACTYVSVFTIVAFSMERFLAICHPLHLYAMVGFKRAIRIITALWIVSFISAIPFGLLSDIQYLNYPLDHSRIEESAFCSMSPKIVNEIPVFEVSFCIFFVIPMILIILLYGRMGAKIRSRTNQKLGVQQGTNNRETRNSQMRKKTVIRMLAAVVITFFVCWFPFHLQRLIFLYAKNMDNYLDINEALFSIAGFAYYVSCTVNPIVYSVMSRRYRVAFRELLCGKAVGAYYNSGFARDHSSFRESSAYDRVHSVHVRASQHPNKFETDSSSANRVLIKKTYSLPLPKNADSTVLSTTDIVIVLENSHTVCEEPKVENDIWIENEETCI

>PPU09239-RA AT

MESLVVTALTFLATSASGETGEAEDDSANDALDPLSNCTNNLCISEDEYLDEMHAYIYPKSYEWVLIVLHCIVFIVGLVGNALVCLAVYRNHTMRTVTNYFIVNLAVADLLVIIICLPPTILWDITETWFLGLMPCKIVLYLQTVSVTVSVLTLTFISIDRWYAICFPLRFKSTTSRAKTAIIIIWVMALLFDIPDLLVFYTHQNRKLHGKTILFTQCLPSWSRENQIAFNIIKLILLYTGPLMFMSFAYCQIVRVLWRNDIPGHNLSTRIINANDLSSQSNVGNPEGQLKSRRKAAKMLVAVVLMFAVCCFPVHLLNILRSSIEIRSSDLVNITSCLVHWLYYANSAINPLIYNFMSGKFRREFKRTFCCPRGGGSHNRAVYRMAARKSSHSAPSARGLSSRVIIIRSSDKAI

>NV13423-RA AT

MESLVVTALTFLATSASGETSEAEDDSANDSLDPASNCTNNLCISEDEYLDEMHAYIYPKSYEWVLIVLHCIVFIVGLVGNALVCLAVYRNHTMRTVTNYFIVNLAVADLLVIIICLPPTILWDITETWFLGLMPCKIVLYLQTVSVSVSVLTLTFISIDRWYAICFPLRFKSTTSRAKTAIIIIWVMALLFDIPDLLVFYTHQDRKLHGKTILFTQCLPSWSRENQIAFNIIKLILLYTGPLMFMSFAYCQIVRVLWRNDIPGHNLSTRIINANDLSSQSNVGNPEGQLKSRRKAAKMLVAVVLMFAVCCFPVHLLNILRSSIVIRSSDLVNITSCLVHWLYYANSAINPLIYNFMSGKFRREFKRTFCCPRGGGSHNRAVYRMAARKSSHSAPSARGLSSRVIIIRSSDKAI

>BmA5

MALRKESLAIITMLIICNYVLSSNFDSIPESIRVRKSVDNTTSRSSLKNLNETMKQSNNETEFGRLLDATEMTTEYDNFTEEPCVGDRAFCNLTREEYMEMLNDYVFPQPYEWVLIATHAIVFVIGLIGNALVCIAVYRNHSMRTVTNYFIVNLAVADFMVILICLPPTVLWDVTETWFFGTAMCRIVLYFQSVSVTVSVLTLTFISVDRWYAICFPLKFKSTTGRAKTAILIIWLLSLLFNIPEFVVLQVQTKMQLRFNVQYFMQCASTWSDESDLTWHIIKALFLYTFPLLLMTIAYCQIVRVLWRSDNIPGHTESHKLCSTQTGQSNWLAASRRTTPSIHTNASTEGQLRSRRKAAKMLVAVVAMFAVCYFPVHLLSVLRVAFDVQQTDVMTCIALISHVMCYANSAVNPLIYNFMSGKFRREFHRSYFKCFCCCHTTPAPEQNGASFEPIGSSRARTIRTTVRRHDSCVSYRLAHLSPSNHNIHRDYIQNTNTSFIEPMNGNRRSKIRDESISDTATRFTVTTDIPCKD

>BmA16

MTTVEDDLNVPKKMKANKIISEHDDRFKTDTNSSEFEEAENETCVGDPQYCNMTKEEYVKMIQEYIYPNPYEWILIATHTFVFITGLFGNALVCVAVYRNHSMRTVTNYFIVNLAVADFMVILFCLPATVLWDVTETWFLGEGLCKVLPYFQSVSVTVSVLTLTFISVDRWYAICFPLKFKSTTGRAKTAILIIWLVSLCFNIPELVVLKLVRFVPLRFELPYLLQCYGTWSPSSELVWHILKVLLIYTLPLVLMAVAYHQIARVLWSSNGIPGQADTKKLATAELTQLRSRRKAAKMLVSVVIMFAVCYFPVHLLSVMRYTIDMGQTEFITIWALVSHVMCYANSAINPLIYNLMSDKFRREFRRAFCCSTSPGQQDFTSMSRVTTKKDSSIMASFKPGHTSTTFVHNNKNGHMT

>PPU12514-RA ETH

MTIFNDTMYYEGDDVTIFASALVTNTPKPVNDFYQLPIYMQVLSVLICVIVMVIGIIGNLMVLIVILGAKDMRNSTNIFLVNLSIADLCLLLVCTPAILVEVNAGPEVWVLGEHMCKAIPFIESTIAHASVLTILAISFERYYAICKPLQANYVCTKSRATMICILNWIIAGFCTSPFLLMVTYKLEVDARGTLVPICGTEALTQWSIVYIATTIGAFFVVPVIVLMMLYSVIVYRLIKRSAIKHEMNRHALHNRNQVIRMLCTVISAFFICLLPFRAMMIWVIDHVLSELGAESDFLRAHVNQV

>NV14019-RA ETH

MTIFNDTTYYEGDEVTIFASTLVTNTPKPVNDFYQLPIYMQVLSVLICVIVMVIGIIGNLMVLIVILGAKDMRNSTNIFLVNLSIADLCLLLVCTPAILVEVNAGPEVWVLGEHMCKAIPFIESTIAHASVLTILAISFERYYAICKPLQANYVCTKSRATMICILDWIIAGFCTSPFLLMVTYKLEVDARGTLVPICATEALAQWSIVYIATTIGAFFVVPVIVLAMLYSVIVYRLVKRSAIKHEMNRHALHNRNQVIRMLCTVISAFFICLLPFRAMMIWVIVSPLEELANFGAEGYYCLLYFSRIMFYLNSALNPIFYALMSTKFKNGFLKILKAAFS

>BmA6-A

MISTINYTQSQTNVNILHVAYSSYGNDIENVTEYRTKSEAAVDLDDAFRNGSLTNTTIGYTNNNFTEYAEIPHYIKITSMTFCIAIMCLGVIGNVMVPIVILKTKDMRNSTNIFLVNLSIADLMVLLVCTPTVLVEVNSKPETWVLGKELCLAVPFVELTVTHASVLTILAISFERYYAICEPLRAGYVCTKTRATLICGLVWFFAALFTSPILAIADHKATSVNGTVVNQCLTQAGTVWEITFFVTIIILLYLLPLIILIVLYSIIAKNLITAASKVVMNKTVDPYNARARKQVILMLGTVVLCFFLCLMPYRALTLWIIITPSGFDGISSEKWYNILYFSRVMLYINSAINPILYNLMSSKFRIGFCKVCICYKKENDLNRRTQRTITNGSTTSSSLTRTTNSLKKFFGHRTSVDRSEAETNSKDEERSLFDRIFPNRAFLRQQSAPVCSNLNPNRINRMRSEGCMDINRPDNIHSNLNPKVIRSEIDADLPRANSLRRNVLINTAKAKSVDSERNVMNYSKKTKVDSVVAFQKSKSVDYEFPESFV

>BmA6-B

MISTINYTQSQTNVNILHVAYSSYGNDIENVTEYRTKSEAAVDLDDAFRNGSLTNTTIGYTNNNFTEYAEIPHYIKITSMTFCIAIMCLGVIGNVMVPIVILKTKDMRNSTNIFLVNLSIADLMVLLVCTPTVLVEVNSKPETWVLGKELCLAVPFVELTVTHASVLTILAISFERYYAICEPLRAGYVCTKTRATLICGLVWFFAALFTSPILAVATFTYEQDEDGTEVPVCLTQADTFWSALFFILTIAIFFIVPLGVLLVLYSVIAKNLMENPVIIAQSSKNTSGTGNVIRYRKQVILMLGTVVLSFFICLLPFKALTLWIIVFPPETIMSLGIDGYYILLYFCRVMLYLNSAINPILYNLMSSKFRDGFVKLLKINKLMRCSRNLRETMQRRDTFNTTTSTGFSSSQNTSDSFWRRYSNRVSSQKNILNNSKKIKEEKVNPIKIGEIINVENTRRNSMKFIAALNEDAQIDNEVEIADNENNKQIQILNLDVKTNSVYSITLDVSKEGKNRFVCVPAQDRDNKNIFIYDYNTKESFV

>DmCG5911-B ETH

MLPQIPSYIRTTAMFFCIVIMLLGVVGNVMVPIVIVKTKDMRNSTNIFLTNLSIADLLVLLVCTPTVLVEVNTRPETWVLGHEMCKAVPFVELTVAHASVLTILAISFERYYAICEPLKAGYVCTKGRAILICVLAWGIAALFTSPIIAISTYSVEPYGDGTDAPVCTTAADGFWSIFYFVGCITVFFFLPFGILVLLYAAIAYKLLRPNNAFHRPTSPQPQQPSGGATSGSSQVPSTKGNSHQQSNGMRKHRKQVIFMLVAVVSSFFVCLLPFRAFTLWVILASAEDVEGLGIAGYYNLLYFSRFMLYLNSAMNPILYNLMSSKFRSGFWRLLLTCLGQRPHHHHRHHYHQRQHPTAGGSGRNASTRQEQDAEEGAALAGTTSARHPRRTLRREATFLINSISTSSGTDRTTSSSAWRSNSLSISGLSERERGILGAAIIGTTAATVTTACLQERRASKI

>DmCG5911-A ETH

MLPQIPSYIRTTAMFFCIVIMLLGVVGNVMVPIVIVKTKDMRNSTNIFLTNLSIADLLVLLVCTPTVLVEVNTRPETWVLGHEMCKAVPFVELTVAHASVLTILAISFERYYAICEPLKAGYVCTKGRAILICVLAWGIAALFTSPILWVAEYKLAEYIDGSSVAVCLTQAISDWTLAFFLMTISVFFVVPFVTLVVLYGIIARNLVSNRAAMLRARPTKPELSLKARKQVVLMLGAVVLSFFVCLLPFRVLTLWIILSTDQTLHDLGLVRYYSLLYFCRIMLYLNSAMNPILYNLMSTKFRRGFKRLCQDAGRLLLELVTLGRRKEDSSRGRRGTLSLGMGTNTNTNTNSSNATGATSSSILSRSSNRRCSEDISRTRLKIEMQMPCGSDLEAMAMLQHSTLGKGIARRVSDSRLMPLRNHQPRRHKPQISFDEESLEENKRSEAKIPTKCREKLPGIAREIVNLTENTL

>PPU01679-RA sNPF

MSSPQPLNYSQNASEAAKDFITSNLAVRLVFTTFYASIFLLGLFGNALVCFVVARNRQMQTVTNLFITNLALSDILLCALGVPFTPSYTFLQKWIFGYYMCHLVPYAQGVSIYISTLTLTGIAVDRFLVILYPFRPRMKIGICLSIIAIIWVVALLLTLPYGYYVQFQSFGDIPYCEENWPDEQFRRTFSLLTSVLQFVLPFIVIAFCYICVSVRLNDRAKMKPGSKTSRREEADRERKKRTNRMLIAMVAIFGVSWLPLNIVNMVNDFYEPAQNWIYYKVLFFMAHCLAMSSTCYNPFLYAWLNENFRKEFKQMHLSCRNRLGYLVNLLVASPLRRLLPRASSSRGEHEVIGGNVCCTEHRTTDRLTPRGALL

>NV15762-RA sNPF

MSSSQALNHSQNASVLEPANDFITSNLAVRVVIITFYVSIFLLGLFGNALVCFVVARNRQMQTVTNLFITNLALSDILLCALGVPFTPSYTFLERWVFGDSLCHLVPYAQGVSIYISTLTLTGIAVDRFLVILYPFRPRMKIGVCLSIIVTIWIVALLLTLPYGLYMQFQSAGKIRYCEENWPDEQFRRTFSLLTSVLQFVVPFIVIAFCYICVSIRLNDRAKMKPGSKTSRREEADRERKKRTNRMLIAMVAIFGVSWLPLNIVNMVEDFYQPAQDWSYYKVLFFMAHCLAMSSTCYNPFLYAWLNENFRKEFKQMHSSFRNRLRFLVNLLVASPLRRLLPRASSSRGEHEVVGGNVCCTEH

>BmA7

MNETIVNYTDLNISNISEKITNATANGLPPFQANSHENIIDNKWVQAMFCVIYTIIFVLGLLGNILVCFVVIRNKAMQTVTNLFISNLALSDILLCIFAIPFTPLYTFRGTWSWGSLLCHIMPFAQGCSVYISTLTLMSIAIDRFFVIIYPFRPRMKIETCITVIIMIWTFSITVTTPYAIFMTYYDFKFGKFCEETWPSERLRRIFGSVTSVMQFVLPFIVIAVCYICVSFKLNDRAKAKAASKNSKKEELDKNRKRRTNQMLIAMVTIFGLSWLPLNIINLCNDYYMYAIHLKYYFLIFFVAHVIAMSSTCYNPFIYAWMNENFRKEFKQLIPCIDSSAQTRGNIQMEQLGAGPEKTFNGNTTTDSYLGSSSQRATSFRHKRKPSAAADVEKSGVELNEDLLTVDVKHCHISTSYNLRRESVKLRLINEESFDGTPSQSQF

>BmA10

MPTETWTSNDTTAYNGTSITVNHSNDNVNASFSDLIEYKGVQAAFCVAYTIIFAVGIFGNALVCYAVIRNRAMQTVTNLFITNLALSDILLCVFAVPFTPLYTFLARWVFGSLLCHIMPYAQGCSVYISTLTLTSIAIDRFFVIIYPFKPRMKIKTCLGLIIFIWFFALLVTFPYGYYMSLTDIYCEEKWPSDHIRKAFGAITTIMQFVIPFIVMAFCYTCVSIKLNDRLKSRPGSKNSKKEDAERERKRRTNRMLIAMVAIFGLSWLPLNLINMSTDFYSLTEIWKYYMLVFFLAHFIAMSSTCYNPFLYAWLNENFRKEFKQILPCLGAFVTKKSKRKFNQSDRTGMYRSEKTCNGNDTVQESLLTSTINKIPSVRYKIEFNDKLKGYDEEAVDNISPDEKPESNPSPNEDCLNMYMFADKSVISSDKEPIVSAL

>BmA11

MFGNDTLQDVMASTIAAKYEPAMSLNGTTYVGGGVLILTRTLTGESVEMIDEPKTNKTIDIIDVKLVQVAFCILYTIIFVLGVFGNVLVCYVVFRNKAMQTVTNLFITNLALSDILLCVFAVPLTPMYTFLGRWVFGRLLCHLMPYAQGTSVYISTLTLTSIAIDRFFVIIYPFHPRMKLNTCIFIVIFIWVFSLVVTCPYGLFMGIQTTNNETYYCEESWPSDRSRKIFGVFTTVLQFLIPFLVIAVCYTCVSIRLNDRARSKPGAKNSKREEADRDRKRCTNRMLISMVAIFGISWLPLNLINIFNDFYAQMTEWNYYFVSFFLAHSMAMASTCYNPFLYAWLNENFRKEFKQVLPFFESNGGVRNSYHPGRVPPHKTNKNVCNGNETIQETLLASSFNRGPSIKQRFEGNGKKDNGIEVENILLEDKTISATFHTKTENVNLQLIDEESHFSDHRDTKSPI

>DmCG7395 sNPF

MANLSWLSTITTTSSSISTSQLPLVSTTNWSLTSPGTTSAILADVAASDEDRSGGIIHNQFVQIFFYVLYATVFVLGVFGNVLVCYVVLRNRAMQTVTNIFITNLALSDILLCVLAVPFTPLYTFMGRWAFGRSLCHLVSFAQGCSIYISTLTLTSIAIDRYFVIIYPFHPRMKLSTCIGIIVSIWVIALLATVPYGMYMKMTNELVNGTQTGNETLVEATLMLNGSFVAQGSGFIEAPDSTSATQAYMQVMTAGSTGPEMPYVRVYCEENWPSEQYRKVFGAITTTLQFVLPFFIISICYVWISVKLNQRARAKPGSKSSRREEADRDRKKRTNRMLIAMVAVFGLSWLPINVVNIFDDFDDKSNEWRFYILFFFVAHSIAMSSTCYNPFLYAWLNENFRKEFKHVLPCFNPSNNNIINITRGYNRSDRNTCGPRLHHGKGDGGMGGGSLDADDQDENGITQETCLPKEKLLIIPREPTYGNGTGAVSPILSGRGINAALVHGGDHQMHQLQPSHHQQVELTRRIRRRTDETDGDYLDSGDEQTVEVRFSETPFVSTDNTTGISILETSTSHCQDSDVMVELGEAIGAGGGAELGRRIN

>BmA4

MPFYDDMGLDPSVNLTLNASAKQLIEASKGIQDPNILLEKFSQNRKVDDPTRSLLIAFYIILVVIGAVGNALVILSVVRKPVMRTARNMFIVNLAVSDALVCVVGTPLTLMELLTKHWPLPDWPSLCKACGAIQAISIFVSTISITAIALDRYQLIVYPTKPGVQTIGALVTMFFIWVTAFILASPLYIFRSLKTHKLGIAGISSLSFCIEDWPITDGRAIYSLLSLIFQYLLPVLVVVMAHIQIHRRLRGRRRTTRKTPAILIAIAVTYVISWLPLNVFNLVADFSSAPFKDEKTMTVTYAVCHMFGMSSAVSNPLLYGWLNDNFRKEFEEILTKCCCRKKPLVNGTRTTNRRMETELTALAQLEHTVTGNTKTSQCSQVF

>DmCG1147 NPF

MIISMNQTEPAQLADGEHLSGYASSSNSVRYLDDRHPLDYLDLGTVHALNTTAINTSDLNETGSRPLDPVLIDRFLSNRAVDSPWYHMLISMYGVLIVFGALGNTLVVIAVIRKPIMRTARNLFILNLAISDLLLCLVTMPLTLMEILSKYWPYGSCSILCKTIAMLQALCIFVSTISITAIAFDRYQVIVYPTRDSLQFVGAVTILAGIWALALLLASPLFVYKELINTDTPALLQQIGLQDTIPYCIEDWPSRNGRFYYSIFSLCVQYLVPILIVSVAYFGIYNKLKSRITVVAVQASSAQRKVERGRRMKRTNCLLISIAIIFGVSWLPLNFFNLYADMERSPVTQSMLVRYAICHMIGMSSACSNPLLYGWLNDNFRKEFQELLCRCSDTNVALNGHTTGCNVQAAARRRRKLGAELSKGELKLLGPGGAQSGTAGGEGGLAATDFMTGHHEGGLRSAITESVALTDHNPVPSEVTKLMPRLEQY

>BmA9

MNDPDISYNSSLLRMREITSTLAPFTTIRNASVAKPNNAYEWRFILPPYFVIFLLSICGNCLVIATLASNRRMRTVTNVYLLNLAISDFLLGVFCLPFTLVGQIYRRFLFGAALCKLIPFLQAVSVSVDVWTLVAISLERYFAICRPLKSRKWQTQCHAYKMIAMVWILSLILNSPIMLVSTLQPMRGNAHQCREVWSSLELERAFNLGLDAGLLLLPFFVMSFAYCLIVTKLWRGMRHEIQHNFNWQRHQTHQASYKNNQLLPATTIKKSNSADVCCNKTNQTNQKKPNKDTSEQVEPDARSTQPYCMHAVDHEFRHFVRSTHIDKSIEAKRKVIRMLFVIILEFFVCWTPLHVINTIYLFYPDQLYEHIGSKGIICSQLLAYCSSCCNPITYCFMNRKFRQAFISLSKSCGIFGLCCREKSESGKQAAPPPVSSSQEVTACVIRGSQTGRTELDGLEGKDCV

>DmCG6881 SK

MFNYEEGDADQAAMAAAAAYRALLDYYANAPSAAGHIVSLNVAPYNGTGNGGTVSLAGNATSSYGDDDRDGYMDTEPSDLVTELAFSLGTSSSPSPSSTPASSSSTSTGMPVWLIPSYSMILLFAVLGNLLVISTLVQNRRMRTITNVFLLNLAISDMLLGVLCMPVTLVGTLLRNFIFGEFLCKLFQFSQAASVAVSSWTLVAISCERYYAICHPLRSRSWQTISHAYKIIGFIWLGGILCMTPIAVFSQLIPTSRPGYCKCREFWPDQGYELFYNILLDFLLLVLPLLVLCVAYILITRTLYVGMAKDSGRILQQSLPVSATTAGGSAPNPGTSSSSNCILVLTATAVYNENSNNNNGNSEGSAGGGSTNMATTTLTTRPTAPTVITTTTTTTVTLAKTSSPSIRVHDAALRRSNEAKTLESKKRVVKMLFVLVLEFFICWTPLYVINTMVMLIGPVVYEYVDYTAISFLQLLAYSSSCCNPITYCFMNASFRRAFVDTFKGLPWRRGAGASGGVGGAAGGGLSASQAGAGPGAYASANTNISLNPGLAMGMGTWRSRSRHEFLNAVVTTNSAAAAVNSPQL

>DmCG6857 SK

MLPRLCADACRQCFAKIARRDTHRGTRTPYGCADTQSRPKPNFLLREVDEVCCTAASASPRLLVLFRDHKRASFFGLTIDAFYHYLRQALPLAKEAAIHLNASNEISAVGDGVTITGTPGDLLNYSGLELDLGLDLDLNLDMDLATTPSSSTLAPAVTVRTPGNRSVVRVSADVPIWVVPCYSAILLCAVVGNLLVVLTLVQNRRMRTITNVFLLNLAISDILLGVFCMPVTLVGTLLRHFIFGELLCKLIQFAQAASVAVSSWTLVAISCERYYAICHPLRSRTWQTINHANKIIAIIWLGSLVCMTPIAAFSQLMPTSRPGLRKCREQWPADSLNYERAYNLFLDLALLVLPLLALSFTYLFITRTLYVSMRNERAMNFGSSGPEVTTSSSAAVAEAGSQRRANGSHCQSLDTIVPHQHNPHQQHHHHSQYYYDYGHCGSKRRLISGGGPCEGRRHLYCMRSASVKSLRHQQINGGGGTLSGTGAGNGECCSRVHRMRQQMQLQQQGYVSDNESRRKSLSQPSLRITEAGLRRSNETKSLESKKRVVKMLFVLVLEFFICWTPLYVINTMTMLLGPTVYEYVGYTSISFLQLLAYSSSCCNPITYCFMNASFRRAFVDTFKGMRVCERLCAPCCFWRRRSKNETNLSVAGNSIALANSVMSSHTILESPRL

>PPU13001-RA

MAATALAATSAVMPPKLDNADDNSSLDMEQMSEILRGFYDDFNSETNYLLIGLYVPVIILAMTANILVIVVVFKYQYMRSVTNYFVVNLSVADLLVTMICMPMAVSQAVSIIWVYGELMCKLFFYLQGVAVAASVFTITAMSIDRYLAIRNPIAFRRVFNRKSTIIVIAALWVVALSIFAPVLRAVTLQSPITDLYNITLSGQWASDEPGVQMPKPPTFYVCSEDFKPLGIHAHIFGAACFVLVYAVPGFIVIIAYSMMGRTLCARKPPFDCDSIEGSASSQQGFRLVRERRRVAWILLLLAVLFALCWLPYNVLRLLVDLGVVHEGRLISDVLSYCLFLGHANSALNPIVYCFMTRNFRRSVAEILCRGNYGLARRKPHRKTLGSAVGVCAGCNNNSAGRAYYPKAHPGDSVGRGIFAPSGLVGQQHQQSMPGTAGPTSSLTLLQPGITGTTMIAGGVPTTALHQISSQGGQTTSSAPAHAVLALRTASNNCSPSNTGSSSGYDSFYSRHSPHRRCYMLRSLPQDQERHKAISPAKECNGDDCNVLVTSKKSLHRHSHSSRSSSSSSHSRIHRDSCDAGKQSSLISSDEQRFM

>NV20652-RA

MAATALAATSAVMPPKLDKAENSSLDMAEMSEILRGFYEDFNSETNYLLIGLYVPVIILAMTANILVIIVVFKYQYMRSVTNYFVVNLSVADLLVTIICMPMAVSQAVSIIWVYGELMCKLFFYLQGVAVAASVFTITAMSIDRYLAIRSPIAFRRVFNRKSTIIVIAGLWIVALSIFAPVLRAVTLQSPITDLDNITLAGQWASDEPGLQMPKPPTFYVCSEDFKPLGIHAHIFGTACFVLVYAVPGFIVIMAYSMMGRTLCARKPPFDCDSIEGSASSQQGFRLVRERRRVAWILLLLAVLFALCWLPYNVLRLLVDLGVVREGRLISDVLSYCLFLGHANSALNPVVYCFMTRNFRRSVAEILCRGNYGLARRKPHRKTLGSAVGVCAGCNNNSAGRAYYPKAHPGDSVGRGIFAPSGQAGQQQSTPGTAGPTSSLTLLQPGTTGTTMAGGAPTTAVHQASSQGNQSPSSTPAHAVLALRAASNNSASNTGSSSGYDSFYSRHSPHRRCYMLRSLPQDQERHKAISPAKECNDEDCNVLVTSKKSHRHSHSSRSSTSSSHSRIHRGSYDAGKQSSLISSDEQRFM

>BmA12

MTTMDTPNFSHVNGLNLSGNASGGLHLETTVDIFFQPEHVLITLYVPVILLSFIANILLIVVAIKCNYTKNVTDIFLVNLSAADLLVTGICMPIQLSKAITLVWFYGETVCKIVNYIQGVAVAASVFTISAMSVDRWLSITPEPRLRPPGRKQATLLLMLLWIAALLIFIPTSLVAGVRKETIPIISKGDKNISIETRDIHFCIEEWPSPETRKQYGMFSFTLVYAIPGSITIMSYACMGRTLCSVRPPFDIDEGNVSMQQGLRLMKERKRVAWILLLLAVLFALCWMPYNIMQLLLDVSVVNAKDLSAYLPYALFLGHANSAINPIVYCFMTRKFQRSVKKLLCGRPLCQQAKFTWRCNQKPEGSSSDYELYHEPHKRCYLQMNTLRYNGHYSAQIPRGIETRAQTTQLSHVTRSSRRTAPAHALSAEMLQRHGHIDYRR

>BmA35

MDRSEISFENMTFLEWNGSMGDELNMPHLDLEVFYRPDIVIIALYVGVLTASLSANTLLIFVVIKFQYMRNVTNIFLVNLSVADLLVTLFCMPVQIAKSVTLLWYFGEVMCKTVNFLQGVAVASSVFTITAMSVDRYLAITQSLRQPWMPSRRGACGLLVCLWLIALAIFAPLLAVAAVERERVPPLSRTKNGSQILVERTIEFCTEKWPDSIKKELYGAFSFILVYAVPGCIVVVSYSLMGRRLCSVLPPFDQTEGSANSQQRLRLVRERKRVALILLLLAVLFALCWLPYNILQLLVDVNAVQVDSVSLVLPYTLLLGHANSAINPIVYCLMTRNFRRSLRKLICHDPGNINSNTHFHVSLHTFSRLSLTSYWNSPLTLIVLTCFAANVSKAREG

>BmA17

MSYGNASDDAQTDEATHAHALATLLAMYVLVSLIGIIGNVSLMAALASGGAARLRTPQMLSACAADFLVCAASAPLAATRAATVETLPCHVTYYIETFPVAASTLSLVAIAADRCGAVHRGRGTTLCSRPFLAVAAVWSMALLLGAAAFTTTCITCPPLAAVHAVVAFCFPVIAVARCHWSVRVKLTALSLTARAAHGELPLPVPLMRRPTHVIIVAGVGPRERRDAVDVGDARSKKKLQPSLLGPQPQTSTLRSRRRLGNVLMGIAGIFAMCWCPHAAIVICGSFGIHVPEMVGHYALLLGYAHSALNAVAYWVLNRHALTSACTAWHLPQLRVREERPSSTNEAALGAFHPRLARPAPSPRPPPSSFLY

>DmCG13995

MNRDNLQQWWENSYRRQHPEPTDDLGLDSAELHLALQEPNQLPADYDYGNFSLGNPYDVDSEHSISPLTLLLLAVSYGLVVFGGVVGNSTLVLTLCSASSVRLRNPLLLAVCIADLLVTGISAPVTLLNLAMNRRTRSLPLVLCKVIHYVQVMPVSASTISFFMLSLDRYATVKHPRLAQLRQRRYLHVSLALLSWLASAAISTPFLFAYKIIAKSMVVKGGGAANTTPNPVSISCTSDLGANAMFMSFIIFHTIAVFVLPGIGVLLNHYGVRRKLCALSLTARAAHGELPLPIPILRRQTHMVIVTGCPNAQQAACGGGTTADDTSNGNGTGTGGGPMAVSPGDIQLHTLQPRQPGSAGSALEPGSYRSSNPISPRAMREIRAHSQRQRINRAGRGPATPGIPLPQTSTLRSRRHLANMLIASAVIFIACWAPHVFCIFYKNFGNNQQCSQTSVYFSLLLGYFYSAISPVIYWALNHNSLRQSPCAPIIRLRSMQNFLRSRFRTHTAPPPPSSTNEAALGAFNPKLIKLTPKQYRAQASSHYLY

>PPU08251-RA CCHa2

MYNITRHFNGSTSTIFSLEEEDEEDQYIPYEDRPETYIVPILFFLILVVGVAGNGVLVLTLLRHANMRNIPNTYVLSLALGDLLVIVTCVPFTSILYTIESWPWGLAVCKLSECAKDISIGVSVFTLTALSAERYYAIVNPIRRHVAGLSAKPLTILTVTLIWLLASILALPAALFSHIPTEPLKGNHSITICSPFPKEFGESYEKGMVLFKFLAYYAIPLCIISGFYLGMARHLELSTRNMPGEHPGAPHHCEQIRARKKVGKMVIAFVIIFFVCFLPYHVFMLWFHFYPASREVFDEFWHAFRIIGFCLSFINSCVNPIALYFVSGTFRKRFNEYLCCCIPSVRRAANAQGRFPRTRGEASTFYETSFNSTYRRHTQELNSSTLLNYSAGESKPA

>PPU08247-RA CCHa1

MAYGTTSLPEEFLLQHHEITGNESFTECLNSTSGCAEENYVPYGDRPETYIVPIVFACILVVGVMGNGLLMLTICRHSNMRNVPNTYVLSLAIGDLLVILTCVPFTFTVYVLDSWPFGLMLCKVSECAKDISIGVSVFTLTALSADRFFAIVDPMRKLHATGGGKRATRFTMLVATLIWLLAIFCAIPASFSYIRVFRVNKNVTFLTCYPFPEEFGPNYPRTVLICRFCIFYVFPLSIIAVFYMLMARHLVQSTRNIPGEMQGQVKQIKARKKVAKMVMAFVAVFAICFFPQHVFMLWFYLHPSAQEDYNAFWHYFRILGFCLAFMNSCINPIALYCVSGTFRKYFNRYLMCYTRALDVVSKVSFICLPGRRVTSPMHEDDAIPRNRRLPSRSFRMGLNNGYEIKVFGASIGGSRDQTITIE

>NV12199-RA CCHa2

MSEGTNAKVLIWLFYLNTPLPKNVYLHLINGAASYPAKFFNYNVISTQPIKPYIFQRMYNITWHLNGSTSTIFSLEEEDEEDQYIPYEERPETYIVPILFFLILVVGVAGNGVLVLTLLRHANMRNIPNTYVLSLALGDLLVIVTCVPFTSILYTIESWPWGLAVCKLSECAKDISIGVSVFTLTALSAERYYAIVNPIRRHVAGLSAKPLTILTVTLIWLLASILALPAALFSHIPTEPLKGNHSITICSPFPKEFGESYEKGMVLFKFLAYYAIPLCIISGFYLGMARHLELSTRNMPGDHPGAPHHCEQIRARKKVGKMVIAFVIIFFVCFLPYHVFMLWFHFYPASREVFDEFWHAFRIIGFCLSFINSCVNPIALYFVSGTFRKRFNEYLCCCIPSVRRTANAQGRFPRTRGEASTFYETSFNSTYRRHTQELNSSTLLNYSAGESKPT

>NV12201-RA CCHa2

MAYGTTILPEELLLQHQNTGNDSFAECLNATSGCSAEENYVPYGDRPETYIVPIVFACILVVGVMGNGLLMLTICRHSNMRNVPNTYVLSLAIGDLLVILTCVPFTFTVYVLDSWPFGLMLCKVSECAKDISIGVSVFTLTALSADRFFAIVDPMRKLHATGGGKRATRFTMVVATLIWLLAIFCAIPASFSYIRVFRVNKNVTFLTCYPFPEEFGPNYPRTVLICRFCIFYVFPLSIIAVFYMLMARHLVQSTRNIPGEMQGQVKQIKARKKVAKMVMAFVAVFAICFFPQHVFMLWFYLHPSAQDDYNAFWHYFRILGFCLAFMNSCINPIALYCVSGTFRKYFNRIQEVYVDDEAKLTLHGLQQHYLKLKEKEKNKKLFELLDELEFNQVVIFVKSVQRCNSLTQLLTEENFPTIGICKGMTQEERLTKYQSFKDFQQRTLVATNLFGRCLDIERVNIVFNYDMPENSDTYLHRVARAGRCGTKGLAITFDCDEADAKILNDVKERFDVNIAALPDEIDLASYIEGR

>BmA14

MTAEANYTMSAENDTDEYMPYDERLETYLVPILFAVIFVVGVLGNGTLVIVYARHRGMRNAPNTYIFSLALADLLVILICVPFVSIIYTLESWPWGELICRISESAKDVSIGVSVFTLTALSAERYCAIVNPFRKLQLRKLPLVCATFIWGAAFIFAIPAAIFSSTVTVELKDNVTIVYCTPYPKDWTNYSKGMTIAKALVYYGLPLIVITVFYSLMARRLLASTREMPGALQGGQGEAQAKARKSVACMVLIFVIVFFICFLPYHAFELWYHLSPTSLLDYNDWTHALRIIGFCLSFLNSCVNPVALYCVSGVFRQHFNRYLCCRRSALHPTCSSRLSRTAICETSFRSTHRHRCNRNPTTESVVISNYDYGSTNKKSNIITRNNADGVTILTIRDSNVFISGEIDDKRINR

>BmA15

MMQETNETYDNSTEIYQPYYERPETYIVPILFALIFVIGVVGNGTLVAVFVRHKAMRNVPNTYILSLALADLLVIITCVPFTSIVYTVESWPWGRTVCQVSEAAKDVSIGVSVFTLTALSADRYFAIVDPLRKLHATGSSKRATRLTIATAIGIWILAGLLATPAFIGSYLRPFVVNPTTQFLVCYPYPQEWGEHYAQIVVMVRFLLYYSLPLAVIALFYVLMAWHLVLSTQNMPGEMQGTQRQMRARRKVAVTVLAFVLVFAACFLPSHVFMMWFYFCPTAENDYNGWWHGLRIVGFCLSFLNSCVNPIALYCTSGIFRKHFNR

>DmCG14593 CCHa2

MYASLMDVGQTLAARLADSDGNGANDSGLLATGQGLEQEQEGLALDMGHNASADGGIVPYVPVLDRPETYIVTVLYTLIFIVGVLGNGTLVIIFFRHRSMRNIPNTYILSLALADLLVILVCVPVATIVYTQESWPFERNMCRISEFFKDISIGVSVFTLTALSGERYCAIVNPLRKLQTKPLTVFTAVMIWILAILLGMPSVLFSDIKSYPVFTATGNMTIEVCSPFRDPEYAKFMVAGKALVYYLLPLSIIGALYIMMAKRLHMSARNMPGEQQSMQSRTQARARLHVARMVVAFVVVFFICFFPYHVFELWYHFYPTAEEDFDEFWNVLRIVGFCTSFLNSCVNPVALYCVSGVFRQHFNRYLCCICVKRQPHLRQHSTATGMMDNTSVMSMRRSTYVGGTAGNLRASLHRNSNHGVGGAGGGVGGGVGSGRVGSFHRQDSMPLQHGNAHGGGAGGGSSGLGAGGRTAAVSEKR

>DmCG14484 CCHa1

MIANLVSMETDLAMNIGLDTSGEAPTALPPMPNVTETLWDLAMVVSQSTQWPLLDTGSSENFSELVTTETPYVPYGRRPETYIVPILFALIFVVGVLGNGTLIVVFLSVRQMRNVPNTYILSLALADLLVIITTVPLASTVYTVEYWPYGSFLCSLSEFMKDVSIGVSVFTLTALSGDRYFAIVDPLRKFHAHGGGRRATRMTLATAVSIWLLAILCGLPALIGSNLKHLGINEKSIVICYPYPEEWGINYAKSMVLLHFLVYYAIPLVVIAVFYVLIALHLMYSASVPGEIQGAVRQVRARRKVAVTVLAFVVIFGICFLPYHVFFLWFYFWPTAQDDYNAFWHVLRIVAYCMSFANSCANPVALYFVSGAFRKHFNRYLFCRGASGRRKKRGQHDTFCMHRDTSLTSTASKRFQSRHSCYQSTIRSCRLQETTITTLPNGGNQNGANISAVELALPVLQAPGHNEAHAPPSYGFLPLNEIVQQTRSSPAKFQESLLN

>PPU08836-RA SIFa

MSVCYSGLPPVASNRPRLQWLHGGEPGVSLRRSAVGLLYERTVLRPYPLRQYPLRPYPPAPVPPCASTRLRQYPPAPVPACARTAMGQNRHGLESAWARIAMGQNRHSSPTRMYQHTNRMSKSSICRSTPIQKTRDNLQAKTQVSCWSYISSYTAALLHELTLTLLIHRIVVSHSAMTSGQMAIICAIKMFYYSDTNKHQQQHHLHADLQYDGLRLSAPAAAASMLQPPSPLLEMASLRLPDSDEFDLERRRSGGQQSATGLPSGEQLGDLRGLVAARAEIFAEYVNGSVQDLLVLASTRRPSPFAGGGGSAGTMDAAGPEDDDEMQRLAGFFNCTNCNISVELVPDRWYRHSVAMSVVYFVAYCLVFVVGLIGNSFVIAVVYRSPRMRTVTNFFIVNLAVADVLVIVFCLPATLMSNIFVPWVLGWFMCKIVPYIQGVSVAASVYSLVAVSLDRFLAIWWPLKCQITKRRARMMIVIIWFIALTSTMPWLLFFDLVPIYSDDPNLKLCLERWPNPEDDSLFFLIGNLMLCYVLPMILISLCYVLIWIKVWRRHIPSDTKDDQMERLQQKSKVKVVKMLIVVVILFVLSWLPLYVIFARMKFGGKIADWEEELLPIATPIAQWLGASNSCINPILYAFFNKKYRRGFMAILKSGQCCGKLRYYETVAMMSSSTSMRKSSYYVNNNNSSTRRAFHGPPVHQDSNVSYIFNHTGV

>NV17731-RA SIFa

MVDQSSVAARHQHYHQHAAQHQQHHLHSDLQYDGLRLSAPAAAASMLQPSSPLLEMASLRLPDSDEFDLERRRSSGQQSATGLPSGEHLGDLRGLVAARAEIFAEYVNGSVQDLLVLASTRRPSPFAGGGGPAGTMDAASPENDDEMQQLAGFFNCTNCNISVELVPDRWYRHSVAMSVVYFVAYCLVFVVGLIGNSFVIAVVYRSPRMRTVTNFFIVNLAVADVLVIVFCLPATLMSNIFVPWVLGWFMCKIVPYIQGVSVAASVYSLVAVSLDRFLAIWWPLKCQITKRRARMMIVVIWFIALTSTMPWLLFFDLVPIYSDDPNLKLCLERWPNPEDDSLFFLIGNLMLCYVLPMILISLCYVLIWIKVWRRHIPSDTKDDQMERLQQKSKVKVVKMLIVVVILFVLSWLPLYVIFARMKFGGKIADWEEELLPIATPIAQWLGASNSCINPILYAFFNKKYRRGFMAILKSGQCCGKLRYYETVAMMSSSTSMRKSSYYVNNNNSSTRRAFHGPPVHQDSNVSYIFNHTGV

>BmSIFaR

MKMAPLRLPVDYYTDDFLNFSTQNPNNERHHTRHNHSHLRESHKNHVADMLSNSIIDAFNTRFVENSVLPDMEPLSSHMDLEERHYPSRMNGTLNRSDFGGDEFMYRHSGAMTAVYCAAYLLVFLVGLVGNCFVIAVVYRSPRMRTVTNFFIVNLAFADILVIVFCLPATLMSNIFVPWVLGWLMCKTVPYVQGLSVAASVYSLVAVSLDRFLAIWWPLKCQITKRRSRMMIVFIWIFAILVTTPWVFFFDLVVVFEENPNVHLCIDVWPNPLSEVLYFVVGNLIFCYILPMVMITMCYILIWIKVWRRSIPTDTQDAQMERMQQKSKVKVVKMLVAVVILFVLSWFPLYLIFARIKLGGPIKKWEEEMLPIVTPLAQWLGASNSCINPILYAFFNKKYRKGFVAIIKSRKCCGRLRYYETIALQSSSTSTRKSWHYNNNNPSITRRSPPVDKNAVSFIFSHTGV

>DmCG10823 SIFa

MMAASGRIRKRKHKSHTSGDVPSTTTSVPMPIPTMAPGKMVAETMEEAAALAGDYNNFTHNFVDLQNLLSFNELNGTSGSGGTAVSSLGSSSAIKLNNSAITDTLLGTVLTTATATVAPAASSLLATLAATTTASARGSLAGKSLAIADATSSTYYSNLLNLSPATTSLISAAAATKSYNDSALRWEQLDGSVDFGFDPLYRHSLAMSMVYCVAYIVVFLVGLIGNSFVIAVVLRAPRMRTVTNYFIVNLAIADILVIVFCLPATLIGNIFVPWMLGWLMCKFVPYIQGVSVAASVYSLIAVSLDRFIAIWWPLKQMTKRRARIMIIGIWVIALVTTIPWLLFFDLVPAEEVFSDALVSAYSQPQFLCQEVWPPGTDGNLYFLLANLVACYLLPMSLITLCYVLIWIKVSTRSIPGESKDAQMDRMQQKSKVKVIKMLVAVVILFVLSWLPLYVIFARIKFGSDISQEEFEILKKVMPVAQWLGSSNSCINPILYSVNKKYRRGFAAIIKSRSCCGRLRYYDNVAIASSTTSTRKSSHYHQNSSRKSPSSKGNAVSYIYEHNSLRRHNMMLKQDSNLSQQMLLKQDSHGSRQFLIKQESSCSDASGIRRPLCQQDSNGSKVSLSKQDSIVSYMEARRSAGHGLNDTLVDRDSVSMDVGRRQGATPSSLLDKRQKFVKQDSVISFVDQRPEQRRHQLVKQDSVISFADQRRGLLHKQDSLMANRTGDAPTHHVSILKKTDSQLSYGSSTSPRRNADLYE

>PPU05054-RA RY

MEICPENRPRKHYAGICTSSSSMTEEVENVTSVVGWLNESLSNETIIYDCDNFAGGPLSVQWFQAILYVLYSSVFVIALLGNVMVCYVVYKTGQQNRNKRSNIQNRQTNYYLVNLAIGDILIDLFCVPTSFISTLVLQYWPFRAELCPVVNYSQAVSVLVSAYTLVAISMDRYVAIKYVLKPRGSTKSAKFSIFIVWLLAIIVAFPILIVSGVDQPQRKYEVCERYVCIEIWSDQKQRYYYTFSLLILQFVVPLLILTFSYTSIAIMVWGKRPPGEAENNRDQRMTRSKKKMTKMMVAVVFVFSICWLPFNLLNLVMDYNESLRHWHGLPYVWASLHWLSMSHACYNPVIYCWMCTTFRRGFYSVLKRVPLVGRYIPDKSRAYNVGGIPLAGSDGQNNSSLRRINTYTTYVSVRRKTNHNYGAPIRSASFRCNNSLRNSGPMHRHFIHLEAQPEESL

>NvRYR

MTSTQDEICSGGDINCSMTEEPAIVTGTADWRNLSVLENGSLIFDCDDFNGNIPFTSTLAQVILYILYGSMFVIALGGNSLVCAAVIRTRNNLRPNNLTNYFIVNLAIGDILINIFCVPTSVLSTLVFHYWLLPSQLCAIFNFFQAVAVLVSAYTLVVISMERYLAIMYLFRPRRGTKYAKISILIVWLLAMAISLPILVVSDVEQPDIRYEMCDFYTCTEKWSDKKQKYSYTIALLILQYLVPLLILLFSYISIAIVVWGKKAPGEAEDNRDQRMIRSKTKTIKMLMAVVIVYTICWLPFNVLNLVMDINEDINRWYGLPYIWAMLHWLAMSHACYNPFIYCWMSTPFRQGIFNMLKCVPIIRRFVPDRSHALNTSAVGIPLTGFDGQHNSSLRRKNNCTTYVSVRKKMNHNHGAPIRSASFRCNNSLRSSGPMHRHFVHLEVQQEESL

>BmA19

MLDMYESESIDFDSSTLSDFLIRNVTSSEYYINATASSNLTKLDYDAICNPSTSSESFFTSATFQTCVYFMYCIVFVVALVGNGLVCFVVQTSPRMKTVTNYFIVNLAVGDILMTLFCVPFSFVSMLVLRYWPFGGIMCKVVNFSQAVSVLVSAYTLLAISIDRYMAIMRPLKPRMGKTAAKMVVAGVWGGAIATATPIYVVSKLERPAEWHKYCQLDICHEEWDHVEQSERYTCALLVLQFVLPLSALVCTYARIAHVVWGGRPPGEAESTRDSRMQRSKRKMIKMMVIVVAVFTVCWLPLNIFIVLWTLHEGDEEWAAWPGMPYVWFASHWLAMSHSCYNPIIYCYMNTRYRRGFKQALGWFFRVRFETTSPRCHHSSICEGMQMSEMVGVNGIVRRGTSSSCVSRLQRAPTCSSCASVRRGVAGGFTTPVPPIRALSVRTHFN

>BmA22

MSMDQSEELSNDMNYKATYAANVHHRNDTIFNQSAYNDTWPSHELFCIYESSSEDFLSSPVFQICVYFMYSAVFLVALLGNGLVCFIVHTSPRMKTVTNYFIVNLAVGDILMTLFCVPFSFVSMLVLRYWPFGAVMCKVVNFSQAVSVLVSAYTLLAISIDRYIVIMRPLKPRLGKGAAKMVVAAVWGGAIITAAPIPIVSQLQRPSPWHEACKVDICSEQWADGKQSEHYTFALLTLQFTLPLTALVYTYGRIAHFVWGGRPPGEAESGRDSRLQLSKRKMIKMMVTVVAVFVVCWLPLNIFIVLWTLHEGDEDWAVWPGMPYVWFASHWLAMSHSCYNPLIYCYMNAKYRHGFKQVLSGLFCLKLNESKRSCQRSSLCERIPLSGELIIFHS

>DmCG5811 RY

MEHHNSHLLPGGSEKMYYIAHQQPMLRNEDDNYQEGYFIRPDPASLIYNTTALPADDEGSNYGYGSTTTLSGLQFETYNITVMMNFSCDDYDLLSEDMWSSAYFKIIVYMLYIPIFIFALIGNGTVCYIVYSTPRMRTVTNYFIASLAIGDILMSFFCVPSSFISLFILNYWPFGLALCHFVNYSQAVSVLVSAYTLVAISIDRYIAIMWPLKPRITKRYATFIIAGVWFIALATALPIPIVSGLDIPMSPWHTKCEKYICREMWPSRTQEYYYTLSLFALQFVVPLGVLIFTYARITIRVWAKRPPGEAETNRDQRMARSKRKMVKMMLTVVIVFTCCWLPFNILQLLLNDEEFAHWDPLPYVWFAFHWLAMSHCCYNPIIYCYMNARFRSGFVQLMHRMPGLRRWCCLRSVGDRMNATSGEMTTKYHRHVGDALFRKPKICIRCKTLHLVSVSVFLFVLLRFWFI

>PPU04628-RA AKH

MSTALVNTTTAASLGYDDLPIDMRFNAGHVVSIVTYSILMIISAVGNITVLALLLRRRGNAARTRINTMLIHLAIADLLVTFLMMPLEIGWAATVSWKAGDAMCRIMSFFRMFGLYLSSFILICISVDRYHAVLRPLQMIDIDRRGRFMIAGSWICSALCSAPQMVVFHVEAHPTFTWYEQCITFNTFPSFTHELTYSLFGMVMMYWFPLIVIIYTYTSILAEMYRRSKDTTSDRIRRSSLGFLGRARVRTLKMTIIIVLVFFICWTPYYVMSLWYWIDSVTATKVDLRIQKALFLFACTNSCMNPIVYGAFNIRKGNKVTRNWDIHTLK

>NV16060-RA AKH

MTTAPVNATTVASLDYDDLPIDMRFNAGHVVSIVTYSILMIISAVGNITVLALLLRRRGNAARTRINTMLIHLAIADLLVTFLMMPLEIGWAATVSWKAGDAMCRIMSFFRMFGLYLSSFILICISVDRYHAVLRPLQMIDIDRRGRFMIAGSWICSALCSAPQMVVFHVEAHPTFTWYEQCITFNTFPSFTHELTYSLFGMVMMYWFPLIVIIYTYTSILAEMYRRSKDTTSDRIRRSSLGFLGRARVRTLKMTIIIVLVFFICWTPYYVMSLWYWIDSVTATKVDLRIQKALFLFACTNSCMNPIVYGAFNIRKGNKVTRNWDIHTLK

>BmAKHR

MDIDEKVSGPGGASQKNWSHLLHVNNTYDELPLEMRFNYSHMVSMTVYSVLMVISATGNLTVLYQLVRRRRAKRASRLDILLMHLAVADLMVTFLMMPLEIAWAGTVQWFAGDLMCRVMMFTRTFGLYLSSFVLICIAVDRYYAILKPLNVTWEATVRRAIIVAWVCAGLASLPQSFIFHVEEHPEVKGYNQCVSYGSLPTEKHEFAYFLVNMILMYVIPLVSTLYCSCAALFEIIRRANTANDKMRRSGIGLLGRARARTLKMTVTIVLVFFTCWSPYYCYCLWYWIDKESIKNLDPALQKAMWLFSCTNSCANPIVYGVFNRNRWNWRAGKFQNGRCRSGSGRKGSRLPHGESTEISAATLSRARHSNGSDHNGRRDSSYANQNGPQKHWNTINNNHVTNGMV

>DmCG11325 AKH

MAKVAEENDHRDLSNWSNVNDTNGTIHLTKDMVFNDGHRLSITVYSILFVISTIGNSTVLYLLTKRRLRGPLRIDIMLMHLAIADLMVTLLLMPMEIVWAWTVQWLSTDLMCRLMSFFRVFGLYLSSYVMVCISLDRYFAILKPLKRSYNRGRIMLACAWLGSVVCSIPQAFLFHLEEHPAVTGYFQCVIFNSFRSDFDEKLYQAASMCSMYAFPLIMFIYCYGAIYLEIYRKSQRVLKDVIAERFRRSNDDVLSRAKKRTLKMTITIVIVFIICWTPYYTISMWYWLDKHSAGKINPLLRKALFIFASTNSCMNPLVYGLYNIRGRMNNNNPSVNNRHTSLSNRLDSSNQLMQKQLTNNSLLNGRGQVMAAAVSATTKLANVVSLKGTANGNGSAAAAGTVPITPPLTVTIAPLATDDEANDDSCLSAVTIRCQDQSPIRQKCGESIELTSVVK

>PPU13782-RA ACP

MDQLQGTRMQLLQDFNNDFRNDSFYSYDFRDNMSMAMPTMPPSMTFTRRTLTIIIVYCICFLVAAIGNLTVFLTLWRGRYRKSRISLMICHLSIADLLVAFFTIPIEIGWRLTVQWIAGNYACKLFLFLRAFGLYLSNNILICVSLDRYFAVLYPLRVNDARRRGKLMLSVAWFFSVLYAIPQSIVFHVENHPNHKNFTQCVTFGAFPSDLVENTYNVFCVLTMYFIPLAIICWVYLKILCEISSKSRDNKPVVIKTGSNGTLESSNSNQGSRMRLRRSDMSSIERARSRTLKMTIIIVVAFIFCWTPYITMNLWYVIDKKSAKEVNEMVQESLFIMAVGNSCANPLVYGSYAIDLKKECCRCFLPCTTTKTNADVNLIQRSLGSKFQKPEMKSPGVSKQIVHSVCQAVHGFFKVGSGQTKSTAVCGKVLAPVSPRLSVSTTSKGAVVEKLPLHTIVHDLFGYSIPVFDISLNN

>NV14629-RA ACP

MDQLQGSRMQLLQDFNNHDFRDNMSMAVPTMPPSMTFTRRTLTIIIVYCICFLVAAIGNLTVFLTLWRGRYRKSRISLMICHLSIADLLVAFFTIPIEIGWRLTVQWIAGNYACKLFLFLRAFGLYLSNNILICVSLDRYFAVLYPLRVNDARRRGKFMLSVAWFFSVLYAIPQSIVFHVENHPHHKNFTQCVTFGAFPSDLVENTYNVFCVLTMYFIPLAIICWVYLKILCEISSKSRDNKPAGSNGTLESSNSNQGSRMRLRRSDMSSIERARSRTLKMTIIIVVAFIFCWTPYITMNLWYVIDKKSAKEVNEMVQESLFIMAVGNSCANPLVYGSYAIDLKKECFRCFLPCTTTKSNADVNLIQRSLGSKFQKPEMKSPGVSKQIVHSVCQAVHGFFKAGSGQTKSTNVCGKVLVPISPRLSVSTTSKGVVVEKLPLHTIVS

>BmA28 ACP

MDESTQMDVTACNDTTCSDTTSTPEQNFVIGVYSILLVIGAVGNVAVLISLLRNRRRKSRVSLLMTHLVIADMIVIFYFIPLEIGWRKTNAWLAGNVACKFLQVFRGFGLYLSSNVLVCISVDRFFAIIYPLRLAIARKRSKMMLYVAWAFALLLSLPQSAVFRVMEHPQIPDFKQCVSFEAFSNHQQELAYNVICLSAMYFVPLLVITICYLCIFYKISRNSKQNSEKEPPSNSRRVILRRSDQRPLVRARRRTLRMTVTIVTVFACCWFPYATMTLWYMLDWESAMRVPKRLQDFFFIMAVSNSCMDPLVYGSYTVDLRALILALRKIFCIRKEPTVLPGIKRPETITLVDQLRISQSRKRVRLSNPRDDLTTPRTSSEPFAYRAHHSFSERAIIMKPTHSCDDFTLSSPKKWYSA

>BmA29 ACP

MKMVNLNFDYEDTKEVPTSEIDKKFWEYFDATSTEISNYSSADLVPLDQGPVLATYAILLAIGGVCNIAVLVKLAKPRRRKSRVDMLMTHLALADVCVTCGVIPLEIGWKYTNAWLGGNFLCKLLLVLRAFGLYLSSNVLVCISIDRFFAVIYPLRLPEAKRRSRQMLYCAWVGALACSLPQSMVFRVKHHPRVIGFEQCVSFDAFNSYEQEVAYNVFCMCAMYFLPLIVITVCYVCIFCEIRKSSKELGDKYHSGLKPVRLRRSDRSLLERARRRTLRMTVTIVSVFALCWLPYAIMAMWYMVDRESASKVSRRIQDLLFAMAVSNSCMNPLVYGSYTLDIRGALRRFLKKCCSSTTPEVKGQAGSSSNKNANFDTPHITEPKNIRTRLGVRFAETSLTAVPERLEVPRAPRGPA

>NV10005-RA Crz

MYVLEEEPCLNIRNASGLISTNILRNSSCLGHAPQLTYGAYLRAVVLLSMTLLSFLANLATIWSIKSNKRKSQNCSAIYSLILHLSVADLFVTVFCMGGEALWSYNVAWIWGNTACKAFKFLQMFSLYLSTFVLVLIGIDRFVAVKYPMKTLNTAKKCNQLISFIWFISFILSTPQVVIFHVAQGPFIEDFSQCVTHGFYTEVWQEQLYTTLSLIFMFIMPLTILITTYMSTVITIARSERLFKSELANSSSAHKTGDVNRRRLIHRAKTKSLRISVVIVVAFVLWWTPYYIMMIIFMFLNPDKHVSADMQKGIFFFGMSNSLVNPLIYGAFHLWPQKKNRKHR

>BmA21

MDNEGNSTILYDANIMYPSELTLRTEFNTDGNNMNVCAIWPIEKCIEILKLNDTKTDDILGRSFIYNDTQLTCLEHAPVLTKTTVIRASVLSAMAFLSFIGNVATIISIRRSKRCRGRARPSWTAIYSLIFQLSIADLLVTIFCIAGEAAWSFAVQWYAGNIGCKLFKFLQMLALYLSTFVLVLIGVDRWLAVKYPMKSMATATRSGRLVIIAWVLSVILSIPQAVVFRVAKGPFFEEFHQCVTHGFYTERWQEQAYTTLSLVFMFILPLIILVSTYVSTVRTIAQSEKVFKPEVRRQEKYFTPDMNRRRLIDRAKMKSLRMSVVIVAAFLIWWTPYYVMMIIFTFLNPDKNQSEELLNGIFFFGMSNSLVNPIIYGAFHLWPRKKRSYQHSDRESGGHHASILRRGDNNTSSVRLTTIRSLRSSAKYSNGQNISLL

>DmCG10698 Crz

MEDEWGSFDRLPSVPSASMDLETENEVVSNWSTLANFTRLVAGAAPEIVNYTLNMIDVGVGMATDISNLSVSTTPLPAYAISNSSSLAHTNSRHEAPPMAEQVPEHVMDHAPQLSRSGLLKVYVLAVMALFSLLGNLLTIWNIYKTRISRRNSRHTWSAIYSLMFHLSIADVLVTWFCIIGEAAWCYTVQWLANELTCKLVKLFQMFSLYLSTYVLVLIGVDRWIAVKYPMKSLNMAKRCHRLLGGTYILSLVLSLPQFFIFHVARGPFVEEFYQCVTHGFYTADWQEQMYATFTLVFTFLLPLCILFGTYMSTFRTISSSEKMFQGSKLANYSTAKLPTQTNRQRLIHKAKMKSLRISVVIIIAFLICWTPYYVMMIMFMFLNPDKRLGDDLQDAIFFFGMSNSLVNPLIYGAFHLCPGKGGKSSGGGGNNNAYSLNRGDSQRTPSILTAVTQVDGTGGSSRQMRAFRQQSYYRSSSNGTAGPGAAPFKEQVGLLHVGPGNGTPGGSVSSGATPQLIRKGSALLARQPSCLREQEHQQRLLLHEKPSTLVVSYDSQRGGVGVGVASGLLDNNERVSSV

>BmA23

MDGSANTSQDDEADWPGNSTLDEYIAQNSTSDVYDTLYDVPTGVIVLLSFLYGSISVLAVVGNFLVMWVVATSRRMQSVTNCYIANLALADIVIGLFAIPFQFQAALLQRWLLPHFMCAFCPFVQALSVNVSVFTLTAIAVDRHRAIITPLSAHTSKRVAKVIIVFIWLLAFTLAAPMEMSWEVVMEDEIDPGTKLVYKKPFCTASEFGSNSLAIYRLLLYIFQYVIPLCVITFAYVHMAMKLWGARAPGNAQETRDANHMKNKKKVIKMLVLVVALFALCWLPLQSYLLLQSFFPSINEYRYINVIFFCFDWLAMSNSCYNPFIYAIYNEKFKKEFKQRFTFGKKPNRFANDSYEDGQSYRTRILSFRSTNDRCLYSTRKSINITPDDSLRLSTHSSVQYTNNQSRENGCECTKTEEAQARITARRYANMRMGCRHPNARKCFSKTNETDEMPIGDERVSELYIFPNSNIVEFRDISYDDKV

>DmCG10626 LK

MAMDLIEQESRLEFLPGAEEEAEFERLYAAPAEIVALLSIFYGGISIVAVIGNTLVIWVVATTRQMRTVTNMYIANLAFADVIIGLFCIPFQFQAALLQSWNLPWFMCSFCPFVQALSVNVSVFTLTAIAIDRHRAIINPLRARPTKFVSKFIIGGIWMLALLFAVPFAIAFRVEELTERFRENNETYNVTRPFCMNKNLSDDQLQSFRYTLVFVQYLVPFCVISFVYIQMAVRLWGTRAPGNAQDSRDITLLKNKKKVIKMLIIVVIIFGLCWLPLQLYNILYVTIPEINDYHFISIVWFCCDWLAMSNSCYNPFIYGIYNEKFKREFNKRFAACFCKFKTSMDAHERTFSMHTRASSIRSTYANSSMRIRSNLFGPARGGVNNGKPGLHMPRVHGSGANSGIYNGSSGQNNNVNGQHHQHQSVVTFAATPGVSAPGVGVAMPPWRRNNFKPLHPNVIECEDDVALMELPSTTPPSEELASGAGVQLALLSRESSSCICEQEFGSQTECDGTCILSEVSRVHLPGSQAKDKDAGKSLWQPL

>PPU14025-RA TK

MTLEETLLQPVLQLHSGISSATAILNSNASNLLLDQLSPSSIGSTTYPGAYYEFDGFINGTGNASLFDDSDAQNSNKFILPWWRQIIWTVLFAGMIVVATGGNLIVIWIVLAHKRMRTVTNYFLVNLSIADAMVSTLNVIFNYIYMLNSHWPFGNLYCKISQFIAVITICASVFTLMAISIDRYVAIVNPLKPRMGKRTTLCIAVAIWAVGAVLSLPMLLFYTTFTHNFPNGEVRVICYPSWPDQNNSGQSYNEYLYNVIFMILTYFLPIGAMTFTYARIGVELWGSQSIGEATQRQLDNIRNKRRVVKMMMVVVIIFAVCWLPFHVYFIVTSYLPELTNEPYIQELYLAIYWLAMSNSMYNPIIYCWMNSRYVIYVSTYTSEQV

>NV10406-RA TK

MTLEETLLQSVQPILNSSTSGLLLDQLSPSIIGSTTWPGAFYELDGFINGTGNASLFDDSDAQNSNKFILPWWRQIIWTVLFAGMIVVATGGNLIVIWIVLAHKRMRTVTNYFLVNLSIADAMVSTLNVIFNYTYMLNSHWPFGNLYCKISQFIAVITICASVFTLMAISIDRYVAIVNPLKPRMGKRTTLCIALAIWAVGAVLSLPMLLFYTTFTHNFPNGEVRVICYPSWPDQDNSGQSYNEYLYNVIFMILTYFLPIGAMTFTYARIGVELWGSQSIGEATQRQLDNIRNKRRVVKMMMVVVMIFAVCWLPFHVYFIVTSYLPHLTNEPYIQELYLAIYWLAMSNSMYNPIIYCWMNSRFRRGFAQFFSWCPLVRIGHEPALSRSEAVTSRYSCAGSPEIRARISRNGKSFSTVIES

>BmA24

MMLDELGPTVASNQSTSLADLDSFYVTFYDVENERYVNDSQNATEPFQSFILPWWRQILWTVLFAGMVVVATVGNLVVIWIVLTNKRMRSVTNYFLVNLSVADAMVSTLNVTFNFTYMLNSNWPFGHFYCKFCQFIAVLSISASVFTLLAISVDRYVAIMSPLQPRLGKRATLGITAAIWAWSSFISSPNLIYFTTENVSLPDGTIRCVCYSHWPDGMTTRSRLEYAYNVLFMVLTYFMPIIAMTYAYSRVGVELWGSQSIGECTQRQLDNVKSKRRVVKMMIVVVVIFAVCWLPFHVYFVVTSYYPDVVSYPHIQEIYLGIYWLAMSNSMYNPIIYCWMNSKFRRGFKQFFWCCGAFGGGGLARHRALGPDRTDRSMRSLSPSRKNGTSM

>DmCG7887 TK

MENRSDFEADDYGDISWSNWSNWSTPAGVLFSAMSSVLSASNHTPLPDFGQELALSTSSFNHSQTLSTDLPAVGDVEDAAEDAAASMETGSFAFVVPWWRQVLWSILFGGMVIVATGGNLIVVWIVMTTKRMRTVTNYFIVNLSIADAMVSSLNVTFNYYYMLDSDWPFGEFYCKLSQFIAMLSICASVFTLMAISIDRYVAIIRPLQPRMSKRCNLAIAAVIWLASTLISCPMMIIYRTEEVPVRGLSNRTVCYPEWPDGPTNHSTMESLYNILIIILTYFLPIVSMTVTYSRVGIELWGSKTIGECTPRQVENVRSKRRVVKMMIVVVLIFAICWLPFHSYFIITSCYPAITEAPFIQELYLAIYWLAMSNSMYNPIIYCWMNSRFRYGFKMVFRWCLFVRVGTEPFSRRENLTSRYSCSGSPDHNRIKRNDTQKSILYTCPSSPKSHRISHSGTGRSATLRNSLPAESLSSGGSGGGGHRKRLSYQQEMQQRWSGPNSATAVTNSSSTANTTQLLS

>BmA32 NTL

MPPWAHNSWICVFSIMLIIAVGGNAIVIWIVIAHKRMRTVTNYFLVNLSLADLMMSALNCLFNFIYMLHSDWVFGLQYCKISNFIANVTVAASVFTLTGISFDRFQAIVRPMRPRMSKTCSLIAIGGIWLGGMVLATPYLLYSTTKEYKSRVGVKTACLLVWPDGMPDVSKMDFVYQIAFFIVTYAVPMVGMSFFYTAMGRELWGSRTIGELTQRQLDSIKSKRKVVKMFILVIVIFGICWFPYHGYFIYTHLDSSILYSRYVQHVYLGFYWLAMSNAMVNPIIYYWMNAKFRSYFRMAIMCRWLEVMWRRRHPLDSPPECPSQSNTRSRSGFYSLTYRGMQRIKRKYSSRMGAPHASDPQRPALAETVFAC

>BmA33 NTL

MDSDLDIQSFINCTQQIFGHEQKWDDLNVSEILDLLPKQILEDINLKITLGNCMGLGERPYSPPWWGQLAWFIVFAVMLLLAVIGNTMVIWIVLAHRRMRTVTNCFLVNLAVADLLMATLNGAPNFVFLVTANWPFGAVTCTASNFTASLTVSAGVFTLVAITVDRYVAIVKPLQHRLSRRVVRAALFTVWIASAMLALPSLLYSDTYKKQYVNGEREICFIKWPDGSYPTSLSDYCYNLVFLSVTYVLPMAVMVWAYAQMSAALTGRAIGECTLHQMQVVRAKRKVVRMFVLVVMVFALCWLPYHAYFVLVYHHQSLATAPFAQHIYLGFYWLAMANSMFNPLIYYWMSNKFRLYFRLVLCWCWKSESATPNDLKKLEVKSYSVSQRHFRDVSSFSRA

>DmCG6515 NTL

MSEIVDTELLVNCTILAVRRFELNSIVNTTLLGSLNRTEVVSLLSSIIDNRDNLESINEAKDFLTECLFPSPTRPYELPWEQKTIWAIIFGLMMFVAIAGNGIVLWIVTGHRSMRTVTNYFLLNLSIADLLMSSLNCVFNFIFMLNSDWPFGSIYCTINNFVANVTVSTSVFTLVAISFDRYIAIVHPLKRRTSRRKVRIILVLIWALSCVLSAPCLLYSSIMTKHYYNGKSRTVCFMMWPDGRYPTSMADYAYNLIILVLTYGIPMIVMLICYSLMGRVLWGSRSIGENTDRQMESMKSKRKVVRMFIAIVSIFAICWLPYHLFFIYAYHNNQVASTKYVQHMYLGFYWLAMSNAMVNPLIYYWMNKRFRMYFQRIICCCCVGLTRHRFDSPKSRLTNKNSSNRHTRGGYTVAHSLPNSSPPTTQTLLAVLAQTLTQPKPQTQLLLSHHSPHPTQPSAAETKSQWKRSTMETQIQQAPVTSSCREQRSAQQQQPPGSGTNRAAVECIMERPADGSSSPLCLSINNSIGERQRVKIKYISCDEDNNPVELSPKQM

>PPU03425-RA CCAP

MNFFIKQLAFADLMVGLISVLTDIVWRSTVAWYAGNVACKIIRFMQVVVTYSSTYVLVALSIDRYDAITRPMNFSRSWCRARALVTAAWSISVLFSVPIIFLYEEKIVEGKNQCWIELGSPANWRIYMTVVCLTLFIIPAIIIGGCYMVIVWTIWSQSSALRHDPTRDTRRASSRGLIPRAKIKTVKMTFVIVFVFILCWSPYIVFDLLQVYGHVPRSQTNIAVATFIQSLAPLNSAANPIIYCLFSTPFCKTVRNMQAVSWFSGLCPSNRHHCFGTNTHGNSTRTTVTTSLTAHSSRRSGHISMLHPSSRKRVMVSLV

>NV17097-RA CCAP

MDFQLPSETWVSASTWPIETALGGILDASIVGYLSTEVSTLNNISRIIERNITDEIDPFYFYQTEQFTVLWLLFSVIVVGNTSVLVGLIFGKRRKSRMNFFIKQLAFADLMVGLISVLTDIVWRSTVAWYAGNVACKIIRFMQVVVTYSSTYVLVALSIDRYDAITRPMNFSRSWCRARALVTAAWSISVLFSVPIIFLYEERIVEGKNQCWIELGSPANWRIYMTVVCLTLFIIPAIIIGGCYMVIVWTIWSQSSALRHDPTRDTRRASSRGLIPRAKIKTVKMTFVIVFVFILCWSPYIVFDLLQVYGHVPRSQTNIAVATFIQSLAPLNSAANPIIYCLFSTPFCKTVSNMQAVSWFSGLCPSNPHLCFGTNTHGNSTRTTVTTSLTAHSSRRSGHISMLHPSSRKRVMVSLV

>BmA26

MDADIMMEGFETDMTTPNYAVNPIMTPMTAADHNHTWENISNATTPGSTINVYYFYDAAQFTVMWILFVSIVVLNSSVIAALLCTNARKSRMNFFIMQLAIADLFVGLTYVFPDILQKIIIAWYAGEFMCKTVKFLQAVVMYASTYVLVALSIDRCDAITNPMNFSGSWNRARVLVVSAWLISVIFSIPLFILYEVKEVQGELQCWIDLGNPKRWRIWVTLVSMMIFILPALTIAACYAVIVLTIWTKSKAVVMSPPISSRRTKTMRNGQIESDPDSRRASSRGLIPRAKIKSVKMTFVIVFVFVLCWSPYIVFDLLQVYGHIPSTQHYSAIATLIQSLAPLNSAANPLICCMFSPYIYTSLRRVPPYKWIWWLGRHKRAGRSTLRSRSDSTAHSDLLSSTHARRSHSVATILNRTRSSSVSRPQSEARKTQLLVLASARG

>BmA30

MEEPCMNCSFFAAFNDTQNEGNYTGNVTFINKFYFYQSAQLAILWILLVTIVAGNATVVLALLLTKSRKSRMNFFIMQLAIADLLVGLISVLPDLIQRVTITWLAGSITCKMMKYLQGVVTYSSTYVLVALSVDRCDAITHPMNFTGSWRRARALILCAWLLSFFFCIPMLLLFNEADIEGILQCWSSISKIQWRIWMTSVFVSLFVAPALIISACYGVIVVTIRQKSHRVLGRRATTTRQYSDDLDSRRASSRGIIPKAKIKTVKMTFVIVFVFVLCWSPYMIFDLLQVYGYVPDTQVNVAIASLIQSLAPLNSAANPVIYFIFSNRIFVSLKNIPPYKWLTCLMKGNDSPAGNESRAHTELLTSSHRRTRHDLTIRVKDDSVKFPKPRVQHRSSTNSRKVRLHLPDGQHQVNNNCHTVRRRDDTFL

>DmCG6111 CCAP

MLHLRLFDSSLYYTLASASESSGLASSTSTERSFNGTQGAGGVAVGGESLTPTDVAAVNLTYFTPAISHVMLAPTTIATTTASATMVQIQTTAAPSHDLETGGNSTSSDPGEFDNLNSFYFYETEQFAVLWILFTVIVLGNSAVLFVMFINKNRKSRMNYFIKQLALADLCVGLLNVLTDIIWRITISWRAGNLACKAIRFSQVCVTYSSTYVLVAMSIDRYDAITHPMNFSKSWKRARHLVAGAWLISALFSLPILVLYEEKLIQGHPQCWIELGSPIAWQVYMSLVSATLFAIPALIISACYAIIVKTIWAKGSIFVPTERAGFGAAPARRASSRGIIPRAKVKTVKMTLTIVFVFIICWSPYIIFDLLQVFGQIPHSQTNIAIATFIQSLAPLNSAANPLIYCLFSSQVFRTLSRFPPFKWFTCCCKSYRNNSQQNRCHTVGRRLHNSCDSMRTLTTSLTVSRRSTNKTNARVVICERPTKVVTVPAMSEV

>PPU12877-RA AVLP

MDVERLHTAACQRTLQKRIASPGIIMEESTTSMIVLPAEDWRDESLAVWEIIVLALILTTTLMGNVLVLFAIYLKKCRGRRQRLTRMHFFVMHLSVADLITGLLNVLPQLAWDVTFRFQGGPILCKLVKFCQPLGSYLSSYVLIATAVDRYHAICYPLSYCRTTSRRSRITVYVAWLLALLLCVPQVFIFSYQEISAGVWDCWATFTVPYGERAYVTWYTVTVFLLPFCVLTFTYAEICCSIWRNREVMVLASHERQQALTKEGRSQTSLISKAKINTVKQTLAVVTLYAASSIPFVGCQLWATWDPVASTSAFFDGPIFTILSLLSSLTSCVNPWIYLTFSYELRAALTKFLRSLIKRDRSSRFERASSNANSNETRSSKRSSFISRMSRYTSFIIYRSNANDRNSK

>NV17951-RA AVLP

MEESATSMIAAPAEDWRDESLAVWEVIVLALILTTTFMGNVLVLFAIYLKRCRGKRQRLTRMHFFVMHLSVADLITGLLNVLPQLAWDVTFRFQGGPILCKLVKFCQPLGSYLSSYVLIATAVDRYHAICYPLSYCRTTSRRSRITVYVAWLLALLFCLPQVFIFSYQEISAGVWDCWATFTVPYGERAYVTWYSVTVFLLPFCVLTFTYAEICCSIWRNREVMVLASHERQQALTKEGRSQTTLISKAKINTVKQTLAVVTLYAASSIPFVGCQLWATWDPFASSSAFFDGPIFTILSLLSSLTSCVNPWIYLTFSYELRAALTKFLRSLIKRDRTSRFERASSNANSNETRSSKRSSFISRMSRYTSFIIYGPMRTIEIVNKLYI

>TcAVLP

MYTPKLSQMDISENSTYLFDKHEDRNNTDRDENLARVEVATLAIIFLVTVIGNSTVLLALWTRRRYAGRKKLSRMYFFILHLSIADLITAFLSVLPQLAWDITYRFYGGFLLCKVVKYGQTLGPYLSSYVLMATAIDRHQAICYPLTYCSWTSRRSKVMVYLAWVASLAFCIPQLTIFTYTSVGEDEYDCWATFQEPWGKRAYVTWYSISVFMVPLVVLIFTYTSICIEIWQSSESSLRPRSSQKSAPGKRTPLISRAKINTVKQTIAVIVMYIACSTPFILAQLWATWDPQSPFIDGPVFVILTLLYSLNSCVNPWIYLAFNRELPRLLLRHYTASSKNYRSATGGNSASNSSGDAQSTSLRPFSRWSLCNSARSNKYPTRVPHRPYVAQYNARRWIVTTTT

>BmA31

MIDGTSSNMHHVRLLIGVEEKAMWSSLNKTTSGPEDVNVSRINETGPLYNEDEYIFDRTDVRAIFITLYTIVFCCCFFGNLLVILVVTLSRRLRSITNFFLANLAVADLCVGVFCVFQNLTIYLIPSWIFGDFLCKMYQFVHSLSYTASIFILVVICTERYFAIIHPITCKQILTSTRLRLVILGVWITSAAYSAPKFIWVETITNDLGNGQMETICIPHRRKYNSEIFDMVNFGLLYVTPLCVMTVLYTRIAVGLWQSSHTLQSLGRVQCCATECPRPEKPRTANYEPQRTFVGATENKNGVSTKVKGPPAKNVRTYEPRDCHHLSHLSRNVLRARRGVVRMLIVVVLTFAICNLPFHARKMWQYWSSGYEGSSDFSTLLTPLTFLITYFNSGINPLLYAFLSKNFRKGMKELLFCNYRAKRKNDQIIVLNPVGGGVLRRSSTRSTRANCSTITMAANGDS

>DmCG14003 TR

MIMTMMQTVRAWQQESDVEHRKQHKQRWRPDGAHISAAYDLNSDNDDGHHRVVHNQNNGSPNSSPNQSTSAFRQRQPHHPPTGQQPPRLPCTVTHFSAHWKTLLILLTLLSASTLTASANVTSTISPPINGSSTDYILLYGESTTSLVPALTTGLSGDGSGAVIEDEEDAEKASEYIFDRTDVRIIFITLYTLVFCCCFFGNLLVILVVTLSRRLRSITNFFLANLAFADFCVGLFCVMQNLSIYLIESWVFGEFLCRMYQFVHSLSYTASIFILVVICMERYFAIVHPITCKQILTAARLRMVIVTVWITSAVYSTPKFVFSKTIKNIHTQDGQEEEICVLDREMFNSKLLDMINFVLLYVMPLLVMTVLYSKIAIALWRSSRGLTPHVVQHQHQQPQQPSCQDIGMGMHNSMYHHHPHHHHHHHQHHQLQSAASSAGVVGVGLGGGGGGGPGPSLASGGSSTTSLSRKQSSKYEKRGVSITESQVSLEADRPIVSACRKTSFYHHGHAHHQRAGNASVGGGSGGAGAGATHMSHSSSNVLRARRGVVRMLIIFVLTFALCNLPYHARKMWQYWSRSYRGDSNFNALLTPLTFLVTYFNSGVNPLLYAFLSRNFRKGMKELLLCSWKKGKGKSSSNSSMHHKRKALQTHSLPTDTTHIGNEQL

>Hheb007010.1 Orphan

MAVAPINKILTALATADMFVMIEYIPFSIYYYIIFPNRATFPYFGAVFVLFHMHFAQLLHTISIALTLSLAVWRYLAIRFPQHNHAWCSDARCKMALWCSLAVSAFACSPSYFVFEIHEQPVQENGITEILYYVNADSYSGSGIAYQINFWVLAVVVKLLPCLILTVISCWLIKELYSYALFLLALFRAIYSKEPGEENKGEIME

>Hheb077040.1 FMRF

MKSSINYLLIGLARCDTVLIITSVLIHGLPAIYAYTGLLFDYKFGVFPQIVRYLYPLSCMAQMVTVYLTLTVTMERYVAVCHPLRARAFCTYGRARLAVLSIVIVSIIYNMPKFWEVDLEKEIHWKYNVTVYCVVPAILRSSDLYITIYVNWMYFFVYYAFPFVALVVFNVAIYRR

>PPU04456-RA RFaR

MTLEPVFNWTTSEDNNSSSLVGNLSIVATSLGPPKECELPVNDGFLEFFVYGILLNVVSFLGILGNAISIIVLSRPQMKSSINYLLIGLATCDTILILLSVLVYGLPGIYAYTGYLFHYKFFVYPKIVRYLYPLSTTAQMATVYLTLTVTMERYVAVCHPLKARSLCTYGRARTALLLIALISILYNLPKLWEVRLGEEIHWRYNITVYCIGATQLRENSLYKTIYVHWLYFFVYYAFPFVALVIFNVAIYKRVRKANRDLRRLSRHQRREIGLATMLLCVVIVFLVCNVLPLVSNVYENMYVNPPGWMIQMGNLLVTINSGVNFVIYVIFGRKFKRIFLKIFCRVSGETGLDCMGRPSRADSPDFQTNEDSIATNLSNVELRNSIRRLHGSHYHHHHGSHHGSNSLRPGGGSTHSGGSNGFSRSSVYYPTSPKPGFHNNGSNHSQHNNHQHHHRDRSTNLEDTTFC

>PPU05580-RA MS

MSNASAGYNFTCGSGVDSFHTSYVAIHGWASLLVCIFGSIANGLNIAVLTRREMSSPTNAILTGLAVADMLVMIEYIPYAVHSYLYHRPKRETYTYAWTAFVLFHSNFAQVFHTISIWLTVTLAVWRYIAVAHPQKNREWCSYNRTILAIVAAYVICPLICFPLYVTTEVTSKNVTLDANDRQVNLTGNGSSLRYLQSGAEANNATLYFVALTESAQNGLKEMNFWMYSVVIKLIPCVALTILSLRLIMALVEAKKRRKKLTSTTMLKMEESINLAESKKRKRKASRMMDKERQTDRTTKMLLAVLLLFLLTEFPQGTLGLLSVVLGPDFFNTCYVKLGEAMDILALINSAINFILYCAMSRQFRTTFKQLFCRWKLLGRWLPVPQHAENNNGNMTTNHTVTQVTQV

>PPU04714-RA MS

MSETAQPGQPAGQHPQEQPSAHQLSNYTELLRRLNITEEDLDYVNNFGSSAGIGGDTGGGCSCGPCHCGGLVRRIAASYRSYHGYVALLVCGFGTLANLLNVAVLTRKELRRAPINRILTGLAAADVLVMLEYVPFAIYEYIVLPERRHFPYGWAVFVLFHMHFSQLLHTISIALTLTLAVWRYIAVRFPQCSRSWCTPARCRLALLCSLLVAGLACAPSYLVFGIREQRLMEENGPVVLYHVDASRGPSSDDDADDRGLLYRLNFWLLGVLVKLLPCFVLTLISCRLIQALYKAKARRRLLRPLDGQLTDAPATGGRSERRSDRTTRMLVAVLLLFLITEIPQGVLGLLSALLGDCFFRSCYHSLGEIMDILALFNGAVNFILYCSMSRQFRTTFGRLFKPSIVVGKWQPACTHQTDIQSTYV

>PpCNMaR-2

MNALVSLMAQRGPARCGWLLAQGRVRRAIAFQNETDAVLEQRLEDQPVVPHHQLPARNHSYEHGFETGFETGFETGIEAYEEEPGDECHSLYFVLDFSHQYYIPFIILLGLVGNLLSCIVFLNTHLKMRSSSYYLAALATADFSFLFSLLLVWLNNSIGWRVFNKDGWCETVVYVSSVCSSLSVWLIVAFTVERFIAVQYPLHRPHMCTIARAKTIVLALVVLAMASHSYSFVTAGVVKTQDGNEMCDMKYEYLETMRIVSIVDSIASLMAPLVLIIVMNTMITRNLIRFSRRFGESSSGGLSTETRCPSRERSDINLNPIPRITCRFSDVRSSSRNLVSTRNQQSITKMLLLISTVFILLNLPSYVIRLCVFFFTLARKDTPSLLWCLQQFFMLLYYTNFSINFLLYAMCGITFRRCLEQLLRKALKSLTRYHCNPQRYI

>PpCNMaR-1

MSRSNNSTTTSTMEGENGYSFSYPFAWSLINGLEMYYLPALVVFGSLGNCLSVYVVFATKMRRTSSSFYLAALALSDTGFLMSVLIAWLTLINVHIMNQQGFCQFFVYLTNICSFLSAWCFVVAFTVERFVAVCYPLRRQWMCTVNRAKILISCVTAAGLILCSPVLLYSRPRLLENSTKPVCYIAEGWDKVASALNFLDTILTFALPFSLIVVLNSLIVRAVWHVDSVRTSLKARASTKDSPPAVHQARVTKMLLVVSSVFFCFNLPAYAMRVYAYLQADGAATEVEFLAQKACNLLFNTNFGISFGLYCASGQNFRAAVAQLFHCQRQLKLRPGMSKRSSTNQINHHGGTETIRISGSVSGDHTIVFEKPWRDSFQLRPYPRSLQRPVADCESAEDCNTPRKDYSAQM

>NV22020-PA CNMaR-2

MNALVSLLAQRGSARCGWLMDESRVRRAISFQDETEASLGQPADGRPATSHHQVPARNRSFEAGIEAYEEEAGDECHSLYFVLDFSHQYYIPFIILLGLVGNLLSCVVFLNTHLKMRSSSYYLAALATADFSFLFSLLLVWLNNSIGWRVFNKDGWCETVVYVSSVCSSLSVWLIVAFTVERFIAVQYPLHRPHMCTIARAKSIVLALVVLAMVSHSYSFVTAGVVKTQDGNEMCDMKNEYLETMRIVSIIDSIASLMAPLVLIVVMNTMIMRNLLRFSRRFGQNSSGGLSTETRCPSRERSDINLNQIPSGSSSNNGANGIALSSGIGARRQPSQQSFQSSKNNHSNHSRTQQQQQQQQQQQQTTTTSPPQAQQAPSTIVPPSTPRNAYVLPEITSRCIHVRSSSRNLVSTRNQQSITKMLLLISTVFILLNLPSYVIRLCVFFFTLARKDTPALLWCLQQFFMLLYYTNFSINFLLYAMCGITFRRCLGQLLRKILKSLTRYHCNPQRYI

>NvCNMaR-1

MSRSNDSTTAAAMEGENGYSFGYPFAWTLINGLEMYYLPALVVFGSLGNCLSVYVVFATKMRRTSSSFYLAALALSDTGFLMSVLIAWLTLINVHIMNQQGFCQFFVYLTNVCSFLSAWFVVAFTVERFVAVCYPLRRQWMCTVNRAKILISCVTAAGLVLCSPVLLYSRPRLLENSAKPVCYIAEGWDKVASALNFLDTILTFALPFSLIVVLNSLIVRAVWHVESVRTSLKARASTKDSPPAVHQARVTKMLLVVSSVFFCFNLPAYAMRVYAYLQADGAATEVEFLAQKACNLLFNTNFGISFGLYCASGQNFRAAVAQLFHCQRQPKLRPGISKRSSTNQINHHGTETIRISGSVSGDHTIVFEKPWRDSFQLRPYPRPLQRPVAVCESIEDCYTPRKDYTAQM

>NV11638-RA FMRF

MTLEPVFNWTTSEDNNSNGSSVGNLSIVATSLGPPKECDLPVNDGFLEFFVYGILLNVVSFLGILGNAISIIVLSRPQMKSSINYLLIGLATCDTILILLSVLVYGMPGIYAYTGYLFHYKFFVYPKIVRYLYPLSTTAQMATVYLTLTVTMERYVAVCHPLKARSLCTYGRARTALLLIALISIFYNLPKLWEVQLSEEIHWRYNITVYCIGATQLRENPLYKTIYVHWLYFFVYYAFPFVALVIFNVAIYKRVRKANRDLRRLSRHQRREIGLATMLLCVVIVFLVCNVLPLVSNVYENMYVNPPGWMIQMGNLLVTFNSGVNFVIYVIFGRKFKRIFLKIFCRVSGETGLDCMGRPSRADSPDFQTNEDSIATNLSNVELRNSIRRFHGSHHHHHHGSHHGSHHGSNSLRPGGGSTHSGASNGFSRSSVYYPTSPKPGFHNNGSNHSQHNNHQHHHRDRSTNLEDTTFC

>NV17617-RA SP/AstB

MSNASAGYNFTCGSGVDSFHTSYVAIHGWASLLVCIFGSIANGLNIAVLTRREMSSPTNAILTGLAVADMLVMIEYIPYAVHSYLYHRPKRETYTYAWTVFVLFHSNFAQVFHTISIWLTVTLAVWRYIAVAHPQKNREWCSYNRTIMAIVAAYVICPLICFPLYVTTEVTSKNVTLDANDRQINLTGNGSSHYYSGRSGADANNATLYFVALTESAQNGLKEMNFWMYSVVIKLIPCLALTILSLRLIMALVEAKKRRKKLTSTTMLKMEESINLAESKKRKRKASRMMDKEKQTDRTTKMLLAVLLLFLLTEFPQGTLGLLSVVLGPDFFNTCYVKLGEAMDILALINSAINFILYCAMSRQFRTTFNQLFCRWKLLGRWLPVPQHAENNNGNMATNHTVTQVTQV

>NV16129-RA SP/AstB

MNEAALQLGQHQQPLAHQLSNYTELLRRLNITEEDLDYVNNFGSSAGIGGTGGTGGTGGGCSCGPCHCGSLVRRFAASYRAYHGYVALLVCGFGTLANLLNVAVLTRKELRRAPINRILTGLAAADVLVMLEYVPFAIYEYIVLPERRHFPYGWAVFVLFHMHFSQLLHTISIALTLSLAVWRYIAVRFPQCSRSWCTPARCRLALLCSLLAAGLACAPSYFVFGIREQKLMEENGPVVLYHVDASRGPSSDDDRGLLYRLNFWLLGVLVKLLPCFVLTVISCRLIQALYKAKTRRRLLRPLDGQLTDTPATGGRSERRADRTTRMLVAVLLLFLITEIPQGVLGLLSALLGDCFFRSCYHSLGEIMDILALFNGAVNFILYCSMSRQFRTTFGRLFKPSIVVGKWQPACTHQTDIQSTYV

>BmMSR

MSEDESYGFCSESATAFQRVYIQAHGYIALIICLLGSAANSVNIAVLSRKEMTSCTNSILTGLAVADLLVMIDYIPLALHLYTKIGSELNQNSYGWAVFIYFHSIFSQTFHTISIWLTIMLAVWRYIAIKFPQKNQTLCNKRNTTLAIILAYAVCPVLCLPIYFAMNIKERLPSDTSNGTNVTLNYTSAANVSDPKQFIIEMTNNNDLLTAIFWIYSVFIKLIPCVVLSILSVLLIMKMKSSDRRRQKLLKKSAITTTEGEKARLNDDGKKGGGRTDRTTRMLVALLGLFLATELPQALFGLLTAIAPHLFLICYYAFGEVMDLMALVGSAVNFVLYCSMSRQFRTTFTRLARKVLPLPQRQREREPLTTVTVS

>BmA3

MTIEAAPNITQTLTSLFEEMLLETEASNHNESNLDLSHFLSALRVQSNSSNHEPLDFGTLIRLVDGFRNKLNLSRPIEPACTYCDGNIRDVILAYNSIHGYISLIVCFFGSLANTLNVAVLTRRDLAAAPINRLLKWLAVADVFVMLEYVPFAIYRYLILPGQREMPYKWAAYLLFHMHFTQIFHTASICLTLSLAVWRYVAIKYSDKNHILCTERRCSTAILSSFIIPPVLCIPTYMVFDIHTAVVLEPTGPMILYHVDSDEEGGLYQINFWVHAVLIKLLPCCLLTVISLWLIREVYSANQHQKKIRVYNACPSNNKAIKRQYKADRRTNRTTKMLVAVLLLFLVTELPQGILGLLSGILGRCFFKRCYDLFGELMDALALLNGAINFVLYCSMSRQFRMTFGQMMWRAHLHRWSPPQASHSDGQTTAKSSVP

>BmA8

MSNKPQSLLDIFQEIFAASGNSSDSYNETQILSMLREQTNRGGAGEEFESLLVKMMKDAKSKLNLTLKPDTCGYCEGDFRDVITVYNSIHGYVSLLVCTIGVLANSMNIAVLTRRDMAAAPINRLLKWLAVADVFVMIEYMPFVIYRHLVLPEKLDFPYSWAMYLLFHMHFAQILHTASICLTLSLAIWRYIAIKYSDRSHILCTERRCSIAILTSFILPPILCTPTFMVFDIHTKNVTNADGNPDIAYHVDSDYQGTLYQANFWVHGVVIKLLPCSILTVISIWLIKALYKANQHQKNLRNYSACPAAEKMVKRQHKADKRTDRTTKMLLAVLLLFLVTELPQGILGLMSGLLGWCFFKRCYDLFGELMDFLALLNGAINFILYCTMSRQFRQTFRQMLLQPPLARFLPPTASHSESHNQNTCTEKIKSMKTSIP

>BmA13

MANDTDAYCVPGATNFNKAYSRLHGYIALVICIVGSATNSINIAVLSRREMSSSTNSILTGLAVADLLVMLEYIPYALHMNIKIGPQVNKNTYAWTVFVYFHSIFSQTFHTISIWLAVTLAVWRYVAIAFPQRNRTWCNRKNTITAIVSAYVICPFLCLPIYFAMTIVPSEVTSKNNSEFAEDLALPQNRTVYLLEMSKNVELVTAIMWIYSVILKLVPSIALSILSTCLISKLTTTERRRQNLLKRSTVGPNEPEKQCLADDSNTRRSSRTNRTTRMLLAVLGLFLSTEVPQGLLGLASAVAPDFFKNCYSMFGDLMDMLALFTSSVNFVLYCSMSRQFRCTFARLARRMLYAAEEPAKFAAKLEPTTQVTGPL

>BmA20

MLSENETRSSYDTQTNLSHISGNLFEDASINSLLNHGGNYTTNIGQVLKVLEDWRNRFNITVVKECDGTEYCAGEFRDLIIAYNSIHGYVSLLVCLFGSLANAFNVAVLTRRDLAVAPINRLLKWLAVADVFVMIEYVPFAIYRYLLLPGQEDRPYSWAAYMLFHMHFTQIFHTASILLTLSLAVWRYLAIKYPAHSPILCTDRRCTVAIMLSFVLPSILCIPSYFVFTIHKDFSYDRNANVFSKVYFVDSDFDGYLYHINFWVHAVLIKLLPCVILTIISAWLIRALYRANYRKKILKGYNACPAETIVNGKGNIFTRRSTKRSKIERRTDRTTKMLVAVLLLFLLTEFPQGILGLLSGILGRCFFKHCYNLFGELMDALALLNGAINFVLYCSMSRQFRTTFGQMLRTRCAKTYRAGSQTELQTTYV

>Dm_CG13802 MS

MVTNMSQPHYCGTGIDDFHTNYKYFHGYFSLIVCILGTIANTLNIIVLTRREMRSPTNAILTGLAVADLAVMLEYIPYTVHDYILSVRLPREEQLSYSWACFIKFHSVFPQVLHTISIWLTVTLAVWRYIAVSYPQRNRIWCGMRTTLITIATAYVVCVLVVSPWLYLVTAIAKFLETLDANGKTIASVPLSQYILDYNRQDEVTMQVMSSTTPDVSWAIPSDSANGTAVSLLSLTTVIPLTTLSTGVTTSSSLGERNVTVYKLYHSALALRDRQFRNATFLIYSVLIKLIPCFALTILSVRLIGALLEAKRRRKILACHAANDMQPIVNGKVVIPTQPKSCKLLEKEKQTDRTTRMLLAVLLLFLVTEFPQGIMGLLNVLLGDAFFLQCYLKLSDLMDILALINSSINFILYCSMSRQFRSTFALLFRPRWLDKWLPLSQHDGEGRVGGSGGLGGYGGYGRQRLLHTDAVSKSMAIDLGLTTQVTNVXQESSGRAAMSAAAGGAAASVALALAATDVDGCPPATDAAVSTNDISLVEKLHLQPSPRGTAISSGQHRRRRSGSGTKCIWPTTDWLRKLRNQKARETEQSSEQDIELGKSSINRRSSVLLMVLLSSSDEVKAKAVLVSEQPPSPADEDVEDAIDALWL

>Dm_CG8985 MS

MASGNNETEPLYCGSGMDNFHTSYKNMHGYVSLVVCILGTIANTLNIIVLTRREMRSPTNAILTGLAVADLAVMLEYIPYTIHDYILTDSLPREEKLSYSWACFIKFHSIFAQVLHTISIWLTVTLAVWRYIAVGYPQKNRVWCGMRTTIITITTAYVVCVLVVSPSLYLITAITEYVDQLDMNGKVINSIPMTQYVIDYRNELLSARTAALNATPTSAPLNETVWLNASTLLTSTTTAAPPTPSPVVRNVTVYRLYHSDLALHNASLQNATFLIYSVVIKLIPCIALTILSVRLILALLEAKRRRKKLTSKPATPGASNGTKSPANGKAADRPRKNSKTLEKEKQTDRTTRMLLAVLLLFLITEFPQGIMGLLNAVLGDVFYLQCYLRLSDLMDILALINSSINFILYCSMSKQFRTTFTLLFRPKFLDKWLPVAQDEMAAARAERSAVAPVLEKGRQQPQVVMASTTTNITQVTNLXHRRSRGRRTLLSRLLSVLKRGRRRSSGEGGGVGGGGAPLAGNDAVEPAFQAIVVVVDKVSGATENQLYTAEQARIVT

>BmA18 CNMa

MCPLPANETDYYLESSEYFNATSSYISAVNETFFDIATENIATLLNVYYTPLLVALGCIGNLLSVFVFYRTKLRLQSTSQYLTALALSDTVFLFQLIPPWLNAVEVTGLFYKHGFCQIFVYVTYVSCCMSSWLVVAFTVERFVAVLYPLRRNALCTVTRARHIIFTVLLASSLINLPVLRFAVPSKNDCNIDFEYLDQAARFNLVDTALSFSVPLFLITILNTWIMIGVWKLERQRRQIMKEEMERARARPYRTTGCPRSQHRVTRMLLIVSSVFVVLNLPAYTMRILAYAYNMNEEEYSGRWAAVQQLSLLFFNTNFGINFMLYCLSGQNFRRAVQQTLPCLRKRADHRAARRATQTRPGSGSSK

>DmCG16726 CNMa

MDMEYITSSSGNITATTEADFSSSLGESNVTEYNTTEMDANESAGEDEEMLRIAFFIGHFVHQYYIPVLCCTGSIGNILSVFVFFRTKLRKLSSSFYLAALAVSDTCFLAGLFAQWLNFLNVDIYNQNYFCQFFTFFSYLASFCSVWFVVAFTVERFIAVIYPLKRQTMCTVRRAKIVLFCLTLVGCLHCLPYIVIAKPVFMPKLNTTICDLNSEYKEQLALFNYWDTIVVYAVPFTTIAVLNTCTGCTVWKFATVRRTLTMHKMKPQTNSMPSNSSNSSGGASSAVASYRLSASLKRQKSTGTHPSGQHNVANRQTDDQEQQQQSQQHQINNCQHHCEITQKPARRKVQNSSQLKVTKMLLIVSTVFVCLNLPSCLLRIEAYWETESARNQNSTIALQYIFHAFFITNFGINFVLYCVSGQNFRKAVLSIFRRVSSAQREAGNTQVTVSEYCRNTGTSTRRRMMTQHCWNEMHELHPLK

>DmCG6986 Pro

MTMSSTSTATATSTATATLDEANATVGEMFSDADMAEVRHVVQRILVPCVFVIGLLGNSVSIYVLTRKRMRCTTNIYLTALAITDIAYLTCQLILSLQHYDYPKYHFKLYWQLYGYFVWLCDSFGYISIYIAVCFTIERFIAIRYPLKRQTFCTESLAKKVIAAVAIFCLLSTLSTAFEHTITIGTRQIDDAYQPCNQTVANISPMPPPPVAVTPPLATPPLPTPATIWQSPDSAMESTTSGSSNQLVDWGSGSGDGEPENIPRHRRHWQSSGFVTLPTLRKTLEEQDQKVADAAQRSGVTESLLQLWRRKRSAENHNINNTDAFAFNVTEYCQNVTYYNHGLSELGYDELYSYLWNLFTLLVFVVFPLLLLATFNSILILLVHRSKNLRGDLTNASSIRRTKRKSNSGLKGSVSQENRVTITLIAVVLMFIVCQLPWAIYLIVNQYMEIQIGTQVVAGNVCNLLASLHAASNFFLYCVLSDKYRKTVRELITGYRYRRRHARNNTSLYVPHTTTTLTQINGDHYGSNYGGAGSRRNRNTGRLIA

>BmRFaR/FMRF

MNGTEGEGCDSDVGVQPADKLFRFVVHGVLLNAIGAAGLLGNALSVVVLSRPQMRSSINCLLVGLAACDTVLILTSVLLFGLTAVYPYTGRLRYYYYHVCPHITPYAYPIANAAQTMSVYLTLIVTVERWVAVCHPFRAKSLCTSSRARWYVLGTAAFALAYNAPKFLEAEVVTRSVDGEPVYCVTADLHFRTETYIVVYIHSLYMIVMYIVPFSALAALNACIVRQVRRAQAERARLSR

VQRRELGLATMLLVVVLVFFLCNLLPLVTNSFEVFLGDQLENLDPLVKTSNLLVTINSSVNFVIYVIFGEKFKRVFLKMFCAGGWRRRTRDSPEQTRDDSFASCGERVSLRLVRNGTLRRSEPRAPPRGRSRRAPSPSVYYPAPLTDVTSALSVSEPPPAAMRWNGHTRSHF

>DmCG2114 FMRF

MSGTAVARLLLRLELPSPGVMPPPPTDYDYGGPISDDEFLASAMATEGPTVRYDLFPQNNSQPTLQIVLNHTEVQTDLQYPHYEDLGLDPDPNWTRICEDVYNPLLENNRIEFWVCGVLINIVGVLGILGNIISMIILSRPQMRSSINYLLTGLARCDTVLIITSILLFGIPSIYPYTGHFFGYYNYVYPFISPAVFPIGMIAQTASIYMTFTVTLERYVAVCHPLKARALCTYGRAKIYFIVCVCFSLAYNMPRFWEVLTVTYPEPGKDVILHCVRPSRLRRSETYINIYIHWCYLIVNYIIPFLTLAILNCLIYRQVKRANRERQRLSRSEKREIGLATMLLCVVIVFFMLNFLPLVLNISEAFYSTIDHKITKISNLLITINSSVNFLIYIIFGEKFKRIFLLIFFKRRLSRDQPDLIHYESSISNNGDGTLNHRSSGRFSRHGTQRSTTTTYLVATGGPGGGGCGGGGGNNSLNNVRLTQVSGSPGLVKIKRNRAPSPGPVVYFPAREMQRSASTTNSTTNNNTSIGYDWTLPDSKKLGHVSSGF

>BmSPR SP/AstB

MAVTIDNSTNDFEFQKPFNYSINENITYFDYTNFTSDDFCASNNSHVYLNVTCEFAISYAEPMYGYIAPFLLATTTVANTLIVVVLSRRHMRTPTNAVLMAMALCDMFTMLFPAPWLFYMYTFGNHYKPLSPVRACQAWNYMNEVIPAMFHTASIWLTLALAVQRYIYVCHAPVARTWCTMPRVMKCLIYIGIAAFLHQLPRFFDRCYTPHKTVWRGRVEEVCRIEMASWVKALSVDAYF

ISYFGFRVLFVHLIPCTSLVVLNVLLFRAMRTAQINRQKLFKENRKSECKRLRDSNCTTLMLIVVVTVFLLVEIPVAVVTILHIISSTIVEILDYHIANILVLVTNFFIIVSYPINFAIYCGMSRQFRETFKELFIRGTVTSRKNGGSSRYSLVNGPRTCTNETVL

>Dm_CG16752 SP/AstB

MDNYTDVLYQYRLAPSASPEMEMELADPRQMVRGFHLPTNESQLEIPDYGNESLDYPNYQQMVGGPCRMEDNNISYWNLTCDSPLEYAMPLYGYCMPFLLIITIISNSLIVLVLSKKSMATPTNFVLMGMAICDMLTVIFPAPGLWYMYTFGNHYKPLHPVSMCLAYSIFNEIMPAMCHTISVWLTLALAVQRYIYVCHAPMARTWCTMPRVRRCTAYIALLAFLHQLPRFFDRTYMPLV

IEWNGSPTEVCHLETSMWVHDYIGVDLYYTSYYLFRVLFVHLLPCIILVTLNILLFAAMRQAQERRKLLFRENRKKECKKLRETNCTTLMLIVVVSVFLLAEIPIAVVTAMHIVSSLIIEFLDYGLANICIMLTNFFLVFSYPINFGIYCGMSRQFRETFKEIFLGRLMAKKDSSTKYSIVNGARTCTNTNETVL

>CG13229 Orphan

MMQETGNQMGQTHMHQRVPFNDTVLKDYHLTSTDIEKFVKLWQEYQMKNMTPQVDECQGYCQGEIYNWLRAYNSIHGYVSLMICIFGTIANILNIMVLTRKEMAKTPINNILKWLAVADMFVMLEYIPYTSYQYIYMGPGEKDLSYTWAVCLLVHMHFTQILHTISIGLTVTLAVWRYVAIRHPNGGCANFLLAHSREAILLPFILSPILCLPTYFVFQVRETYDVDKVNSEAMYHVYFDKDSVLYRFNFWIHSVLIKLLPCGILIVISAVLMHVLCEASRRRLKLRDYNNPAKYAIQLNLNETKSKKPPRCDRRNDRTTLLLVAVLVLFLITEFPQGLLGLLSGVMEKCFFAHCYPPFGELMDLLALINAAVGFVLYGLMSKQFRTTFRSLFMKRHFGSTEMTRLTRVTTTCV

>CG33639

MITRLYNTEEDPAYCSFIWGSNLTSSVDVLAANATSVFSSDLRDDFYRDVEDPRTESLREYCYGLVLPIICAMGIIGNVLNLVVLTRRNMRGTAYIYMRAYSTAALLAIVFAIPFGIRMLVHKDRGQWEEFGPAFYTAHLELYLGNGCLGVGVMMLLVLTIERYVSVCHPGFARPVMGPPGVVVFLTCLATVIVYLPSIFRGELIKCILGSSDVYVYLRRDNTIYQQTIFYRVYKIMLEVIFKLVPTLVIGGLNMRIMMVYRRTCERRRKMVLSRPHAQGHGHGHGHGHGHGHGHAHGHGYLKDDDPRKFAEERRLFLLLGSTSILFLVCVSPMAILHMTIASEVYPSFPFQVFRASANLLELINYSLTFYIYCLFSEDFRNTLVRTIKWPWLKGKFCHQAEHENPTNGPGVPMACFTKVDRGHQKHHITTTSGLGRSSSI

>RPRC005858+RPRC001892 Serotonin

MEWENTSNILWNITTILGNNSNITADDLERWSKHLQPTTAVTTIQQPTQQHSSHRDWTFLFVLLFVAAGGLGNILVCLAVCLDRRLQNVTNYFLLSLAVADLLVSLFVMPLGAIPGFLGKQICKCFYLPQQYKDLEDGIVSLTGFGVSSDIMNGYNKKFRPVLTPGQSILLETHNFDFNTFVVVSRIMDSTAIQNIIFHDTRFKLITLGTFYNNMAANVVATEQKASKVLGLVFFTFVLCWSPFFVLNIIFAACPSCPVPTHVVDVCLWLGYVSSTINPIIYTIFNRTFRAAFIRLLMCKCSRWNRLARYRSVNEHRTGGGVNTPSTQTGMGSAVPLSLSLQGTPILSPGTASTYLRTPSTFPDSFTVGEQQT

>RPRC010931 Serotonin

SLLQISQGWILGPELCDMWTSSDVLCCTASILHLVAIAVDRYWAVTNVDYIHTRNRGRIVGMIVVVWSVALVVSLAPQFGWKDPDYLDRITIQQRCLVSQDVGYQIFATCSTFYVPLLVILVLYWKIFQTARKRIRRRRTKQHTAQMKIDGKQKSSGPFKFLSKKKLSLICVNWFRLVQEWETKRTGEVALVEGNVETDKEGSDAGATTAFTISTNHGHTPSNVSPERSTAELTANSKPPPPLQPPSTPVVQTKPKRDAKKESIEAKRERKAAKTLAIITGAFVVCWLPFFIMALLMPLCHTCYINENLASLFLWLGYFNSTLNPVIYTIFSPDFRQAFKRILCGVSHSRPRNLR

>RPRC002007 Orphan

MTGSLSDDKMAVSSAKVKVAVFGCSGRYLLNGETSRRFLFLIESRQLPTFSSISTVLVSFELANALILASLRWIRRPLSPTLHISLSLAGADMFTSLVIGIGLIVNSLLPQVFSIQIDKCSQLVIEALRMGGMYTSIGHLLTLAVNHYLGIKKPLHYPSLMTTRNITVIVLALWIIPPSSFAIYFSLLEQDGFAIIGCDYE

>TINF_H9TUR5Q02IXHQI_1 Orphan

FFVSQVLLVMNEKMGDYEDNTLKLHYYKIQNYSEKNNYSNEHLNFIIYLFHCCFCVFSVLVNALILASLRWVRRPLSPTLHISLSLAAADMFASLVIGVGLVVNSLLPQVFRIQIDKCSQLVIEALRMGGMYSSLGHLLTLAVNHYLGIRKPLHYPSIMTTRNITFILMTLWIIPPASFAIYFSLLEQDGFAIIGCDYKVPNCL

>TDIM_IAZY42G01B8KLV_2 CAPA

AVVSTQSGHYGRGEISSEVVIYVCLSYLFKKIYFNKRKTCILKIFQIEQIISLWRNIIYVGQNIYHLKILLPITITYSVIFISGLLGNLAVCIVIAYNKTMHNATNYYLFSLAMSDLVLLLLGLPNDLSVFWQPYPWVFGILVCKIRALVSEMSSHVSVLTIVAFSIERYIAICHPLQSYTTDKLNRVIRVIAALWLISFMFLQYHLQFIQLLTTLISTRFWKEVIDSAFCVHAGLTCPPX

>RPRC000057+RPRC004783 ACP

MDPLFSNFTIEFNYTESIYYSPTNNTLYELPKFDDNALIVVIAYSLLFIIAAIGNLTVFITLVRGRHRKSRISLMITHLAAADLFVTFIMIPLEIGWRLTTQWVAGNIACKLFLFLRAFGLYLSSNVLVCVSVDRYFAILHPLRVSDARRRGKMMLTMAWIFSLICALPQMSIACYGAQVCSGVCKMRLQTDQWRTGHEERTRGRMRLRRSDMSNIERARARTLRMTVTIVLAFIWCWTPYVVMTLWYMFDRESAEKVDPRLQDALFIMAVSNSCMNPLVYGSYALNFRRECTTCFCYLFSSHQQLDRRSTDAAAHTSRVLRVPKCVLTFVRGSGITRSTAVTGYGGTLGSRNHLTVPRKNVMRPASAEHLVIRGRMIETAPLNPEEFHSDPGTNTGIYLVTS

>RPRC007712 AKH

MTVTEVLRVGLRDLDLGALVNSSFREPAGDFMRKVWSASWSVCNFRHLPHWLQDNDYLHDGHRPPLPSFKMCFKSIFRIHTETGNIWTHLLGCVAFIAVLLVFMTRPSEELPLSDKIAIGTFFVGAITCLGLSFVFHTVYCHSEFVGKLFSKLDYCGIAILITGSFVPWLYYGFYCQIRPRIIYLTVVIVLGIASIVVSLWDKFSESRFRPLRAGVFAVFGLSGVIPAVHYALAEGWLNALTNASLGWLILMGSLYLLGAFLYAFRVPECLYPGKFDIWFQSHQIFHVLVIAAAFVHLHGITEMLTYRMSIGACAVQSAALVL

>RPRC004706 AstA

MSGSPASVIVGAIGSMPPIKYDPHNNFTNNTINFNNNIHNFNNNNIDMRNFYSNVTDTEIFMEEISPELTEKIVAIVVPVLFGIIVILGLFGNALVVIVVAVNQQMRSTTNILIINLAIADLLFIVFCVPFTATDYIFTFWPFGDTWCKMVQYLIVVTAYASVYTLVLMSLDRFLAVVHPIASMLIRTEKNAITAILVTWIVIVISNIPVFLCHGEVTFNYSSSEHTVCIFLEMDQLIRPDGFNKVAFQAALKWLHKIINADDQAT

>RPRC013486 AstC

DTTVSWLADSLENGSIESLYNSSYGNETQFCGSTDQPTLHIFTQVLYAFVCIVGLLGNTLVIYVVLRFSKMQTVTNLYIVNLAVADECFLIGIPFLIATMSLQLWPFGNVMCKLYMASTSINQFTSSIFLTIMSADRYVAVCHPITAPKMRTPFISKIVSLSAWTASAIFMIPIFMYANIMDDDQVKSCNILWPEGENLSGQTAFTLYSFVLGFAVPVVLIFCFYFMVIRKLQTVGPKNKSKEKKKSHRKVTKLVLTVITVYVLCWLPYWITQMALIFTPPKQCQSKFTVTVFLFAGFFSYSNSAMNPILYAFLSDNFKKSFVKACTCAAGKEVNATLHLENSVFPRRTQRGGSERARAGKNRADHTDEGAETGPLVSRGEHSTTALTSRSNITVTSDTTTPVKNGVKINLTPTEL

>TPAL_H9TUR5Q02IYTLQ_5 AT

TPSISSSVCRRTDWQLLSLSCSSSSGHENCHQLFIVNLAVADFLVILICLPPTLVWDTTETWFLGHVLCKLVLYLQTVSVAVSVLTLTFISLDRWYAICFPLKLKSTTSRAKTAILIIWIISLLYDIPELITLRTARRKKFHVETILFTQCIASWDDVAERHYTTSKIVFLYLLPLTITSAAYFQIVKVLWKSDNIPGHRYQRDVCFISGSSVDSRRYIYYVX

>RPRC007766 CCHa1

MEYKDQLNNDSLVNTTENAITVIPYSERPETYIVPIVFAVIFLVGVLGNGTLVLIFIRHRTMRNVPNTYILSLALGDLLVIISCVPFTSTIYTVNSWPYGLFICKLSEATKDVSIGVTVFTLTALSADRFFAIVDPMRKLYSSIGGRGATRCTIMIACAIWLLAIACAIPGALFSYIRIFKQGNHTLFEICYPYPEELGSVYPRGLVMAKFLIYYAIPLTVIGCFYILMARHLVLSTKNMPGELQGQARQVRARKKVAKTVLAFVLVFAVCFLPQHVFLLWFYNNPNSDRDYNEFWHVFKIVGYCLSFINSCINPIALYCVSGTFRKHFDR

>RPRC000608 CCHa2

MDPETVEDFKYVSSVNSNISNVEEYTPYPERPATYIVPIVFAVMFLVGVLGNGTLVLIFIRHRTMRNVPNTYILSLALGDLLVIISCVPFTSTLYTIESWPYGGFVCKLCEATKEISIGVSVFTLTVLSAERYCAIVNPIRRHISTKPLTIVTVFCIWVISFLLALPAAIFTHVSKANITNGRTIEFCSPFPEEYGPTYRKLNVLLRFIIYYAGPLLIIAWFYILMARHLLLSTKNMPGELQGQSNQIRARKKVAKVVLVFVIIFIICFLPHHFFMLWFHFNPDSDEEYNLFWHVLRIVGFCLSYLNSCINPIALYCISKAFRKHFNRYLLCSFVRDSTLDEISLGNMNSSTKHVRQSSIITSHYTITQSEKT

>RPRC001248 CCAP

MDWVIRDNYSNPAANITNTTDEINSFYFYQTEQFTVLWLLFAAIVLGNSAVLLALLFNKSSKSRMNFFIMHLAFADLSVGLISVLTDIIWRITVEWKAGNVVCKVVRFMQAVVTYSSTYVLVALSLDRLDAITRPMNFSGSWRRARLLVGFSWTLSAFFSSPILILYEERLIQGSFQCWIELGSTLKWQIYMSLVAVSLFLVPALVITACYTVIVYTIWTKSIHISRDSQQSTPLKNGGDKGDDNDIRRASSRGIIPRAKIKTVKMTFVIVFVFILCWSPYIVFDLLQVFGYVPRTQTNIAVATFIQSLAPLNSAANPVIYCLFSTHICRALSKLPPFSWICCCFANRGAESSSIIDTVTSTLRRATIRNQDNL-

>RPRC000969+RPRC012063 CCAP

LIIFFRKIDELIEVKENHVRGLQSWLGQTEQFAVLWLLFLLIVCGNSAVLAALKCAKKPKSRMNFFITQLALADLCVGVLSVLTDIIWRSTIAWNAGNIACKVIRFSQMAGKTLVFIVCWSPYFIFDLLQVYGYVPTTQTNIAVASFVQSLAPLNSAANPLIYCLFSTRICRGIRVSTPQKIGKGITE-

>TDIM_isotig12092_6 CCAP

ICTKMANLHESCCSYIVSSTCIGNNCLLYCDCLYYMDEHSYLKRQPAINTIKKWGQRRKYPKSKFKRNHPKSIKTVKMTFVIVFVFILCWSPYIVFDLLQVFGYVPRTQTNIAVATFIQSLAPLNSAANPVIYCLFSTHICRALSKLPPFSWICCCFASSGTESSSIIDTVTSTLRRATIRNQDNX

>TINF_IAZY42G02HL8UN_4 CCAP

SGINAEWAVCGNRLGNVPVPAKYSLRTLLKVRSHRVGRRNGLVGILILYNTKNISWHVSIRLRSCGTVKWAETLNSEYPCTQTLLKTALGRFNGANPISEMLLIFLRNYKLLFCFIQIICNFTAGQWRTGYEERTRGRMRLRRSDMSNIERARARTLRMTVTIVLAFIWCWTPYVVMTLWDHFFLFSLFKYPLYLLMGYLNKRK

>RPRC000523 Crz

MQTLFPNISDETTLRQLQDHLINSDDGHRFILPLELCDLWNITVSSSSRIQCLEHAPQLTSSARTRAIVLGVMAVISFIGNVLTIISIRSSRRRRRNQNWSAVYALILHLSVSDLLVTIFCIAGEALWSYTVAWTADNVTCKLFKFSEMFALYLSTFILVLIGLDRFVAVRYPIKAISTAKRCGRFVAGAWFLSFLLSLPQVFIFHLSKGPFYEEFYQCVTYGFYTEPWQEQLYTTFSFVCMFMLPLLILIISYVSTIITISRNDKMFRDESNNTSATRKLDINRRRLIHRAKMKSFRISLVIVVTFIVWWTPYYTMMIIFMFLNPDKHLSEELQKGIFFFGMSNSLVNPLIYGAFHLWRPSKKTGSARSVSVYFLLLLIFFL

>TINF_IAZY42G02H0ZZ5_5 Crz

SSIQCLIFLVTIFCIAGEAIWSYTVAWTADNVTCKLFKFSEMFSLYLSTFILVLIGLDRFVAVRYPIKAISTAKRCGRFVAGAWFLSFILSLPQVSIFFFTLPFIEMNYYRIQCFLYINRRVINSISYVNTGKTSLFLLVTRIYVHMSSSYSQLYEKFYYPIVELLYRNITIYSRNQTRPRGRNGHSALIPLX

>RPRC001551 FaLP

MNFTNTNFSLDDNETAYISTDNENDDKSEILFEFITNGVLLNLVGILGIMGNIISMVILSRPQMRSSINYLLTGLARSDTVLIITSILIFGLPALFKYTNSQLLFSYYYRVYPFLAPVVYPLAVIAQTVSVYLTLTVTLERFVAVCHPLQARSLCTYGRARLYVLLIIIFSILYNLSRFWEVKLEQEYLVQYNVTVYIPLPSSLRSNQIYISVYIHWLYLLFIYFLPFSCLAVLNAAIYRQVRKANQERQRLSRLQKKEIGLATMLLCVVVVFFICNILALVSNVLEAFYGILLTKMVKTSNLLVTINSSVNFIIYVIYGEKFKRLFLKLFCSHSPRICDGGVRESPDCATLHEDSVMLSNGDARHSVRGNRANESVKRAARALPCVYYPARHNSKWNQDDTTTTTLNQI-

>RPRC015267 Pro

MGNVVTVIVMTRKRMKSSTNTYLTALAVSDLLFLIFNMILSFEHQPAIRQSQYVTYWHLHKWTIWLVDATGACSNWLTVSFTLERYIAVKHPLRGKVLCTESRARKVIXTCNITSLPLNNYYSIMECRRDRRVALQSTWLGQHPTYKSVFYWFSSITVTAIPLASLSVLNYLLVAAVRRSTKGRNQLTEDARGVRGSFRPTCNAGNSIYSRCGSERSPPIQGMSQRRVLKERQENKVTIVLISVVFLFLICQAPSAITVIVKVFYEPESDTSGDYLLRSAGNICNFLMVINAASNFFLYCALSDTYQRTLTTTFCRRERRWNERNDTLSTAASFRNSSVKQLRHENETQ

>TDIM_H9TUR5Q02GDFPZ_1 Pro

SGINAEWPLRRGPAMLASILISLGLDIFDISIRDGNKMVKCPLISSFINNKIYFCLIILMYFISINIMFCFTFTLMNKLKNIVVLGACPNWLTVSFTLERYIAVKHPLRGKILCTESRARKSYSVCYIVGLLLNNYNSVVECRRSRRQVRNIINMKP

>RPRC000494 LKs

MGSRVSWGIFCSYELNKKIMNCSFLEDELGPLPPSANCSWLLHNQSVYFYEERHERAILPSTSIKEKECIPHQSLYEVPAGVIVLLSVFYGTISVVAVGGNFLVMWIVATSRRMQNVTNCFIANLALADIVIGLFAIPFQFQAALLQRWNLPNFMCPFCPFVQVLSVNVSVFTLTAIAVDRHRAVLNPLSAPPSKLRAKALLGAIWILAAILATPMAVALNVTYVEENDHVGHVYTKPFCINTKLSNNHMMAYRMILVSVQYLTPLCVISYAYAKMALRLWGSRAPGNAQHSRDANLMRNKKKVIKMLVIVVALFAICWLPLQTYNVLQDIFPQINGYRYINIIWFCCDWLAMSNSCYNPFIYGIYNEKFKQEFQQRCPFSRRRKWTHGFGAGGSDSLDLDKTIHRFGSVNRNSSRWIRYSSRVQYTPAQHYIYHCANSNTVHHSSQSEIEELCLYVLKVMKRLEISITGQNTIANYSTYCRVQDPCLNY

>GL563029 NPF

MVCRLVGTFRNKIELCVIMELNDTFNFSLNEVYRILIEHKNDDHNVDPVAEAILIALYALLIVVGILANLIVSFVVARRPQMHTARNLYIVNLTVSDMTLCLVCMPFTLVNILRRAWTLGIVLCKLVPALQGTNIMVSIGTITVIALDRYFTIVRGQDSATTRRRVIISIALVWFFSFLATLPVVEPFKFEAVILYETCIERWPSQELKVAYAVCVLMIQAVIPALVVGCIHAKIASYLNAHAKTQRDSKRAQRELQRNKRTTLLLSGAVAVLFAVSWLPLGLFSLMADLLYPPGSETHISSQSLYITLAACHLLAMSSAISNPVVYGWLNSNIRRELVQLLPSRCTSRQQQSQQQTTNAPSPTIMLCQNGQNIPHQQPATTYTAL-

>RPRC008364 NPF

MGKGLKEIHKYKGDIPLHVSTLTLLLIAWDRHRFLKDPMKPRIPAFVCATGSWLTAICLVLPYPVYTTYMDLGFSNFSPPMLYLYFWRSYGQKPDWGFMRTTGFDNDYENYIKNCEK

>TPAL_H9TUR5Q01BIIZA_2 PBAN

HVVAVVSTQSGHYGRGRYPYVFGEAFCLLRGLAAETSANATVLTITAFTIERYVAICHPFLAHTMSKLSRAIRFILAIWVIALAFAIPQALQFGVIYGNEPFQILCDVKEILIVHSFEVSTLLFFIGPMTLITVLYALIGLRLRRSALLTRNSGSFGHGDSGRKGANSCRHHSSQRVLKMLVAVVVAFFICWAPFHIQRLVAIYIKEMNFISGTTPIX

>RPRC001000 RFa

LILVCIPVKLAKLFSFTWTMGVFLCKMMHYMQSVSAICSVFTLTAMSVERYYAIVHPMKAKYVCTISQARKIIFTTWVASFFLAVPILFVQVQMPVGGRIKAYWCVRDWDWVVAWRCHEVYMLVLVLLLPASVMTVTYSAICREIFRVMQRRFHMTSGKATMNCESFPLSKPKKKRPPR

>RPRC002266+RPRC002268+RPRC002269 sNPF

MNNTTIEEDLSMIVDCIVTQYNISQYKGQWPNCTEIGFRKDIIDDKIVQAIFCLLYTSIFVLGLFGNILVCYVVGRNRAMHTVTNCFITNLALSDILLCTLAVPFTPLYSFLGCRWIFGNALCHLVVYAQSTSVYISTLTLTSIAVDRFFVIIYPFKPRMRLSTCLAVIFFIWTFSLIATIPFGLFMDHKSIAGRFYCEEKWPSENFRQVFGGMTSTIQFVLPFIVVTFCYVRVSVKLNDRARSKPGAKTSRKEEVDRERKKRTNRMLIAMVTIFGVSWLPINLINVINDLYMHTSSWTYYNLFFFLSHAVAMSSTCYNPFLYAWLNDNFRKEFKQIS-

>TDIM_H9TUR5Q02INV8X_6 sNPF

VLFFFLVCPSFILFLTFYFKVVWIIRCSVPSCGICPKYVRVYLNINIDINSCGSVFLFIIYPFKPRMRLSTCLAIIFFYLDIFINRNNTVWVVYGSINCWQVLLRTMAIRKITSVWRHDSYITVVVPFIVVSFCYVRVSVKLNDRARSKPGAKTSRKEEADRERKKRTNRMLIAMVTIFGVSWLPINLINVLNDLYMHTSSWTYYYLCFFLTHAVAMSSTCYNPFFIRLAELQKRITSTSMFWRTNWSPVFRKAWLEVRKNV

>RPRC000835 SIFa

MATIVFGSEMANVSSNSMANSILFELGSEFEKYEETMRKSRFPNRANNPQKIDLVCQIRKPGCHAQSIVGMRMNTEEEQPFKEYRRAMLRNYAWQAREQEVVAAGVSHVYNKLMMNSLFPLTSITSDQSGDKKFECFLRAMNGEWGFSLISLNYEHVFLAIWWPLKCQITTRRARLMILVIWVVALTTTIPWALFFDLVVIFTDNPEVKVCSEVWPEYLNGSLYFLIANLLFCYILPMILISMCYVLIWIKVCKRHIPSDSKDAQMERMQQKSKVKVVKMLVVVVILFVLSWLPLYLIFARIKLGGEISGWEEDMLPMATPVAQWQSLVNVRTLCP-

>RPRC013738 SIFa

MTEKTSKMMTIRVALPRPKWVKILIIINQHYYREVLAQLLERVRSVDVGNKNQTVPCSEGNGTTCSTASIYEFRYSFPVTVFFCLAYTSVFLIGVTGNCFVVSVVYRSPRMRSPTNLFIANLACADLLVNVICLPFTLISNVMTDCDK

>RPRC004565 SIFa

MSQPRYLLTSLASNDLAIGVLVTPFGFLPALFKCWPYSETLCQIQVVECLKEINLEDLGTNIIILKKNFVN

>GL545664+GL552047+GL562893 SK

MIHIISFILLQIKMLPTESWWEAGKVQIPTYSIIFLLGLVGNILVILVLVKNKGMRTVTNVFLLNLAVSDILLGVLCMPFTLVGSLLKDFVFGHFMCRLIPYMQGFFSAVSVSVAVWTLVAISLERYFAICRPLKSRRWQTQFHAYKMIAIVWAMSLVWNSPILFVSRLLAMGGKGEGRHKCREVWPGRRSEGAYIIFLDIVLLMIPLLIMSLAYSLIVLKLWKGLQRELKHSNSCLKSFSLLLQVIRMLFVVVAEFFICWAPLHVLNTWYQFRPDLVHQYVGSTGVSLVQLLAYISSCCNPITYCFMNYRFRQAFISLF-

>RPRC003160 TK

MAYTENSTLGWNELTQNITVNETYDDEDGNQFILPWWRQLIWTFLFGGMVIVATGGNLIVIWIVLAHKRMRTVTNYFLVNLAIADAMVSSLNVTFNYTYMVNSDWPFGTLYCKISQFVAVLSICASVFTLMAISVDXXMAIMHPLRPRMGRRMTLCIAVSIWIVGSFFSLPMLIFFTTFVQEFPNGDNRVICYAEWPDGSTNESRQEYLYNVLFMVMTYFIPIASMCFTYVRVGIELWGSQSIGECTQRQLENIKSKRRVVKMMMVVVSIFAVCWLPFHIYFIITSHMPEITKLPYIQDLYLTIYWLAMSNSMYNPIIYCWMNMRFRRGFKQFFSWCPYVHVPPEGLTRREAVTSRYNYSCSGSPEAHYRIVRNG

>RPRC001687 TK

MRTTTNYFLVNLSISDLLMSLFNCIFNFTYMLDSHWPFGAIYCTINNFVANVSVAASVFTLVAITLDRYMAIVRPLKHRMSRRKARIALLIIWAASSLLAIPCLLYSTTKSRRTINGQTSTVCYMMWPDGHYPKSYRYNLIFLVVTYLGPVVAMAICYTLMGRELWGSKSIGEQTQRQLDNIKSKRKVVRMFIIVVTIFIFCWLPYHGYFIYAYHNNSVVGSWYVQHVFLSFYWLAMSNAMVNPIIYYWMNNRRFRVYFRQIICLCCCVRPSMHPELQSTPNNRLVRSELLLRSKSCKPQG

>TINF_IAZY42G02H6C37_1 TK

FVAVLSICASVFTLMAISVDRYMAIMHPLRPRMGRRMTLFIAVSIWIVGSFFSLPMLIFFTTFVQEFPNGDNRVICYAEWPDGSTNESRQEYLYNVLFMVMTYFLPIASMCFTYVRVGIELWGSQSIGECTQRQLENIKSKRRVVKMMMVVVSIFAVCWLPFHIYFIITSHMPEITKLPYIQDLYLTIYWLAMSNSMYNPIIYCWMNMRFRRGFKQFFSWCPYVHVPPEGLTRREAVTSRYNYSCSGSPEAHYRIVRNGVSSRMPRNLNNGPDLLCNLPVNTNNFHQEYLKLNEVHCIVQHVIX

>RPRC015456 Orphan

LTKNVLFVSLLQLSKDIGYVLYSALGSFYIPSCIMVFVYIRIYYAAKARARRGIRKAVARPRPAEKVTSFSKKEPLTSPVDRNSNNSPVAVTVEKPVIPVVTCDFASDISTSDNIEQPDPTAPKDTLNVSKLPTCSLTPNVTFKGSTLSVNGDLAAMSRCRAPSVGIDVDMVSEFDPSSSDSGVVSRCAVVKPLKLRLCKPIFGRKSSKAKREVIDMGRVISTTGSQEIPQEIPKVQKPRDPEREKRRLARKKEKRATLILGLIMGSFIACWLPFFFLYILTAICSACQIPDFAFAVAFWLGYMNSALNPVIYTIFNKDFRRAFRRILFK

>RPRC004128 Orphan

QVRNATAVFIINLSVSDLMSCCFNLPLAASTFWRRSWRHGLLLCRLFPLLRYGLLAVSLFTVLAITINRYVMIGHPTIYPKLYRKQYLGLMVAATWICGFGALIATWLGRWGKFGLDPKIGSCSILPDSSGRSPKEFLFLVAFVIPCICIVVCYARIFYIVRKTALKSRVAGRSAASVTSGGTTLTRSGYYGKVKLVRKGNTSSTDDSAFATSSTAQSFSTEKSSTILDNGEMGGNETVIKMVNLAPSPHLLAPQRRSKICAEASSSSGIEEGLREDDEVVSTRSDSPISACSSSPPPAHYSVKVQKIKKRSERVNSTLSHMASVFRRTSHARGVLSPSRRQSCAPPQPGKMTAKDKKLLKMILVIFASFVTCYLPITLSRHTEI

>RPRC008570 Orphan

MMNLSNGSWPDEEEETLYDPPVSLVVFLSLCYGSISIAAVVGNGLVIWVILTSRRMRNVTNYYIANLALADIVIGLFAIPFEERKLILLDGSYYILKTKKIAGEMTLVKAISAFKCDIWERHNVLKKKCDSNDFLEKFLPPSKVPILGIVRFYHLHKYLIIKRLRNNPTIRVCLNSTKFQAALLQRWVLPHFLCPFCPFIKVLSISVSVLTLSAIALDRYRAIIHPLTMKSRKQIDDRTQSMPIQSVVDVEAISSSTSIISTAPAEDVLTPSLIRSAAFILLRPGTA

>RPRC00203 Orphan

MFNNDWNSSYLLGLDRRQHNGQTYYFTFYSEFGERLAETYIEVGLLVTTLAASVIFNLALVLPLWGSKPRTVTNCFLLNLGLADILFAVGIPAVVTVRINPRWPTFAGELMCKLLPYSQLVCGFTILWSLTLISVERYRCLSLDPNFKISSPQSAHLANIIMWSSAMVLFSPFLFWFRHENDLEICTLLFPKPGINVALMFTILITFFTCILPMTILVFNYQRIFIKMVETRQKWATPCVLTSSLSRSHHFLGALSVALLNTAVNPFLTARLRINSCLRKSMKDKTIFGGSGLFKIGSSIN

>Hheb089310.1 sNPF

MEDPSIMNTEIYDESMFSGNLTVPEDYVTSLIWVQAIFYFLYGLIFVVGIFGNALVCFVVVRNTQMQTVTNLFITNLALSDILLCVLAVPFTPLYTFLGRWVFGKTLCRLVPYAQGVSIYISTLTLSSIAVDRFLVIIYPFHPRMKIKVCLSVILGIWVVALFLTLPYGLYMSLEEPQGRVPLCEEHWPDPTFRQIFSSFTSILQFVIPVLVIGFCYVCVSIRLNDRARHKPGTKTSRREEADRERKRRTNRMLIAMVGIFTICWLPMNILNIVDDFNVDISNWAYFRLCFFITHALAMSSTCYNPFVYAWLNDNFRKEFMHVYNFDYIY

>Hheb077620.1

MVRLLLLCFLAGALGYVDGHGCPAPCICKSVGPQNERLRVKCNKDIQDIKEINVNSVSIELYHLDLSKNSIYIIEPGIFQNLTNLRRLDLSINKITALEEGCFSGLENIERLDLSKNRIASIDALVFRQLKNLKKLDLSGNKITTVETNLFHDLLALERLKLNGNLLKTLSEGTFHGLKLLRQVDLTNNPWDCDCYLYWLSNWKNTSLFKLIPAPTCASPPPLHGHSLLDLRFSDELQCQFTSPIIDLQPDQNQVVFAGDSMTLHCSVPSITDDRSARLKWYWNPSIFEEAGAFVDPQDTLSNIKVENRYLSDSGAIDSSITIFPVTKEHNGQWNCELTSVYGNRSKTISMIVISDETKYCPLVITRNNKGMYAWPRTVVGWRVELPCEGLGLSGLVPIPLRASYHCNATGSWIDLNTEACPFISPITKALEQYSKVNLSLTKGNLLETAIRFKNHTSDSTKITDPIEIHFITKTIENYLNFLVEEKELGAMLIDIVSSIMNLPKDMLKFAETSYNACTRLIKAVELITEFTPSIQLHKNNMALEEFRVKRENFGGLTCTWYSETSGDKEVKLLHCATNNKTSILSTRDKAIEASIQLPPSLLRRLDLTVAHQLMISMYTDNSLFPVTSGFSPKIEVTSGVIGAKLIGLQVANLTEPVYVMLKVPEFLSRRPKPVIWDTVGNSSEWSTTGCQLVNLINDLVIFHCDRLGYYGLLEDTSHLEAVMAPVGEKFRYSNPAIYIGTFIIISCLTITSVTYIICHASIVMPKRAKHCVVNTWVSITLLCFLYTAGIQQTDNLEICQSVGLVQHYLSLCSLLWMAVTASTMYKRLAKPDITQVPDDEIPEQPIQKPLLGLYLVGWGIALIVCGISGAINLREYASYSFCFLSSGAALAALFVPAVILIFYLTIFYLLVRCAIRSGDHNGQLSEGTQATENMDLELLEPNDNRADQNSVHSTQTVSSEVEDVEHSQITQLKGHIVVLVLYLIMWCAAAAATSRPFNPHLPHEETIFAVLYAISASSLGFFVLLFYGIARSDVRSQWTIMRCWLRRKKNRCCRTRSVTDANPSLPAQPLVQNLTMPVQGTPQIVSDTNSLSSSRITSASRACNALKISDGGSDTPSITKKGPNMNLVVLHRQQYRSNNSVTTFTEATHSVEMFYNPHQSGVARKFFKKQRRHTKHNNLGPRKQGDGGATSDGGSCVSIPRPAKIQDSEIERSIFGSSAKVNNTNIHVELNPVTATKNPNILSDSGGSISEDRTMPLRYVIGQEHTRISRKINNDDIRMIHNHPPERIRINPNAIECIRIDPNLASESVRMSQNVADCIRMSPNESDLETRTEEEKHLRNVSQQCSLEYSSEMDSGGQMLSERSDHDLPEIDETPETPDKITEGDFKCSSLHELTQMVDDKALARSEHSSSLYCLSEDRFEEPPLRTSYRSSYNDVTSLGTSSRNCEDFESEGKPTTDDNSRCSSFSNVNRMIPSDEELELLNELTLPNLNERAIEPSDYEKEYNSMTDLTAIDITLGPTRHLDMNASIGVDYEDANYENSQHFSEDGVPLDDAIIDANHGKKETSV

>Hheb081860.1 Orphan

MKAQLPLGLSEDIGYVLYSALGSFYIPSCIMVFVYIRIYFAAKKRANRNIRKAPRPRPAIAIPPESPDIRQTSFTQLTPATDSRLSSGINTMDNVATIESPQIQIPIVTCDYASDVSTSEADPGANYNNMEEKDTLKSLVNLQVNAPMPMQKLNLKATLSVNGNEGQSTSPKLPARCRAPSVGIDVDMVSEFDPSSSDSGVVSKCAVVKPLKLRICRPIFGKKAINKVKKEHSSDGKHINRDGVVVDNSTPRPRDPEREKKRIARKKEKRATLILGLIMGSFIACWLPFFFLYILKPLYANLNTPGAAFAVAFWLGYMNSAFNPVIYTVFNKDFRRAFRRLLYK

>Hheb006770.1 AKH

MNATRELPIDMRFNDGHVVSIIMYPILMVISIIGNVTVLYLILQRRRSNRSRINTMLLHLAVADLLVTVLVMPLEIGWAITVDWRAGDAMCRIMSFFRLFGIFLSGFILICISIDRYYAVLKPLQLMDVDRRGKIMLVSAWVGAFICSAPQVVVFQQKSHPEFTWYNQCISLGSFPSYAHELTYFIFGMTMMYWLPLSVIIFTYSSILLEIYRKSKEAGDKIRRSSVGFLGRAKIRTLKMTITIVVVFFVCWTPYNIMSVWYFLDRESAREVDQRVQKFLFLFACTNSCMNPIVYGVFNIRRDTRGSTSQQAVTIGKKTSIENVVCRVSWRRQETGSIRDNNDQNGQDTDVCLDIANQLSYNIGKNHENLTKSFGEAQYDDLFAIAVKHPKEKKLNHHSGKQTLIATKYGSSDTKFLFMFDSTSRRDCQFGGEIPLNTTFREAMENGLTESAFQVFSDKKYHPRVYKNSRIQEGWKHANCMSNSLLDVDYLRDLNVTNMRVRRRGDQPSIHKLDELNCIGEQKLVSTLNTLIEYTNQILEELRRNTQETRYVRKLLEECQGCKAPLPPLPPLRPSCDYNAPQCYPGGQCRDTASGPICVCPPGYRGNGIQCERVSACATAKCYPGGQCRETERGPVCSCPPGHIGNGITCERIRTCDDRPCFPGVRCENTPRGYRCGPCPPGHEGNGETCQTIRITCEMSPCGRGVTCHPIHEPPHHRCGGCEPGWKQHGSECRDIDECDLENPCRPNEECKNTLGSYRCIPCAPGYRGSRTGCVDIDECSTNNGGCVPNSECINTPGSYRCGQCIQGFTGNQTSGCYQVGNLCPDRVTVCHERATCNCIVPNVEYTCQCQVGWAGDGFACGIDSDNDRHPDEELNTDGDIIPADNCPHIPNSGQEDVDGDGVGDACDDDADNDGVLNSSDNCPYDANVAQEDTDRDRKDGVGDVCDNCPSIYNPHQEDTDDDGIGDACDRDIDNDGIINENDNCRFVKNEDQYDSDGDGVGDVCDNCRSVPNSNQSDSDRDGVGDACDTGRDRDRDGIQDDVDNCPDVPNADQLDTDNDNIGNACDDDIDGDGVPNLIDNCPYVYNPRQEKSHPGISGDACWNDFDNDTIANPYDNCPNNSQIWSTDFRQYERIPLDPVGDAQLDPKWYTHDDGAEIEQTVNSDPGIAIGFDHFGGVNYEGTMFVNTDIDDDYIGFVFAYQNTHKFYAVMWKKNSQTYWRSEPFRAVALPGIQIKLVDSETGPGKEMRNSLWHTEDTPKQVKLLWRDPKNVGWKEKTSYRWELLHRPKIGLIRLWIHQGDKVVVDSGNIFNSALKGGRLGVLCFSQEMIRWSNLQYSCRETVPQIVYDELPADLQAKVGVDSPKY

>Hheb008350.1 ETH

MKMLTTMSSTAFELNSSYYTTAIGSSSTIDGFSVSVLPPATNATSIPYVLPAYIRITSMVVVIIVMVLGIVGNLMVPLVVLRGKDMRNSTNIFLVNLSAADLCVLLVCAPTVLVEVNSGPQVWPLGEHMCK

>Hheb008990.1 Orphan

MHSRMGNAILLSHRPSRDLNNLNGELNSGGSSKTLTLNEVNQDHHLHTPTKDKNLMKMKREHKAARTLGIIMGTFILCWLPFFLWYVSTSLCGAHCHCPEIVVHIVFWIGYTNSALNPLIYAYFNRDFREAFKNTLQCAFCSLCRREPFDLEALDIRRPSLR

>Hheb055420.1 Orphan

MEEPSTEALMQAGFIFVVSIAIILSNLLIIATYLNFRGPSEVINCYLLSLATADLLCGLLVVPLSVYPALMKRWVYGDIVCRLVGYLEVTLWAVSVYTFMWMSVDRYLAVRKPLRYETVQTKTRCQCWMAFTWISVAMMCCPPLLGFNKPIFDEQAFICMLDWGNMAAYTITLSILILGPSVITIVYTYFYIFSMKLKLRSGVPIHDKEYATALSENLSNPSHYMSFALIMTFWLSWAPYALLRIYISIQGAAEIPLLHFAVVWLGITNSFWKAFILGTMSPQFRLAARVLCLTLCCRHRRLPPELLGLDDDD

>Hheb074400.1

MSSDYLNVTNKIYPGTTKAELNLPYAVCEIVVAVCAVLGNGLVIIVFSREKKLRRRTNYYIISLASADLMVGLFAIPFAILASIGLPTNFYACLFTVSVLVVLCTISIFCLVAVSIDRYWAILYPMGYSRNVRTKTAIAIICVCWITGTLVGFLPLLGWNAGFKKTDEKCIFVEVMDYNYLVFLYFATIIFPAFLIAAFYAHIYRVVIQQQLMSRKPINIEGKRRKRKGNNNNNEPSSGTMLRLLGAAQKREVKATQNLSRIVIFFIICWFPLYTINCVQAFCPDCTVSEFVLNATIILSHLNSVGNPILYAYHLKDFRAALKNFILRILNPGRCRRDNNAMGMNEIQNKIISQRNLAKKSLNTAIKHTNSNLSEKGVTLALPEPNNIDSSPSSTPTINRSPTICIKYEFPEEDVGYVDESLSSNVKTSDNSENYNNSNNYDLIEDSRDFKDDSVLQLREHQITIEESQLKEIE

>RPRC001428 GPA2/GPB5

MTKSSSQFARNLASTNSATIVGNMAAGTSNLVTTRTRTPSSQTKVTEMLLVVSTVFIILNLPSYVVRVWIYLTDTHNVGTEQKVTMYVLQQYCNILFNTNFGINFALYCISGQNFRRALLSLFRPEIQRRSGETTQTTED

>RPRC001049+RPRC001048 Opsines

MELMLMPSAGFLAASIILFLIGFLGFFGNLIVIIIMCRDKNLWTPVNFILFNVIVSDFSVAALGNPFTLASAIAKRWFFGQSMCVAYGFFMALLGITSINSLTVLALERYLIVSQPVSHGSLSRPTALTIVGSIWLYSFVITAPPLVGWGEYGLEAANIRSANSTMNAGRVNKAESRVTWMIFVMIFAFFLAWTPYAILALMIAFFDSNVSPAIATIPAIFAKTSICYNPFIYAGLNTQFRQSWRRVLGGKREDSTTMATATSFGLNSKRYKEVSCVIDVKGKDKIKLSALNKSTATETAI

>DmCG11144

MKQKNNNGTILVVVMVLSWSRVVDLKSPSNTHTQDSVSVSLPGDIILGGLFPVHEKGEGAPCGPKVYNRGVQRLEAMLYAIDRVNNDPNILPGITIGVHILDTCSRDTYALNQSLQFVRASLNNLDTSGYECADGSSPQLRKNASSGPVFGVIGGSYSSVSLQVANLLRLFHIPQVSPASTAKTLSDKTRFDLFARTVPPDTFQSVALVDILKNFNWSYVSTIHSEGSYGEYGIEALHKEATERNVCIAVAEKVPSAADDKVFDSIISKLQKKPNARGVVLFTRAEDARRILQAAKRANLSQPFHWIASDGWGKQQKLLEGLEDIAEGAITVELQSEIIADFDRYMMQLTPETNQRNPWFAEYWEDTFNCVLTSLSVKPDTSNSANSTDNKIGVKAKTECDDSYRLSEKVGYEQESKTQFVVDAVYAFAYALHNLHNDRCNTQSDQTTETRKHLQSESVWYRKISTDTKSQACPDMANYDGKEFYNNYLLNVSFIDLAGSEVKFDRQGDGLARYDILNYQRQENSSGYQYKVIGKWFNGLQLNSETVVWNKETEQPTSACSLPCEVGMIKKQQGDTCCWICDSCESFEYVYDEFTCKDCGPGLWPYADKLSCYALDIQYMKWNSLFALIPMAIAIFGIALTSIVIVLFAKNHDTPLVRASGRELSYTLLFGILVCYCNTFALIAKPTIGSCVLQRFGIGVGFSIIYSALLTKTNRISRIFHSASKSAQRLKYISPQSQVVITTSLIAIQVLITMIWMVVEPPGTRFYYPDRREVILKCKIQDMSFLFSQLYNMILITICTIYAIKTRKIPENFNESKFIGFTMYTTCIIWLAFVPIYFGTGNSYEVQTTTLCISISLSASVALVCLYSPKVYILVFHPDKNVRKLTMNSTVYRRSAAAVAQGAPTSSGYSRTHAPGTSALTGGAVGTNASSSTLPTQNSPHLDEASAQTNVAHKTNGEFLPEVGERVEPICHIVNK

>Hheb080560.1 Serotonin

MMRDLNASACNELYEAVEWSGPGIVGTLVVLAIVDVMVILGNVLVILAVYHTSKLRNVTNMFIVSLAVADLLVGVAVLPFSATWEVFKVWIFGDIWCSVWLAVDVWMCTASILNLCAISLDRYLAVTRPVNYPQIMSPKRARLLVAAVWVLSFVICFPPLVGWKDQMSHPTKTDPVPEKNGPFNTTIILVPVKPCPWICELTNDAGYVVYSALGSFYIPMLVMMFFYWRIYNAAVSTTKAINQGFRTTKGSKMFGSRFDEQRLTLRIHRGRGSVHNGANNNSTSSPRSPESSRSASVRRDKIKISVSYPSTETLNTKCNTLERTPSKCSQISVHYTNGQTQNQLCTTSRNTHLKVGGINRVGSARRPSRRSSCESQVTGDEVSLRELATSSEDKPPRVMKMGKRNIKAQVKRFRMETKAAKTLGIIVGGFILCWLPFFTMYLVRAFCPNCIHSTVFSVLFWLGYCNSAINPCIYALFKATEAQVIQYVCHSRAYLSRTLIQIQALSIMSTHRVTLDDRRVKYGERLV

>Hheb033210.1 RYa

AVSVLVSAYTLVAISIDRYIAIMWPLKPRMSKKQAKLLILAVWLVALTVSSPIAFVSQLLQPNERYKKCNQFICQEYWPSAHQRYYYSIALLVLQYLVPIVVLMFTYTSIAIRVWGKRPPGEAENTRDLRMAKSKRKMIKMMMTVVIAFTVCWLPFNVLTLILDNNESINSWRGLPFAWTALHWLSMSHSCYNPVIYCWMNARFRSGFISALAGIPCFRKFWPERRTPPYNTSTAGGIALTGNVIYSVPKEQLKNPQQPLKKNQTKIQHCVDTKTL

>Hheb00794 CNMaR-2

MYTIQSYCAGSRLRRSLLQQEASLSSNDSYENGYDYEEEPLPCTMTDFTFLIALLFVWLNSELGWKVFNVAGWCEILVYVSAVCSSLSVWLIVAFTVERFIAVQYPLHRPQMCTISRAKTIICALVVLSLVCHSYAFITAGVVTVGDSDYCDLKVEYMDVMKIISTVDSIASLIVPIVLIVVMNTMIMRNLLKFSRRFKQTPMNSLITSNQCPSRERSDINLNQIPNKSSSQSGIMLATIVGKRGGSQQSFHSSRNSHSHHSSSGSGGPVIITTTQTANVTSPAPPSSSGARNISYQVPEVGGAKCISLYHHTKSIGTSSKSVVSTRNQQSITKMLLLISTVFILLNLPSYVIRLCIFFFTLAKRDSPDLLWCLQQFFMLLYYTNFSINFLLYAMCGMTFRRCLEQLVQKAFKGVTR

>Hheb09984 NTL

MTSCCLENESNITMPAYMAIMRPLRYHLSRRRTIGALVLIWLASVLLAIPGLLYSTTMTRRYSNGKTRIVCYIMFPDGDYLNSRIEYIYNLIFLGVTYLIPMTVMAVCYSLMGRELWGSKSIGEHTKHQKESMKSKRKVVKMFIIVVMIFAICWLPYQGFSIYLYHHSDISSSSYIQHVYLSFYWLAMANAMVNPLIYYWMNNRFRVYFQKIICSCCVIGRSNAGSCQMHELADFHRSDTARSNSGRLKFTTIRWRQSTAESHVHPYKIKSRSICEKIHTSRQDVAVI

>Hheb08603 CNMaR-1

MEERVITSNKTNQSDTEDWIRMGSTTDWVETTMIAIQMYYTPVLVCLGTLGNCLSVYVFFRTKMRRASSSWYLSALVVSDTGFLISLFFAWLDMVGIGIFNLSGYCQFFVYLTTLCSFLSVWFVVSFTVERFIAVQYPLRRQSMCTVARAKMILIGLTCVGLVLCSPVLWFSSPRPIDKKPNVTGCRLVEEWEAWASAFNVADTILTFVLPFTVIVILNGLIARTVYRLARVRRTLTTNGRNRRDQKCNGIGVSQTKVTKMLLIVSTVFLCFNLPAYVMRVRAFLEVHDLRSTIIAQQICNIFFETNFGINFVLYCASGQNFRKAVVRLIFRRPRRWHSGTPVSNHVSDFRRSSSTMGRQRTIVYEVPWTEAYEMRGLDSRVSRQLSQQSNQGKL

>Hheb03416 SIFa

MVGGLYTAKTLNSFVKSYYESILKITGAQRVHTWVLGRFMCKTVSYIQGVSVAASVYSLVAVSLDRLYLLPFWLSLLPPQVVSPVYPLTTDLATFTHPMLLLTHQSIMLPMLPMSMLSTSPLLQSYTLHQLFLAIWWPLKCQITKRRARLLIVIIWCVALGITVPWLLFFDIKIIPSSVDQAFCVESWPRKEGERLFFLIGNLMFCYVLPMILISLCYIAIWVKVSRRNIPTDTKDAQMERIQQKSKVKVVKMLVVVVILFVLSWLPLYVIFARIKLGSSPTIWEEDVLNIATPIAQWLGSSNSCINPILYAFFNKKYRRGFMAILKSGRCCGKLRYYETVAIMSSSTSMRKSSYYVNNNNSSTRRTCHGPPVHQDSNVSYIFNHTGV

>Hheb05423 TKR

MLLTYFLPIGSMTFTYARVGLELWGSQSIGEATQRQLDNIRSKRRVVKMMIVVVVIFAVCWLPFHVYFIVTSYRPEITNQPYIQDVFLAIYWLAMSNSMYNPIIYCWMNSRFRRGFAQFFSWCPFVKVTPEPGLSRSEAVTSRYSCTGSPDGHTRIARNGTSTCSSSDQSLSTIASGTTRRDTILHERVDIDLCEFTSP

>NL A42 ELeR

MKSALIADSAHDNSSEFHLKNMSYDGDYLEEYDYDLNDTCRVPWEELTPAVAVYSLTFVLGIVGNSLIVFTIFRYRRMKSTTNVFLASLASADLLLIIICIPLKIAKLFSYTWTMGVFLCKMVHYMQNVSAICSVLTLTAMSIERYYAIVHPMKAKYICTISQARKIIIGTWLASLLLGAPIMFIQDHIEVGAKIKGYWCVRDYVNNREWVTAYELYMLILILIIPTSIMGITYSSICWEIWRVMKQRKTMTSGKATLTTETFPLSSKRSTCSIKTTAKSCIRTTDEENGTVRQVIKMLVMIVIVFVICWGPLLVDNVLTAFGVLPDQKSGTLKHMYTGFTLMAYFNSCVNPIVYGFMSKNFRESFQKALCRCCRGPPKRTLSMSQTRTTSIRVSRLRRKQSKFQDMELTVM
