# Supplementary material for: Identification of Neuropeptides and Their Receptors in the Ectoparasitoid, Habrobracon hebetor
Source: Front Physiol. 2020 Oct 16;11:575655. doi: 10.3389/fphys.2020.575655 (PMC7596734; doi:10.3389/fphys.2020.575655)
Supplement: Supplementary file 7 [file Table_4.DOC]

>Hheb107890.1

MAWSDIDFNSPIIQQQMLEMGALRCRNGSAPEPGWCPETWDSILCWPPTPAGALALLPCPRYIAGFDLQANATRQCMPNGQWYENPETNYTWTDYSQCYNSELVTVLMDLPEVEERNKTLIKTYLPIVSKMSKIGYAVSFSTLLVALVILATLKRLKCSRNKLHMHLFVSFMLRAFMAMLKDILFVSDLGLASDVITKNGESFWVVDDVVHNNWQCKMFTSFWQYFILANYSWIFMEGLYLHNLVFFAMSADSNASIKGHVVSGWGLPVIFVIPWVVARALYDDTYCWTTNNYQSLFLIIRVPTITTIVMNFLLFINIVRILYVKLRNSVAEELERYKRLARSTLVLMPLFGVHYAIFLTISFSGLNEKVELVWLFGDQLFASFQGSFVAVLYCFLNGEVKTEVLRELRSKKWLGSRWGLGSGPRHSTRSNSTCSCNGKSGKLRKPRWKRPWFGLFHRRTIHRSSHSMASTQDVTTRGGSLTSSHGYLDSVVVEGNKTYNTDLHDRSVTQMSPLCSKFTDQSMLSFCSNYLPANFRLTR

>Hheb003130.1

MTNGDLERFLQDQQDLCDLHIAEQAGLNNGLCPPSFDGLLCWPATLANTTAQLACPPRVVLGYENPNNGTATKRCLASAVWYNNSEGIAWSNYTACTPPQGYYITAIENIEITHAEYAKANNSTLLMKWLPIIRNVSKVGYATSMISLIIALIIFSLLRKLKNHRNRLHMHLFVSFVMRAFMALLKDWLFVDGIGLAWDIVLVDGKSAFIKEKNIWVCKAITSAWQYFIVANYTWILMEGLYLHNLVFLAFCADTSAITLYVVFGWGLPAVIVGLWIIIRILMEDTLCWTTHTNPSLFLIIRIPIMVSLLFNFMLFLNIVRVLLVKMKTSDHLQRKKMRYGRWARSTMVLMPLFGAHYTIFMGLSYHEDHQIELIWLFCDQLFASFQGCFVALLYCLMNSEVLSEMRRAWRAQCARRGGVWLDLTQRNFGKNSERGHGNKGTNNVTSNVSML

>Hheb011390.1

MEVEGKDDEVWCRWAWDSILCWPPTKASTTARLSCPLENGIDTTKFAERRCSDDGRWEGKGGVINEQTDAPSGWTNYTPCFTPEMLLLLQKLYSGNEEDAKVKLNIAQRTRTLEFVGFSLSLAALLISLAIFCRFRTLRNTRTRIHKNLFVAMVIQVVIRLTLYIDQALDKTEVYNAQQGINNTPVLCEASYVLLEYARTAMFMWMFIEGFFLHNMVTVTVFHETSYYRMYRLVGWGFPAIMTITWATITAIYYHPSKCWWGYNLTGYFWILEGPRMAVVLLNFLFLMNIVRVLIVKLRQSRSSEMEQVRKAVKAAVVLLPLLGITNLISMAAAPLEVTIWFALWSYSTHFLTSFQGVFIATLYCFLNGEVRLALNKSVSLYMSVRTTERQARRQSTFSACQPQRIESENETAALGVNAINAPTIEHTSWLQVCWHGKNIQDQKQPADYRMKQIVTNQTVKIPEGLTVTVKSRRVTVTGPRGTLKRCFKHLAVDIHMVSPRLLKVEKWFGTKKELAAVRTVCSHISNMLKGVTKGYQYKMRAVYAHFPINCVTTENNSVIEIRNFLGEKFIRRVKMAPGVTVCNSAKQKDELILEGNSLEDVSRSAALIQQSTTVKNKDIRKFLDGLYVSEKTTVVQDDE

>Hheb104700.1

MALNASDQSEEVNYIHGTLNYINELNDSLLIKEFECLKAQHFEALEGRKTESTFCQVDWDRLLCWPTSPPGTLVKQPCFEQLHGIHYDSSQNASRWCWQNGTWDSYSNYSQCQELRMNVIESGIEITTTLYFIGYTISLSTLLVAVAIFAYFKELKCLRNNIHTHLMLTYIFADLMWIVTTIMQVSMQTGMPTCIIFFSLLHYFHLTNFFWMFVEGLYLYQLVVKTFTGDNIKLRSCLAIGWGAPMVIVIIWTALKSWAISADQSSQNVALYRHCPWMVHHNFDWIYQIPAIAVLAINVMFLFMIMWVLITKLRSANTVETQQYRKASKALLVLIPLLGVTYVLVLTGPESGQVADIFTYIRAILLSSQGLLVALFYCFLNSEVQTAVRHRLSRWSTARNLRSDRKFYSNCSPRSRTESI

>Hheb039610.1

TPGNLTTTVGSTVQEEELRRIILERKQQCMEMLALNTTLPAEPYCPGIFDGWSCWPNTPAGQKAYTKCPPFVTGFDTSFYAHKVCEADGSWFRHPVSNQIWSNYTTCVNVEDLNWQQGINVIYETGYTISLIALVLSLAILTYFRSLRCARITIHMNLFASFAVNNTLWLMWYGLVLPNTEVLVENGITCRFLHVILHYFLLTNYAWMLCEGFYLHTLLVSAFTSEHNLVKWLMAIGWPTPAIIVLIYAILRGTSDDPDDNSQCWIDEGNYINVLVYPVCVSTLLNLLFLFNIVRVLLTKLRAGPAIGSRPSRSMLQAFRATLLLVPLLGLHYLLTPLRPPKNHPWSQSYEIISAITASFQGLCVAILFCFCNGEVIAQFKRKWEGSALMRKRANSCTATTVS

>PpB1 PPU07227-RA

MALILKFLASILVLHYFDEVESVSNSTGESHLVEMLRRETMLEELYKCLMDIGPCNSEESVHLRDAIPMVFMTYCRTCDNHQKHTLLIFLMWHMFHSLDTLLIDDNDSTEMNIPLLPGNNTLSGVPTAFIAVGRLCQNCLLEPAYFAYAMTDCAFNYFCDIKMPGNTTMAGSTMQEDELRLLKLERQQECLGQLAVNATPPPEPFCPAIFDGWSCWPNTPAGTTAYAPCPDFITGFDPTLKAHKECEPNGTWFMHPVTHKIWSNYTTCVNLVDLSWQQQLNVLYETGYTISLVALLVSLGILTYFRSLRCARITLHMNLFASFAVNNALWLIWYGSIVANAQLLLNNGFMCRFLHVILHYFLLTNYAWMLCEGFYLHTLLVSAFTSEHKLVKWLMALGWPIPAIIVTIYTVLRATSNDNADTLQCWINEGNYMVVLIYPVCVSTLLNLLFLCNIVRVLLTKLRAGPAIGSQPSRSMLQAFRATLLLVPLLGLHYLVIPFRPEKGHPWEYAYGVMSAITASFQGLCVAILFCFCNGEVIAQFKRKWEGTAFARKRANSCTATTVSFVRSTAGPMAGEEKV

>PpDHR PPU05559-RA

MSNASLSLAYEAYEPDCQLLTSIYADENQRKEAKLLGDGYSCAAAWDSVLCWPKTKVGSMAVQPCFEELHGIRYDVSQNASRWCWANGTWDSYSNYSQCRELHQPESTVGESGVEITTYLYLVGYGLSLSTLIIAVIIYLYYRELKCLRNIIHTNLMFTYILADFLWIVMTVSQVSLQTNIPMCIIFYSMYHYFQLTNFFWMFVEGLYLYVLVVKTFSGDTIKLKMCLFIGWGLPFIVVVIWGMTKALAEDSITAGSHNEALSNHCPWMIPHPYDWLYQVPAILVLCMNVIFLFMIMWVLITKLRSANTAETQQSRKAAKALLVLIPLLGVTYILVIAGPTEGQAANMFTYSRAILLSSQGLSVALFYCFLNSEVRNALRHHYQRWSTERSLGHHPRQYAQYAPRSRTESIRLYSRHGGPGSGPGLDGCTNNHIKQHHNYEHSIHEHGNHVDTTMIQKEPHLMMSENSCVTAIVTVDDESTAATTYCEPGRCC

>PpB2 PPU09199-RA

MKSANKRLDTKLYDVIKSDDVAASANRRRYGGGGGDGSSSIRWRTDDMDFPSANGNDTQVHCELQNRNFIMPEDEIWCNWAWDTILCWPPTRASTVTKLRCPSANGIDTRKFVEKRCGDDGRWEGRAGTAGERDSPNGWTNYTPCFTPEMLLLIRKLYTGSEDLAKVKLDIAAKTRYLEFVGLSISLTALLISLSIFCRFRSLRNTRTRIHKNLFVAMVIQVVIRLTLYVDQAVFEPNGLQHGIQNTVLCEASYVLLEYARTAMFMWMFIEGLFLHNMVTVTVFQESSHYRTYRLVGWGYPIAMTLTWAIVTAFYYHPSKCWWGYNLTLYFWILEGPRMAVILVNFLFLLNIIRVLVVKLRQSHTSEIEQARKAVRAALVLLPLLGITNLISMAKAPLDKSIWEFALWSYTTHFLTSFQGLFIATLYCFLNGEVRLALDKTISVYMSVRGTDLTRRQSTISGCQPHRMASVIEVEESEIRPGAGWIRLCCRGGNMPPPDRPAETTL

>NvB1 XP_003424370.2

MDDLELASEVPGNTTMAGSTMQEDELRLLKLERQQECLGQLAVNATPPPEPYCPAIFDGWSCWPNTPAGTTAYAPCPAFITGFDPTLKAHKECEPNGTWFMHPVTHKIWSNYTTCVNLVDLSWQQQLNVLYETGYTISLVALLVSLGILTYFRSLRCARITLHMNLFASFAANNALWLIWYGSVVANAQLLLNNGFMCRFLHVILHYFLLTNYAWMLCEGFYLHTLLVSAFTSEHKLVKWLMALGWPIPAIIVTIYTVLRATSDDNADTLQCWINEGNYMVVLIYPVCVSTLLNLLFLCNIVRVLLTKLRAGPAIGSQPSRSMLQAFRATLLLVPLLGLHYLVIPFRPEKGHPWEYAYGVMSAITASFQGLCVAILFCFCNGEVIAQFKRKWEGTAFARKRANSCTATTVSVRWRERRRYDYQSAPSADPRDDNHSNNNRFSGSRDLPEPQLQPLTIVGTAPAETTSNGSAANHAPRLLLRRSDSLLHEAANTTSNNNNNNNHQSQC

>NvPDFR XP_003427082.1

MDFPSANGNDTQVHCELQNQNFIMPEDEIWCNWAWDTILCWPPTRASTVTKLRCPSANGIDTRKFVEKRCGDDGRWDGRAGTAGERDSPNGWTNYTPCFTPEMLLLIRKLYTGSEDLAKVKLDIAAKTRYLEFVGLSISLTALLISLSIFCRFRSLRNTRTRIHKNLFVAMVIQVVIRLTLYVDQALFEPNGLQHGIQNTPVLCEASYVLLEYARTAMFMWMFIEGLFLHNMVTVTVFQESSHYRTYRLVGWGYPIAMTLTWAIVTAFYYHPSKCWWGYNLTLYFWILEGPRMAVILVNFLFLLNIIRVLVVKLRQSHTSEIEQARKAVRAALVLLPLLGITNLISMAKAPLDKSIWEFALWSYTTHFLTSFQGLFIATLYCFLNGEVRLALDKTISVYMSVRGTDLTRRQSTISGCQPHRMASVIEVEKAEIRPGAGWIRLCCRGGNMPPPDRPAETTL

>NvDHR

MSNATLGNLRLGEPEPDCQLLMSIYESKNQRKEDELVGKSCPVGWDTLLCWPKTKAGSMAVQPCFDEFNGIRYDVKQNATRWCWPNGTWNGYTNYTQCRELYVPELPVDSSGVEITTNLYLIGYGLSLSTLIIAVIIYLYYRELRCVRNIIHTNLMFTYILANFLWILMIVSQVSLEKNIPICIIFYSMYHYFQLTNYFWMFVEGLYLYVLVVKTFSGDIVKLKTCLFIGWGIPLIVVLVWGIIKALAENSAAAGSPVRALSNHCPWMIPHPYDWLYQVPAILVLCVNVIFLFMIMWVLITKLRSANTPETQQYRKAAKALLVLIPLLGVTYILMIAGPTEGQVANLFAYSRAVLLSSQGLLVALFYCFLNSEVRNALRHHYQRWSTERSLGHHPRYYAQYTPRSRTESIRLTLKEHKILES

>BmB1

MNDPDRIARDLLLCEEFNNRTLPPPGLYCEGTFDRWLCWPHTPANSTAYGSCPEFVPGFRPDLLAHKECTANGTWYKHPETGLPWSNYTTCVVEEDDVNHIIVVYEAGYSVSLVALLLSLAILLYFRSLRCARITVHMNLFGSFAVNNALWLAWYGLVVRDPTTLQEPPVWCCALNAVLQYAMLTNYMWMLCEGMYLHTVLVSAFISERRLVRALVAAGWMLPLPCILIYATRRALDGDPLCWAEEPESRPELAVPVGLAVLLNLCFLCNTVRVLCTKLRAGGAAGGAARPSATALHALRATCLLAPLLGLQYLLMPFRPANTVSWWRAYEYATAAATSLQGLCVAVLYCFCNGEVLAQLRRRWRALTFRPRANSTTATTVSFVRSAATAGGEDKA

>Dm_CG17415 DH31

MSDQIGNPNATFSGSGSGSGTNVASIAESVAESGPDFDALRAACETRLNASGQLAGSGGPGAEAGTHCAGTFDGWLCWPDTAVGTSAYELCPDFITGFDPARYAHKECGLDGEWFKHPLTNKTWSNYTTCVNLEDLNWRHTVNLISEVGYGTSLLAILLSLAILGYFKSLKCARITLHMNLFASFAANNSLWLVWYLLVMPNSELLHQSPMRCVALHITLHYFLLSNYSWMLCEGFYLHTVLVAAFISEKRLVKWLIAFGWGSPAIVIFVYSMARGLGGTPEDNRHCWMNQTNYQNILMVPVCISMFLNLLFLCNIVRVVLLKLNAPASIQGSCGPSRTVLQAFRATLLLVPLLGLQYILTPFRPAPKHPWENTYEIISAFTASFQGLCVAILFCFCNGEVIAQMKRKWRMMCFSNRPRTNSYTATQVSFVRCGPPLPGEEKV

>BmB2 NP_001127733.1

MTVESRRLTPELSLISTGVRYCQPRFDGYLCWPPTAAGETTYLRCPVARLSDSTKNAYRRCGIDGLWNTKKFNETSEIGWTNYTPCFPMEIRNMLNELYEEDETGAQDKFNVALRTRYLEIFGFTLSFIALSISLYIFIHFRALRNHRTRIHKHLFGAMLVQVLIRLTVYIDQAVVRSSISSTDDNTTMTRGIDNMPYICEGSYVLLEYATSAMFLWMFMEGLYLHNVVAANNLRERVPYQWYCVWAWGAPVVITSIWTILTALKYRGEKVQTCWYGYNFTGIYWIVQGPRLIVILINFVFLLNILRVLIMKLSKSARREIVKVRKAVRAALVLLPLLGITNIMNMFEAPLSSDPIRFAVWSYLTHFLRSFQGFFIALIYCFLNGEVKQCLSKAYTNYMAERALLVRQNAICVPSMDEEPAKEKKASYLSCCFPSKTEKRTSKDYRACALDLNKDEEPAKEKKASCLSFCFPSKTEKRTSKDYRAGALDFNNTARARRLELHMVSNSQLPTPLQSPCPAVVRRQMELQELKQSTPLIPRRHIVSAPKRAVRIPFDDDDEAVTKEPFSCYADTLEMAELGAVSPWVYRRRCSPPEMGIFDTCSLTRGSCDEQPPIHIERCIYATRAIVPSSPYVADTDAREVSK

>Dm_CG13758 PDF

MTLLSNILDCGGCISAQRFTRLLRQSGSSGPSPSAPTAGTFESKSMLEPTSSHSLATGRVPLLHDFDASTTESPGTYVLDGVARVAQLALEPTVMDALPDSDTEQVLGNLNSSAPWNLTLASAAATNFENCSALFVNYTLPQTGLYCNWTWDTLLCWPPTPAGVLARMNCPGGFHGVDTRKFAIRKCELDGRWGSRPNATEVNPPGWTDYGPCYKPEIIRLMQQMGSKDFDAYIDIARRTRTLEIVGLCLSLFALIVSLLIFCTFRSLRNNRTKIHKNLFVAMVLQVIIRLTLYLDQFRRGNKEAATNTSLSVIENTPYLCEASYVLLEYARTAMFMWMFIEGLYLHNMVTVAVFQGSFPLKFFSRLGWCVPILMTTVWARCTVMYMDTSLGECLWNYNLTPYYWILEGPRLAVILLNFCFLVNIIRVLVMKLRQSQASDIEQTRKAVRAAIVLLPLLGITNLLHQLAPLKTATNFAVWSYGTHFLTSFQGFFIALIYCFLNGEVRAVLLKSLATQLSVRGHPEWAPKRASMYSGAYNTAPDTDAVQPAGDPSATGKRISPPNKRLNGRKPSSASIVMIHEPQQRQRLMPRLQNKAREKGKDRVEKTDAEAEPDPTISHIHSKEAGSARSRTRGSKWIMGICFRGQKDKCVMPGSQKTQQIFMTSQMPPTSTLAAVATTITTTSTTTTAAKTTIASIATIATMTKSKAKAKAISKSHQIQMPKA

>BmB3 BAG68437.1

MFRCCSVEDLLYLLILFVSLTKGESFSDDAFDLLDGDLDLWHERGSRVAIREDAPTCERQSFYICEEPLNGVPPEAHKTCNYRNVDYHEQVFRWVAGRGCIIYTPSFLFVGGANAMNTSQCFYRNYRVRCLEIPKEDGTCGCYPFDPSFAEVANIIREAIIPSAHGRWERCFYAAQDCCSHYMLEDTNSIDKNECGVTFDGWTCWMAAEAGTVSNAVCSEFAYSNTGPSCNHFSSKECYQNGTWEVESVYNTCSVTPRLVQRYRFHIAMLSISVASCLPAVFIFFFYKRLRITRVALHRNLLIAIILRNTFVIISRNEIYLDELQNTGDTVMTIHGLACRFLAIGERVAGNAVFVCMLVEGIYLHRLIVAVFKQKLNIKSLYAIGAVIAVAPVIAWVAVMAVHNDHSCWLVYTIEHVQWILDVPRVAILLVNTVLFGDILRVLLTKIRNNENSHQLSTTKATLFLMPLFGTQFLLTAFRPNTTDCTGEQIYYYVSYTVEGLQGFVVAILYCYINKEVRSLIKATYKKTESAVVSRIRRDSTHPSMSVDPSSGRRMTYSTGIPSQGDIIKEQYAVVAPKLHVAEIISIQPSERLVEIIEPVYETISNSVNNDSYDALDRSDNDPKGRSKIDDYYNFTNASSITIDCPEWIRCVSSPNSSIYNNSLNDNENKYLASHIPDIPESCNEMKHSKNEQAEKNNLPKHDIGENNEDFEDYDADFETKTSADDSGNMLEEIMQCIENKNDLSIPNLDPERLCPNRNGEDKIIFLNE

>BmB4 NP_001127735.1

MLSRFLNKLFSQTNVTENPQLKVLYTRYEECFVSNETHNTIVQSEYDENAPRCPKTFDGFSCWDETPSNTTVVQNCPELINGFDPSRTAFKECLENGTWLEHPESKKVWTNYTTCIDYDDLRFRNVINNMYVGGYAISLIALILSLMIFVFLRHFQYKRTRIHANLFISFILNNIMWIVWYKTVVDYIEVVQENTIWCQSLHILTYYHMMTSYMWMFCEGLHLHVGLVSINEYISVRSYCAIGWAIPALVVALYTGVRMQLRYGTERCWMDQSHALWIIVIAVVIILTLSFLFLVNVIRVLLKKMQPPASNQPNEAAKKAARATATLVIYFMIPLYGLHFILIPFQRAPESIGEKIYQVVSALLTSLQGLCVSILFCFTNNDVKTALTNYLARYKRKTEAIQMTGITTGESAVNPNAA

>BmDHR XP_004933474.1

MIAAIIERLYGQEMLNDTAIIEAKTKVPCTIRKYFCRLPMFTFGALSLDKLEHKPRVNIFIMYIFNGKLEDLQTLESAESVIENATARTECLARNANLTEYYCPAYFDGLLCWNPTPSHTVAVQKCFKEFFGIKYDETQNASRLCLDGVWQNYTDYSNCTERIANVSPTDVASLIYLTGYSLSLAVLSLAVFVFLYFKDLRCLRNTIHTNLMTTYILSACSWILNLALQNWSDEAQQDQTSCMILVICMHYFYLTNFFWMLVEGLYLYMLVVETFTAENIKLKVYTTIGWGAPAIFITIWVVSRCFVNVMPSTGPDGLALAGEAKMCIWMHEHQVDWIHKAPALAGLALNLFFLVRIMWVLITKLRSANTLETEQYRKATKALLVLIPLLGITNLLVLCGPSDDSWFAYSFDYARALMLSTQGFTVALFYCFMNTEVRHAIRYHVERWKTGRNIGGGRRRGASYSKDWSPRSRTESIR

>Dm_CG12370 DH44-R2 NP_725175.3

MADDDLRALVDSLDDASQEDLAKVIANFSVDMLQRASALIGAQQGSSGGQLQNRTLQCQQQQQREEEQASLEALASGGKRILQCPSSFDSVLCWPRTNAGSLAVLPCFEEFKGVHYDTTDNATRFCFPNGTWDHYSDYDRCHQNSGSIPVVPDFSPNVELPAIIYAGGYFLSFATLVVALIIFLSFKDLRCLRNTIHANLFLTYITSALLWILTLFLQVITTESSQAGCITLVIMFQYFYLTNFFWMFVEGLYLYTLVVQTFSSDNISFIIYALIGWGCPAVCILVWSIAKAFAPHLENEHFNGLEIDCAWMRESHIDWIFKVPASLALLVNLVFLIRIMWVLITKLRSAHTLETRQYYKASKALLVLIPLFGITYLLVLTGPEQGISRNLFEAIRAFLISTQGFFVALFYCFLNSEVRQTLRHGFTRWRESRNIHRNSSIKNRRHRASKDYSLRSRTESLRLTSTSPIPTGHYE

>Dm_CG8422 DH44-R1 AAF58250.1

MSDHNHIDSVNASGSDPLLDLHNLDGIGESVELQCLVQEHIEASTYGNDSGHCLTQFDSILCWPRTARGTLAVLQCMDELQGIHYDSSKNATRFCHANGTWEKYTNYDACAHLPAPESVPEFEVIVELPTIIYYIGYTLSLVSLSLALIVFAYFKELRCLRNTIHANLFFTYIMSALFWILLLSVQISIRSGVGSCIALITLFHFFTLTNFFWMLVEGLYLYMLVVKTFSGDNLRFNIYASIGWGGPALFVVTWAVAKSLTVTYSTPEKYEINCPWMQETHVDWIYQGPVCAVLIINLTFLLRIMWVLITKLRSANTVETRQYRKAAKALLVLIPLFGITYLVVLAGPSESGLMGHMFAVLRAVLLSTQGFSVSLFYCFLNSEVRNALRHHISTWRDTRTIQLNQNRRYTTKSFSKGGGSPRAESMRPLTSYYGRGKRESCVSSATTTTLVGQHAPLSLHRGSNNALHTMPTLAANAMSSGSTLSVMPRAISPLMRQGLEENSV

>Nl B1 BAO01101.1

MTGSNNTTEIVYEQEIIKQKYQQCMVQLELSNQTGPHFKEPYCKGVFDGWSCWPDTPAGTVAFAPCPEFITGFDPTFIAHKDCTANGTWFKHPISGLVWSNYTTCVNMEDLDWSQKINTLYETGYAISLVALLLSLIILSYFKSLRCPRITLHMNLFTSFAINNLLWLLWYRLIVPYPDIVKQNGVWCQCLHVILHYFLLTNYSWMFGEGFYLHTLLVSAFISEERLVKWLTRLAWIIPFIFITIYMKLRLFFGQTDKCWIEDSPYMSVLTVLVCCSMVLNLIFLCNIVRVLLTKLRSTPSGMASSTSQAPSRSLLQALRATLLLLPLLGLNYLVTPFRPHKGHPWEKYYELTSAITASFQGLCVATLFCFCNGEVIAQIRRRWQYIMFRPRANSYTATTVSFVRSTACPNTGEDNV

>Nl B2 BAO01102.1

MPVRRVSSDGNRDPLVAHRASLLAVPGLHPTSGVGSDVGSCRQLDLSLTSPADTTAAAMSDDISPECVSPAVLLVPPPYPEASDEVSSTSKSDQGIQITVVEDTTEDDKQPQSQPQPQSLHHRPVANKRSLELCAARYHEYSAPLGVLYCNWTWDQVLCWPPTRAGTTATQRCPRDKGIDPTKFATKKCSAEGRWEGKHPGEEATSPQGWTNYTPCYTPEMLELFRKLYVGSEKAAMKKLEIAERTRTLEIVGFSISLAALLISLAIFCHFRSLRNNRTRIHKNLFVAMVIQVVIRLTLYIDQALVKGNSLTGQRVSSSSSSSGGFRQGIDNTPVLCEASYVLLEYARTAMFMWMFIEGLYLHNMVTVTVFQEKSYYAAYTMVGWGVPVLMTAAWAMTTASTLGSTRCWWGYNLNKFFWILEGPRLIFILMNFLFLLNIIRVLVVKLRQSHTSEIEQVRKAVRAAVVLLPLLGITNLVNMTEAPLTRAVWEFGLWSYTTHFLTSFQGLFIAILYCFLNGEVRMAVRKSVSTYLSLRPQHFTPQRRNSAFVSANMTEQVVSTPPPRPATSAPRSWFRSCCSHPSQPTPETRV

>Nl B3 BAO01103.1

METFLEGDLTLEQIIITRRENCEDIKDFTPANDTGEVYCPREFDGWSCVNWTSAGQVAHFPCPYFILGFDPKRFGQRTCLMDGTWFRHPDSNKTWSNYTTCVDLEDLELRKHVNFIYKTGYSISLAALIISLFIFFYFKSLSCTRIQIHKNLFLSLTINNCLWLIWYEAVVDNLPVLMTNGLGCQALHLLVQYFLVATYLWMFCEGLYLHTLLVVTFLTESRVMPLLYFIGWGVPAILVLIYAAMRSSLREEKLHCWIHESLYSWTLSGPVCISMLANLVFLINIVRLLLTKLHTGQPVSPKASFQDPQSSVRLRRRNTVLSQTGVMSGRTRKAVRATFILIPLLGLQYILMPFRPQQGAAWEPAYQIISAVVTSYQGLCVASLFCFFNGEVSKEGN

>Nl B4 BAO01104.1

MSPEQQNASLRFFKKLKEDCDHKRRLKLVSAAEGVTEIEELQCPAVFDGFTCWDATPAGENAFAPCPDFVTGFEKTRFAFRSCMENGTWFRHPQTGKYWSNYTTCVDMEDLEFREFVNSLYVTGYSVSSAALLISLLIFLTFRSLRCTRIAIHVHLFVSFAANNLMWIVWYKQVVGVTKVVQENDIYCQALHIILQYLMVANYMWMFCEGLHLHLALVVVFVKDDCAMRLFYFIGWCLPLFITTVYTLVRMSYPHDTSQCWMGDSYSQWVLIVPVLLSMLASLAFLINVVRVLLTKLHCNSANPAPIGMRKAVRAALILIPLFGIHHILIPFRPEPHAPLEMTYQIFSALLVSLQGFCVSVLFCFANVDVHCAFKNVLRRMRRRRAGDNNGTVTQQTQTREMV

>Nl B5 BAO01105.1

MGRAQYASHCPRSWDSLLCWPPTKPGHTATMACFPELNGIKYDTTLNATRRCLLNGTWDNYTDYTSCKDLSPDQPDLEPGIEVTTMIYSAGYALSLIALVLAVSIFLYFKDLRCLRNTIHTNLMCTYIMADFMWILNITVQMSMPTNVPACVILVVLLHYFYLTNFFWMFVEGLYLYMLVVETFSSKNIKLRAYVCIGWGVPCAVIIIWTIVRSLMPPTSDNMGLSGIVLKSCPWMATHNIDWIYQGPAAVILFVNVIFLVMIMWVLITKLRSANTVETQQYRKAAKALLVLIPLLGITYILVIYVPSHGVTANPLAYCRAILLSTQGFTVALFYCFLNSEVQHTLSSHFERWKEARQIGGETLC

>NlB6

MTPGGELVSLPCPEYYRDFDPQKRATRRCLEDGRWYWSDESNATWTNYTECFSKKAPYVFPTPSTLISSYLPIVKAVSQIGYSVSLITLIVAFCILATFKGLRCPRNKLHMHLFLSFILRACTTLFKDLVTDSLSSRLVISETSVSETVILHQQTEDWTTCRAVTSLWQYFLLANYCWILMEGLYLHNLIFLALFTDSSSIALYIVLGWGLPAIFVLPWIFVRAAWENTLCWTTNSNPYYLLLIRGPTTVSILINFALFINIVRVLLSKLQASICEENKKFRKWAKSTLVLVPLFGVHYAIFIGMSYVEGSELEIAWLFGDQLFASFQGFFVAVLYCFLNGEVRTEISKKWRRWWRSRKSSPHNSNYVDKNAYGRCSTLRGCFNALAHGKNGYNMREGDYSKREST

>NlB7

MTEANDDDELTIKVKELEAKCHLIQNRIEGVGLTCPPVWDGLLCWPATPAGIVKSLQCPDYFHGFNPTRQATKRCLEDGTWYWSHESNSTWTNYTQCFSKETVANTDFSSHHQILFRYVPIVKFVSQVGYSVSLFTLIIAFCIFATFKGLRCPRNKLHMHLFLSFMLRAFSTLFKDLETNRLERFSATSGELISLREDWASCRAVVTLWQYCLLANYSWILMEGLYLHNLIFLALFSDTSSISLYVALGWGLPLLFVAIWALARIVWENSMCWTTNENALIVMLVKGPTTISVLLNFVLFIKIVRVLLTKLQASVNEQNRRFKRWAKSTLVLMPLFGVHYAIFISMSYVDCGPEIEIIWLFGDQLFASFQGSFVALLYCFMNGEVRAELNKKCLRRWPSNSAANKRRRQSETTGQYSNTCAVRPHHYGKGRDNRKSLGRLETSVTSITTSCPFDPSSRKSLPSNGLHRNHSESTWLTEQSSFVNSKVTAGNGIELMALHEKYEEL

>Tc67 XP_969953.1

MTNIYVKKILDNQETARKAAKEKCISMFGPGVLQEFAEPVYNETNKTLKCPAFWDTILCWPDTPSGTVINQSCPVYVAGFLASSNATRQCMENGSWYIRDNRTWSNYSSCYKDKITTIYLDLNKTNISGVLQNYAPVVQVISETGYIVSFATLIIAFAIMLFIKKLHCARNILHMHLFASFILRALTFIVIKSTFVEGLGLPSDLNYRNGSLYFDINSETNNWACKLLTSLWQYFITANYSWILMEGLYLHNLIFRALFADSSNSIKWYVVMGWGLPLIIVGFWVAARLLVEDNLCWTTHENYDVFLIIGIPTMVSILINLLLFMRISMVLYSKLRSPINEDSRRYQKWVKSTLVLVPLFGVHYALFLALYYLIKTNKIVEVVWLFCDLLFGSFQGFFVAILYCFLNGEVKSEIQPHLYYFLTYLATNKYSKCLFPCRKKFLRSVVGRSSVCTTMSCSSLYTNGVLHRNSKCRLDSLPKIKTTDKPKDHFCNNTSRRNSRSQRHSQAFNSSATIPTAETTLCIENNSGKCRSESEINEESLKMVVHSDF

>Tc68 XP_975039.1

MSEFQDILNTAKRKCYASKTTEIITGKFCELIFDDILCWPPTPAGLLANQSCSNEYIKWTRNNYATRQCDSDGQWFIPENSTESWTNYSQCGNISEFYPILDDSLRNHTLYNKWLPIIKNITQCGYILSTVSLIISLFVFIRIKRLHCARNKLHIHLFASFVMRALMSLIKDGLFIEGTALPHEIIQINGKLVYNKTNFSWVCKAIISLWNYFIISNYMFLLMEGAYLHNLLFLKLLSENGVVIYYSLGWGIPLLFIIPWIVLKAGNENIYCWTTKSSKFIAMLIDVPIGLTVVINFILFTIIVRILFVKLTSMYIQQRWTKYQKLIRAILILVPLFGIPYTISFVLSFYALEDQTFEIMWLFFDQTFTAFQGLFASLVYCLLNSEVQMEIMRKYNSFKDRNREFKRRSRTISHTQQIPLTEELQEMPHNAGIKDQLCINKTSDYF

>Tc69 XP_971738.1

MCDEIKECAQITRTTRTIEMVGLSFSLISLIVSLIIFFQYRVLKNNRTKIHKNLFIATLLQVVFRLIKYVDQELKAGDRIIENTPILDEACITLLEYSKTAMFTWMFIEGLYLHNVITVTVFQEYSYIKIYFYVGWTAPAVITAVWVWTMKMVRSNWGFYYFLPYYWILEGPRSTIIVVNLLFLINIIRVLIVKLRESHTSEIEQVRKAVRAAIFLLPLMGIANILFWMGYRFTQGWKLALWSYTSYFLNTFQGFFVAILYCFLNGEVQTAVKNSFYLHMSLRNHDYTPCRNLTLISTAPDPEQHIEKESTNWIRYCLKQNKKSPEEKEISVCDLNDRRNSAIVTALTMVETKPLTRFVVATRISQYVAKAIRRNRYDDNRRQKFRTFATPESHNEHGERKNLFPSIAISMTLMSPYHKVYKSDITT

>Tc70 XP_969030.1

MEQMRQHCLVFYKCEKDGEWNFNEQFNKSWVNYTTCINIEDFEFRQQIILIYCVGYGVSLVALLVSLALLTYFKSLRCARITVHMNLFSSFAMNNFLWLLWYSLVVNDQDVLHENKLWCRVLHVVLFTFLISNYSWMLCEGIYLHTVLVSAFISERRLLRCMLALGWGIPLLTTSIYAPVRSVLGENVDELGRCWTQDGRFNKILMVPVVITVFLNVIFLVNIVRVLLIKLRKGPANGGSGSGASRTSLQALRATMLLVPLLGLNFLLTPFRPEANHPWEYVYEVVSALTASLQGIQPFLMFFWYRDQNHSW

>Tc71 XP_008193920.1

MDVTNGTNCGGKYTRPGYCPEIFDEMLCWPETLGGTTVNQSCPKKMGYDSRRFAYKDCLENGSWFKHPKSGKIWTNYTTCVDHEDLAFRTHINHLFVIGYSISLAALVISLAIFFTFRTLKCTRIRIHIQLFISFALNNLMWIIWYKEVVPNPFVTIRNELWCQALHLVVHYLMLANYMWMFCEGLHLHLALVVVFVRDAETMKWFFALGWGAPFIIVLIYSVVRIFILKDNYMCWMADSYYSSWILTAPVCISLLVSLIFLINVLRVILTIMHPNSANPAPMGLRRAARAALILIPLFGLQHILIPFRPDMYDPYEHLYQYVTVVVVTLQGLCVSCLFCFANQDVHQAIRGFMHRKVYRTTRWSNYHYTGAADSAGVYVVNGSSHCNNVGLLSLKRKSTTTVKL

>Tc72 XP_968807.1

MRFNYIVVILISVCLLISTKNIQAQAKLCRSSQGLIHRSFWNRWTGYLCYHYDKRVKFKYNYTIIPYDSSYIPVTFINNKTDFFWYDFRNETSLNYIRPSYKSDTIFEKWKTCAQEASNCCDYSMTSENINPSDEYPCPAIWDDWSCFSPAKAGSVSKILCPDLTFSSEIRECELQAQKECFRNGTWKSKTDYGPCSVPAVLKTRHRFHIIVLSVAAVLSAPAVAIFYSFRAFRLQLRFILHRNLILVIIIKNLLVVITKEMVIMEALTSDGDDTILNGNSVTCRVLAFFENVAKNGVFAAMFLDGFDLHRSIVRPFADAMSSRFVYCAFFVLSCCPALIWALLRGLHQGEFCWVVDNTGDQWVTDAFRLTILTVNFVLLVDIIRVIVLKLRHDSVSQQTKTTLRVTLFLVPLFGVHIVITVNRNMVPDNTCDSMDVYYFATYLIEGLQGVLVAFLFCYINKEVHHEIMNAWRKTVICLQQKLDAKSERTTSATIVESMTCY

>Nl A50 BAO01100.1

MQRRAVATALVTAVTLTHFTVKADQDGVEPHPTWPFAVKADQNGVEPHPTWPPPPQLPPMVQSHEPGCSCSDHRDPPRLLVKCKCRGDHLQRVPSDLQRGLHILSITRAAIEVLAADSFQPYRESLTDLSLVRLPNLRLIEPGVFNNLPHLRTIDIHSAPMLTIISDAVFQTHLPRLRIFRCTNTGLQQIPALRDLESKHQLHLVDLDSNRISLIPERSFYITSDQVSLNYNQIEEIEAFAFHNSTIATLSLKGNRNLHILSEDSFSGLNSLRKLDLSETAITFLPTLGLRGLDELRLQGTTSLKVFPSVYSFDSLKDVYLTYSCHCCAFRFPARHDPAGFRRHKEFVEKMIRDCSSSFSGDNVSYNDRANQQNISFGPANTSFWNNIEFTSGSSTTSSEETFHSIVAVSPNGQLQVRCGHMLGGGGSKGRLQGEGPRCFPAPDAFSPCEDLLGSGWKVRISAWLVSLFALVGNTCVLLVLLSSRFRMSVPKFLMCNLALADLCMGLYLLLIAIADARSQGAYFNYAIDWQNGIGCQAAGFLTVFASELSVFTLSVITSERWYTITYAIHLNKRLRLGSASRIMAAGWLYSIAMAALPLLGVSSYSITSICLPLQTSSAVETVYLATMLAVNGVAFGVVCVCYGLMYASIRGQGQGRSGRVRSDLSVAKRMALLVLTDLVCWAPVAFFGLTALAGHPLIDLPSAKLLLVSFYPLNACANPYLYALLTRQYRRDLLALFARYGICSKQAATRHRGGLAGGGGHGEARGTGGRGSRAGEGEMVLGHSPHHTGGLYSPP

>GL561032

MSDETGNQSFLDPHAELVNSRYLQCLTTINESLSRSLQGNICGLQCEATFDGWSCWPATSAGETAFAPCPHFITGFDPLIFIYFQMSMDTDMVSCIILVILLYYFHLTNFFWMFVEGGLYLYMLVVETFNRENIKLRAYLAIGWGVQRGCAWMSPNSSDWINQAPAIIVLAVNLIFLVMIMWVVLITKLRSANNVETQQYRKAAKALLVL

IPLLGITYILFIVGPTEGQYAVIYSYIRALLLSTQGLTVALFYCFLNTDVQNTVRHHLSRWREARDIDARRYTHTKDWSP

>RPRC004735

MGTWFRHPASNKTWSNYTTCVDLEDLKMRTQVNMIYKGGYAISLAALTLSIFIFFYFKSLTCTRIQIHKSLFISLAVNNLLWLIWYEAVADNLPVLFANGFGCQLLHILVQYFLVATYLWMFCEGLYLHTLLVVTFVTESKVMPFLHLIGWGVPAILVTIYAILRMSNKEDSVHCWIHESLYSWTLSGPVLVSMIANFVFLINIVRLLLTKLHTTAQTSNENAPSGRTKKAVRATLILIPLLGLQYIVTPFRPNQGTPWEYAYQVTSALVASCQGLCVALLFCFCNGEVVAVMRKKWRQCRISKKRPWHSCSGVTSVSRHRRI

>GL562334

LIFIYFQMSMDTDMVSCIILVILLYYFHLTNFFWMFVEGGLYLYMLVVETFNRENIKLRAYLAIGWGVQRGCAWMSPNSSDWINQAPAIIVLAVNLIFLVMIMWVVLITKLRSANNVETQQYRKAAKALLVLIPLLGITYILFIVGPTEGQYAVIYSYIRALLLSTQGLTVALFYCFLNTDVQNTVRHHLSRWREARDIDARRYTHTKDWSP

>GL563066

ADYCPRSWDGILCWPPSPSATIVYLPCFEELHGIKYDTSRKFLFLCINASRWCLWNGSWANYSDYDSCSHLQIPPFAADPGLVVVTMIYLIGYGISLIALCVAVAILIYYKLRCLRNTIHTNLMCTYILAAFMWILNFTLQVSLIFIYFQMSMDTDMVSCIILVILLYYFHLTNFFWMFVEGGLYLYMLVVETFNRENIKLRAYLAIGWG

>RPRC009680

VSKRCLENGKWEGIGETGWTNYTTCYSPDLIQLFKKLFTGPYSHDAIKYQIAERTRTLEIYGFSISLAALFISLYIFSHFRVLKNNRTKIHKNLFAAMVAQAVIRLTLYVDQAIIRARKVQGIDNTPILCEASYVLLEYARTAMFMWMFIEGLYLHNVVSVRVFQETFHYKLYTSLGWGAPVIMTSAWAVTLAVQMKTECWWGYNLSIYFWILEGPRFAVVILNFLFLLNIIRVLVVKLRQSHTNEIEQVRKAVRAAVVLLPLLGITNLANMLGAPLDRQVWEFAAWSYATHFLTSFQGFFVAALYCFLNGEVSIQTI

>DmCG11144

MKQKNNNGTILVVVMVLSWSRVVDLKSPSNTHTQDSVSVSLPGDIILGGLFPVHEKGEGAPCGPKVYNRGVQRLEAMLYAIDRVNNDPNILPGITIGVHILDTCSRDTYALNQSLQFVRASLNNLDTSGYECADGSSPQLRKNASSGPVFGVIGGSYSSVSLQVANLLRLFHIPQVSPASTAKTLSDKTRFDLFARTVPPDTFQSVALVDILKNFNWSYVSTIHSEGSYGEYGIEALHKEATERNVCIAVAEKVPSAADDKVFDSIISKLQKKPNARGVVLFTRAEDARRILQAAKRANLSQPFHWIASDGWGKQQKLLEGLEDIAEGAITVELQSEIIADFDRYMMQLTPETNQRNPWFAEYWEDTFNCVLTSLSVKPDTSNSANSTDNKIGVKAKTECDDSYRLSEKVGYEQESKTQFVVDAVYAFAYALHNLHNDRCNTQSDQTTETRKHLQSESVWYRKISTDTKSQACPDMANYDGKEFYNNYLLNVSFIDLAGSEVKFDRQGDGLARYDILNYQRQENSSGYQYKVIGKWFNGLQLNSETVVWNKETEQPTSACSLPCEVGMIKKQQGDTCCWICDSCESFEYVYDEFTCKDCGPGLWPYADKLSCYALDIQYMKWNSLFALIPMAIAIFGIALTSIVIVLFAKNHDTPLVRASGRELSYTLLFGILVCYCNTFALIAKPTIGSCVLQRFGIGVGFSIIYSALLTKTNRISRIFHSASKSAQRLKYISPQSQVVITTSLIAIQVLITMIWMVVEPPGTRFYYPDRREVILKCKIQDMSFLFSQLYNMILITICTIYAIKTRKIPENFNESKFIGFTMYTTCIIWLAFVPIYFGTGNSYEVQTTTLCISISLSASVALVCLYSPKVYILVFHPDKNVRKLTMNSTVYRRSAAAVAQGAPTSSGYSRTHAPGTSALTGGAVGTNASSSTLPTQNSPHLDEASAQTNVAHKTNGEFLPEVGERVEPICHIVNK
