# Supplementary material for: Identification of Neuropeptides and Their Receptors in the Ectoparasitoid, Habrobracon hebetor
Source: Front Physiol. 2020 Oct 16;11:575655. doi: 10.3389/fphys.2020.575655 (PMC7596734; doi:10.3389/fphys.2020.575655)
Supplement: Supplementary file 8 [file Table_5.DOC]

>Dm_CG11144

MKQKNNNGTILVVVMVLSWSRVVDLKSPSNTHTQDSVSVSLPGDIILGGLFPVHEKGEGAPCGPKVYNRGVQRLEAMLYAIDRVNNDPNILPGITIGVHILDTCSRDTYALNQSLQFVRASLNNLDTSGYECADGSSPQLRKNASSGPVFGVIGGSYSSVSLQVANLLRLFHIPQVSPASTAKTLSDKTRFDLFARTVPPDTFQSVALVDILKNFNWSYVSTIHSEGSYGEYGIEALHKEATERNVCIAVAEKVPSAADDKVFDSIISKLQKKPNARGVVLFTRAEDARRILQAAKRANLSQPFHWIASDGWGKQQKLLEGLEDIAEGAITVELQSEIIADFDRYMMQLTPETNQRNPWFAEYWEDTFNCVLTSLSVKPDTSNSANSTDNKIGVKAKTECDDSYRLSEKVGYEQESKTQFVVDAVYAFAYALHNLHNDRCNTQSDQTTETRKHLQSESVWYRKISTDTKSQACPDMANYDGKEFYNNYLLNVSFIDLAGSEVKFDRQGDGLARYDILNYQRQENSSGYQYKVIGKWFNGLQLNSETVVWNKETEQPTSACSLPCEVGMIKKQQGDTCCWICDSCESFEYVYDEFTCKDCGPGLWPYADKLSCYALDIQYMKWNSLFALIPMAIAIFGIALTSIVIVLFAKNHDTPLVRASGRELSYTLLFGILVCYCNTFALIAKPTIGSCVLQRFGIGVGFSIIYSALLTKTNRISRIFHSASKSAQRLKYISPQSQVVITTSLIAIQVLITMIWMVVEPPGTRFYYPDRREVILKCKIQDMSFLFSQLYNMILITICTIYAIKTRKIPENFNESKFIGFTMYTTCIIWLAFVPIYFGTGNSYEVQTTTLCISISLSASVALVCLYSPKVYILVFHPDKNVRKLTMNSTVYRRSAAAVAQGAPTSSGYSRTHAPGTSALTGGAVGTNASSSTLPTQNSPHLDEASAQTNVAHKTNGEFLPEVGERVEPICHIVNK

>Hheb110870.1

MRGRRIVITAVTLITMVIGLAVMMYYFNQDKCPMGTFLCQNSTECKPQRYWCNGRIDCPEGDDESFTNCFDASGNWEWFFKKRPQPPVLICDPRDCPSPECSCQGCRANCKGFTEHLPNLSPNITSMTLFNASVKQWRAHNLERYAEIRSLYLASNKIETLEEEAFSKQAKLYWLVLSHNKIKQIKRGHFKGLSSLETLLADDNRIAKADFSDFAESTSLEMIDLANNSLTEMTLIFPNLPAMKELILDNNNIKSINRDFLAGMPQLRSLSIERNKLITIDSGVFRNLGELTELNLADNRIRVIEEHLFDPLVNLTQLMIGYNPIENLPVSSFNELTNLRSLGLEDVDMENFDTNAFVPFAQLEFVYFKKFHYCTTYAPNVRKCRPASDGVSSLSHLLGKPLLKVAVWSISSVTCLGNALVLWGRFTAKDENRVLSILIKNLAVSDMLMGVYLLIIGLVDVQFRDTYYQQASSWMSSWSCTLLGILAMISSEVSVLILSFMSVERFILIAQPLRGQQRALTPQAAAFSMTFIWICGIILAFIPVIHWRSSTRFYGLNGLCFPLHIDDPFLVGWEYSAFIFLGLNLVGLVTIGYVYAGMFTSIWRTRHNTPLFVGDSEFALRFFFIVLTDAACWAPIIALKIVAFWNYPVPPDLHAWVVIFVLPVNSAVNPLLYTFTTPKFRERLNEGWMGQLKNYVFDRRATQDSQASAGSSQDVVPNGFLSLAAIGKWAEFDRKPSTHKQN

>Bm LGR1

MFWRFNVVTIISILLFANQPVCCQLSEGVSVVLHQTNITRLTARALHAAGYHLHIIEHLSIMGAPKLEHVVVEDLIRMPRLKSFFITQAPLLHRVAPLPALPELRTFMITTSGLLEVPNLSHVHDSRKANTSLSYLQAIDLEGNHIKTIPSHALRVRADQVSLNYNLIEEVPKHAFKNAQISKLSFKGNTKLKRLDEHAFAGNLLLRQLDLSNTAITSLPTKGLEKLQILRIERTPSLKYIPSIYEFQQLEKAYLTHHFHCCAFKFPEIHNPARHKLYETQMAMMMQRCASIQKSQARKRRSLEPIRPVTDGAQTVTALEDDDASTMTASEEYENFEEYFSDSGSYEDGDQGEFHDIVNDTVVSISADCGNFNTRNRNVECTPASDALNPCEDVMGWSWLRASVWVVVAAAVVGNVAVLLVLLTNHTELTVPRFLMCNLAFSDLCTGLYLLMLAVVDLRSYGEFFNYAYNWQYGVGCKIAGFLSVFSGQLSVITLTIVTLERWFAITYAIYLERRISLSTAAKIMLGGWLFSSLMAGLPLLGVSDYSSTSICLPVESKDIGVVIYQGSLFLTNALAWVTIVVCYVQIYRSLGGGGENYGGRRAAAAAERRIANKMALLIGTDLLCWAPVAFFGVTALAGVPLVDVSHGKVLLVFFYPLNACANPFLYAILTKQYRRDFITLVARTGQCNWLVEKYKLSTTPPPTAHTNPSTPAQLMPLVDQKNHSQISKEFSDFKA

>DmCG7665 GPA2\GPB5

MEKHPSLSQRMGTTYRPRKGLKCLSFEFQCRLLLHHLLLTSLSGRHFVYATSAVGGALSANNCHDIHHGFDVYPNLTAVSLAQSTDTPLTATMPRSAWKCCCWNASNQAEEVECRCEGDGLNRVPQTLTLPIQRLTIASAGLPRLRHTGLKVYGSTLLDVAFTDCLQLELIQDGAFANLTLLRTIYITNAPKLTFLSKDVFLGISDTVDIIRIINSGLTRVPDLGHLPPHNILQMIDLDNNQITRIDSKSIKVKTAQLILTNNEISYVDDSAFFGSKIAKLSLKENKKLQMMHPNAFDGIIDITELDLSSTSLVGLPSAGLQNIEALYIQNTHTLKTIPSIYNFRNLQRAYLTHSFHCCAFQFPSRHDPQRHAQRMLEIEKWRKQCKSDSGTRKERSTLDNPFNMPEDFGSFGGTDDSATDITPITFASFDYMADDTMNKGTFHEKIILNPGDDSSAELCGNFTFRKPNIECYPMPNDLNPCEDVMGYQWLRISVWIVVALAVVGNVAVLTVILSIRPESTPVPRFLMCHLAFADLCLGLYLLLVACIDAHSMGEYFNFAYDWQYGLGCKVAGFLTVFASHLSVFTLTVITIERWLAITQAMYLNHRIKLRPAALIMLGGWIYSMLMSSLPLFGISNYSSTSICLPMENRDVYDTIYLIAILGSNGVAFSIIAVCYAQIYLSLGRETRQAHQNSPGELSVAKKMALLVFTNFACWSPIAFFGLTALAGYPLINVTKSKILLVFFYPLNSCADPYLYAILTSQYRQDLFTLLSKLGLCQQSALKYKDSLSGQATTRFTIHGSIQRHSSLTCKMQTVMGAETQKMLKNSEDYV

>NlA50

MQRRAVATALVTAVTLTHFTVKADQDGVEPHPTWPFAVKADQNGVEPHPTWPPPPQLPPMVQSHEPGCSCSDHRDPPRLLVKCKCRGDHLQRVPSDLQRGLHILSITRAAIEVLAADSFQPYRESLTDLSLVRLPNLRLIEPGVFNNLPHLRTIDIHSAPMLTIISDAVFQTHLPRLRIFRCTNTGLQQIPALRDLESKHQLHLVDLDSNRISLIPERSFYITSDQVSLNYNQIEEIEAFAFHNSTIATLSLKGNRNLHILSEDSFSGLNSLRKLDLSETAITFLPTLGLRGLDELRLQGTTSLKVFPSVYSFDSLKDVYLTYSCHCCAFRFPARHDPAGFRRHKEFVEKMIRDCSSSFSGDNVSYNDRANQQNISFGPANTSFWNNIEFTSGSSTTSSEETFHSIVAVSPNGQLQVRCGHMLGGGGSKGRLQGEGPRCFPAPDAFSPCEDLLGSGWKVRISAWLVSLFALVGNTCVLLVLLSSRFRMSVPKFLMCNLALADLCMGLYLLLIAIADARSQGAYFNYAIDWQNGIGCQAAGFLTVFASELSVFTLSVITSERWYTITYAIHLNKRLRLGSASRIMAAGWLYSIAMAALPLLGVSSYSITSICLPLQTSSAVETVYLATMLAVNGVAFGVVCVCYGLMYASIRGQGQGRSGRVRSDLSVAKRMALLVLTDLVCWAPVAFFGLTALAGHPLIDLPSAKLLLVSFYPLNACANPYLYALLTRQYRRDLLALFARYGICSKQAATRHRGGLAGGGGHGEARGTGGRGSRAGEGEMVLGHSPHHTGGLYSPP

>NlA49

MTCVLDAVTITRFVRLHWLMVSILESTASSAEATSVVRNWTSQQDVLLPPLIQSTDSGCTCKNLTGSDFTGCTCRGDDLKHIPDNLAPNLRTLTVTNAAIEVIEADSLISYRSTLADVSLNHLTRLRVIEQGVFNNLPALRTIVINYAPSLKSIPDAFFRTSLPELRIFRCTYTGLGVVPAMKYLGSKHPMSIIDLDNNQIEKLTRGSIQVTSDQLWLNYNKIREIEALAFFNSTLATLSLKGNRELKMLDSDAFTGLRSLRHLDLSDTSITFLPTSGLRGLEELKIQGTKSLKVFPSIYSFDSLKEVWLTYSCHCCAFHFPAQHDPLGYLRHQEFMLRIKEEQCTSQKNHHTELKKHHGMGAFDQDWGFSVTKVLPTGSFLEGQFHSIVSTNKKINALCGNISKNYEEVKCFPRPDAFNPCEDLMGNWVLRVAVWLVAILALLGNLAVLLVLLSSRFRMTVPKFLMCNLALADLCMGLYLLLIAIMDARSIGDYFNYAIDWQNGIGCKLAGFLTVFASELSIFTLTVITCERWYTITYAIHLNKRLRLSSAAQIMVLGWIYSITMATLPFFGISSYSITSICLPMENAKTSDLVYLVTLLVFNSLAFWVICACYSRMYVSIRGGQGQATADPLSCPDMRVAKRMALLVFTDFACWAPIAFFALTALAGLPLIDVPKTKILLVFFYPLNSCANPYLYALLTQQYRRDFFILLSR

>NlA48

MMSFQVSSIILHLALIANHAVAKDRIGMYWKCDCNFNNNSQDVPLKTDCHSFGVESKHLPHNKNATILWSLSNGAVTDIVKKCFLSHNTTLTDISFARLKFVDEFDKQAFTNLRLLESISITQSPALRYLPNGVFYPVSKNLRVLRMTHTGLEAVPTLPALSKKIMHLIDFESNKIRSIPSNSINIQTEQLVLAYNSITKVEGWAFNGSSIGKLSLRGNNRLVELSSDAFRGLKGLLDLDLSQTAITFLPVVGLEMLEMLKLQGTTTLKIIPSIHDFKSLQVAELTYSFHCCAFKYPARHNPARHELHVEYLQIAKERCGGNAPKRIANRFSRIKREADGSFGEIQNDGDEYGKFSHSVEDDSQQYPQSGSVSQENGDRNHSKLGGWTDAFEPPSTKKSESWKDGGVFHQTHASPTSFEAFCGNLTIRTNPVDCWPKPDALNPCEDIMGYDWLRISVWFVISTALFGNTAVLIVLIANRSDTTVPRFLMMNLAFSDLLMAVYLLLLAFTDIQSTGMYFNYAFDWQRGYGCRIAGTLTVFSSQLSIYTLTLITIERWFAIRHALYANVIDLKVAIQAMIVGWVYSIGIAVLPLFGISSYSTTSICLPMDVHNAASTIYVLTLLMVTGVAFVIICICYIQVYLSLGKNTRHCPESSVARKMMLLVGTNFACCAPIAFFSLTAVAGYPLIDVTRSKILLVFFYPINSCANPYLYAIMTAQYRKQLIQLMAKCGFCTECAQQYKMVYQPELEQKPRPSRATLLSTAHSTEDTVFNNNDSTRDTCNDISEREHQTEDNV

>Tc TcasGA2_TC009127

MIFCMFMIWLVSAVEGSDMLHKLPEETAFVSKVEPHETKPCTCECYNTTENFQIDVECTCSGKELQHIPDFLNKTLTKLVITDSDIKRIRKDELKPYRDTLKDVTLGNLPYLRVIEDGTFADIPNLRTLYISHAPQLKFLDGLLMGVTSKKFYSLRIVQTGLAEVPDLSYLPPENVMHLLDLDLNKIDKLKANSVKIQAEQVTLNYNEITVVEDLAFNGSQIGKLNLGGNKRLKKLEPNAFKGLQSLRELDLSSTSIENLPVVGLGEIETLRIEDTPSMKVIPSIYDLENLKVAKLTHPFHCCAFKYPEQHNPERHAQYEETTKRACKESTVAVDTGQPDGGNKRTKRWFQDDYDPSLNVLGYEHIGSRPLPPKDWTPNLDHFKGHPNKSDHSHLNPLRGRTIDHEEARPLDYEEDFGTFHAKSAEIPQQHKVYAVCGNLAMMRTPSVKCYPEANALNPCEDIMGFSWLRISVWFVVVLAVVGNLAVIVVVLFSGGELTVNRFLMCNLAFADFCMGLYLLLIASMDLHSVGTYFNFAFDWQYGFGCKLAGFLTVFSCHLSIFTLTIITLERWFAITYAIHLTRRIRLGAAAKTMLGGWMYSILVASLPLVGVSNYSSTSICLPMEVNRVADRAYLYSIILVNAIAFALIAFCYAQIYLSLGQETRHEMAIAKKFALLVFTDFATWAPISFFSVTALAGYPLIGVTKSKILLVFFYPINSCANPYLYAIMTAQYRKDFFILLS

>tetur01g15880

MWILLMLNGFTLSSGFTLTTKKPINSNHDSRHLPRGWFGAIPDDDASNHNNDDNDEVALVVENVDPSEEAKKDEKDHFFHDNQTPSSVPSDKSNIDGQSSDVSVDFSHSFSPTNPSIDSHFSTSLPTSSTKQTFSFENSSDDWVTTDLVYNNSDENENEVDQQNKVNEEDGFMIRGSSETQEEATAFDKHFCACKNETTHSWDHVEISCKCFGETIVDIPNHLTKGVAKLSMISTGIQTLVQNAFHSYSASLKDLYLESVKKLRWIEPGAFNNLPFLRTVYIKQAPLLKFIHDGVFFGSFPKFQVLKIIQSGLEVLPSMKYFETKGIISLLDFDSNRLKNISSASIRVRASNMILDYNSIEKVYSYAFLGSHIAKLSLKGNRRLVTIDDEAFVGLRNLAKLDLSETSITRLPTKGLEEVEVIKIVDTFTLKVFPSVFNFKNLKEAQLTYPYHCCAFKFPATHDPKEFASRVFEERRNCRQGTSGSTVLSTPSSTTTVATTSTTLLTSLHSLNLINNYELKSNGDLIGSYWKFIGDGIKHYTRRLNSLLSDNNFIGSINSQKSIESSSLLFSDNIISEDEQDENEDNNNENNEKDKIDDDNGARDKMRREMKNHLDDSVRFNSSSIINSNSLPNGEYITTTLSPLMDQSIFGKLIVSPSDAIDQKVNSSSIDILAGALNPQSPGFGQVQPSIFELPNEQLKHSEGDFIGQFHPTAASVLPEKPIHAFCGEYAQIFRDIKCSPEPDAFNPCEDVMGNLMLRIADWIISIAAVLGNLAVMVVLMSGRFKMNVSKFLMCNLAFADFCMGIYLLIIAIIDIHTVGVYFNYAIDWQHGLGCMITGFITVFASELSIITLTVITLERWYAITHAIHLHRRLKLNLAVKTMIGGWIYSLLMASLPLFGISGYSKTSICLPMENKDTVDIIYLITLLSFNALAFLLITACYGKMYHAVASQHGRITANDQTIAKRMALLALTDFACWAPIAFFGLTAVAGYPLINLTNSKILLVFFYPLNSCANPFLYAILTKQYRRDFFILISRYGLCRGSAARYKGTSNNCKHRRKGSCKKGRFCQVAYDCRTDRHKCKRCRHCLHSNTSSEENSSRSVIYSAIELYPDSSVSGDSVHEVLKMGHVICDDKQQSCSHHHHHYHHHYHHHKSNKESKKFPNHRPISQTTIIYSDDESNSSISLKKSPLIIKNGKELRRLPSSERDEVLRKMLGDSTLAPTTAGKNVVPSVWTTSNSCTSIHKSHKNCTSWIQRDSSSGEKSSAGNNNSSSNGTAIEIIKREKRCYCCKHCKRIPIENGSFVLSSHSNSAADDTEI

>PpLGR2

MLPRPRPSLVVGLLLLAAIGLEVVRAEVVLGCDVLGGPAARELVCRAAGIETLDRLPDLANVTRIDLTSNNLTNIPARGFQRYPYLEILLLRRNRIESIDVEAFDNMTRLALLELDDNNLTEFPRALARINSLEELSLSNNRIRVIEADSLQDARNLLSLDLRGNPIREVQPAAFQQLSRLRKLILSNLKELSEFPSLEGCRSLEFLRLDRARIERVPDELCRHAPKLKSLDLKSNRLTVVPNLKDCHDLRVLDLATNLIFSLEGKPFEGLGALHDLLIPNNLLESVPQDAFTGLTKLQVLDLESNSIDFVHPDAFEQIKQLEDLNLGNNIFPTLPIKGLSGLLHLKTFNNPALREFPAPELFPRVRTMVLSYAYHCCSFVSAEELEASYEADAGLSGGLETAGEEPVQESVLFPTDNDFDMSLWNQSLTDIWPQLHNLSDKFGSKINELWDNFGTDFTYPGNLPAYVEEYFDDQDGRSTSTPATSAHPHVQCLPQPGPFLPCRDLFDWWTLRCGVWVVFLLAMLGNGTVVFVLIFSRSKMDVPRFLVCNLAAADFFMGLYLGMLAVVDASTLGEFRKYAIPWQMSVGCQLAGFLGVLSSELSVYTLAVITLERNYAITHAMHLNKRLSLKHASCIMAAGWSFALGMAALPLLGVSDYRKFAICLPFETTSPSAMAYVVFLMMINGVAFLILMGCYLKMYCAIRGSQAWNSNDSRIAKRMALLVFTDFLCWSPIAFFSLTATFGLQLVSLEQAKVFAVFVLPLNSCCNPFLYAILTKQFKKDCVLICKAIEESRVTRGIGRCRHSSNFSNRLTPANTNSLVDRSAGSRDLHPRPHPHQQHHQINLYQQQQQQQQQQATCVCSNSARLILERGQSAGSTTTTAAAVRWWQAKIAWPCSKSQRQRRNVAGDPYAYRIAAEIQQKQHKRASSMSSSENYSSSRSDSWRQNHPCGIPLRLLDPKRRTSSWLITRKPSQDSNLSSSRNDSSGSGNTASTSTWRMTRSSTSLDRPKPRLTRQMAFQEPDSPGSPGRLAVRLLATIPSAAEMSEQCDEESAALAEKEEEEEPNADDDDDDGEEVKIRIKDAKES

>NvLGR2

MLLSLVGRLLLLWAAIGLQAVRTETPDCELLGGPAARELSCRAAGIEALDRLPELANLTRIDLTSNNLTNIPARGFRRYPHLEILLLRRNRIERIDPEAFDNMTSLALLELDDNKLTEFPRALARMSSLEELSLSNNRIRLIEADSLRDAKNLLSLDLRGNPISEVQPAAFQHLSRLRKLILSNLKELSEFPSLEGCRSLEFLRLDRARIERVPDELCRQAPKLKSLDLKSNRLSAVPNLKGCRDLRVLDLASNLISSLEGKPFEGLGALHDLLIPNNLLESVPQDAFTGLTKLQVLDLESNGIDFVHPDAFEEIKQLEDLNLGNNIFPSLPIKGLSGLLHLKTFNNPALREFPAPELFPRVRTMVLSYAYHCCSFVSAEELEAGVSGASEAAAGEELLQESVLFPTDNDFDMSLWNQSLTDIWPQLHNLSEKFGSKINELWDNFGTDFTYPGNLPAYVEEYFEEQEGRSPRPATQPARIQCLPQPGPFLPCRDLFDWWTLRCGVWLVFLLAMLGNGSVVFVLVFSRSKMDVPRFLVCNLAAADFFMGLYLGMLAVVDASTLGEFRKYAIPWQMSAGCQLAGFLGVLSSELSVYTLAVITLERNYAITHAMHLNKRLSLKHAGYIMSAGWTFALGMAALPLLGVSDYRKFAICLPFETSGPAAMAYVVFLVLVNGLAFLILMGCYLKMYCAIRGSQAWNSNDSRIAKRMALLVLTDLLCWSPIAFFSLTATFGLQLVSLEQAKVFAVFVLPLNSCCNPFLYAILTKQFKKDCVLICKAIEESRVTRSIGRCRHSSNFSNRLSPANTNSLVDRSAGSRDLNLLQHPHQPQVNAYQQQQATCVCGNSARLILERGQSASSTTTAAVRWWQANISWPCSKSQRQRRNLAGDPYAYRIAAEIQQKQHKRASSMSSSENYSSSRSDSWRQHHRCGIPLRLLDPKRRTSSWLISRKPSQDSNLSSSRNDSSGSGNTASTSTWRVTRSSSSLDRAKPRLTRQMAFQEPDSPGSPGRLAVRLLATIPSAAEMSEQCDEESSALAEKEEEQPDADDDEDDGEAVKIRIKDSDES

>BmLGR2

MQSAYSSSVGAPGAGPSLRRGTETILRRDRCHSKTTNHRPKASPLDEEVTQALKNQNSHLLGDLAADQTRLAVKYRRRTRNPHIGHVILQVSPGVWQRLTAAGRVHVDLQRIRVFDQSPLVQCSICLGYGHGRKHCNDTVPLCSHCGDPHLRADCPLSLAGTAPHCRNCRETKNSNAEHNAFSTECPVNLQRSKLATDELMLEAQKRKVLGTLLQEPYVGSVKKMKSYRGTRIFQNSAVGEGTVKAAIVIYQPELDIIQYPQLTTNNIVVVGVRTRAWNITLVSYYFEPDQNMGPYLEHLKRIELETGQSRLIIGGDCNAKSAWWGSPNEDQRGEQMCGFLEELGLQVLNTGDIPTFDTIRGGKRYSSHVDVTACSADILDLVDGWRVEEGLTGSDHNGITFGINVIKSKGISKYNLSSNSLRHSCLRRKMFGIRTPVKKSNGSEEKSELKELPCLSVRRSISDWPPTAEKEQQEQQRPKIPSPPKAVPQPLPPRPKTKASIAQEAKADLGRQATRVSDSPPNIVPSRFPSKTAEAKACLMKIRKQLNVVQRGFAQKLCRAYRTVSLNSALVLAGILPLDLRIREVATLYKAKRGVPQPVLGDREVERMAPMIKAPHPAEQVNLEFKSLIDEEQYEHHNNFEVRIFTDGSKLEGKVGAALSLWDRVSETKALKLTLPPFCTVYQAELLALQRAVREALNHSGTAFGIFSDSMSALQTVTNVSSPHPLAVETRDTIRRCMLQNKSVSLFWIKAHVGLEGNERADQLAKEAALRSKRKPDYDLCPISFVRRQIRLETLDEWDRRYRRFLRTFQANSAPSHVMYLLAAMKSPAPPRCVPGSPLHRVRDGVRVRRRMWAAALWAAGALWAAAAMCSGGGGGERLDCRSAGLQALPPLHHNLISLDVSNNNISSLPRDALLPATGLRDLNLSSNRLELVSAGSLSGAALARLWLDRCSLRRLPAHALRDLRRLHYLSAEDNLISELEGGAVGARGLRTLRLSRNLLRAVPTHALAPLHHLQTLSLSGNLITELSDSSLPPLPALHTLVLKRNRITHIDRPAYSGAPSLSRLRLEENLLSELPPAIQLLPVLQDLLLSGNRIEVVEAGILQQCRLLKRLDLRGNPLTRLHRHALQHLPHLRTLILSEARGLREVPSLNGSAQVRTLRVERARLTRLSTDLCRHAPLLQSLEMKSNYIDRVPDLHECSELHLLDLSSNEISAVQGSSFRGLHKLLDLLLARNRLRHIPSDTFIHTPELQRLNLEENQIEHIDMEAFVSISKLEDLNVGNNIFPWLPASGLQRLLHLKAHNNPNLRHFHPPDVFPRIQTLVLSYAYHCCEFMPLMEGGTVTEEETSEEDASTDLVIIPSQSIDAEAWLNATDVWSQLNVSAAGGSRWQAMLEDWESDLVEGVSEHRVEPSRRVQCLPLPGPFLRCVDLFDWWTLRCGVWAVFLLALLGNGTVVFVLICSRSRIDVPRFLVTNLAAADFFMGIYLGFLAVVDAGTLGEFRAHAIAWQMSGGCRLAGFLGVLSSELSVYTLAVITLERNYAITHAMHLNKRLSLRHAAFVMAAGWGFSLTAASLPLLGVSDYRKFAVCLPFETSSPVALGYVVSLLAINGVAFLVLLGCYLKMYCAIRGSQAWNSNDSRIAKRMALLVFTDFLCWSPIAFFALTAAFGLQLVSLEEAKVFTVFVLPLNSCCNPFLYAILTKQFKKDCAIVCKAIEESRVTRGIGRCRHSSNFSNRQTPANTNSLAERSSRGQHHAACACRRMLPAGAPPPPPPAARRARLLRWLRACGGIESIIPWHYGNITIRPATVCAGGSAPPPIPSLP

>Dm_CG8930 Bursicon

MAARCRWSWRLALCPLLLQLLLQLLLLPPSAMGHDETKENPAPDMQNSQEQEPYVHLQHLQQQQQQNPQTVQQLSQITVNRTSKSASVTPTGIRENVMLPSADPEKEAQILYEKSLQEYHGSQLSTASTATDVIAGKRTLHSICERWLQKHCHCTGSLEVLRLSCRGIGILAVPVNLPNEVVVLDLGNNNLTKLEANSFFMAPNLEELTLSDNSIINMDPNAFYGLAKLKRLSLQNCGLKSLPPQSFQGLAQLTSLQLNGNALVSLDGDCLGHLQKLRTLRLEGNLFYRIPTNALAGLRTLEALNLGSNLLTIINDEDFPRMPNLIVLLLKRNQIMKISAGALKNLTALKVLELDDNLISSLPEGLSKLSQLQELSITSNRLRWINDTELPRSMQMLDMRANPLSTISPGAFRGMSKLRKLILSDVRTLRSFPELEACHALEILKLDRAGIQEVPANLCRQTPRLKSLELKTNSLKRIPNLSSCRDLRLLDLSSNQIEKIQGKPFNGLKQLNDLLLSYNRIKALPQDAFQGIPKLQLLDLEGNEISYIHKEAFSGFTALEDLNLGNNIFPELPESGLRALLHLKTFNNPKLREFPPPDTFPRIQTLILSYAYHCCAFLPLVAMSSQKKTSQVQEAVLFPSDAEFDMTLWNNSMMNIWPQMHNLSKQLGASMHDPWETAINFNEEQLQTQTGGQIATSYMEEYFEEHDVSGPATGYGFGTGLFSGMSTEDFQPGSVQCLPMPGPFLPCADLFDWWTLRCGVWVVFLLSLLGNGTVVFVLLCSRSKMDVPRFLVCNLAAADFFMGIYLGILAIVDAATLGEFRMFAIPWQMSVLCQLSGFLAVLSSELSVYTLAVITLERNYAITHAIHLNKRLSLKQAGYIMSVGWVFALIMALMPLVGVSDYRKFAVCLPFETTTGPASLTYVISLMFINGCAFLTLMGCYLKMYWAIRGSQAWNTNDSRIAKRMALLVFTDFLCWSPIAFFSITAIFGLQLISLEQAKIFTVFVLPLNSCCNPFLYAIMTKQFKKDCVTLCKHFEESRVVGGGGPGGRGAVARTKRGDLPPPLLPAAAVAHPPGCRCLRMLPSEMPNWHKMEQTPSMWQRLRTFCCGENRRRRKQRRQPQQRRQRAYTAAAANPYQYQFAELRQQRQNRASSISSENFCSSRSSSWRHGPPSSAPVPPGNCSMPLKMLEPHAHPHGHGRRRHSAWLITRKTSQDSNLSSSRNDSSASATTASTSTFRLSRSSAGSSTPLPSIIAHNGKAQLDAVKPRLVRQEAVQEEEDSSPPRLGVRFLPTIPSAADSSVVMEDGDSANTGVASFLGMPLPGASSGFLIAPTTAATTSPPPVVLQPAKPPPDPNDAPL

>Tc Bursicon

DLTSNNITALNETSLSHYTHMEELTLSENKLESIHPKSFAKNVQLKRLLLQGCALSEIPVEVLRPLSKLQTLHLGNNEIWKLDGTTFQQVPALRSLRLDGNRLRGVPSEALSSLLHLEVLNIGNNLINALPPAAFPSLDKLVVLLMKRNQISEIAEEAFANLTSLKVLELDDNFLTEIPAAVTKLAKLQELSISGNRIKYIRGGLLQKTPALALLELKGNPLTGVDAHAFSFLPRLRKLQVLYVTSCAYISLRINTRTLSAFTTRNSISKSVLDDFMGVATLRKLILSEARELSTFPNLNGTTALEILRLDRAGISSVPSTLCTTCPRLKSLDLKSNKLKTIPDLNDCREMRVLDLASNHIRTLENRPFRGMYQMHDLLLAHNEIQYIPQDAFYNLSRLQVLNLEDNQISFIHPDAFLPISKIEDLNLGQNVFPHLPSAGLERLLHLKTFNNPNLREFPPPEEFPRIQTLVLAYAYHCCAFLPLIPSNPPPKAKDFIVFPDIEDIDMNMWNSSLNDYWPSQQNLSHKFGKKFETIWENLRSDFTYPGNFPSYMEEYAEEEAQRVAGGDGPPGKIQCLPLPGPFLPCQDLFDWWTLRCGVWIVFLCAMLGNGTVVFVLIFSRGKMDVPRFLVCNLAAADFFMGIYLGFLAVVDASTLGEFRMYAIPWQMSAGCQLSGFLGVLSSELSVYTLAVITLERNYAITHAMHLNKRLSLKHAGYIMICGWSFAIVMGLLPLFSVSDYRKFAVCLPFETKDAASLTYVVFLMFINGVAFLILMGCYLKMYCAIRGSQAWNSNDSRIAKRMALLVFTDFLCWSPIAFFSLTAAFGLQLISLEQAKVFTVFVLPLNSCCNPFLYAILTKQFKKDCVMICKAIEESRVTRGIGRCRHSSNFSNRQTPANTNSLADRSSRENQNHVPTCTCNVKLLGERSAPPPRTDRKTRTREWLLSKARWLLCVRRQPRHRPRSDQYTYQIAEIQQKQHKRASSVSSSENFSSSRSDSWRHNHHCGIPLRLLDPKRRASSWLVTRKTSQDSNLSSSRNDSSGSATTASTSTWRMSRSSASSEPNRIRAKPRLTRQSAIQDEADLPGSPGRLTVRFLTTIPSA

>NlA46

MVGVWWWIAVVLWGLIVGGGLGGAGGGHPPACVVQGGDALCRGAALTKIPTNLPQAIVKLDLTSNNITELGDNAFVELPQLEELILGDNKISKIHPRAFSANPRLKRLSLQNCGLAEVPWETFQPLRQLTSLQLDGNEISQVEAVSFAALGNSLHSLRLEGNRLTAPPTAALATLPHLEALNLGSNLISSIPVNSFPDLGNLIILLMKRNQISTIDEDAFSNLTALRVLELDDNLLTHIPIALTKLTSLQELSVSGNRIKFVAGGILQRAPSLSLLELKGNPLVGVDPLAFSALPKLRKLILSEARELREVPCLNGTTGLEILRLDRASITSIPSSLCHTCPRLKSLDFKSNKLTRVPDLGGCKDLRVLELSGNQINSLAGRPFVGLHQLHDLLLAHNVIPYLPPDAFTGLTRLQVLDLESNQISEIHPEAFLTFTQLEDLNLGNNAFSELPTAGLERLLHLKTFNNGHLRDFPPPSAFPRVQSLVLSYAYHCCAFLGPPGGGQAPLQVGGHAPGANRGTPLHESVLFPTDNEFDMTLWNSSLTDIWPQLQNLSKKFGTQINELWDNFGSDFTYPGNLPAYVEEYFEEQERTGGGGAVAEGGYGGAPGRIHCLPAPGPFLPCVDLFDWWTLRCGVWVVFLLAMLGNGTVVFVLVFSRSKIDVPRFLVCNLAAADFFMGVYLGMLAVVDASTLGEFRKWAIPWQMSAGCQLAGFLGVLSSELSVYTLAVITLERNYAITHAMHLNKRLSLKHAGYIMLCGWAFAVVLATLPLMGVSDYRKFATCLPFETTTGPWALAYVVFLMFVNGVAFLILTGCYLKMYCAIRGSQAWNSNDSRIAKRMALLVFTDLLCWAPIAFFSTTAAFGLQLVTLEQAKVFTVFVLPLNSCCNPFLYAILTKQFKKDCVLICKAIEESRVTRGIGRCRHSSNFSNRQTPANTNSLVDRSSRDGGQPCSCSAKLLSEAAAAASSPANARGANRQPLTTLQRLLRCLRRSTGSGRHRTRSDQYAYQIAEIQQKQHKRASSVSSSENFSSSRSDSWRQNHNHHHCGIPMRLLDPKRRASSWIVTRKTSQDSNLSSSRNDSSGSATTASTTLSRVSRSSASSGMSELTGISSRGGTKPRLTRQAAVTEVPPAAPPHYEGGPDPNRGLTVRFLATIPSEGGNTTTNDEASDSSTAILHDNGN

>tetur11g03260

MKQLFCQEQHQQPTRQPIDINGVTLLIFCIILSSVIIPIGCNTHQLKSGLSSIVTPKCPIECSCNGVYVDCSNRRLINFPGNISSATKKLNLSFNKLTSWINGTFRSYPDLTHLFLTSNMIEEIVIGMFDGLESLILLDLSNNNINQIDPDSLKPLVKLKELDLSSNALAEVPVAIRKLPLLKEIMINKNRIEVIDEDSFNSNNYLQLIEIKGNPLKSLDYSSFLNCTRLRKIVISDGHGLSRWPNLSNANSLQHISFDRTNIQTVSPLLCISSAALKSLIVKSSRLRYLPALNNCAKLELLDFSYNQLTMIGEKHFITQSQLTHLFLSHNWIQEIHGNSFTGLENLKMLDLSNNLIKSIHPNSFLPLLNLKDLNLGANKFSSLPTIGLRGVQQIKVHNNPYLISFPGPEFFPSIQVLALSYAYHCCSYNNLIDEASSSSSTNRINHFEEDIIWLHRDDVNMSIWNTSWPNIWSTRINSSSSPIWSPETDSFLMNISQFSEEYLDDYKTEINYDNIVFKYPIKCLPQPSPFTPCEDLFDWWTLRCGVWIVFLLALLGNGVVVVVLIFGRSKLDVPRFLVCNLAMADFLMGIYLGMLAVVDAATLGQYKVYALPWQRSFGCQIAGFLGVLSTELSVYTLAVITSERNYAITHAMHLNKRLSLKHAAYIMAVGWTFALIMALLPLFGVSDYRKFAVCLPFEITDIWSRTYVISLIVVNGLAFFILMGCYLRMYCAIRGSQAWNSNDSRIAKRMALLVFTDFLCWAPIAFFTLTAIAGYELITLAEAKVFTVFVLPLNSCANPFLYAIFTKQFKKDCILLCKRIEESRVTRGIGRGRNSSNFSNRHTPVNTNSVADKKSSGDSNQLVCKCGLASTAQSTVSINKLLPNGSCNGQPLFRKMATKWWQGRSVTKDLVGKLDATDSGSVQRRSNAPNNRLDSISSGNYSSRSDSWRHSYISPQLLTTKRSAAMVRRRSSWTASSSTSTRKLSTTRSSVSSDSSSAVFRHDLKGPARLPIADDYLLTFNSKQRSNSVVSSLVNFKPSSAKATFLCPECSRIGTMKQNEASTSINVNNLTTKYGRETEKIMMAAVVASSRDSNKFFNKLTELSRKDKKDSKEKHFVLSSGESIKNETTETSLQDSIATDTQMLSSRSNSISSPLTEIQIECTDDDDFNDDYDDLFSPERDYDNSYTLYPEPESSRASSNLSIRTQIPKGGTASPIGVGVSSDAELHVDESNNLSGSRELTGHENGSDQTADNNHEPMGSNLTQSRKVSSDNSLKSPFQVIPSLFKSFSSVGSQIFRSNKNANENQNFKENRKAGKSEGKQSQPAQRYLLSPDVPDCSIRVSKSCQTMVTSENFESTLRKSCTMAQLADKNWNDQIDTLTSPKLKNVSTNMDKDDTRDLHLDEDVPLIFFTHRDA

>DmCG34411

MCIAHLPITFTLAILLAIASNEGAQGVESATRTAIEAIRTGIGTKPETEIADATEAEAPVREVISLLGIIDGAESDILVPDADDKCPGGYFHCNTTAQCVPQRANCDGSVDCDDASDEVNCVNEVDAKYWDHLYRKQPFGRHDNLRIGECLWPNENFSCPCRGDEILCRFQQLTDIPERLPQHDLATLDLTGNNFETIHETFFSELPDVDSLVLKFCSIREIASHAFDRLADNPLRTLYMDDNKLPHLPEHFFPEGNQLSILILARNHLHHLKRSDFLNLQKLQELDLRGNRIGNFEAEVFARLPNLEVLYLNENHLKRLDPDRFPRTLLNLHTLSLAYNQIEDIAANTFPFPRLRYLFLAGNRLSHIRDETFCNLSNLQGLHLNENRIEGFDLEAFACLKNLSSLLLTGNRFQTLDSRVLKNLTSLDYIYFSWFHLCSAAMNVRVCDPHGDGISSKLHLLDNQILRGSVWVMASIAVVGNLLVLLGRYFYKSRSNVEHSLYLRHLAASDFLMGIYLTLIACADISFRGEYIKYEETWRHSGVCAFAGFLSTFSCQSSTLLLTLVTWDRLMSVTRPLKPRDTEKVRIVLRLLLLWGISFGLAAAPLLPNPYFGSHFYGNNGVCLSLHIHDPYAKGWEYSALLFILVNTLSLIFILFSYIRMLQAIRDSGGGMRSTHSGRENVVATRFAIIVTTDCACWLPIIVVKLAALSGCEISPDLYAWLAVLVLPVNSALNPVLYTLTTAAFKQQLRRYCHTLPSCSLVNNETRSQTQTAYESGLSVSLAHLGGGVGGGSGRKRMSHRQMSYL

>DmCG31096 LGR3

MVYGRSIAVGFCLMTVVLLLAAVIFYLSLGPCPAASFACDNGTLCVPRRQMCDSRNDCADSSDENPVECGLLYGSKEIADKIVRNAIEKKQQRLISAVSNASGADSTTSMVPRNQSLTLNMTCDIVTYPKACQCGQGTILYCGRYAKLRRFPRLSSEVTNLIIIRNNLTLRDNIFANFTRLQKLTLKYNNISRVPLGSFSGLFHLERLELSHNNVSHLPHGVFLGLHSLQWLFLVNNHLHHLPVEQLRFFRRLEWLVLSRNRLTLRNVQLPKIPTLYEVYLDFNRIEYIGEETFSQLDNLHLLDLQHNLITHIHGRAFANLTNMRDIRLVGNPIKELSGETFLHNTRLEALSLALMPIHISSSLMEPLNISFLNLTGIRYDHIDFEAINSMRNLTYIIYDRFFYCSMTPRVRMCKPSTDGVSSFQDLLSKPVLRYSAWVMATLTIAGNVLVLWGRFIYRDENVAVTMVIRNLALADMLMGFYLVTIGVQDYRYRNEYYKVVLDWITSWQCTLIGTLAVSSSEVSMLILAFMSLERFLLIADPFRGHRSIGNRVMWLALICIWITGVGLAVAPVLLWRTSTLPYYGSYSGTCFPLHIHEAFPMGWLYSAFVFLGVNLLLLVMIAMLYTALLISIWRTRSATPLTLLDCEFAVRFFFIVLTDFLCWVPIIVMKIWVFFNYNISDDIYAWLVVFVLPLNSAVNPLLYTFTTPKYRNQIFLRGWKKITSRKRAEAGNGNVATTTTGTATGSSQHPDDFTIFAKAAMRCH

>Tc TcasGA2_TC015772

LEQNKICPILGYFQCENTTICIPQQNNCDGKVDCPGGSDEVIGCDDRAKDDYWDHQFKKRPSALNDHLAHICNLSYNGSCVCRGRDLLCGHKNMVKIPGDLPADNITLLDFEGNNFGVLSGNVLEKVPLFVEKINFAHCNIDELKPHTFHQLTHIHDLHLDNNDLKTFPSDLFPESNNLRLLSVTHNHISRISSDAFRKLRALEELDLSGNKISEIKREVLAPLAKLRLLILRNNQIRYISNSTFPQLPLRKLSLIENRIDRLDPGAFANLTQLQELYLTNNRLVHLKNGTFFNLSQLLVLFLGDNFIKTIEVGVFIDVLNLTSLTLERNQFRTLDKKVLAPLTILQHIYFDRFELCESALHVRDCEPKGDGISSQYHLLDSIVLRTSVWIIAAIGCTGNLIVLLGRLLAPTNNVVHSLYLRNLALSDLLMGVYLFAIAIADQHYRGDYLRYQYSWRHSYVCNICGFLSTLSCESSVLILTLVTWDRFVSVTQPLARKQPSPKTAAFTLVVLWSIAAAVALAPLSEGYFGDEFYGNNGVCLPLHIHEPYAKGWEYSAAMFMLVNALALTFICYAYMRMINEIIASGVACRSTRQSQERDKVAQRFGIIVFTDCLCWVPIIVVKLVALAGYPIPKDLYAWLAIFILPINSALNPVLYTLTTTVFKKQ

>tetur15g00700

MTLNQVCTVISLIAIFATVFLLPLILLFGEDVANIERSYNEPHLDLFSDSDDNSSNFSINISTLDSDEDPETPICANIYDFPCHRSFDQDNQVICLNRDYQCNKVKDCPNGEDEEECNDIYGALDEHFTRQAKKWGTLIVSNKSQSNFIHANCTLADIYQSRCPCILNDSQRILCTNHDFTEIPSNLSTTITVLNLDNGKIDSIDSQVIAKYFNLTTVSLKNNRLTTIKSDSFSSLIQLKRLYLIGNEINHIDQDAFQKLINLKFLELHTNRLENIDAAQLFKGLTKLETLYLEFNQLKSVGKFPHLDSDLSNNQLTEIQDIFRNLVSLKVLNLSSNKLKRLPADAFHYNKRLIHLEVALNSIESIDVTAFHNLRDLRKLNLSYNPLTTLPRHVFKESNALQSLDLSGIEIRNIHPGHFNRTVALRHVYFSKFRYCMYAPRVRVCRPFSDGVSSVKHLLLYPILRMSVWIVAGITCAGNTLVLVWRSMAKKEHQSLSLLVKNLAIADLMMGIYLVVIGSYDQVFREKYNNYALQWMHSWKCNFCGFLATLSSELSIFIVLMITIERYRSITMTCRLVTLRWTASILLLIWTVSLAVSLFPILYWSDPEDPLYYASNGVCFPLHLEDPFMFGWQFSTFIFLGINLPAIVAIIGLYTRLFLVIKRDRQLTRPALLGKADHEDVILAFRFFCIVVTDCLCWVPIVSIKIFSFTYMYISPKVYAWLVVFILPINSALNPMIYTIAAPTGFRRKATKYLRQARRHLGPLLGWTSSGSMHQDRYSSSVPSNGTSSLDHTRSSNCSVLSMAAVNSITKNGLITNHVSSEKEIVKMRIK

>AmLGR

MRYKYIGTIGAFLIATTCLLSGLMYYFSQDTCPMGTFLCQNTTICLPQRNWCDQEIQCPYGDDEQNCCRFIVFIFHPSFSLPKRNPLQKNNDLDILLFAHDYHGVLDWFGSDRDSKAVKEFVCDSTDVPVSCKYVMCRATCQGYSDIPRNLSSQTTSITLYDSSIERVSAGAFAQYSEIRILYLDGSNIRELEKGAFANLTKLFWLALDNNEIGEFLPGHFTGLIKLESLKANKNRLTMADFSDLKGSVYLKLINLNENQLTSKGLRLSELPELSEISMQRNKIKGIEEDTFQNLEQLVELNLAFNEITTLPLNVFQPLKNLTSLQLGYNHLHNLPITVLSPLTRLRSLDLEGINLDSLEKDTFNVFEMLDFIYFKKFHYCATYTPNVKRCRPTSDGVSSLSHLLDKTLLRAAVWIISGVTCVGNVLVLWGRFTAKDENRVLTIIIRNLAVSDMLMGLYLFIVALTDIIFRDNYYQTASTWMSSWFCTFLGILAMTSLEVSVLILSFMSMERYVLIAAPLKGHRTMTPQTASTSVIIIWIIGITLALAPVIIWRSSTRFYGVNGMCFPLHIDDPFLIGWEYSAFIFLGINLMGLITIGFAYFGMFLSIWKTRHACSLSVGDSEFALRFFLIVLTDAACWAPIIVLKIRALLKYPIPADLHAWVVIFILPVNSAVNPLLYTFTTPKFREKLHDEWLRKMHNCVTRKSSQGISL

>Nl A47

MSKVMLMVICTCILHLRDAGVIGSSAVASKVVTQETDTQMVPFSCNTTVEAFQCDAATCIPTTSACNGIMECPNGQDESVSVCGCLPNEFRCRNSCVDLVKRCNRQKDCEEGEDEEDCKTFLCPITHFKCSNHYCIPLDSVCDFKDDCGDNSDELKCVRRKCWNPEFKCDSGECIRPGFLCDGSPDCKDGSDESPDTCHPRKFVKCGDGTRIHKYYWCDGWPDCTDNHADELNCEECLGEDDFKCPNGRCIRKANVCDSQCDCVNSFNNSLTYYYDKVCADEVNCTHSYKLHQGTGALECELGSTLGCSTPGSSHTKSRCIAPQFLCDGVNDCHNGDFLSDEFGCPYNQKHQKLDLDEVFQCRDNRSLPKRFRCDFNIDCLNGDDEEDCPEIEICHENEYRCENGQCIPKSGYCNLVFDCYDRSDESDCYNNLCGEGLLRCATGQCLPEDQWCDFFIDCLDASDETNCTIRECQSDEFKCDNGQCVSSTQKCYTSGNPRTGCADRSHLLNCRDWSCPESQFKCHQGPCLNMSLICDGNIDCPGSWADEDNCTFSCSNSAPQCECQDVMINCTGKGLSQLPDAEKEITSFHLGNNDLGPTLNNETFLNLDRLLYLDLSNNSITHLMPLMFCNLWRLSVLNLQNNKISILGNGSFYGLFGVSGLYLQGNQIQKIQTMAFIGLSSLTFLDLHGQRINQIEPSAFVGLRNLAGLDLDLSHNEIKYLEEGILQGMSRLLSLDLRYNKIKVIATQVFLNTPNLQKLITDEFRFCCLARHVEHCEPPPDEFSSCEDLMSNIVLRICVWVLAIVATLGNVLVIMWRSRYKHCNQVHSFLITNLAVGDLLMGSYLLLIALVDWHYRGVYFIHDSDWRSSQLCAFAGCISTFSSELSVFTLTVITLDRFLGIIFPFRVRRLEMARTRQLMAFGWLLAGILSALPLSRFTYFHNFYGRSGVCLALHITPDKPSGWEYSVLVFLVLNLLSFGFIAVGYLWMFLVARTTQKAVTKERQMNEAAMAWRMTMLVATDAACWVPIILLGIVSLAGYTVPPQVFAWVAVFVLPLNAAINPVLYTLSTAPFLTPARRGLLRFRRSCKMSLSADPRRTYSSGVATPMPIRRYSSACSNADLYAVTKRASAKWTRVDTTVSERGEVFPLSRLDNK

>LsGRL101

MATMSGTTIVCLIYLTTMLGNSQGVNLKIESPSPPTLCSVEGTFHCDDGMLQCVLMGSKCDGVSDCENGMDESVETCGCLQSEFQCNHTTCIDKILRCDRNDDCSNGLDERECDIYICPLGTHVKWHNHFCVPRDKQCDFLDDCGDNSDEKICERRECVATEFKCNNSQCVAFGNLCDGLVDCVDGSDEDQVACDSDKYFQCAEGSLIKKEFVCDGWVDCKLTFADELNCKLCDEDDFRCSDTRCIQKSNVCDGYCDCKTCDDEEVCANNTYGCPMDTKYMCRSIYGEPRCIDKDNVCNMINDCRDGNVGTDEYYCSNDSECKNFQAAMGFFYCPEERCLAKHLYCDLHPDCINGEDEQSCLAPPKCSQDEFQCHHGKCIPISKRCDSVHDCVDWSDEMNCENHQCAANMKSCLSGHCIEEHKWCNFHRECPDGSDEKDCDPRPVCEANQFRCKNGQCIDPLQVCVKGDKYDGCADQSHLINCSQHICLEGQFRCRKSFCINQTKVCDGTVDCLQGMWDENNCRYWCPHGQAICQCEGVTMDCTGQKLKEMPVQQMEEDLSKLMIGDNLLNLTSTTFSATYYDKVTYLDLSRNHLTEIPIYSFQNMWKLTHLNLADNNITSLKNGSLLGLSNLKQLHINGNKIETIEEDTFSSMIHLTVLDLSNQRLTHVYKNMFKGLKQITVLNISRNQINSIDNGAFNNLANVRLIDLSGNVIKDIGQKVFMGLPRLVELKTDSYRFCCLAPEGVKCSPKQDEFSSCEDLMSNHVLRVSIWVLGVIALVGNFVVIFWRVRDFRGGKVHSFLITNLAIGDFLMGVYLLIIATADTYYRGVYISHDENWKQSGLCQFAGFVSTFSSELSVLTLSTITLDRLICILFPLRRTRLGLRQAIIVMSCIWVLVFLLAVLPLLGFSYFENFYGRSGVCLALHVTPDRRPGWEYSVGVFILLNLLSFVLIASSYLWMFSVAKKTRSAVRTAESKNDNAMARRMTLIVMTDFCCWVPIIVLGFVSLAGARADDQVYAWIAVFVLPLNSATNPVIYTLSTAPFLGNVRKRANRFRKSFIHSFTGDTKHSYVDDGTTHSYCEKKSPYRQLELKRLRSLNSSPPMYYNTELHSDS

>PcGPRgph

MFFFFFFFFLETFQCSKSQFKCGNGFCISREHLCNFEDDCGDLTDETDCKYRECWPSEFRCDNQECIRPGMVCDNVPDCRDSSDETGCLSETCDDGKKIHKNTKCNGWPDCDDHKDELNCKLNLRNCDLSSGNYFQCPNTRCLRKSRICDGICDCLGETPCFDEINCDDYYTIVNGYQMCRVGSTISCRTTISGVTIERCISHEYICDGFNDCLNGNNNLSDEYGCVSGVDVWKNILEPNGEKFIFCGGLDQRKLSHSFICNYQIDCLNGEDELDCVWPNCTENEFQCDNGYCIPIRDRCNAQIDCSDKSDEINCTGFKCIVDIRDLDEKSNKLKQCDNGQCINSNFWCDFIQDCSDGSDENNCGNQKEFCTGNEFKCETSGQCIPKNEYCYKNENLRQGCADKSHLKGCANFTCPEGTFKCKMGPCLHTSLLCDGKFDCLDFWMDEKNCTHECFEFCTCIDITANCTGLGLQTIKNIDKMEDPYRKFYFANNNLSKVLNENFTYNIRFAILLDLSNNSICSITPGIFKDLRDLKTFVLQNNCITVLESQTFDGLSNLNGLHLEGNKIQTIKEKAFYGLSSLPTLNLKHQLIKHIAEGGFIGLRKLSNLDLSQNKIEILYHGTFQGLQKLTHLDLRGNPLRKLESDVFKRPELSIQVLLFDNFKFCCLAKHVPNCQPTSDEFSVCEDLMGNFVLRVCIWVLGIIASFGNLLVIGWRMNYKHKNKVHSFLITNLAVGDFLMGFYLLIIASVDAHYRGVYSVHDEEWRSSKLCSLAGFLSTLSSELSVFTLILITFESFLVIMFPFKVTRLQMSEIRWVMLGVWIAAVCLSGLPLLYKDYFKNFYGRTAVCLALCITIDKNSGWKYSAFIFIFLNFISLILIAMGYIWMYGAAKNTRLAVSRNLESKRMEQVMARRMIFIVATDAACWVPVILLGILSLNGVSVPSQVFAGIAVFVLPLNAAVNPIFYTISTATFLNPAKKNIKRFNNSFRFSPSGCVKNSTTSSIITHP

>ApGRL101

MNTENLQFIENMTLGCLQNEYRCTNKCIELVKRCDKIADCDGGEDEKDCTCDFKDDCGDGSDELQCKHRECWHGEFKCKNSECIRPGYLCDGEVNCADGSDEEYCETSDFIKCGGSHSVHSTFWCDGWPECADNHADELLCNASCAGNKFQCPNGRCINDANVCDGLCDCLPSIDGNCADELNCTKFYNKTDDVIVCTTGSTLSCWMPGGNPSRCIRQKYICDGQNDCFNGFSISDEFGCDKPNSHLNDEFFRCHDGRWLPFKHRCNYKAECLDGDDETDCEVSLPCGEEQFRCASGECVKSENRCDGRTDCWDKSDEIGCASVPCPGENWSRCKIGKQCVPMEKWCDYRVDCMDGSDEKNCDYRLCKADEFRCDSGQCIPLEYKCKKYREEQMGCVDKSHLRNCVDSKCAENEFKCHRGPCIHQSMVCDGQLDCDLTWDDEDNNCYFMCSDIASGCQCQDVHINCTGHGLDQFPYDVEKEITFFHLGGNNFSEGLHENTFEHLDRLVYLDLMNNSIKHLEPLVFSTLWRLKTLNLQHNEITILRNCSFLGLGQLTGLHLQGNNIYKLSSMAFQGLSSLTTLDLSHQNITDIETEAFVGLRSLKSLDLSHNSLTHIRDGTFRGMPQVVFLNLKNNQLRVIDKNVFFTMPLLETLFTDEFRFCCLARYVKQCDPLPDEFSSCEDLMSNIVLRVCIWILAVVAITANLLVIVFRAKYKHTNQVHSFLIVNLALGDFLMGSYLLVIAVVDWYYRGVYFIHDSDWRRSSMCNVAGFISTFSSELSVFTLTGYRFKSYT

>tetur15g00150

MNCQFNVSPVLLFNLFISLVNGKSRQQLKFQLLLMTFLIPISVVNCCDPGFFPCNDSDICIEQRLNCNNEPDCPDGSDEEHCEDNHKREYWDKLFRKRPDEDREKKNSTKCAMKNIPGECSCSMFNVFCEQQNLDRIPKIPMEARILDLSGNRVEKLRRTSFSYLPRLKTLILTSTEIQTIEKDAFANLPNLSSLFMSGNQLYTINNKIFSNNSNLVLLFLSHNPIQILKNQSFTGLSSLEELDLRNCRLSKFPKRVFEPLVKLQTLLLDGNLITSLPAKIFRSLNNLQVLSLTKNRINIIQDVNFIGLISLRSLSLSINNIEELKDNAFGNLTTLLKLDLRKNKLKLLESNVFSNLSDLESLDVRKNMIKKLPLNIFDNLQSLTHIYFDEFRLCSYALHVRVCEPRGDGISSFQHLLDNIVLRFSVWVVAFIACVGNVFVLIGRMLMHEPNQVHSFFIKNLAFADLLMGIYLFIIAYYDASFRGQYIKHEDAWRHSWQCNLSGFLSTLSSESSVFILTVITVDRYASVIYPLSQKRRTKGFAISCMVAIWTGAVVLALLPLLTDDLFGDEFYGNNGVCLALQIHDPFSKAWEYSTFLFCGLNSGAFIYITYAYINMSLTIASSRLGLRTTQQQQDRCITKRFGFIVATDCLCWMPIVIIKILALAGVPINDDLYAWVAVFLLPVNSALNPVLYTLTTKLFKQHFARIIAYGLQRSNSPGGDNNSGIHETRHSNGSTKEDLRSPSKDSFKNGFSFRKYCLSEQLSTFHREKSTDDEDGRTETF

>tetur09g05380

MEAYFPLVTLQAVVQLHIQGTTATALVEDASMINPMESEFDTNNETICLSGEFRCNDLCIEDVLRCDTKSDCSNGQDELNCESYICPSNHIKCDNHFCISIDRVCDFVDDCGDGSDEQNCTFRQCWYQEFRCKNEQCIQGYRVCDGKVDCVDGSDEAYCDEKSHFKDCGDGNRAHKSVWCDGYVNCPKNHADELNCINCSSDEFSCSNTRCIPSSSVCNTICDCVDNCEDEVDCKQFYHQVNGLKFCNTNVTFACPWDGPCVTIDSICNNISNCPYDDAYSGALDEYGCGITKEKCETFGNGFWCPEERCISASLKCNFIPECLNGEDELDCFSEPCDKFQCKNGQCIDFDKRCDSKIDCFDKSDELDCQNYPCPDDWVQCASGQCVKRSFWCDYTEDCLDGSDEAYCDYKSNPPECDPLTEYTCKNGQCIKLLNRCLVTQDRRDSCSDGSHLVNCSDYICPTNSIKCANSFCVHSSLVCDKKIDCLRSWTDEEGCPFVCSSTLCPCIDIVINCTNFGINYIPDDIETGISRIQVKDDEQEESKDEDTYQQGNNLGSNLTQSTFAKLDTKMVYIDLSNCSIQRLESGVFQSLNLLKVLVLSDNQITELTNENIFAGLISLRTIFLDGNGIKMIASYAFKGLSGIKSLDLANQQLTIIKRNTFNGMRSLVTLDLSNNQLFYLEEGSFTGLIKLTSLDLTGNKFSEMGTQVFTGLTNLKKLSTDEFRFCCLARHVQNCLPEPDEFSSCEDLLSNLVLRICIWILGVLSIVGNCMVIFWRTMHRYRAAVSSFLIANLAIGDLLMGVYLIIIGTVDFTYRGKYFIHDAHWRSSKMCQLAGFISTLSSELSVFTLTVITIDRFLRITFPLRFHRFKMTNARLVILATWVFTVILAGVPLLDIKYFDNFYGRSGVCLSLHITNQRPNGWEYSVFVFLVLNFISFTTIAIAYIWMFTVAKNTRSALKSSDIRLSSTMAKRIMLIVMTDFWCWMPIIALGVISLNGVKLPPQVFAWVAVFVLPLNAAMNPILYTISTLPFFKRTYSRSAQESKSSVVLKNGRSKSVIQRKNYRAKHHYFFNSKFTNQSEIKTV

>RPRC001663

MIKNNNPLLLTAYILVHRMMYVCDRTSAENLSKKFGTQINQLWDNFGTDFTYPGNLPAYVEEYFEEQEYNNKQTDPPPAKIQCLPTPGPFLPCVDLFDWWTLRCGVWIVFLLAMLGNGTVVFVLIFSRSKIDVPRFLVCNLAAADFFMGVYLGLVTLTLVDASTLGEFEMYAIPWQMSAGCQLAGFLGVLSSELSVYTLAVITLERNYAITHAMHLNKRLSLKHAGYIMLCGWSFATIMATLPLLGVSDYRKFATCLPFETSTTWSLTYVVFLMFINGVAFLIRWVAI

>RPRC014721

ETYTCPTTHFKCNNHYCIPIDLLCNFEDDCGDKSDESKDCNHRQCWNLEFRCENGECIRPGFVCDGRKDCKDGSDEALCAEDDFVMCRDGSRVHRSYWCDGWPDCPGNHADEWNCEVCDGPNDYKCPNGRCIKKANICDSQCDCAPHNGSLECADEMNCSKYYRSVHGKVDRCIASKYICDGSNDCHNGKYLSDEYGCQPSENQYSESTFRCLDNRTLPESLLCDYKNDCLDGDDENLCRALYQCDETMFTCNNSQCIDKNGRCNVTYECLDKSDELGCLDVPCPEGMVKCTYGGQCIPEKLLCDYFIDCPDESDEKNCPVTECNKLQFQCDNGQCVSIEHHCFISGNQRDGCADNSHLKNCKNFTCMRDHFKCRLGPCLNQSLLCNKKIDCQHTWEDEDNCTFTCSEKYPECPCKDIYINCTALGLESVPLDTEGEITWFHLGSNKLNASLTNETFSSLDRLLYLDLSNNSITGLPPMMFSNLWRLTVLNLQNNRIHTLVSSSFYGLASLKGLHLQGNGIRVVRMLAFYGLSSLRNLDLHDQNINLIEPDAFLGLRSLVGLDLSQNKIEYISDSTFRGMPHLLYLDISNNYIDVIDANAFRMATTLEKLVTDEFRFCCLARHVKSCLPPRDEFSSCEDLMSNMVLRICVWALAVIATVGNILVIACRARYKHCNQVHSFLITNLALGDLLMGSYLLLIAVVDWHYRGVYFIHDSSWRSSQLCSFAGFISTFSSELSVFTLTVITLDRFLVIIFPFRVRRLEMTRTRRLMAFGWIVAISISAVPLIHIDYFKNFYGRSGVCLALHITPDKPNGWEYSVFVFLFLNLVSFTIIAVGYLWMFLVARTTQHAVNKDRRTSESAMAWRMTLLVATDAACWVPIIILGIVSLAGYTVPPQVFAWVAVFVLPLNAAVNPVLYTLSTAPFLTPARHGFLTFRRSCKMSLSQDQRRTYTSGLNHYAGKSS

>TPAL_H9TUR5Q01C6ORS_3

TYKQWYQRRVAITAAGSIVSGDGPHCSNLKKIRVVQGYSMDIDNGEVSPIGYIMTIPSSKQEQIETLNNERIDCILKNDTNTFADGCPTVWDGILCWPNTPSNTLASLPCPVYFAGFSSHVSQNCLFLFFTFHHHHIICIFFKNIWAIYNGISITRLINKNFLFWENV

>Hheb00749 LGR

MERIDSRAFTNLSKLRVLELDDNLLSEIPEAIETISTLEDLSISNNRIDRIPANAFRGNKNLMSLDLRGNPIKVIDEGALQNHRKLRKLIISNVRSLADFPNLNGTRSLEVLRLDRASLKTVPEDLCKQCPKLKSLDLKSNYLESIPNLTECSDLKVFNMITSLANKPFSGQRFLHDLLLSNNNIKTIPEEAFAGLVRLQVLNLENNLIEYIHPAAFEAIKQLEDLNLGNNVFPDLPTKGLGNVLHLKTFNNPALREFPPPHLFPRVQTMILSYAYHCCPFLTAEYNEEVTKSSLQESVVFPDENDLELDPWNSSLTDSWSSSENSTNKFGAELKNLWDSYEGEYTYPGNVPTYIEDYFEDQEGRVSAPSGSGFPAHVQCLPQPGPFLPCQDLFDWWTLRCGVWVVFLLAMLGNGTVVFVIVFSRSKLDVPRFLVCNLAAADFFMGIYLGFLAVVDASTLGEFRKYAIPWQMSLGCQIAGFLGVLSSELSVYTLAVITLERNYAITHAMHLNKRLSLKHATYIMIVGWSFAFSMAILPLFGVSDYRKFAICLPFETNGIASLTYVVFLMLINGIAFLILMGCYLKMYCAIRGSQAWNSNDSRIAKRMALLVFTDFLCWSPIAFFSLTATFGLQLVTLEQAKVFAVFILPLNSCCNPFLYAILTKQFKKDCVLICKAIEESRVTRGIGRCRHSSNFSNRPTAANTNSLVDRSSRENHQPQCACNARLLEVNECRKTWWNSRILWSCRREMRSRPYNRSDAYAYQIAEIQQKQNKRASSMSSSENYSSSRSDSWRQAHHCGIPLRLLDPTRRASSWLITRKTSQDSNLSSSRNDSSGSATTASTSTWRMSRSSASLEFNSRTTPRPARSKPRLIRQFAIQEPEPPGSPSRLAVRLLATIPSAAEMSEQQEEESNADKE
