# Supplementary material for: Identification of Neuropeptides and Their Receptors in the Ectoparasitoid, Habrobracon hebetor
Source: Front Physiol. 2020 Oct 16;11:575655. doi: 10.3389/fphys.2020.575655 (PMC7596734; doi:10.3389/fphys.2020.575655)
Supplement: Supplementary file 9 [file Table_6.DOC]

>Hheb03289 NPLP

MQSIENQQLEQIGQNSIALIGNDRIKNLTIGYLTAIKGGLKDRQGLAISGAISMALDEINNNPRLLPNVQLVMRWSDTRGETVEATKAMIDMICDGVAAFFGPEGSCYVEAIVAQSRNIPMISYVPTFARTEPPDTQVTKSVIALLLHYGWNKFTIITEMAWISVAKSLENQAARNNLTVNHYKTVEDRHTCCEERLPCCQVSVWFQLIQETKNMTRIYIFLGTAMSLIDMMNSMQNQRLFDNGEYMVIYVDMMTYSQKEAQKYLWKPEHFDNLKNCLEPKDFLKRARSLMVVASTPPTQSYEEFTKKVRNYTSNEPFNFTVPDLLMNMKFDKYVSIHAAYLYDSVMLYAMALDQLIRERPESTIDELASNGTLIIETIIKKHTYLSVSGQTIKLDKSGDSEGNFSVLALKKEFFQRNNFSCDFQMKPVGQFQQGETLVYRPSESMDWPGKNKPEAEPGCGFLNEHCPKDDTHMRGVVVAGLLAVTLFCAAVITMSIYRRWKIEQEIEGLLWKIDPNDIAGYPVNDKIMASPSRLSLASAMSMESRVGGQVFAQTGQYHGVVVRIKELKFSKKKDVSRDVMKEMRALREIRHGNLNSFIGACVEPMRILLITEYCAKGSLYDIIENEDIKLDIMFIASLVHDLIKGMLYIHESSVLVCHGNLKSSNCIVTSRWVLQVSDFGLHDMRHCAESDSIGEHQYYRNLFWKAPELLRNPHASIKGTQEGDIYSFAIILFEIIGRKGPWGGVNLEPKAIGEPDKECPEYIVSTITDCWSESPELRPDFKSIRTRLKKMKAGRHRNIMDQMMDMMEKYADNLEELVSERTRLLFEEKQKTEDLLHRMLPEPVAHCLTNGIGVEPEAFDICTIYFSDIVGFTAMSAESTPFQVVNFLNDLYTVFDRIIKGYDVYKVETIGDAYMVVSGLPIKNGNRHAGEIASMSLDLLNAVKHYPIAHRPKDTLKLRIGIHTGPVVAGVVGLTMPRYCLFGDTVNTASRMESNGEPLRIHISAQCKEALDKIGGYIVEERGLVQMKGKGEVKTYWLVGANEKAIQKREVDVTDLPPLFCRPRRSPKLNPDSRQASLLTGLAAGSRRTSCVPRPSPDDSASQCGNSSPAPARQMRLSKLERNQLHLVDSKTTLDNVTVCEDAEIRAATIKQVLDGVFPENQQEAQRVLSTIASSSTTSDHPSAIIRESKSLDPFPSELHRDSPDVQRKEPKRSFRSLENVDNYGSKGDLRILNNNHPNGDIILKDNFVQDEEVNAPLLGDHNEAEMGIIKKWRSLDQVLVNNVSGDMVPEKKSSARNSIRSWLVNLFNGNTIRSSNVSIRRTVITGYDLQGERESIV

>Hheb03286

MQLINLERACLLVSLLSGFVGAETFTLGYITGSKRRINDLEYERPGIRISGAINLAVEEVNSGELGKLGHKLDFIVAETYGEEDTSILVTADLWTKNISGYIGPQETCVHEGKMAAAFNLPMISYFCTHHETSNKAEYPTFARTRPPDTQISKSVVAVLLAFNWTKVTFMYMNSTVTEFNSNWASVASTILELFQSSGITVTHERCWDEPYHTINVSNPFYRLVETSYKETRIYVILGNYDEHRGLLMALDEKKLLDNGEYWVVGVDIEQYKAKYPDEYLRGLLQDRTGPSLLRAYRSYFSIVASAPNISKKFTKIINEYRTKPPFNFKNPLQRFGGIVEVVPETAYLYDAVHLYARSLLSALKEGRDPRDGRKMVEMLHGVHYRSAMGYMVYMDRNGDAEGNYTLIALENHPDKGHGLYPIGHFVGKEESSNLPKLHLTRNITWLAGGPPVAEPACAHTGEIVGGIAGGILLILLAIVLVLYRNWRYEQELDSLLWKPVVRTSQVSLSSNPDADFRYSMIYTQIGFYRGRMFAIKKIRKKSIEITREMKKELKVMRDLRHDNLNAFIGACTEPPNICIVVEYCPRGSLKDIIENEDMKLDNMFMASLVGDIIRGMMYLHESVIRYHGNLNTSNCLVDARWVVKIADFGLREFKRDAECDSQDILKKYQSLLYRAPELLRSRQLPPSIRDFQKADTYSFSIVLYELHGRQGPFGPTHLSPADILAHLCNPTPSTPPLRPALDDLENCFDFVRDCLEECWSEDAELRPDFKTIRNKLRPLRKGMKQNIFDNMMAMMEKYANNLEALVDERTDQLTEEKKKTDALLYEMLPKYVAEQLKKGHRVEAEGFDCVTIYFSDIVGFTHMSAESTPLEVVNFLNDLYTCFDATIENYDVYKVETIGDAYMVVSGLPIRNGIQHAAEIASMSLCLLDKIKEFTIRHRPCEKLQLRIGIHSGPVCAGVVGLKMPRYCLFGDTVNTASRMESTGLPLKIHCSQETKELLDKIGGFYLEERGIVNMKGKGERLTYWLYGEHRGIRDARYQQIEQSLVPKSSLKNKTIRMSFLRCCSESPKRLRFASSDQLDGGNIENDEWSPCKGCMEGSKSASSSCPCVEKIEFGGSCFKDACKSVPASPNFLGKGKFCEILEGEADPLI

>Hheb087320.1

MARSGLHQDWWWLRMTIFICLLTGQYALPQRPIRTGLPIPMPPIDRDQSDVVIFLRNNCNTDKTSTISDEANEIGQFLSKYNEDGQLSVNILEKYLACGTKSWGLQVLIDVLGQASTKALVAALDVNICEVAEKLAHLWNKPLLTWTCPARMEDTNERTSTIRLSPSLPAVAQALGEIFLHFKWKSVSIISLDEEPWLSLDRAVIGVLRSVDIIPRHHVLVSRNARSEQIHQSLSPLESVIPRGIVLCLPIEDEKLMSRVMGELKAIRTSRTDNSLFLLVDPEGPGFFLPSPLTGEEEKSVESLADNSTGRPASMTSRWWHTQRQNFDQIPKRLGGRTTMNLLAFAPFDKRYDLLKIYNGTQEYSEPRMLDHLNESLTILTRDNSTLNTNNNKMFTYGLLDWRSSGSGANDGHWQPIAEVIESRDRLDVRELIENDLIDEETLDLALCLSGNDCGGHNNNVEGDEKETEEREDHQLPLKTSHIIVIILICFLLIVVLFIITLLVRRHIMTKRVAKGPFKIILTASDFVFPQPADNRRVDEGIETMLCCWLQQLQEFGGPEVEKPDLLQGSVGSLKPHLKASTGSLAWHTILKDPRARYNGDLVQLKELPCQSNFELKSKAMDVLVMIHGLRHENLNPFIGCLTEPARPCLVSEYCARGSLEDVLVQDEIKLDWSFRLSLLTDLVRGMKYLHSSPIRVHGYLTSRNCVIDARWVLKVADYGLPAFYEAQNIVPPPKSARDLLWTAPELLRHTGLRRKGTQPGDVYSFGIIMQEVVVRGEPFCMLALSPEDIIEKVKKPPPLIRPSVSKGAAPPEAINIMRQCWAEAADMRPDFNAVHDLFKKLNHGRKVNFVDTMFQMLEKYSNNLEELIRERTEQLDMEKKKTEQLLNRMLPSSVAEKLKLGMPVDPEEFAEVTIYFSDIVGFTTISAHSTPFQVVDLLNDLYTCFDDTINAYNVYKVETIGDAYMVVGGCPVRIQDHPSQIATMALDLLHQSGKFKVRHLPRTQLRLRIGLHTGPCCAGVVGLTMPRYCLFGDTVNTASRMESTGAPWRIHLSQATRDRLCQVGGYHIEYRGCTDVKGKGKMPTYWLLGKQGFDKQLPTPPPLGDDHGLEESLEGFKLEGIDDPKSECSEDSPTTTTTTEAHVLHKQATDERTTSSLKDEDDNSSGQSVCSFTTTCTSSKFSNAPTSLTRKVAVSVHDEREPTAAPSNLSAATGPLLGPAASTTSLSSISSSAFRPATSAPSRHRRVGHIDEDDLSTPYNHYRCLSPNEHHTKSSSRLLKRQFSLDRADEPVSIISESSMSLISTRPPPRLYKQNSAGAANDLEKIEEVPSLPPPHQTYRHAASMSLSVESLTLH

>PPU05511-RA NPLP

MRPGPPSTSRRRPPRRQQQHQTPQEQHLHRIHQPSLHRPTRLYRPSVGSSRQQQQQQRHRCGPLGVLLLLLLATCPGRAESPSSGQDVTSSSLPVQHQPRKPQNLTIGYLTAIKGELKDRQGLAISGAFSMALDEINNDPNILPNVKLVMRWNDTRGETVEATKAMVDMICEGVAAFFGPEGSCYVEAIVAQSRNIPMISYKCSDYKASNVNTFARTEPPDTQVTKSVIALLLHYNWNKFSIIAEKPWSTVAKSLQNQAAYNNLTINHFLSVEDRHVCCEDRLPCCQGGGWFPIIQDTKNMTRIYIFLGTPISLIEFMNAMQNQRLLDNGQYMVIYVDMMTYTPKEAMKYLWKPEHYDNLRDCQDPKDKDFLKRARSLMVVASTPPTQNYEEFTQKVRDYSSKEPFNFPVPERFLLDKFEKYVSIYAAYLYDSVKLYARALDQLLRDYPDQPLEEIASNGTLIIETIIKNHTYQSISGATIKLDSHGDSEGNFSVLALKKEPLHIQNFSCDFQMKPVGQFQQGDNLAYRPSEAVDWPGKNKPEAEPGCGFLNEHCPKDDTHMRSIVAAGVLAVLLFCAAVITMSIYRRWKIEQEIEGLLWKIDPSEIHGYPHLDNMMSSPSKLSLVSAMSYESRCGGQVFAQTGHYHGVVVRIKELKFSKKKDISRDVMKEMRILREIRHGNLNSFIGACVEPMRILLITDYCAKGSLYDIIENEDIKLDDMFIASLIHDLIKGMLYIHESSVLVCHGNLKSSNCVVTSRWVLQVSDFGLHDMRHCAESDSIGEHQYYRNLFWKAPELLRNLHAPIRGTQEGDIYSFAIILFEIIGRKGPYGGVNLEPKEIIDRVKRYPEDGEPPFRPNVDILSESEADCADYIVNTITDCWAESPELRPDFKTIRTRLKKMKAGKHRNIMDQMMDMMEKYANNLEDLVSERTRLLFEEKQKTEDLLHRMLPEPVANCLTNGIGVEPEAFDLVTIYFSDIVGFTAMSAESTPFQVVNFLNDLYTLFDRIIKGYDVYKVETIGDAYMVVSGLPIKNGNRHAGEIASMSLELLNAVKHHTIAHRPAETLKLRIGIHTGPVVAGVVGLTMPRYCLFGDTVNTASRMESNGEPLRIHISAQCKDALDKVGGYIVEERGLVQMKGKGEVKTYWLTGATEKAIQKREVDVNDLPPLFCRPRRSPKLNPDSRQASLLAGLGAGSRRQSSVPRPTPSDNTDSASQGGNSSPLQARPALTARKLERSPLYLTESSSKTTLDNMAIVREEAETRAANIKLVLDDLFPNNSDRHPVLAGNGLPSLQPSGAKFRKNARVLSTIAASSTSTSDHHGQPAAQCKALALRESRSLDPFPVDMLNSNSRKLEPAQLLTWKLQPRKSSFRSLENCDKCSPSRRGSKTSLANEKLLNNNYPNGNVIIVPAQSNGDDQRANSNVTSHQQQLVPFQHNDQDAAETPLLLGENCLLSSGELSMPVKRWRSLDQVAAPDNGSSGGGCVAVGGGLSDKKSTARNSIRSWLANLFNGNGLRSSDASLRRGVIPGYDMQSERESIV

>PPU05512-RA EH

MPVAQVERACLLVTTLLVHALLDGSGSVNAETFTLGYITGSKRRPGDWEYSRPGLQISGAITLAIDEVNSGELGRRGHRLNFNVAETYGDEERSILMTADLWTRNVSAYIGPQETCVHEGRMAAAFNLPMISYYCTNHETSNKKEFPTFARTRPPDTQISKSVVAVLKAFNWTKSSSTTAVAFMYMNASSFDPYERPTVAKTILSSLRSAGISVNSISSWEESYRVVENLINPFHKLVAETHVEARIYVILGNVEEHIGLLMALDKRKLLRTGEYWVVGVNTETYTDREPDVYVRGLMRNHTDTHSLRILQSYFSIIATAPIGYLNFTNKVNEYRQKPPFNFRNPLKNFKEEGSIQAVPETAYLYDAIHLYAQSLIKALDEGRDPRNGKEIISSLYGLHYRSAMGYMVYMDENGDPEGNYTLIALDNQAPKGPGLYPIGRFIGKENHTNLPKLHVIRSIPWVNGRPPVAEPYCGYHGEKCYSHTGEIVGGIAGGLLLVLLAISLMLYRNWKYEQELDSLLWKVNYKDIEIKETKDDSTSPSEQQLFKNNSKNPLQPHVRTSQASLSSNPDADFRYSMIYTQVGIYKGRIFAVKKVKKKSIEISREMKKELKIMRDLRHDNLNAFIGACTDPPNICIVVEYCARGSLKDILDNEDIKLDNMFMASLVGDIVRGMIYLHESIVKFHGSLTTSNCLVDSRWVVKLADFGLHEFKRDAELEPADVMKKYRGLLYKAPELLRPRAVEPTIRDFQKGDVYSFAIVLYELQGRHGPYGITELSAPDILKRIITIENPPFRPPLDQLENCFDFVRDCLLECWAENPEFRPDFKVIRNKLRPLRKGMKPNIFDNMMAMMEKYANNLEALVDERTDQLSEEKKKTDALLYEMLPRYVAEQLKRGHKVEAENFDCVTIYFSDIVGFTAMSAESTPLQVVDFLNDLYTCFDSTIENYDVYKVETIGDAYMVVSGLPIKNGIQHAGEIASMSLHLLDAIKQFSIRHRPLDKLQLRIGIHSGPVCAGVVGLKMPRYCLFGDTVNTASRMESTGSPLRIHCSTETKQLLDQLGGFSLAERGLVSMKGKGERLTYWLIGEEPSMREERNRERLARRAGLTGGTDNLLHPSSSGGYLDPLVPRSSLKNKSLARAAFLRCSSESPKRIRFASSDHLDQAAKGSRNNDGNKLESIVDGSPCNGRGLCSSGRSSCMEGTRSSSSSCPCVEHIGEAPLPLRLPAAVQLKLAPHLDCNDQLTNSAPILRAESPLAKDSEIDDEDNDGDTGWRVGGKVLRFGKKGLLRAACRSAPSSPKRGSTLLVSSPKRAVQSNEELDEWDVAPLIYCNSSSRRFE

>NV15866-RA

MPVAQGERACLLVTTLLVHALLDGSGSVNAETFTLGYITGSKRRPGDWEYSRPGLQISGAITLAIDEVNSGELGRRGHRLNFNVAETYGDEERSILMTADLWTRNVSAYIGPQETCVHEGRMAAAFNLPMISYYCTNHETSNKKEFPTFARTRPPDTQISKSVVAVLKAFNWTKVAFIYMNASSFDSYERPTVAKTILSSLRSAGISVNSISTWEESYRVVENLVNPFHKLVDETHVEARIYVILGNVEEHIGLLMALDKRKLLRTGEYWVVGVNTETYTDREPDVYVRGLMRNHTDTHSLGILQSYFSIIASAPIGYLNFTNKVNEYRQRPPFNFQNPLKNFKEEGSIQAVPETAYLYDAIHLYAQSILKALDEGRDPRNGKEIISSLYGLHYRSAMGYMVYMDENGDPEGNYTLIALDNQAPKGPGLYPIGRFIGKENRTNLPKLHVIRSIPWVNGRPPVAEPYCGYHGEKCYSHTGEIVGGIAGGLLLVLLAISLMLYRNWKYEQELDSLLWKVNYKDIEIKETKDDSTSPGEQQLFKNNSKNPLQPHVRTSQASLSSNPDADFRYSMIYTQVGIYKGRIFAVKKVKKKSIEITREMKKELKIMRDLRHDNLNAFIGACTDPPNICIVVEYCARGSLKDILENEDIKLDNMFMASLVGDIVRGMIYLHESVVKFHGSLTTSNCLVDSRWVVKLADFGLHEFKRDAELEPADVMKKYRGLLYKAPELLRPRAVEPTIRDFQKGDVYSFAIVLYELQGRHGPYGITELSAPDILKRVITVENPPFRPPLDQLENCFDFVRDCLLECWAENPDFRPDFKVIRNKLRPLRKGMKPNIFDNMMAMMEKYANNLEALVDERTDQLSEEKKKTDALLYEMLPRYVAEQLKRGHKVEAENFDCVTIYFSDIVGFTAMSAESTPLQVVDFLNDLYTCFDSTIENYDVYKVETIGDAYMVVSGLPIRNGIQHAGEIASMSLHLLDAIKQFSIRHRPLDKLQLRIGIHSGPVCAGVVGLKMPRYCLFGDTVNTASRMESTGSPLRIHCSTETKQLLDQLGGFSLAERGLVSMKGKGERLTYWLIGEEPSMREERNRERLARRAGLTGGTDNLLHPSSSGGYLDPLVPRSSLKNKSLARAAFLRCSSESPKRIRFASSDHLDQAAKGSRSNDGNKLESIVDGSPCNGRGLCSSGRSSCMEGTRSSSSSCPCVEHIGEAPLPLRLPAAVQLKLAPHLDCNDQLTNSAPILRAESPLAKDSEIDDEDNDGDTGWRVGGKVLRFGKKGLLRAACRSAPSSPKRGSALLVSSPKRAVQSNEELDEWDVAPLIYCNSSSRRFE

>NV15865-RA

MGEERKEALTGGGGGHAPRPAEHEPPQTSLHRPTRLYRPSVGSSRQQQQRHRCGVLGVILLLLLATCPGRAEPPSSGQDVTSSSLPVQRQPRKPQNLTIGYLTAIKGELKDRQGLAISGAFSMALDEINNDPNILPDVKLVMRWNDTRGETVEATKAMVDMICEGVAAFFGPEGSCYVEAIVAQSRNIPMISYKCSDYKASNVNTFARTEPPDTQVTKSVIALLLHYNWNKFSIIAEKPWSTVAKSLQNQAAYNNLTINHFLSVEDRHVCCEDRLPCCQGGGWFPIIQDTKNMTRIYIFLGTPISLIEFMNAMQNQRLLDNGQYMVIYVDMMTYTPKEAMKYLWKPEHYDNLRDCQDPKDKDFLKRARSLMVVASTPPTQNYEEFTQKVRDYSSKEPFNFPVPERFLLDKFEKYVSIYAAYLYDSVKLYARALDQLLRDYPDQPLEEIASNGTLIIETIIKNHTYQSISGATIKLDSHGDSEGNFSVLALQKEPLHIQNFSCDFQMKPVGQFQQGDNLVENVQSSLTRGAVDWPGKNKPEAEPGCGFLNEHCPKDDTHMRSIVAAGVLAVLLFCAAVITMSIYRRWKIEQEIEGLLWKIDPSEIHGYPHLDNMMSSPSKLSLVSAMSYESRCGGQVFAQTGHYHGVVVRIKELKFSKKKDISRDVMKEMRILREIRHGNLNSFIGACVEPMRILLITDYCAKGSLYDIIENEDIKLDDMFIASLIHDLIKGMLYIHESSVLVCHGNLKSSNCVVTSRWVLQVSDFGLHDMRHCAESDSIGEHQYYRNLFWKAPELLRNLHAPIRGTQEGDIYSFAIILFEIIGRKGPYGGVNLEPKEIIDRVKRYPEDGEPPFRPNVDILSESEADCADYIVNTITDCWAESPELRPDFKTIRTRLKKMKAGKHRNIMDQMMDMMEKYANNLEDLVSERTRLLFEEKQKTEDLLHRMLPEPVANCLTNGIGVEPEAFDLVTIYFSDIVGFTAMSAESTPFQVVNFLNDLYTLFDRIIKGYDVYKVETIGDAYMVVSGLPIKNGNRHAGEIASMSLELLNAVKHHTIAHRPSETLKLRIGIHTGPVVAGVVGLTMPRYCLFGDTVNTASRMESNGEPLRIHISAQCKDALDKIGGYVIEERGLVQMKGKGEVKTYWLTGATEKAIQKREVDVNDLPPLFCRPRRSPKLNPDSRQASLLGGLGAGSRRQSSVPRPTPSDNADSASQCGNSSPLQARPALTARKLERSPLYLTESSSKMTLDNMAMVREEAETRAANIKLVLDDLFPNNSDRHPVLAGNGLPSLQPSGAKFRKNARVLSTIAASSTSTSDHPGQPAAQCKALALRESRSLDPFPVDMLNLNSRKLEPTQLLTWKLQPRKSSFRSLENCDKCPTSRQGSKTSLANEKLLNNNYPNGNVIIVPAQSNGDDQRANSNITSHQQSHQQQLVPFQHNDQDAAETPLLLGENCLLSSGELSMPVKRWRSLDQVAAPDNGSSGGGCVAVGGGLADKKSSARNSIRSWLANLFNGNGLRSSDASLRRGVIPGYDMQSERESIV

>CG42636

MTRWPFNLLLLLSVAVRDCSNHRTVLTVGYLTALTGDLKTRQGLAISGALTMALDEVNKDPNLLPNVYLDLRWNDTKGDTVLATKAITEMICDGIATIFGPEGPCYVEAIVSQSRNIPMISYKCAEYRASAIPTFARTEPPDTQVVKSLLALLRYYAWNKFSILYEDVWSPVADLLKDQATKRNMTINHKQSFIDNRVKCCEQMLDCCRSGYWYQLVQNTMNRTRIYVFLGAANSLVDFMSSMETAGLFARGEYMVIFVDMMVYSEREAEKYLRRVDQITFMSNCHSTENFNQMARSLLVVASTPPTKDYIQFTKQVQKYSSKPPFNLEIPRLFVESNFSKFISIYAAYLYDSVKLYAWAVDKMLREETRVLTDDVIFEVASNGTRVIDTIIKNRTYMSITGSKIKIDQYGDSEGNFSVLAYKPHKWNNSNNMPCNYHMVPVAYFHQGEEHPEYKLINGSIDWPSGGEKPADEPMCGFANELCKKDDTHYTSTVAAVVLGVLLFCSGVITMSIYRKWKIELEIEGLLWKIDPNEIKGYSGNEIVSSPSKVSLMSAQSYGSRWTNQFVTSTGRLRGAVVRIKELKFPRKRDISREIMKEMRLLRELRHDNINSFIGASVEPTRILLVTDYCAKGSLYDIIENEDIKLDDLFIASLIHDLIKGMIYIHNSQLVYHGNLKSSNCVVTSRWMLQVTDFGLHELRQCAENESIGEHQHYRNQLWRAPELLRNHIHGSQKGDVYAFAIIMYEIFSRKGPFGQINFEPKEIVDYVKKLPLKGEDPFRPEVESIIEAESCPDYVLACIRDCWAEDPEERPEFSVIRNRLKKMRGGKTKNIMDQMMEMMEKYANNLEDIVTERTRLLCEEKMKTEDLLHRMLPQSVAEKLTMGQGVEPVSYDLVTIYFSDIVGFTAMSAESTPLQVVNFLNDLYTVFDRIIRGYDVYKVETIGDAYMVVSGLPIKNGDRHAGEIASMALELLHAVKQHRIAHRPNETLKLRIGMHTGPVVAGVVGLTMPRYCLFGDTVNTASRMESNGEALKIHISNKCKLALDKLGGGYITEKRGLVNMKGKGDVVTWWLTGANENAIQKKLVDMMDMPPPLFSRPRKSPKLNPDSRQPSIQAMHFCGTGSRRQSTVPRAMDGESTYSLQGSVRESPRMVSKRDRDRERPPINGLGAGHFVGGALLESAQASLSTLNHSETNETNCDMDGGSGGVSGSGSGLVRQPNALHKPLAMVRPHRIISAAQLPQLGDNDDDSADTLLRESRSLDPMPMQQLRKRHDRVKLPPSKLSKNNSRSLDTGVSLISGNPNGEVHSSQLDLDNEMTANPVDATDGYDDELGLLMRHDNGQLPALRYSGSFPNAQISIVPTGRSAGGGGGGREGGGSNCAKHLNNNCNGGVNVEDDLESPLLQRQASLSVPPEEMLAHNKRWHSLEHMDGPGGHGGNSVSYAADIDNRHPGDLDFFSGSSNQHHRSKAAGGSKLTNWMTNIFKGNGVRSGEARRVGILPSGVHGARTGFTDMAASAAARDRESIV

>CG42637

MTRWPFNLLLLLSVAVRDCSNHRTVLTVGYLTALTGDLKTRQGLAISGALTMALDEVNKDPNLLPNVYLDLRWNDTKGDTVLATKAITEMICDGIATIFGPEGPCYVEAIVSQSRNIPMISYKCAEYRASAIPTFARTEPPDTQVVKSLLALLRYYAWNKFSILYEDVWSPVADLLKDQATKRNMTINHKQSFIDNRVKCCEQMLDCCRSGYWYQLVQNTMNRTRIYVFLGAANSLVDFMSSMETAGLFARGEYMVIFVDMMVYSEREAEKYLRRVDQITFMSNCHSTENFNQMARSLLVVASTPPTKDYIQFTKQVQKYSSKPPFNLEIPRLFVESNFSKFISIYAAYLYDSVKLYAWAVDKMLREETRVLTDDVIFEVASNGTRVIDTIIKNRTYMSITGSKIKIDQYGDSEGNFSVLAYKPHKWNNSNNMPCNYHMVPVAYFHQGEEHPEYKLINGSIDWPSGGEKPADEPMCGFANELCKKDDTHYTSTVAAVVLGVLLFCSGVITMSIYRKWKIELEIEGLLWKIDPNEIKGYSGNEIVSSPSKVSLMSAQSYGSRWTNQFVTSTGRLRGAVVRIKELKFPRKRDISREIMKEMRLLRELRHDNINSFIGASVEPTRILLVTDYCAKGSLYDIIENEDIKLDDLFIASLIHDLIKGMIYIHNSQLVYHGNLKSSNCVVTSRWMLQVTDFGLHELRQCAENESIGEHQHYRNQLWRAPELLRNHIHGSQKGDVYAFAIIMYEIFSRKGPFGQINFEPKEIVDYVKKLPLKGEDPFRPEVESIIEAESCPDYVLACIRDCWAEDPEERPEFSVIRNRLKKMRGGKTKNIMDQMMEMMEKYANNLEDIVTERTRLLCEEKMKTEDLLHRMLPQSVAEKLTMGQGVEPVSYDLVTIYFSDIVGFTAMSAESTPLQVVNFLNDLYTVFDRIIRGYDVYKVETIGDAYMVVSGLPIKNGDRHAGEIASMALELLHAVKQHRIAHRPNETLKLRIGMHTGPVVAGVVGLTMPRYCLFGDTVNTASRMESNGEALKIHISNKCKLALDKLGGGYITEKRGLVNMKGKGDVVTWWLTGANENAIQKKLVDMMDMPPPLFSRPRKSPKLNPDSRQPSIQAMHFCGTGSRRQSTVPRAMDGESTYSLQGSVRESPRMVSKRDRDRERPPINGLGAGHFVGGALLESAQASLSTLNHSETNETNCDMDGGSGGVSGSGSGLVRQPNALHKPLAMVRPHRIISAAQLPQLGDNDDDSADTLLRESRSLDPMPMQQLRKRHDRVKLPPSKLSKNNSRSLDTGVSLISGNPNGEVHSSQLDLDNEMTANPVDATDGYDDELGLLMRHDNGQLPALRYSGSFPNAQISIVPTGRSAGGGGGGREGGGSNCAKHLNNNCNGGVNVEDDLESPLLQRQASLSVPPEEMLAHNKRWHSLEHMDGPGGHGGNSVSYAADIDNRHPGDLDFFSGSSNQHHRSKAAGGSKLTNWMTNIFKGNGVRSGEARRVGILPSGVHGARTGFTDMAASAAARDRESIV

>AGAP012163-PA

SGSYITKMPENSPHQKFRNNRTVLTLGYLTAVKGDLIEKQGLTISGALTMALDEINNDPELLPNVTLALRWNDTRGETVVATRVITEMICDGVAAFFGPEGTCQTEAIVSQSRDIPMISYRCSELQRSSPIPTFARTEPPDTQVTKSIISLLTYYGWRKFSIIHEQLWKNVATSLETQAKNNNLSVNHVEMVFDNYKCCQDDMDCCRSGYWYTVIQKTMNRTRIYVFLGNSNQLVDMMATMDGMQLFAKGEYLVISADMMTYSPKLSNKYLWRVEKPPNVKNCMDLPGDFERRSKSLLVVVASEPLPTFEAFTHKVREYTQKEPFFFKQPSLFHQFVKYVSIYAAYLYDSVKLYAWALDKLLKEEQQHRPLTSDVIRDVASNGTKIIQTIINNRTYHSVAGATIKIDDYGDSEGNFSVLALKREFYEEANFSCEFQMRPVAHFQMRQPQHLNDTNQHRNDEIPEFKLSKVGNAIDWPGSDRPMDEPSCGFMNEHCMKDDTHIMSMVVAGVLALILFCAGVITMSIYRKWKIELEIEGLLWKIDPGDIKGYFNTEIVSSPSKLSLASAQSFGSRCSNQVFTPTARFRSVVVRIKELKFSRRKDISREIMKEMRLLRDLRHDNINSFIGACVEPMRILLVTDYCAKGSLYDIIENEDIKLDDLFIASLVHDLIKAMIYIHSSALNYHGNLKSSNCVVTSRWMLQVTDFGLHDLRHCAENESIGEHQHYRNLFWKSPELLRQPSVYGTQKGDVYAFAIILFEIIGRRGPFGYTELEPKEIIDRVKALPEPGKDPFRPDIESVIENENVSDYVINCIRDCWDENADLRPDFPNIRNRLKRMRGGKSKNIMDQMMEMMEKYANNLEEIVQDRTRLLCEEKRKTEDLLHRMLPQPVAEKLTKGLGVEPVSYDSVTIYFSDIVGFTAMSAESTPLQVVNFLNDLYTVFDRIIKGYDVYKVETIGDAYMVVSGLPITNGNRHVGEIASMALELLQAVRSHRIAHRPNETLKLRIGIHTGPVVAGVVGLTMPRYCLFGDTVNTASRMESNGEALKIHISQQCKDALDTLGGYVIVERGLIAMKGKGEVMTYWLEAATEQAIQKIPVDVRDLPPPLFCRPRRSPKMTYDSRHPSIIGLPGGGMITTVSGMGSYMPGSRRQSCAHRVGGGADHESSYSLQGSMFGPMVPMMRADSSPPPRHRLDACTLDQRPMGPTAPTVTTLGSKIASSDNLKRALFGTNSPGRRLSHKLRSITSADDYKQLCSSMAANGGRSPEAALLRESRSLDPFPVTLEPRRKRLDALTKRVPRLAKTIAAPSRTGSQSFVSVPPAVSTISHESIADSTHHHHHHHHHHKYLHNNNCNGSIGNGAAEEQATCPLLTRQTSLTTPQEHEHHCPGLYTNNSSSKRWYSLEHVGVPDEDSCSKKSLTRSSLKSWLVGFIHGNGFKSSDSSLRKVGVLPVGVGGVTGFGELQPTPEKESMV

>AAEL008390-PA

MTRWPVLLLSLLSVAFAGAPNSDGTFQPSAAGSATILSQPLPPASPMLAAAQTALLGTSNQQQQQQQQQLSQSLFSLPQAQNPYASGATFIPKLTTSNSRHQKFKNNRTQLVVGYLTALKGSMKDKQGLAISGALTIALEEINDDPDLLPNMTLALRWYDTRGDTVTATRAITEMICDGVAAIFGPEGTCKTEAIVSQSRDIPMISYRCSELHRSTPIPTFARTEPPDTQVTKSIVSLLMYYGWKKFSIIHEEMWKNVAVSLENQAQRNNLTVNHVEEVIDYHKCCENNLDCCRSGYWYTIIQNTMNRTRIYVFLGNPNALVDMMTQMDAMQLFSKGEYLVIFVDMMTYSLKESKKYLWRLDRVSHHKNCNDMESFLQRGRSLLVVVASEPHPEFEQFTNKVREYNQKEPFSFRMPVLFKEYAKYVSIYAAYLYDSVKLYAWALDKLLKAEQAHRPLTSDVIYEVASNGTRIIETIIQNRTYKSIAGAMIRIDEFGDSEGNFSVLALKNEFYTEGNFSCDFQMKPVAHFQMRQFQQSNDSNQKRSDEIPEFKLSRAGTAIDWAGSERPMDEPSCGFMNEYCTKDESHVTSMVIAGILGLVLFCACVIMMSIYRKWKIELEIEGLLWKIDCNEIKGYFNTEIVSSPSKLSLASAQSFGSRCSNQVFTPTARFRGVVVRIKELKFSRRKDISREIMKEMRLLRDLRHDNINSFIGACVEPMRILLVTDYCAKGSLYDIIENEDIKLDELFIASLVHDLIKAMIYIHSSALNYHGNLKSSNCVVTSRWMLQVTDFGLHDLRHCAENESIGEHQHYRNLFWKSPELLRAEQRTEATYGSQKGDVYAFAIILFEIIGRRGPFGYSDLEPIRIIELVRAIPEDGQEPFRPDIKSVIENDCIPDYVINCITDCWDENPDSRPDFASIRNRLKRMRGGKSKNIMDQMMEMMEKYANNLEEIVQDRTRLLCEEKRKTEDLLHRMLPQPVAEKLTMGLGVEPVSYDSVTIYFSDIVGFTAMSAESTPLQVVNFLNDLYTVFDRIIKGYDVYKVETIGDAYMVVSGLPITNENRHVGEIASMALELLQAVRSHRISHRPNETLKLRIGIHTGPVVAGVVGLTMPRYCLFGDTVNTASRMESNGEALKIHISGQCKEALDRLGGYVVVERGLISMKGKGEVMTYWLEAATDKAIQKQPVDYRDLPPPLFCRPRRSPRLTYDSRHPSIIAIANYTAGSRRQSSALKDLESNYSLQGSSFEPSVRDSPRAYQRKLERIPLCINDDSQSTLEQQNAIGKTHSSDSLVKKVIFNNSSKKVRNMLRSIASTDDYHNICSSGTFLRESRSLDPFPSDVRKRLESIKLERKPRMGHKGSQSLDAGVSTISSESIDKDQSSKMIITTVPEDYSQGDGHKYLNNNCNGSIGNNEESHCPLLMRQTSLVTPGQEDSLSTHKRWYSLENVAVPDEDSCSKKSLTRGSIKSWLVGIIGFKTSDSSLRKVGVLPVGVPGVTGFGELQSTPEKESMV

>CG10738

MFAHPCPAPAGNYHSGLLHPLLLLLFLLAFSNFRPTHGEVFTLGYLTASQRRPGNLDYNRPGLTISGAISLAVEEVNAGRLRDRGHSLQFVVAETYGEEVVSIRQTAALWTQQVAAYIGPQETCVHEGRMAAAFNLPMISYYCTHRDPSNKADFPTFARTRPPDTQISKSVVALLLAFNWTQVSFLYLDDASGQYQPVAETILSTLTDAGVSIRDIRTWNTIYHHGFMDNPFEALVEQTYANTRIYLILGHYYEHVGLMVSLQRRGILSKGDYFVVGIDIEQYDPAKPEKYLRGLLLEDVEPLAVQAFQSYLGIVPTASVSFATFANEVNKYMERPPFNFPNPLGPFGGVKQISAEAAYLYDAVHLYAKALMEVLDSGGRPRNGSAIVAAIKGSRYRSAMGYHVYIDENGDAAGNYTVLARGSVRNGRNQTVLGLRPVGTFIHRNSSLSSISKALPNLKLFSPIDWVGGTRPAAAPRCGFGGEKCVNYTGEISAAIAGGALLLLSLVSLVLYRNWRYEQELDSLLWKIDFREVQIHENEREQQSQKQTRSTHPLIRTSQVSLSSNPDADFRYTTIFTPIGLYKGQLYAIKKVRKKSVDITREMKKELKLLRDARHDNICAFIGACTDPPNICIISEYCTRGSLKDILENEDVKLDNMFIASMVADIIRGVIYLHDSPIRFHGALCTSNCLVDSRWVVKLTDFGLFAFKQGIEDSSTDMQHMSAKCLKLLYRAPELLRQGPSSLVMGTQRGDAYSFGILLYEMHVRRGPFGETGLTPMQCLQKVLQPQDYLNPYRPSLQPLETAFDCVSECLRECWAERPEDRPDFKTIRTKLRPLRKGMRPNIFDNMMAMMEKYANNLEALVDDRTDQLQEEKKKTDALLHEMLPRCVADQLKKGHKVDPEHYEQVSIYFSDIVGFTAMSAECTPLQVVDFLNDLYTCFDSIIGHYDVYKVETIGDAYMVVSGLPLRNGDLHAAEIATMSLHLLSAVSEFKIRHRPTNRLLLRIGIHSGPVCAGVVGLKMPRYCLFGDTVNTASRMESSGVPLKIHCSWQCRQLLDRLGGYHFAERGVISMKGKGDQRTYWLLGEDEEARTRRTYERSQRRGSRALNKFIQGTIKQAQEQANEYGIRSSLKQKNLPRNSLTRSSSLESPKKLRFAAGSLLEHHRYHSDEALLEVDSYTGLRRSSGGSTQSRYEETTLSLTLSCQSIEIVGGQHNKRRPSSYPTANTPLLMNHVEV

>AGAP012161-PA

LQISGAITLAMTEVNDRYFSQHGHELRFEVAETYGEEVTSIRKTADLWTRDVIAYLGPQETCVHEGRMAAAFNLPMISYFCTHNETSNKKHFPTFARTRPPDLQISKSVVSLLLAYNWTQVSFLYRASDNGELDAVAETLKTTLRTASIRIRSVGTWTDIYHHGYSSNPFERLVEDTYEDTRIYLVLGYHYEHIGLLVSLRRRGLLDRGDYFVVGVDIDQYDAALPTKYMHGLLQTTPDPDAVEAFRHYLGIVPSAPVRFEEFAVKVSWSARDRSRLSRRQRAAVNKYLELPPFNYRNALIFFGGVKQIRAEAAYLYDAVHLYANALMQVLLSGGSPKNGSAIIEAIKGRAYISAMGYLVHIDENGDATGNYTILARKPVPSTGATNQYGLFPIGRFSSPTVDRIPVSGVCSEIRLFDTIDWVGSGPPVAEPRCGFRGEKCISYTGEITGGIAGGALLLLGVVSLVLYRNWRYEQELDSLLWKVDFREIQMHENEKETAGQKMTRVSVCVCRSHAYYSTHPLIRTSQVSLSSNPDLDFRYSTIFTPIGLYKGQLYAIKKVKKKSIDITREMKKELKLLRDMRHDNLNAFIGACTDPPNICIITDYCNRGSLKDVLENEDVKLDNMFTASMVADILRGMIYLHDSPLRFHGSLRTSNCLIDSRWVVKLSDFGLFAFKQGSEEVPDEKEKLEEKCQKLLYRAPELLRAGPTATVPGTPKGDVYSFGIVLYEIFTRRGPFGEIECTPMECLKRVLNPLDPNTPFRPAIQPLETSFDCVRDCLKECWAERPEDRPDFKTIRNRLRVLRKGMRPNIFDNMMAMMEKYANNLEQLVDERTDQLQEEKKKTEALLLEMLPRPVAEQLKRGHKVEAESYDLVTIYFSDIVGFTSMSAESTPLQVVDFLNDLYTCFDSIIGHYDVYKVETIGDAYMVVSGLPIRNGLIHAAEIASMSLQLLEAVAEFKVRHRPNDRLYLRIGIHSGPVCAGVVGLKMPRYCLFGDTVNTASRMESTGQPLKIHCSLQTKEILDSLGGYQFQERGLVPMKGKGDQRTFWLVGEDPDARARRTKERTERRGSRALNKYLGMLKSVTNLPGVRSSLKSRSLGLPRGSLPRSSSLESPKRLRFASGAMLEQHRYHRDDALMEVISDSSIRRSDYSISDGAEDITASCPCIEHLGTDPPADPAYLLTNGPTVTTPLLNNSIAT

>AAEL008387-PA

LQISGAITLAMEEVNDIYFSRHGHKLQFEVAETYGEEVTSIRKTADLWTKDVIGYIGPQETCIHEGRMAAAFNLPMISYFCTHNETSNKKHFPTFARTRPPDLQISKSVVSLLIAYNWTQVTFFYRTSENSEYDAVAETLKTTLKTNGVRIRSTRTWSTIYHHGYSPNPFDRLVEETFIDTRIYLVLGHHDEHIGLLVSLKRKGLLTAGEYFVVGVDLEQYDAALPKKYMHGLLQTAPDQDAVPALQHYLGVVPSAPVKFEEFAVKVSFSDRVIKLLIPCSSRTLGVNKYLERPPFNYRNPLVYFGGVKQIRAEAAYLYDAVHLYANALLRVLLAGESPKNGTAIIEAIKGRAYLSAMGYLIHIDENGDATGNYTILARKPIPSATSERDYGLFPIGRFSTPNADTIPVSCVVRDIKLFDAIDWVGYGPPVAEPYCGFQGEKCISYTGEISAAIAGGALLLLGVVSLVLYRNWRYEQELDSLLWKVDFKDIEMHENEKENAGQKMTRVSENDRAELNPSRNTHPLIRTSQVSLSSNPDTDFRYTTIFTPIGMYKGQLYAIKKVRKKSIDITREMKKELKMLRDMRHDNLNAFIGACTDPPNICIITEYCTRGSLKDVLENEDVKLDNMFTASMVADILRGMIYLHDSPLRYHGSLRTSNCLIDSRWVVKLSDFGLFGFKQGADIQEEKEKAESKCEKLLYRAPELLRAGPVSCVPGSPKGDVYSFGIVLYEIFTRKGPFGEIECTPAECLKRVLHPLDPNNPFRPAIQPLETSFDCVRECLRECWSEKPDDRPDFKTIRNKLRVLRKGMKPNIFDNMMAMMEKYATNLEQLVDERTDQLQEEKKKTEALLLEMLPRPVAEQLKRGHKVEAESYDLVTIYFSDIVGFTSMSAESTPLQVVDFLNDLYTCFDSIIGHYDVYKVETIGDAYMVVSGLPIRNGLTHAAEIASMSLQLLDAVSEFKIRHRPNDRLYLRIGIHSGPVCAGVVGLKMPRYCLFGDTVNTASRMESTGLQLKIHCSLQTKEILDSLGGYHFEDRGFIPMKGKGEQRTFWLVGEDPEARAKRTQERAERRGSRALNKYLGMLKQSNNSNGVRSSLKTRAVIPRSSLPRSSSLESPKRLRFASGSMLEQHRYHHDDALMEVVSDASTRKSDNSLAETEDFTSSCPCIDNLGLNTERFLAQSCPTVTTPLLTNTVAT

>CG33114 OGC1

MPGPCASAAAFSCILVLLLLGCQRSNPLAAGATVSSMRRLTDTINIGFLAEYSQMRVTLGGLPLAIEDVNKNPNLLPGKKLAFKPVDIGHKMSAYRVKPLRAMTQMREAGVTAFIGPDESCTTEALLASAWNTPMLSFKCSDPIVSNKSTFHTFARTLAPASKVSKSVISLLNAFHWNKFSIVVSSKPIWGSDVARAIQELAEARNFTISHFKYISDYIPTTKTLSQIDKIIEETYATTRIYVFIGEHIAMVDFVRGLQNRRLLESGDYIVVSVDDEIYDSNRRVNIMERIDICSKIKDYARKTPFLVPYHQRVFDNISVPIYGLHLYDSVMIYVRAITEVLRLGGDIYDGNLVMSHIFNRSYHSIQGFDVYIDSNGDAEGNYTVITLQNDVGSGASIGSLAKMSMQPVGFFAYDKNSVIPEFRYIKNDRPIQWLNGRPPLAEPLCGFHGELCPRKKLDWRYLVSGPLCALVVVVAIALLIKHYRYEQTLAGLLWKVDMKDVTVINLGEYNNPTNKNIFQICRQSILVVGEPNKRSFTNIALFRGNIVAMKKIHKKSVDITRSIRKELKLMREVRHENIINFIGASTDHGSVIIFTTYCARGSLEDVLANEDLHLDHMFISSLVSDILKGMIYLHDSEIISHGNLRSSNCLIDSRWVCQISDFGLHELKAGQEEPNKSELELKRALCMAPELLRDAYRPGRGSQKGDVYSFGILLYEMIGRKGPWGDTAYSKEEIIQFVKCPEMLQHGVFRPALTHTHLDIPDYIRKCLCQCWDEDPEVRPDIRLVRMHLKELQAGLKPNIFDNMLSIMEKYAYNLEGLVQERTNLLYEEKKKTDMLLYQMLPRPVAELLKRGDPVEAECFDCVTILFSDIVGFTELCTTSTPFEVVEMLNDWYTCCDSIISNYDVYKVETIGDAYMVVSGLPLQNGSRHAGEIASLALHLLETVGNLKIRHKPTETVQLRIGVHSGPCAAGVVGQKMPRYCLFGDTVNTASRMESTGDSMRIHISEATYQLLQVIGSYVCIERGLTSIKGKGDMRTYWLTKRQQPELTPDLISTVDTLDTYCSGPRESMEVSVHQYCSPASNNYRLGSCNCDTKCLYSRRSDDNVTNSHGTSEFPKVSEPAQVNCNQLCVCRLNSSQMFNNRGPRSAPSITFRL

>AGAP008848-PA OGC1

LSCRKMTEMRDNGIVVFIGPDETCTSEALVASAWNLPMISYKCADIAVSDKTVYSSFARTLPPATKVSKSVAALLLANNWHCFSIVASKHPAWSMEIAHAIELQAELNNLTVNHFRIYSDYIPSKIYELQEIVDNTYRNTRVYVFVGDHIEMVDFVRCLQNRKLLSTGDYIVISIDDEIYDPNMKRNIYQGNYSDFYQKYIGNSKDKHQQNRKRYNYKDQERLQEAFQSVLRISPLFPMNPKYRKLCHQFKLYSRKDPFRVPLPYNRHIFDEIQVPIYGAYLYDALIIYARAATEVLRDGGDVSDGRLIMRHIFNRSYHSIQGFDVYIDANGDAEGNFSVIALQKDDKVNNSLHMSMQPVALFAYGARNATGTGTTLPEFRYLNPNRPIMWLKGRPPLAEPVCGFYNEKCRPKAKDWRYITGALVVILFMAIFTIILFKHYRYEQTLACLLWKVDMKDVILITSPDALYNNELRKNLVSRIKYKELQINHFQRGEKVCQQSIMVNSAGDVNKRAYTTIGLYRGNIVAIKYLHKRTVDITRNIRKELKQMREIRHENLITFVGASIDHGTVSILTSYCARGSLVDVLSNEDLKLDHMFVSSLVSDIVKGLIYLHDSDVGSHGNLRSSKILIDSRWVAQIADFGLHEFKSCQEEPSKFEKELRRSLWKAPELLRDPNCPPKGTQKGDVYSFGIVLYEIIGRKGPWGDLNMSWQDIVARVMSPEEYGIFRPSLRGIDAPEYVIQLLHSCWEEDPEDRPDIRLVRVKLKPMQAGLKPNIFDNMLAIMEKYAYNLEGIVQERTNQLSEEKKKTESLLLRMLPKSVAESLKRGERVEAECFDCVTIFFSDLVGFTELCAQSTPFEVVEMLNDLYTCCDFIISSYDVYKVETIGDAYMVVSGLPLRNGDRHAGEIASLALHLLNSISNLEIRHRPGEFIQMRIGIHSGQCVAGVVGLKMPRYCLFGDTVNTASRMESNGEALKIHISSITYGLLKKLGGYKCEERGIIKVKGKGEMRTYWLLGEDDQKRMDR

>AAEL005330-PA OGC1

FQVILGALLLAIENINNDSTLLPGKRIKLKPVDIGAQKSLKAFPIRKMTEMRDEGIAAFIGPDETCTTEALVASAWNIPMISYKCADAAVSDKTVYSSFARTLPPASKVSKSVISLLAAYNWHCFSIVAGKHPAWSMEIAQAIKVQAEGNNLTVNHFREYSDYIPSKIYKLQNIVDETYRTTRIYVFVGDYIEMVDFVRCLQNRNLLSSGDYMVISIDDEVYDPSTKRNIYQVTWVQRYSFKDIQERLQEAFLSVLRISPLFPMNPHIKPVPIYAAHLYDAVIIYARAATEVVQAGGNLHDGRQIMRHILNRSYHSIQGFDVYIDENGDAEGNFTVIALQKDEKVNNSLNKSMQPVGMFVYSSNGTNLPEFKYLSSQRPIMWVKGRPPLAEPPCGFHGEKCRPITRDWRYITGIMMGTLFITIFAVILFKHYRYEQTLACLLWKVEMKDVTIMSSPDLMYGSDSKKKLTQVCRQSILANGADTTKRAFTNIGLYRGNIVAINYLHKRSVDITRTIRKELKQMRKLRHENLITFIGASVDHGVVAILTSYCARGSLADVLANEDLSLDHMFVSSLVSDIVKGLIYLHDSDVGSHGNLRSSKILIDSRWVAQISDFGLHEFKSGQDEPNKFEKELHRSLWKAPEILRNPNTPSRGTQKGDVYSFGIVLYEIVGMKGPWGEINLNYQEIIARVISPQNYGIFRPPLRGLEASDYVIQCLQACWEEEPDDRPDIRLVRVKLKPMQAGLKPNIFDNMLAIMEKYAYNLEGIVQERTNQLTEEKKKTESLLLRMLPRSVAESLMRGERVEAENFDCVTIFFSDLVGFTELCAQSNPFEVVEMLNDLYTCCDFIISSYDVYKVETIGDAYMVVSGLPIRNGDRHAGEIASLALHLLNSLSNLEIRHRPGEFIQIRIGIHSGQCVAGVVGLKMPRYCLFGDTVNTASRMESTGDAMKIHISSVTYNLLKKIGGYRFEERGVINVKGKGDMRTYWL

>AAEL006806-PA OGC2

MLKSKRALVLSVISLLSSWICSVQSENRTSRSYNNADSSHYKYNHSSIFNNISNSSSGSNVIEHNNPMATNDLHRNPPLWDSSNEKTAYQYAINDNDSNDAPTIASHLHFSSTLSAEHGSIVEKTVFNGGTFEGSHTPYHKKTVVRSASEDSYHEPHIKFAILLPEHGRSRDSRILSTVRPVIEMATNLVTGPNGVLHNLKIEIDYRDTQCSSTYGALGAFDILLKRKPDVFFGPICDYVIAPIARYNAVWGIPILTTGGLADAFTIKSPNYPTLTRMMGSYSDPGLALREMYRHFNWTIQAFIYHDNDEKRGMGHSDCSMAILSIFRVLNTTEYFSHSFDETETDYKGYLRILEETKRKARIVIMCASPSTIREIMLAAAELNMVDSGEYVFFNIDIFSSMAATKIPSWHMANDTEERNLKAKNAYTAMLQVVARQPEDEEYRRFSEEVKLLTKTKFNYTYAEDEPVSTFVTAFYDAVLLYAYALNDSIGLLGEQRALKQPINGTYLTHLMWGKSFKGITGNVTIDSNGDRISDYSLLDLNPETGMFEIVANYFHDGGLQFVEGKEIHWSGGRTKAPPDRPICGFDGSLCPDKSLPGYAILSLILGLCVICMGIASFMGYRHYKLEAEINSMTWKVQANEVLSCHSSQGHRGSLHMMAKRGSQVTFYSDELNSLPGDRQIYIQLGYYKGCKVAIKKININNLNLNRTMMLELKRMKDIQHDHLVRFYGACLDPSPDPFILTEYCPKGSLQDILENETIKLDWMFKISLMHDIVKGMAFLHSTELHSHGSLKSSNCVVDSRFVLKITDFGLHQLRGSLDDQDQESYAYWKKLLWTAPELLRDPHRDPAGTQKGDVYSFGIIVHEIVSRQGPFYTGDDEKSPKEIIKLVINGPDGYNPPFRPKVDEMYYEDVNNIMVKCWSEDPMERLDFTVLKTIIRKINKENESGNILDNLLQRMEQYANNLEALVEERTQDYFEEKRKCEELLYQLLPRSVAAQLIMGKSVIAETYDQVTIYFSDIVGFTSISAQSTPMQVVDLLNDLYTCFDSIVENFDVYKVETIGDAYMVVSGLPVRNGNLHAREISRLALALLAAVHKFTIRHRPNEQLKLRIGLHSGPCVAGVVGLKMPRYCLFGDTVNTASRMESNGEPLKIHISHTTKTLLDTFGTFEVTERGLVPMKGKGEMLTYWLNGERTVPMQTGFKSNKFSDNNTLESGPLALLNGTPPTGILNNNNNSNMYSLNSAVGGPNKKLNNVSYNFIKSSSTKNMLNSKGRRSLGGEDTIRSVTQPLLTQIN

>AGAP003283-PA OGC2

MSARANDDWGRAVVRCCVTLLLYVLASSVPGHERTSLVYGSIDSGTVAAQLATRNSTNYHLLSSDNGSNSSHPSDPSPLAAVTVTAPRDGNHFVNRSSRDTVGEAPYDDGCDHVRANTGVVNQHPRIADDTQGNYVKFAILLPKKPSKNRDIRILSTVLPVIEMATRVVTAPGGLLQNLRIEIDYRDTQCSSTYGALGAFDIFLKRKPDVFFGPICDYVIAPIARYSSVWGIPLITSGGLTEAFTLKAPHYRTLTRMMGNYHAFGLMMREIHRHYNWTIQAYLYHEFDEKSGRGFTDCSMAITSINRAIGGNETSSGTFDEETAKYADYLRLLRNIKKRARIVIMCASPSTIREIMLAAAELNMVNSGEYVFFNIEIFGSMTATKQPPWYAKNDTDERNQKAKEAFTALLQVVAREPEDEEYRQFSKEVKELTKTKYNHTYAEDEPVSTFVTAFYDAVLLYAYALNDSIAQLGVERALRQPINGTHLAQLMWGRSFKGITGNVTIDSNGDRISNYSLLDLNPETGLFEVVANYYYGGGLQFVEGKAIHWAGDRTKAPPDRPTCGFDGSLCPDNSLPGYAILSLVLGVCVVCMGIASIVGYRHYKLEAEINSMTWKVNPNDVLSCNPSRGHRGSFHSMVKRGSQATIYSEDLNSLPGDRQIYIHFGFYKGCKVAIKKINVQNLSLTRSLMLEFKRMKDIQHDHLVRFYGACLDLHPEPFILTEYCPKGSLQDILENETIKLDWMFKISLMHDIVKGMAFLHSTDLHSHGSLKSSNCVVDSRFVLKVTDFGLHQLRRSTDDADIESYAYWQKLLWTAPELLRDPQCPPAGSQKGDVYSFGIIIQEIVSRQGPFYLGTEEKSPKEIIKLVRDGPGILDAPFRPKVDESSYEDVNNIMIKCWSEEPTDRPDFSGLKTIIRKINKENESGNILDNLLQRMEQYANNLEALVDERTQDYFEEKRKCEELLYQLLPKSVAAQLIMGKSVIAETYDQVTIYFSDIVGFTSISAQSTPMQVVDLLNDLYTCFDSIVENFDVYKVETIGDAYMVVSGLPVRNGNLHAREISRMALRLLAAVYKFTIRHRPNEQLRLRIGLHSGPCVAGVVGLKMPRYCLFGDTVNTASRMESNGEALKIHVSHTTKLLLDTFGTFDLTERGLVPMKGKGEMLTYWLNGERTEAIAAAPLPNGLKVMSPAGTSPASIMASRNGPTLLATDSVPASDPQQQPLLLPTATSASQVNGTVASPPAGILINHHNHHHNNNHNNNNNNNNNSPCSINHNHSNSSPISCHNPSAPAKKLNSVSYSFAKAPGNSSSNNSNGAAAAAAAAASAKASKPKLPEVEALRGASVTKPLLT

>CG31183 OGC2

MPQAGQGLSLLLILCLLQAHLFPSAAWFYDAESVGGVASIGVGSDEHSFKTPETKNSHKSQSTELVQHFPSLPMPDHRRLGARRQLVFVALLPSVESDNKNDCIMPKVLPVLELAIRHVQRMGFVGGSHFDIQLISRDTFCSSKYGPIGFFEIYTQWPEVNAVFGLPCEYVLAPISRYADVWQVPVLTTGGNAKEFNKKSESYSTLTRLKGAQVNNLGNVVRAILNSFNWTRTALIYQNENAKVKGNSVCFLCLAAIHDTIEEHSVYQLGFDTSTWTKADITRMLKNVAMQSRIVIMCADPQSIRQIMLTAEELNMIDSGEYVFINIELFSRVQYLTSQPWYDKNDTDLNNERAQKAYTAMLTVTPKQPNDNEYTRVSNEIKAIAAEKYNYTFSDNEPISAFVTSFFDGVLLYANALNESIREDPTMLTRPINGTDMVRRMWNRSFTGITGNVTIDANGDRLSAYSLLDMNPTTGRFEIVAHFLHNRLEFEANKEIHWAGDREEAPPDRPICGYDGALCPDNSLPGYAILSIVLGTMVVVMAVCFFFGYRHYIAEAEINSMSWKVSLEDVMFRDAAERGLRGSFHSLVKQSSQLTLMSEDMVSINGDRQIFIPVGMFRKSKVAIKPVEVDNVQGLLTRSLMLELKRMKDLQHDHLVKFYGACLDQRRSFLLTEYCPKGSLQDILENEQFQLDWMFRLSLMHDIVRGMQFLHSSDIRSHGNLKSSNCVVDSRFVLKITDFGLHTLRRTRFDLESDGGNCNSHAYWSKLLWTAPELLRVEHNRPPEGTQKGDVYAFGIIVHEITTRQGPFYLGRCAYEKSPQEIIELVKGYNPHRMQKPFRPELEPNGDTKADINGIIRRCWAEDPAERPDFNTLKSMIRRFNKDNETGNIVDNLLKRMELYANNLEELVEERTQDYHEEKKKCEKLLYQLLPQSVAAQLISGQPVVAETFDQVTIYFSDIVGFTAISAESTPMQVVQFLNDLYTCFDSIVENFDVYKVETIGDAYMVVSGLPIRNGNQHAREIARLALALLEAVHNFRIHHRPEDRLKLRIGLHTGACVAGVVGLKMPRYCLFGDTVNTASRMESNGEALKIHISETTKEALDEFGTFVTTRRGFVPMKGKGEMLTYWLEGEVPRPNSLISPSKLMLTRRSSLKQPQRSQSHNLHKQYSELVVASPPPQLPPPAIALPPPPSPSKDSPNLRVKRKISSSSPKLNGGGFDYHKTENHYLDTAAAAQRNCDIYSSRSLRDMEETSDSWELAHFRLPLSGALKSHHHLNNNSHGYVHSNSSSNLSGILQPPPLGVQRTRLKPSPTISTLLTSARSEANASPGPETGSMGSGQLRIKFAEGDETPLLPTPPPLAAPPPIPQMVAASPQRSGSGQNIPADSSHYGFLVKSYKQQCPPVAGSAVTQPLLAKINS

>AAEL011318 OGC3

GERVAIKKVAKKKVYITSTLLWEIKQARDVSHENTVRFVGACIDLPRPTILILTEYCPKGSLKDVLENEAIQLDWNFRMSLIHDVVKGMAYLHNSDVGVHGKLRSCNCLIDGRFVLKISDFGLRTLTTPSEFVRDQNYYNSNLNALDLLPTTVIPGTPATQKGDVYSFAIILEEIVVRGGPYETARQVAAHESPPFRPFVGQRDCPPDLLDLMEKCWSDSPDDRPSFIQIKSTVKLIMKGFCENLMDDLLRRMEQYANNLESLVEEKTEQLSMEKRRTEELLYQVLPRPVAQQLLAGEMVQPEQFECVTIYFSDIVGFTALCAQSRPMEVVDFLNDLYSTFDRIIEFYDVYKVETIGDAYMVVSGLPERNGHDHAREIGLMSLAILDAVKSFTIKHKPEQQLKIRIGIHSGPVCAGVVGQKMPHYCLFGDTVNTASRMESTGHPLKIHVSEAAKQILDKFGTFRLELRGEVELKGKGICTTFWLLECSEPDPRPPTPMKNYIDNDVPFPILFPAIGK

>AGAP008691-PA OGC3

VYHVGVLMASHLDSPFDLERCGPAIDLALELVNQSLMKVHNVRLSKVQRSYATCSGSKSPGLAADLHFKHSVIAFIGPACAFALEPVAQLADYWNTPIITGMGDQPPSEGELSVTSGILGRLSNRWKNDSSGMFKDKSRYQTLTRMSYCQCRLKLVFSSIFRQFGWRHIALIIDRSDLFSLTVGKNLEYGLKDEELLKFVRELDGNDEEDIEAYLKDASMYARVIILSVRGSLVRKFMLSALALGMTRGEFTFLDVEIFQSSYWGDHYWELGDEDDFKARKSYEALLRVSLLQPTSPTYQYFAEKVRALAKQDYNYTFVEDEEVNFFIGAFFDGVYLLGMALNDTLNEGGDIRDGTAITRKMWGRDFEGITGHVRIDDNGDRDADYSILDLDPITGRFEVVAHYYGITREYSPVKGKKIHWPGGREGPPPDVPKCGFLGTSPACQGNDMIIRYGLVGFGIISAFAAAVTYILCKQMKLNSELNNMSWRVRPDEVLLEVGKMFGSKMGLQKLNYENFSLQQFGLNSGRVSIASGNSQLPAQLFTTIGIYKGERVAIKKVAKKKVYITSTLLWEIKQARDVSHENTVRFVGACIDLPRPTILILTEYCPKGSLKDVLENEAIQLDWNFRMSLIHDVVKGMAYLHNSDVGVHGKLRSCNCLIDGRFVLKISDFGLRTLTTPSEYLLPATVIPGTPATQKGDVYSFAIILEEIVVRGGPYETARQFLDPQAIVERVALHESPPFRPFVGQRDCPPDLLDLMEKCWSDSPDDRPTFSGIRSSVRLIMKGFCENLMDDLLRRMEQYANNLESLVEEKTEQLSMEKRRTEELLYQVLPRPVAQQLLAGEMVQPEQFECVTIYFSDIVGFTALCAQSRPMEVVDFLNDLYSTFDRIIGFYDVYKVETIGDAYMVVSGLPERNGHDHAREIGLMALAILDAVRSFTIKHKPEYQLKIRIGIHSGPVCAGVVGQKMPHYCLFGDTVNTASRMESTGHPLKIHVSEAAKQILDKFGTFRTELRGDVELKGKGIVTTYWLLECSEPDPR

>CG3216 OGC3

MHLLGISIHFFFLMYVNCFSAHPNPRRNDITWDDLNKDISLDSTTSLAGLNASDAGLEQRMYERSRESKSTQLSRYTEVGEMGSTMRVYNVGVLMASHLDSPFDLERCGPAVDLALDEINKVFLKPHNITLLKKKGSYPSCSGARAPGLAADMYFQDDVIAFIGPACAFALEPVARLAAYWNKPIITGMGDQPPSSEGELTVTSGILGRIHKWKNENTGMFKDKSKYPTLTRMSYCQCRLILVFASVIRQFNWNHVALLVDRSELFSWTVGKNLEYGLRQEGLLSFVKELNGNEEEVYENYLKDASMYARVVILSVRGVLVRKFMLAAHSLGMTNGEWVFLDVEIFQSEYWGDKGWEMKDEHDAKARKAYEALLRVSLLQPTSPKFQDFADNVRENALYDYNYTFGEGEEVNFFIGAFYDGVYLLGMALNETLTEGGDIRDGVNITRRMWNRTFEGITGHVRIDDNGDRDADYSILDLDPINGKFSVVAHYSGVHKQMKLSKELNNMSWRVRPDDVLIEMGGMFGSKGGLQRLDVENISLQQFGIHSGRASIASFTSLPPQVYTTIGQFKGERVAIKKVNVKKVDLTPQLLWEIKQARDVSHENTVRFVGACIDLPRPTVLILTEYCSRGSLKDVLENEAIELDWNFRMSLIHDIVKGMNYLHNSDVAAHGKLRSCNCLIDGRFVLKISDFGLRTLTTPSDFVRDQNYYLKLLWIAPELLPLTTIPGCCPATQRGDVYSFGIILEEIVNRGGPYQEARQQMDVHTILHKVRQCNGFRPLIRERECPPDLLELMEKCWADNQEERPTFSTIRSNIRTIMKGFCENLMDDLLNRMEQYANNLESLVEEKTRQLSLEKQRTEELLYQILPRPVAQQLMAGDLVEPEEFSSVTIYFSDIVGFTELCARSSPMDVVNFLNDLYSTFDRIIGFYDVYKVETIGDAYLVVSGLPEPNGDKHAREIALMALDILRAVSSFNLRHKPEYKIQIRIGMHSGSVCAGVVGKKMPHYCLFGDTVNTASRMESTGQPGKIHVSSATKAILDKFGTFQMEQRGDVELKGKGTVTTYWLNSTSEGEARPPTPQILTTDEVPFPLLFAGMGK

>AAEL007359-PA OGC4

MAHRLFNIPMEKEEFVEERMKIHEAAAVNGYDEEFVNKILRKHERKKHRQIATTLQPHKEEPLRISLPFYPKLTNPIQGILKQYGMQAAYKSGHTLKENYLLKNRLSKGPNKIVLSPSDFVFPVDMRRVDEGIEAMLCCWLQQLQEFGGPEVEKPDLLKGSIGSLKNLGLPPPPKTGSGSDTLVRHNTTVAGALELKARYNGDLVQLKEIPPSSGSSSSQELKTKAMDLLVMAHGLRHENINPLIGWLNEPTRTALVYEHCSRGSLQDVLIMDEIKLDWSFRLSLLTDLVRGMRYLHGSPIRVHGTLSSRNCVVDARWVLKITDYGMLNFYDAQGITPPSKSAKDLLWTAPEALRATKGYPKGGTQAADVYAFGIIMQEVVVRGEPFCMLSLSPEEIIAKIKKPPPLIRPSVSKGAAPPEAINIMRQCWAESPEMRPDFVMICERFKQLNHGRKVNFVDTMFQMLEKYSNNLEELIRERTEQLDMERKKTEQLLNRMLPSSVAERLKLGLAVEPEEFSEVTIYFSDIVGFTTISAHCTPVQVVDLLNDLYTCFDATINAYNVYKVETIGDAYMVVGGLPVRTPDHAEQIATMALDLLHQSGNFKVRHLPGVPLQLRIGLHTGPCCAGVVGLTMPRYCLFGDTVNTASRMESTGSSWRIHMSQQTCNLLEQAGGYIIEPRGPIEIKGKGKMHTYWLLGKKGFDKVLPTPPPIGLDIAILRKSLFQSEQHNQQQFVVASGSHNGSTANHSSSHSPSVAGESIDVKVEITPPVGCDNPHLAQSFSVDSNSSNANNCTLNMTDFQSKTALPSPQARKLSEIVTDTAFLSANSSFNRLNPSPTGTTSTRLIKRIEELMDLSSPYNYYKCLSPSESNLSQCMDNRYHGYQINRLDCSSKPGSTRFLRRQFSLDKDDVSPSMHTSQKATLDTVSSISIDREQLARMGTLSSIPSISSTTSISLGSKQQRAIHKQQSASVAQDLEKIEEIPISPQSFLLNHTTTSSNSTSSLTSDVNDKTRTRSSIQNGKELCLSIEALGLR

>AAEL012988-PA OGC4

MDEIKLDWSFRLSLLTDLVRGMRYLHGSPIRVHGTLSSRNCVVDARWVLKITDYGMLNFYDAQGITPPSKSAKDLLWTAPEALRATKGYPKGGTQAADVYAFGIIMQEVVVRGEPFCMLSLSPEEIIAKIKKPPPLIRPSVSKGAAPPEAINIMRQCWAESPEMRPDFVMICERFKQLNHGRKVNFVDTMFQMLEKYSNNLEELIRERTEQLDMERKKTEQLLNRMLPSSVAERLKLGLAVEPEEFSEVTIYFSDIVGFTTISAHCTPVQVVDLLNDLYTCFDATINAYNVYKVETIGDAYMVVGGLPVRTPDHAEQIATMALDLLHQSGNFKVRHLPGVPLQLRIGLHTGPCCAGVVGLTMPRYCLFGDTVNTASRMESTGSSWRIHMSQQTCNLLEQAGGYIIEPRGPIEIKGKGKMHTYWLLGKKGFDKVLPTPPPIGLDIAILRKSLFQSEQHNQQQFGVASGSHNGSTANHSSSHSPSVAGESIDVKVEITPPVGCDNPHLAQSFSVDSNSSNANNCTLNMTDFQSKTALPSPQARKLSEIVTDTAFLSANSSFNRLNPSPTGTTSTRLIKRIEELMDLSSPYNYYKCLSPSESNLSQCMDNRYHGYQINRLDCSSKPGSTRFLRRQFSLDKDDVSPSMHTSQKATLDTVSSMSIDREQLARMGTLSSIPSISSTTSISLGSKQQRAIHKQQSASVAQDLEKIEEIPISPQSFLLNHTTTSSNSTSSLTSDVNDKTRTRSSIQNGKELCLSIEALGLR

>AGAP002233-PA OGC4

MSSLRNYIIKTRLSKGPNKIVLSPSDFVFPVDMRRVDEGIEAMLCCWLQQLQEFGGPEVEKPDLLKGSIGSLKNLGLPAPTKPSSGSETLIRHSTAAIDHKARYNVSILSFPVSGDLVQLKEIPSSSASHELKTKAMDLLVMAHGLRHENINPLIGWLNEPSRTALVFEHCSRGSLQDVLIMDEIKLDWSFRLSLLTDLVRGMRYLHASPLRVHGSLSSRNCVVDARWVLKITDYGMLSFYEAQGIAPAPRNAKELLWTAPEALRDSRTYPKAGTQPADVYAFGIIMQEVVVRGEPYCMLSLTPDEIIAKIKKPPPLIRPSVSKGAAPPEAINIMRQCWAENPEMRPDFATICERFKQLNHGRKVNFVDTMFQMLEKYSNNLEELIRERTELLDIERKKTEQLLNRMLPSSVAEKLKLGLAVEPEEFAEVTIYFSDIVGFTTIAAHCTPVQVVDLLNDLYTCFDATINAYNVYKVETIGDAYMVVSGLPVRTPDHAEQIATMALDLLSQSGHFKVRHLPGVPLQLRIGLHTGPCCAGVVGLTMPRYCLFGDTVNTASRMESTGSSWRIHMSQQTCNLLEKAGGYVIEPRGPIEIKGKGKMHTYWLLGKKGFDKALPPPPPIGLDVAILKHSLFQSQQDHGAKINNASIQSNTCSTANHSSSHSPSVAGESIDVKVEITPPVPPSLDPGQQQQQPGQDTAAVLSTSFSIESSSSNNTFTLNLGEFGQPKASPLPSPQARKLSEVIADGSFLSAGATFDRLNPSPSSASASSTRLFKRIEELIDLSSPYNYYKCLSPSENNLSQCTESRYGSYQLRSDSCSSKPGSTRFLRRQFSLDKDDVGPSSGGHGVATLSAKATLDTISSISVDREQLARMGTLTSIPSISSTNSATLPGGGQKQRSLAMHRQQSASVAQDLEKIEEIPLSPQSFIINHTGGGGGGGGGGSSSTSSLQSEVNERGRSFRNGASTAPNGHSGKELRLSVEALGLR

>CG34357 OGC4

MKLTTCQIAKVFPLLTAFFFLNSPSAASLSFGLSPTFASASASSSLSSSSSSSSHFHSEKPLHSDVLVLRAAKPSRAIATSADAATRSVPASAAETTLGSKSIWPTAIQTKSPRNAREAGRHKRQMQKPAIQQQPLQQQQQRINPPQQKRQQQQQQQQQVQHQAQRVGVLIPPSLHVDLTHVQQGFQNFLDFFQLHLFNVTVDFLRDVDLSGFIKLLELPKYTSVIKTLNAGIMVASHVDVDQNSTIYIGTPSQPRQQHRLHSKQEQEPHRHHHEDSKAKQFPAGEEESHSSGETEENHWNDARIFSGHCHQLAEQLALDFNKTVVLWPCPRMKISSNFLPSFEAISLAVQSISTKLNWSQVDIYIGDDNWGLGLAIAANLHVPYRIEIGRTIRDLHSQDKPGKAIIITAPLNDASTILLLSTIELDWTEQARKDGQGSRPGRQHTKILLIDMAASSLDTQHGFYKYLSRMGGSQSDAFARNTGSPAEVVSSNLLVLTLLNDRHRLFLNAAGLMATMQNFRAPLNGNYKDPPPSGHRNASLHKLYPYRNLLPLYDTIVTTTTQSDTSETSPQFDFVVLDIVRDAPVATYKWRPLLILESDPGSRGGGSVVEGGGSYISHSIHPGYDEWLLVSSVLLWQCGAICWTIAAICVCLLVIMIAGIVAGGIAMRNYFLRKRLSKGPNKIVLSASDFVFPVDSRRVDEGIEAMLCCWLQQLQEFGGPEVDKPDLLKGSIGSLKNLGFVIPGAAAPGSAGAITTTANGKSGSSATGSLARHNPAHLDMRARYNGDLVQLKEVNINGSAELRTKAMDLLVMAHGLRHENINPLIGWLSDPNRTAMVFDYCSRGSLQDVLIMDEIKLDWSFRLSLLTDLVRGMRYLHTSPLRVHGALTSRNCVVDARWVLKITDYGLNSFYESQGLPPRTRSAKELLWTAPELLRNMKLHQHHHQHGRIQLGTQLGDVYSFGIIMQEVVVRGEPYCMLSLSPEEIIVKIKKPPPLIRPSVSKGAAPPEAINIMRQCWAEQPDMRPDFNSVYERFKMLNHGRKVNFVDTMFQMLEKYSNNLEELIRERTEQLDIERKKTEQLLNRMLPSSVAEKLKMGLAVDPEEFSDVTIYFSDIVGFTTIAAHCSPVQVVDLLNDLYTIFDATINAYNVYKVETIGDAYMVVSGLPVKIPDHAEQIATMALDLLHQSGRFNVKHLPGVPLQLRIGLHTGPCCAGVVGLTMPRYCLFGDTVNTASRMESTGSSWRIHMSQETRDRLDARGGYAIEPRGLIDIKGKGMMNTFWLLGKKGFDKPLPAPPPIGESHGLDESLIRNSITLKAQANKSRTSTNPSSSQSSSLAGESVEVKVEITPPTNADLASGTNLPNSYSLDSNSTNTISPNATLCPEFPGKTTPSSTSPQSRKLSELTPENLLNPNSFNRLPSSTGGSSSRLYKKIEEMMDLSSPYNHYKCLSPSESNLTQFYDGKYLYGSVAGGAGGGQAGGGACASIALSGSGCGGGASRFDSKPGSSRLLRRQFSLDRDDQQAKGEQQHHQHSLQANTYSGLVGGCGGGVKSSMLDIPLLHDTTRSPKGTLTRSHKQNSASITQDLEKIEEIPLSPASSQHHSSLDSNLNRSPPSTLEAQSPPLPPMSPPQLSAPTSPAPSRTLNGIYAHNSNSTSSHGAANNNNGTHPGPELTLNAEQLLSR

>DmCG11144

MKQKNNNGTILVVVMVLSWSRVVDLKSPSNTHTQDSVSVSLPGDIILGGLFPVHEKGEGAPCGPKVYNRGVQRLEAMLYAIDRVNNDPNILPGITIGVHILDTCSRDTYALNQSLQFVRASLNNLDTSGYECADGSSPQLRKNASSGPVFGVIGGSYSSVSLQVANLLRLFHIPQVSPASTAKTLSDKTRFDLFARTVPPDTFQSVALVDILKNFNWSYVSTIHSEGSYGEYGIEALHKEATERNVCIAVAEKVPSAADDKVFDSIISKLQKKPNARGVVLFTRAEDARRILQAAKRANLSQPFHWIASDGWGKQQKLLEGLEDIAEGAITVELQSEIIADFDRYMMQLTPETNQRNPWFAEYWEDTFNCVLTSLSVKPDTSNSANSTDNKIGVKAKTECDDSYRLSEKVGYEQESKTQFVVDAVYAFAYALHNLHNDRCNTQSDQTTETRKHLQSESVWYRKISTDTKSQACPDMANYDGKEFYNNYLLNVSFIDLAGSEVKFDRQGDGLARYDILNYQRQENSSGYQYKVIGKWFNGLQLNSETVVWNKETEQPTSACSLPCEVGMIKKQQGDTCCWICDSCESFEYVYDEFTCKDCGPGLWPYADKLSCYALDIQYMKWNSLFALIPMAIAIFGIALTSIVIVLFAKNHDTPLVRASGRELSYTLLFGILVCYCNTFALIAKPTIGSCVLQRFGIGVGFSIIYSALLTKTNRISRIFHSASKSAQRLKYISPQSQVVITTSLIAIQVLITMIWMVVEPPGTRFYYPDRREVILKCKIQDMSFLFSQLYNMILITICTIYAIKTRKIPENFNESKFIGFTMYTTCIIWLAFVPIYFGTGNSYEVQTTTLCISISLSASVALVCLYSPKVYILVFHPDKNVRKLTMNSTVYRRSAAAVAQGAPTSSGYSRTHAPGTSALTGGAVGTNASSSTLPTQNSPHLDEASAQTNVAHKTNGEFLPEVGERVEPICHIVNK
