# Supplementary material for: Identification of Neuropeptides and Their Receptors in the Ectoparasitoid, Habrobracon hebetor
Source: Front Physiol. 2020 Oct 16;11:575655. doi: 10.3389/fphys.2020.575655 (PMC7596734; doi:10.3389/fphys.2020.575655)
Supplement: Supplementary file 10 [file Table_7.DOC]

>DmCG11144

MKQKNNNGTILVVVMVLSWSRVVDLKSPSNTHTQDSVSVSLPGDIILGGLFPVHEKGEGAPCGPKVYNRGVQRLEAMLYAIDRVNNDPNILPGITIGVHILDTCSRDTYALNQSLQFVRASLNNLDTSGYECADGSSPQLRKNASSGPVFGVIGGSYSSVSLQVANLLRLFHIPQVSPASTAKTLSDKTRFDLFARTVPPDTFQSVALVDILKNFNWSYVSTIHSEGSYGEYGIEALHKEATERNVCIAVAEKVPSAADDKVFDSIISKLQKKPNARGVVLFTRAEDARRILQAAKRANLSQPFHWIASDGWGKQQKLLEGLEDIAEGAITVELQSEIIADFDRYMMQLTPETNQRNPWFAEYWEDTFNCVLTSLSVKPDTSNSANSTDNKIGVKAKTECDDSYRLSEKVGYEQESKTQFVVDAVYAFAYALHNLHNDRCNTQSDQTTETRKHLQSESVWYRKISTDTKSQACPDMANYDGKEFYNNYLLNVSFIDLAGSEVKFDRQGDGLARYDILNYQRQENSSGYQYKVIGKWFNGLQLNSETVVWNKETEQPTSACSLPCEVGMIKKQQGDTCCWICDSCESFEYVYDEFTCKDCGPGLWPYADKLSCYALDIQYMKWNSLFALIPMAIAIFGIALTSIVIVLFAKNHDTPLVRASGRELSYTLLFGILVCYCNTFALIAKPTIGSCVLQRFGIGVGFSIIYSALLTKTNRISRIFHSASKSAQRLKYISPQSQVVITTSLIAIQVLITMIWMVVEPPGTRFYYPDRREVILKCKIQDMSFLFSQLYNMILITICTIYAIKTRKIPENFNESKFIGFTMYTTCIIWLAFVPIYFGTGNSYEVQTTTLCISISLSASVALVCLYSPKVYILVFHPDKNVRKLTMNSTVYRRSAAAVAQGAPTSSGYSRTHAPGTSALTGGAVGTNASSSTLPTQNSPHLDEASAQTNVAHKTNGEFLPEVGERVEPICHIVNK

>Hheb037010.1

MRREMCQSIDIRNRVDQFSKLRGCRVVEGFVQILLIDHANETAYINQSFPELVEITGYLVLYRVSGLKSIGRLFPNLRVIRGHSLFINYALVAFEMMHLQEIGLHSLTDILRGSGNKPQNGCPVCDKKCPMRSMTHLKRYEERLCWNQEHCQLVCGTSSCLAKNTSSCCHESCLGSCEGTSAGHCLVCKDVSVDNMCVNKCPDNTYEFMNRRCVQEGECRRMRKPREALNTVKEYPYKPFNGSCIMECPAGYMEEEIDGKASCKKCKGICLKECSGINVESIATAQKLRGCTHIRGSLEIQIRGGKNIVKELEDNLNMIEVIDGYLKIVPVLDNQNLQELWDWSTHPKITIGAEGTEPKLFFHFNPKLCLQKIEELRIKAGLQPFTDLEVAPNSNGDKVACNVSKLHVRIHKRTAEAVLVGWEPFEHHDSRSLLGYVVYSIEAPEQNIAMYDGRDACGGDRWHVDDVASNEQKNDGNETRLQNHILTLLKPYTQYAFYVKTYTIATERSGAQSDLMYVTTMPGTPTPPRALTMWSNGSSELVIQWQPPMRANGELTHYNVYGRMEPDDAEFLQQRNYCNEPIHLPEKKSIATMAAEERERAEVERQLAKQPENPTCQCSGDKNFNEEDDREKEVSSSIAFEDALHNQVYIKRTINHRRKRHIDLSIPTDFETLYQQIFGDADKNEQCNDDDTCLAFKNKRETLDSQDNIEKELTDETIGNNTYMSFVKRVMVQNNSKDELPSITITGLRHFAAYNIEVQACRQRADGGKDLDLNPECSTKTMRTYHTLPLDGADDIPAGTFKLEMTGNSSQPVVQLQWAEPPEPNGIIVTYQIEYKRVDIQNIQAIPICITRTDFLRANKYYYLRGMTSGNYSIKVRATSLAGPGAYSEVKYFIIDASTTSSYVWIILGVFTSIILFSIVAFAFFCRRKIMGNVPNMRLIASVNPEYVSPTYVPDEWEVPREQIEVVRELGIGSFGMVYEGIIKDADKEDAEELRVAVKTVRKKASDHAKAEFLNEATVMKAFHTNHVVKLLGVVSCSQPTLVIMELMRNGDLKSYLRSHRPDNKDPKNQPTTEPPTLKRLMRMAVEIADGMAYLTSKKFVHRDLAARNVMIAEDMTVKIGDFGMTRDIYETDYYRKGTRGFLPVRWMAPESLKDGVFTNSSDVWSYGVVLWEMVTLASQPYPGLGNDMVLKYVISGGLMECPDNCPNSLYLLMRKTWHQKAHRRPTFIDITKILINNIHYEDFSQVSFYHSPGGIESRYLNVQHSTVKAKDLEISFDDLWEDYIEEEEQTENSPLRRDFGDFACIEPGRMPDCGDSHNGSEPCGESSKLVTNFHDLNSSEAPRKTVPIDSNGRSLNSSRDTLDCPFADSLRSSKNSPSIRHNNVSTTPHISPASASNSIPLTKRNNTSMGNFANNSSLSKKTHDYENHTPEGVATIENKDTVSIRITCPSVEDIEVDVINGDDRGEPSTVDHNNGTETLNGYIGNTAT

>Hheb003390.1

MGKNLMKTVVLGVLGIWWFLVICADGNSRLLRINNELATDKDTRKNESDRDLERRVERAIGDSSSNDGNLLMDSVPSQPGYTELTPIPETNSSEIKAEKKKIKGTKRFKDITVAPGGICRTVDVRNYLADLEILRPCRVIEGTLQVVLMENVDGNKTIEPFDQLREITGYLLFYRVANLKSIGQLFPNLEVIRGQQLLADNAFMVYEMATLQELALPNLVEISRGGVMIQKNPSLCYAETIDWDRIVRAGENSIRENNNNPSQCPVCTLNCPEGHCWSTRECQKMEKEECHPQCLGHCFGPTDRDCHVCRNFKLDGQCIPECPKHLYNYLERRCITAEECWAIEEPDINSLETVHLTPFAGMCKQTCPHGYEIGRDAKNMSTCVECKGKCQRSSLGVTIRRASEAQRLRGVVVIKGSLELQIRTGNSNLIMSELAASLGQLEEIMGYLKVTHSFPITSLSFLKNLRLIHGTKTDTNNASLIVLDNPNLSQLFPEDQEVKILSGKLFMHYNPKLCMSHIMRVVEKSGITNITNMEVEPESNGDKVACDIVDINITVSEKGPTYANLEWTAYKPATGQTLLTYLLNYVDTKYENITHDTNSCSGKQWQIIDVESDPESLDPVIKLKITDLKPYTKYAVYVKTMMTKDKNRAGSGTGQSRIIFFMTEPDTPDVPIDVTSFSVSESEIMVNWLPPERPNGPLGYYKIAGFLRPENPSVLHNRNYCDFPPELYVPDDVSEVTIKTPIVPKSCCDKDTSPSLSSHKFHIFCYDNMAISALPLNGRKQCDTQNHYGVNLMSPMSSIGKQSSLDYTHDVLDYQMTRMVNDTYYSFVFDVPSRNTSYLLKKLRHYSLYTIAVAACAEKRSNSGEMCSMFELTSAMTNKKDAADNIRKLEAQESNDTIVMLTWEPPIDPNGLTVAYTIEWVNLMIKDAKSTSECLPASLFHGYKIISNLSPGRYSARVRAISLAGEGPWSEGVTFTVGMDSSNISTIAMGITFFSIFGIVVFVFFLFRNHQKRKKQQRLIASVNPDYIESKYVKDSWEVPRENVMIIRQFGEGNFGTVFWGILNGEKPVAIKTPPRGSSEEGKNEFLNEASVMKKFSSHHIVRLVGVVSDGIPPYVIMELMENGDLKTYLRKLREAGQLSLDVPRIIRMASEIADGMAYLESKKFVHRDLAARNCMVSKDIVCKIGDFGMTRDIYETDYYKVGQKSMLPIRWMAPESLSDGVFTSDSDVWSYGIVLYEILSLAELPYQGLSNDEVMHHVMRKGTIDIDRDCPEVIQRVMEKCFKWRPFERPTFMEIISELEPFIGQDFCEKSFYHSEQGVEIRNSGAKKVYHQAAQIRFHWGNETARWIREFEDSAALLEPDKASTSRGKIFKNGFQQLGTEPIMEDVPLNR

>PPU05140-RA PTTH

MSCSSSRSLSLPVVFWLLLLLLLPPLQHVQALVLEDSLRLALCQARCPDDLKCIKECDSASNNVTNIPYLQTSSESNIHLQCRDANRLVLRHQAGIYVIETVDSFYNNWTSPTISTNCSHESANLMPEHRYRYRIRKVTSQGVSLPEITEWFQTNAETYVPKEVKNLQIGKINPNKNIPGHLQARLDVIPADDLNCQYNVVHWGGEHDLYQYQLDAAREYSLELRHLNYGRNNTVYVVSRNELGSRQSKNATLTFQTPPCLSVHRNLNVCAPAPVTGLHVVHQSNKNAVDFKVAWEKPELQPNNYTIIVQSLDFENSSVEVTVSGNETAVDIAKVVPSHQYKIAILAESEGGTSLEWKLITADNSGLTVESQVIIAISTLTTLVLVVVFGYTCIRYKRIKGHSSCRYSFFENINRKACGFHDEKGPLKSHLSPYESEDNSGSSKMRDKFEIQMDRLSMKKILGSGISGVVKLATLQEDKKKIIQVAVKMLREDASSDDVQNFHREIALMKSAGNHPNIVSIIGCCTLSKQPLLVVEFCSKGDLQTYLRTILEEMMTAVFKKVNWSTAQSNVPSTLQRESNNFSLAINNRLYDIQQESGMENSEINPEDLLNFARQVASGMEFLSSNRIVHRDLAARNVLVCADQTVKISDFGLSRDIYQENVYKKQTNGKLPIKWMAIEALTHQVYTTHSDVWSFGVLLYEIVTLGGNPYPGLPCCEILHFLKSGYRMERPSNCGFQLYEIMRSCWHENPLNRPTFYNLKQQLDKLLTTFAGADQDEP

>PPU00285-RA NP

MHYISGDADAAYQEDRTLAADFGFRPPADRMNSLQKECAAKRDARSAFNLNRHRAQMKLKMWGLRSVAILLILAFGIRAVNSNQCLTMLEEVNPKRTARLDGQTLNIQFEISRRVTQRLVSRVMRIFLQEILGYTDITLVEKEDEFNVTATFARLSDNLTNSRKSMIPESMVNMEVWIPPHLDTVPLLNKHDVLKVGVVAPPGHFGWFIPDRLSRLNDSWITFTKQETAARFDVDEVNLRKIINATMKTDNEYYCQESFCHEGMYIPPQCQSHKLKSQPCALLLADYSEATKFIKDHIDNWKLYVRVAWVGPNLKQIIKSLTKEYLQLTQNSALADRSLVILHWTPSNIIPNEREFASVEFPRCGSRGTSMGCQYETSKLEKLVWNGLESIAKLAFEAINRVQYTESMYENLISKYNEKLINKYNSYNKLSAMEQEVACDWLKENLNYTLDNWMPNDEDKNTLIIGGIFPMTGTFYTAKSIVLAASMARIAIDKNNTVLRDYNLKLLINDGQCKSDFVMKSFIDYILHNYYRKLIGVLGPACSETIEPLAGVSKHYYTVIMSYGAEGSSFSDRSRYPYFFRTIGENRQYKHVYLQLLKKFNWNRVAAFSEDGLKYTEYISYMQEMLRENGITFVANIKFPREWQPEILTKYLEDLKQKRARIIISDVYDQVARLVMCEAYRLEMTAAQGYVWFLPLWLRENWYNTDYYNQLGENTPCSTVEMMKAINGHLGISHATFAPDDSIMQEGITVREWRNNYEHRCAIQKEITSPYAGYTYDAMWTYAYAIDKLLKENQSYIFDLHSEHTTARFTDIISKTDFNGVSGRIKFVGGPSRFPVINIYQHVDGKSRLVGNFHPNISEELNQVIGGTLDLNMSAIVWLSGKKPDDGSEPPKKCVFSGFAELLNVSCEGAIVIVNIIGFGLLGIFLVIVVILIKRKYDQKVRLHEKYMKSLGIDLLQPDTSSLDKWEIPRDRVVINRKLGEGAFGMVYGGEAFFPEKGWLAVAVKTLKVGSSTEEKLNFLSEVEVMKRFEHKNIIKLLAVCIKCEPVLTVMEFMLYGDLKTYLLARRHLVNDQHYEDSDEISNKKLTAMALDVARALSYLAQLKYVHRDVASRNCLVNAQRIVKLGDFGMTRPMYENDYYKFNRKGMLPVRWMAPESLGLGIFTPASDVWSYGVLLYEIITFGSFPFQGMSNNQVLTHVKSGNSLTVPKGVKPQLEGLIRSCWSVEHTKRPTAPEIVDFLATNQRVIAPCLDVPLASVQLEHTGEMEMQLNTSTDRKFSFPWPGQSNKQNNKSPPTIDMPLVDINDSTSTKNQNIDSILGTSSTSEIESTKPLLGTDTTDSVPSNSQSVGSSRYVNLQPGVTSAFLDVPSSDSDNYGIAMSERRSMLPATNNFNNMPLL

>PPU07299-RA ILPs

MTTTTSSCNESTSCRCCCCSCGRELSDAVRLCPLCQRLQHDSEAGGDERRDRRRKNHHQQEDSGRRRRYDDNTEKDFRSTTTSTRRRRSASSVVRWVSDKCSRTKITRRTTGFWPGALSTALVLLILLLTLGRAVAENAKTAVEGTAPNSASGKVCQSIDIRNKLSEFSKLDGCHVIEGFLQIILLESTNEAHYENMSYPDLTEITGYFMVYRVQGLRSLGHLFPNLTVIRGHSLFMNYAFVAFEMTSLQEIGLNSLTDIMRGSVRFEKNPVLCYADTVDWDLIAHAGKGGNVISANRPKNECPVCEKDCPRRQTNLEERLCWNKQFCQKVCTSCQDKDGQDRTCTIGSRPDNKTTCCHRNCLGGCTGPTHLDCKVCRDVVVKQNECVSTCPADTYQFLNRRCITKRECQMMRKPREAPPDTRPYPFKPFNNECTMQCPPGYEEVAEQDMWSCKKCTGPCLKECSGMVVDSIAAAQKLRGCSHIRGNLEIAIREGQNIVHELEESLSNIEVINGYLKIFRSFPLISLSFLKNLVEIKGQELDMKDYSLVVLDNQNLQQLWNWTGRPPLKIGSKNPVPKVSFHYNPKLCIQTIEELRERTGLNPFREVEVSPTSNGDKVACNVTEIKTQVYTISSKAAMIKWEPFEHHDMRTLLGYVVYSKEAPFQNVSMYESRDACGGDGWKVDDVSVTDDDNNRRQKIDTTISEKNPDSTSYNVTNNRSSTYTQYTILAPLKPFTQYAYYVKTYTISTERSGAQSKLKYFKTLPGQPSQVRALTIYSNASDTLVISWLPPHESNSNLTHYEITGQMERYDLTFLRQRNYCIEPMQVLEYKPISEIAAEEKKRAEQELEASKTPEPSACECKKREPVQSELDVYSSIAFEDALHNQVYVKRYDNRRRRDVSYTSVMLKAVRMKRELTNYQEKDVISHSKKSLQNQSKTNSEQERTLEQQKVLNPDDDYIEDGAIVYFKRKVPANELNFVMRGLRHYGKYNIKVTACRDKAANVDELPCSSESMKSLQTLRLDHADEIPDGTLRLQKQANNDSTTSIKLTWDEPPRPNGVIVTYQIEYKRVDVQYLKAEVACTTRDKFNEDGNSYILQNLATGNYSVRVRATSFAGNGEYTEYKYFYVPESNYSTPTWKVILIVLLSISVILGACFIVAWFFKKKFMRNVPSMRLIATVNPEYVSTSYIPDEWEVPRKNIQLLKELGNGSFGMVYEGIAKDVIKGIPEMRCAVKTVTENASDKERVDFLNEASVMKGFNTHHVVRLLGVVSQGQPTLVVMELMMNGDLKSYLRSHRPDVCENFSRQPPTLNRIMRMALEIADGMAYLTAKKFVHRDLAARNCMVSDDLTVKIGDFGMTRDVYETDYYRKGTKGLLPVRWMAPESLKDGVFTSFSDVWSYGVVLWEMVTLASQPYQGLSNDQVLRYVIDGGVMERPENCPDILYGMMRQTWCHKATGRPSFIDIVRELMLELDTTEFEKVSFYHSPDGVEVRNQNPSQYRTDKELEMAMQESHEEEAEGGEESPLRQDFGDFSSFEPMTRRHNGSSGNFGMEPFTDNSKSPMSLALGFLDLNSSKAPLKAGFDDFDGVSVGSLASSKDTLNLPFAEDSVKPAKNSPFTEKKSSSKGNVSQSSAGKSSLSPQSPSIIATTCNLGSKPSPSLVSSTRSDPEYENRSPEVVDQPQQQPPTRDVASLRVSFPSMDAIDSDETAIPETTSSIVVDSNSSSATLPSAKQQYANKSGEQQAAPLNNGYIGGPAT

>NV17744-RA

MWGLRSVSILLILAFGIRVVNSNQCLTMLEEVNPKRTARLDGQTLNIQFEISRRVTQRLVSRVMSIFLQEILGYTDIALVAKEDEFNVTATFARLSENLTNSRKSMIPESMVNMEVWIPPHLDTVPLLSKHDVLKVGVVAPPGHFGWFIPDRLSRLNDSWITFTKQETAARFDVDEVNLRKIINATMKPDNEYYCQESFCHEGMYIPPQCQSHKLKSQPCALLLADYSEATKFIKDHIDNWKLYVRVAWVGPNLKQIIKSLTKEYLQLTQNSALADRSLVILHWTPSNIIPNEREFVSVEFPRCGSRGTSMGCQYETSKLEKLVWNGLESIAKLAFEAINRVQYTDSMYENLISKYNEKLINKYNSYSKLSAMEQEVACDWLKENLNYTLDNWMPNDEDKNTLIVGGIFPMTGTFYTAKSIVLAASMAKTAINRNNTVLRDYNLKMLINDGQCKSDFVMKSFIDYILHNFYRKLIGVLGPACSETIEPLAGVSKHYYTVIMSYGAEGSSFSDRSRYPYFFRTIGENRQYKHVYLQLLKKFNWNRVAAFSEDGLKYTEYISYMQEMLRENGITFVANIKFPREWQPEILTKYLEDLKQKRARIIISDVYDQVARLVMCEAYRLEMTAAQGYVWFLPLWLREDWYNTDYYNELGENTPCTTAEMMKAINGHLGISHATFAPDDSIMQEGITVREWRNNYEHYCAMQKELTSPYAGYTYDAMWTYAYAIDKLLKENQSYIFDLHSEHTTTRFTDIISKTDFNGVSGRIKFVGGPSRFPVINIYQHVDGKSRLVGNFHPNISEELNQVIGGTLDLNMSAIVWLSGKKPDDGSEPPKKCVFSGFAELLNVSCEGAIVIVNIIGFGLLGIFLVIVVIIIKRKYDQKVRLHEKYMKSLGLDLLQQDTSSLDKWEIPRDRVVINRKLGEGAFGMVYGGEAFFPEKGWLAVAVKTLKVGSSTEEKLNFLSEVEVMKRFEHKNIIKLLAVCIKCEPVLTVMEFMLYGDLKTYLLARRHLVNDQHYEDSDEISNKKLTAMALDVARALSYLAQLKYVHRDVASRNCLVNAQRIVKLGDFGMTRPMYENDYYKFNRKGMLPVRWMAPESLGLGIFTPASDVWSYGVLLYEIITFGSFPFQGMSNNQVLTHVKSGNSLTVPKGVKPQLEGLIRSCWSVEHTKRPTAPEIVDFLATNQRVIAPCLDVPLASVQLEHTGEMEMQLNTSTDRKFSFPWPGQSNKQNNKSSPTIDMPLVDINDSTSTKNQNIHSILGTSSTSEIESTKPLLGTDTTDSAPSSSQSAGLARYVNLQPGVTSAFLDVPSSDSDNYGIAMSERRSTLPATNNFNNMPLL

>Nv torso XP_008211645.2

MRCSSTWPSLCVPVLWLLQVRASLFEDSLRLALCQARCPDNLKCIEECHSASNNVTDIPYLQKSSESNVHLQCKDANRLVLKHRAGIYVIETLDSFYGNWTSPIISKNRFYESANLMPRHRYRYRIRKVHSQGVSLPEITEWFQTNAETYVPIAVKNLLIGEINPNKNRPGQLQARLDIIPADDLNCQYNVIHWGGEHDLYQYQLDAAREYSLELRHLNYGRNNTVYVVSRNELGSLQSENATLTFQTPSCLSVHRNLSACAPAPVTGLHVAYQSNKNAVDFKVAWEEAELQPNNYTIIVQPLDFENSSVEVTVSGNETTVDIPKVALSRQYKIAILAESEGGTSLEWKLITATTRLTVESQVIIAISTLTTLVLIVIFGYTYIRYKRMKGHSGCQYSFFENINRKACGFEDIKAPLKTLSPYEAEDNKSRDKFEIQVDRLSIKKILGSGISGVVKLATLQDDKKKIIQVAVKMLREDASSDDVQNFHREIALMKSAGNHPNIVSIIGCCTLSKQPLLVVEFCSKGDLQTYLRTILEEMMTAVFKKVNRSAESNGPQSILQRESNNFALAINNRLYDIQQESGVENKINPEDLLNFARQVASGMEFLSSNRIVHRDLAARNVLVCADQTVKISDFGLSRDIYQENVYKKQTNGKLPIKWMAIEALTHQVYTTHSDVWSFGVLLYEIVTLGGNPYPGLPCCEVLHFLKSGYRMERPSNCGFQLYEIMRSCWHENPLNRPTFYNLKQQLDKLLTTFTGNEYLNLSELLREPTTQHRYFLKHDRINSNILTIPHII

>NV14476-RA

MSNLHRSTMTTTTSGCIETSSCRCCCCSCGRELSDAARLCPLCQRLQHDSKACGDERRGSRRKNHHQREDSGRRRKYDDNTEKDFRSTTTSTRRRRSASSVVRWVSAKCSRTKIARTTGFWPGALSTGIVLLMLLLTLGRAVAENAKTAVEGTAPNHASGRVCQSIDIRNKLSEFSKLDGCRVVEGFLQILLLENTNEAHFENMSYPDLVEITGYFMVYRVQGLQSLGHLFPNLAVIRGHSLFMNYAFVAFEMTSLQEIGLASLTDIMRGSVRFEKNPVLCYANTVDWDLIAHAGKGGNVISSNRPKNECPVCEKDCPRRPTNLDERLCWNKQFCQKVCTSCQDKDGRDRTCTVGSRPDNKTICCHENCLGGCTGPSNLECKVCRDVVVNQNECVSSCPNGTYQFFNRRCITKRECQMMRRPREAPAETRAYPFKPFNNECLMDCPAGYEEVAEKDMWSCKKCTGPCLRECTGIVVDSIATAQKLRGCSHIKGNLEIAVREGQNIVHELEESLSNIEVITGYLKIFRSFPLISLSFLKNLVEIKGETLDMKDYSLVVLDNQNLQQLWNWTTRPPLKIGSSNPVPKVSFHYNPKLCLQTIEELRERTGLNPFREVEVSPTSNGDKVACNVTEIRTIVFSISSKAAMINWEPFEHHDMRTLLGYVLYSKEAPFQNVSMYESRDACGGDGWKVVDIGVNEEDKIKMKKIDTSISDSNTNNVTNNRSSTYTQYTILTQLKPFTQYAYYVKTYTISTERSGAQSKLKYFKTLPGQPSIVRALTIYSNASDTLVISWLPPTEANSNLTHYEITGQMERYDLTFLRQRNYCIEPMQVLEYKPIAEIAAEEKKRAEQELEASKIPEPSTCECKKRDPVQSESEVYSSITFEDALHNTVYVRRQDNRRRRDVSYTSVMLEAVRMKRELTNYQEKDVISHSKKSLQNQSKTNPEQERSLEQQKILNPDEDYIEDGAIVYFKRKVPANELNFVMRGLRHYGKYNIKVTACRDKKANAHELPCSSESMKSLQTLRLDHADEIPSGTLRLQKQANNDSTTSIKLTWDEPPRPNGVIVTYQIEYKRVDVQNLKAEVACTTRDKFNEDGNSYLLQNLATGNYSVRVRATSFAGNGEYTEYKYFDVPESNYSMPAWKVILIVLFSISFILGAFLVAAWFFKRKFMRNVPSMRLIATVNPEYVSTVYIPDEWEVPRKKVHLLKELGNGSFGMVYEGIAKDVVKGKPEMRCAVKTVNENATDRERVEFLNEASVMKGFNTHHVVRLLGVVSQGQPTLVVMELMVNGDLKTYLRSHRPDVCENFSRQPPTLNRIMRMALEIADGMAYLAAKKFVHRDLAARNCMVSDDLTVKIGDFGMTRDVYETDYYRKGTKGLLPVRWMAPESLKDGVFTSFSDVWSYGVVLWEMVTLASQPYQGLSNDQVLRYVIDGGVMERPENCPDILYGMMRQTWCHKATRRPTFIDIERELMLEVDTTEFEKVSFYHSPDGVEVRNQNPPSQYRTDKELEMVMQESREDEAEGGEDSPLRQDFGDFSSFEPMTRRKNGSSGHFGMEPFTDNSKSPTSLALGFLDLNSSKAPLKAGFDDFDGVSVGSLASSKDTLNLPFAEDSVKPAKNSPFAQKKSSSKGNVSQSSAGKSSLSPQSPSIIATTGNLGSKPSPSLVSSTRSDPEYENRSPEAVDQLQQQPPTRDVASLRVSFPSMDAIDSDETVIPETTSSIVVDSNSSSATLPSAKQQYANKSGEQQAAPLNNGYIGGPAT

>CG1389 torso

MLIFYAKYAFIFWFFVGSNQGEMLLMDKISHDKTLLNVTACTQNCLEKGQMDFRSCLKDCRINGTFPGALRKVQENYQMNMICRTESEIVFQIDWVQHSRGTEPAPNATYIIRVDAVKDDNKETALYLSDDNFLILPGLESNSTHNITALAMHGDGSYSLIAKDQTFATLIRGYQPSKMGAVNLLRFVPQPDDLHHIAAEIEWKPSAESNCYFDMVSYSTNSVNMDEPLEVQFRDRKKLYRHTVDNLEFDKQYHVGVRTVNIMNRLESDLQWLPIAVPSCLDWYPYNYTLCPPHKPENLTVTQKQYLPNILALNITWARPRYLPDNYTLHIFDLFKGGTELNYTLDQNRSHFYVPKITVLGSHFEVHLVAQSAGGKNVSGLTLDKVHREGNMVKLVLFIIVPICCILMLCSLTFCRRNRSEVQALQMDAKDAKASEFHLSLMDSSGLLVTLSANESLEVMDELEVEPHSVLLQDVLGEGAFGLVRRGVYKKRQVAVKLLKDEPNDEDVYAFKCEIQMLKAVGKHPNIVGIVGYSTRFSNQMMLLIEYCSLGSLQNFLREEWKFRQEQNAIGLKKNLEQNVDNRRFNRLPRNSIHDRIEDINNSMLSTVEEESESDQTHSSRCETYTLTRITNAADNKGYGLEDIENIGGSYIPKTAEAPKDRPKRKLKPQPKKDSKQDFKSDNKKRIFENKEYFDCLDSSDTKPRIPLKYADLLDIAQQVAVGMEFLAQNKVVHRDLAARNVLISVDRSIKIADFGLSRDVYHENVYRKSGGSGKLPIKWLALESLTHQVYTSQSDVWSFGVLLYEITTLGGMPYPSVSPSDLLQLLRQGHRMKRPEGCTQEMFSLMESCWSSVPSHRPTFSALKHRLGGMILATNDVPERLKQLQAATESKLKSCDGLNSKVEQVPCEEELYLEPLN

>AGAP005763-PA

SNASLVKIENLAPNRRYNVTATMLTSEYEYYYVEKHQYRTLPHDYMPGIVTDIMVERFETNARDSRLVDAVISWTPAKDRTCHYEIVCHASYSPDFLLKPIDVQQPEVLYKYTIKALKLSANYMIAVRSKNTQNAMRESQLHWQSFHTPSCTNHTNGTQVCAPEPITNVRVTQVPLYGDHYQLNISWDRPTIAPDSYVVKVFDLHNPETEEPGNSVTKNLTGDAVGVLIESFEMFGPHFEVLVAAYSRVGVSSENTIKALQIGRVSSESWIRTKLVFIILTPVLMIGLLKISISLICRRRAKLKRYEERCEYFKELEQKAPVDPSTEFEPTSKQMQDLLATSHPFSPDLIAPINDELEIEMEHIKLHDMLGEGAFGLVRKGVLQRTGDDDEPAQPVAVKMLKECPRVEDILEFRREMEVMKSVGTHPHIVGIVGHCTKNVRKMMLLTEYCGRGNLLNYLRLQWQRLLRQNRTTGTRSIGGTSGTNITATLPSPPPPPPPPPTRSLEMAEHECLTPSLDNNKMPENVFNFDTSFVNDKKSLTYKNISDHRQTLLGVAFGLAGGSDGSSASRSPQIIENKLYPLFSDESDGDETAQLTHCTNACKNAVEIVEGTWEVGEDVKGSVRIKPCSCHGAPVDSTLTNNTVENRWYQSCSPQSEEKEDPLPNSGQLLEFSRQIALGMEFLARNKVVHRDLAARNVLVCDSNTVKIADFGLSRDIYQENLYRKTSNGKLPIKWLALESMTHQVYTSQSDVWSYGILLYEICTLGGSPYPAISTNRLLRYLESGYRMERPKSCSELLYDLMYSCWNLHPGERPTFSKIVHTVEQLQARDTANDPVVIDLSAIVDSHCTKNTTEENSYLKPVEY

>AAEL002404-PA

MRNVSGDATALLIDSLAIHGARYELVISAYANNRSSHTTNINDAPIWRSPTDHWHGGRLAVIILTPVLTLGLMKIFISIICKRRAKVKRYEQRCEYFKELEQKAPIDPSTGFEIKVKNIQEIVHPATFPSDLIAPINDEMEISVDQIRLLDLVGEGAFGRVRKGILLHPVGTYTEVAVKMLKECPSLEDVKEFRREIEVMKSVGVHPNIVCIIGHYTQNVNEMMLLTEYCSEGNLLNFLRSEWHKVLQNRDRSASTTKQSLKALTPKDDVFEGCRSPSLECNKKPENVFNFDAPFIHDKKSFAYKNISDHNTPEPTESAPEMKITENRLYPLLNDTFDNNFSLCAEVESNDLKDNTKICTNSCKCNVEILQSNSSEDIETWTKRTPCSIKVSGCECDSISNGSGAQSKQDEICNMVNNQCYYKELCSEKRNQSEEYIITSRQLLEFAKQIAIGMEFLARNKVVHRDLAARNVLVCYDKAVKISDFGLSRDIYQQNLYRKTGTGKLPIKWLALESLTHQVYTSQSDVWSYGILLYEICTLGGNPYPLLSTCDLIMELKRGYRMEKPDSCSKELYELMLSCWSALPIDRPTFTSIHNRMEELMFQNMKKDMIDLDAIIDIQSTKTSSSEHSYLKPVEY

>AAEL001915

MTAENGYIWFLPVWLTNLWNLSNDSPIRSMVRCTRQEMLKAINGHFSLAHAPFADSHSSLDTIEGTVGKWRSDYRETLRRHSYMESDYAGYAYDAVWVYALALDRLIREDPSYLSDLHSIKTTKRLMEVIRATDFQGVSGRIKFGDEGSRYTIINVLQWINGTPNIVGQFTPNISESKYKLLGGSLALNQSAIVWMTKDGKTPEDGALDCTLSGLARFFGMGCDGTVYVLVGCLCVMTIAIISLASFCYFQVRYDRKMKHSAKYLQKFGIDLLSPSSIPVNTLDKWEVPKDRVVINRRLGEGAFGTVYGGEAQIGDEGWTAVAVKTLKIGSTTEDKVDFLSEAEAMKRFDHNNIVKLLGVCLQTEPVYTIMEFMLYGDLKTYLLARRHLVNSKQSEDSDISPKRLTMMALDVSRALSYLAEQKYVHRDLACRNCMVNAQRVVKLGDFGMARPTFENDYYRFNRKGMLPVRWMAPESLALGIFTPASDVWSYGVLLYEIISFGSFPYQGMTNNQVLEHVKEGNCLTIPTGVKPQLEGLMKACWNQDYKKRPSASEVSEFIANYPRLLSPCLDVPLASVQMAETDSDQFELLPGLRRRKDEPTADLLLGTSQMNDLNQTTTGYTKMNMRRGINLNDLNVDTDFRRNTLPNAETTTTTTNGGVTLNMYNPVEPLLQRQPEVAKSSNNILRYVPMFGLGRNKAPVLITHGNGSVTIGSRSTSTSVL

>CG18402 InR

MFNMPRGVTKSKSKRGKIKMENDMAAAATTTACTLGHICVLCRQEMLLDTCCCRQAVEAVDSPASSEEAYSSSNSSSCQASSEISAEEVWFLSHDDIVLCRRPKFDEVETTGKKRDVKCSGHQCSNECDDGSTKNNRQQRENFNIFSNCHNILRTLQSLLLLMFNCGIFNKRRRRQHQQQHHHHYQHHHQQHHQQHHQRQQANVSYTKFLLLLQTLAAATTRLSLSPKNYKQQQQLQHNQQLPRATPQQKQQEKDRHKCFHYKHNYSYSPGISLLLFILLANTLAIQAVVLPAHQQHLLHNDIADGLDKTALSVSGTQSRWTRSESNPTMRLSQNVKPCKSMDIRNMVSHFNQLENCTVIEGFLLIDLINDASPLNRSFPKLTEVTDYIIIYRVTGLHSLSKIFPNLSVIRGNKLFDGYALVVYSNFDLMDLGLHKLRSITRGGVRIEKNHKLCYDRTIDWLEILAENETQLVVLTENGKEKECRLSKCPGEIRIEEGHDTTAIEGELNASCQLHNNRRLCWNSKLCQTKCPEKCRNNCIDEHTCCSQDCLGGCVIDKNGNESCISCRNVSFNNICMDSCPKGYYQFDSRCVTANECITLTKFETNSVYSGIPYNGQCITHCPTGYQKSENKRMCEPCPGGKCDKECSSGLIDSLERAREFHGCTIITGTEPLTISIKRESGAHVMDELKYGLAAVHKIQSSLMVHLTYGLKSLKFFQSLTEISGDPPMDADKYALYVLDNRDLDELWGPNQTVFIRKGGVFFHFNPKLCVSTINQLLPMLASKPKFFEKSDVGADSNGNRGSCGTAVLNVTLQSVGANSAMLNVTTKVEIGEPQKPSNATIVFKDPRAFIGFVFYHMIDPYGNSTKSSDDPCDDRWKVSSPEKSGVMVLSNLIPYTNYSYYVRTMAISSELTNAESDVKNFRTNPGRPSKVTEVVATAISDSKINVTWSYLDKPYGVLTRYFIKAKLINRPTRNNNRDYCTEPLVKAMENDLPATTPTKKISDPLAGDCKCVEGSKKTSSQEYDDRKVQAGMEFENALQNFIFVPNIRKSKNGSSDKSDGAEGAALDSNAIPNGGATNPSRRRRDVALEPELDDVEGSVLLRHVRSITDDTDAFFEKDDENTYKDEEDLSSNKQFYEVFAKELPPNQTHFVFEKLRHFTRYAIFVVACREEIPSEKLRDTSFKKSLCSDYDTVFQTTKRKKFADIVMDLKVDLEHANNTESPVRVRWTPPVDPNGEIVTYEVAYKLQKPDQVEEKKCIPAADFNQTAGYLIKLNEGLYSFRVRANSIAGYGDFTEVEHIKVEPPPSYAKVFFWLLGIGLAFLIVSLFGYVCYLHKRKVPSNDLHMNTEVNPFYASMQYIPDDWEVLRENIIQLAPLGQGSFGMVYEGILKSFPPNGVDRECAIKTVNENATDRERTNFLSEASVMKEFDTYHVVRLLGVCSRGQPALVVMELMKKGDLKSYLRAHRPEERDEAMMTYLNRIGVTGNVQPPTYGRIYQMAIEIADGMAYLAAKKFVHRDLAARNCMVADDLTVKIGDFGMTRDIYETDYYRKGTKGLLPVRWMPPESLRDGVYSSASDVFSFGVVLWEMATLAAQPYQGLSNEQVLRYVIDGGVMERPENCPDFLHKLMQRCWHHRSSARPSFLDIIAYLEPQCPNSQFKEVSFYHSEAGLQHREKERKERNQLDAFAAVPLDQDLQDREQQEDATTPLRMGDYQQNSSLDQPPESPIAMVDDQGSHLPFSLPSGFIASSTPDGQTVMATAFQNIPAAQGDISATYVVPDADALDGDRGYEIYDPSPKCAELPTSRSGSTGGGKLSGEQHLLPRKGRQPTIMSSSMPDDVIGGSSLQPSTASAGSSNASSHTGRPSLKKTVADSVRNKANFINRHLFNHKRTGSNASHKSNASNAPSTSSNTNLTSHPVAMGNLGTIESGGSGSAGSYTGTPRFYTPSATPGGGSGMAISDNPNYRLLDESIASEQATILTTSSPNPNYEMMHPPTSLVSTNPNYMPMNETPVQMAGVTISHNPNYQPMQAPLNARQSQSSSDEDNEQEEDDEDEDDDVDDEHVEHIKMERMPLSRPRQRALPSKTQPPRSRSVSQTRKSPTNPNSGIGATGAGNRSNLLKENWLRPASTPRPPPPNGFIGREA

>AGAP012424-PA

MCLLLRGRAELCQSAQSLYKKCVYFCSSVDVRNTPLHLDRLRNCRVVEGFVQIMLIDKYGNDSFDNYTFPLLTEITGYLLLFRVNGLQTLGQLFPNLTVIRGSELANNYALVVYELMHIKELGLTSLIDIQRGGVRIEKNPNLCHADTIDWKAIAPYGENWIKGNQDGNECTTCPSNVTVSLPSGITQTIRCPLRDVNRLIHNEKASHLCWSTNHCQQKCPAHCPKSCNKTGECCSTSCLGQCSSNNKSHCMVCRKYYYIHNNQTRCVDKCPDHMFLFSESRCLTEEECYKIYKPLQRIADITDNYPYVPAQGECRLDCPLGYTLTRATGSQRLACVPCKGPCRSECKGMVIESISQMQQLRGCTIIQGSLSIRLRQLGGENVVRELEKVLYSIEEIYGYLTIVRSYALMSLGFFRNLKIIHGTVLNANLSLSVIDNQNLQELWNQNVTIKRGNVRFNDNPMLCVKKITSLKSHFDEGVGIENEEQLNKTNGVRVACEIKKLNTHPTKISLELAVIQWDAFKDLPDMRQLLGYVVYYIEAPHENVTFYDGRDACNSQGWRVDDVAYIQENDDATHILTKLQAFTQYAYYVKTYTLSSENLGGQTDITYFRTSPGTPRIVKDLHVYIDNDTLIVSWQEPTKINGKLSAYRIGATLNDERNEMIRQRNYCHDGMIYSFTSIATTVVPAKDNQNQCSKEECEAFCKTSSISESDSGPKIDINEVEMSIGFEDWLHNYVYIKNPKSSRKRREDGSSSYYRSLGATTNQTRITFPLSHFKHFALYSFRVLACREPAAKVPGVVVQDMADACGPEAMFIYRTPTKPEADDIPIDSIELDDQSNHTQRVIRVRWKGPSKPNGVLVSYSIKYHRTDLDSVQPTVRCVTVDVHTLLGYALLTKLEAGNYSVRIMATTTAGNGPYSPPKFLYLEKRDTDSTVVTWWVITATILIIMVLVIGVVYYLKHNYIPMSNMRLFAQVNPDYAGVTYKVDEWEVPREHIIQLEELGQGSFGMVYKGIMTKLGNDVNVPCAIKTVTENATERERDSFLIEATIMKEFHTHHVVRLYGVVSVGQPTLVIMELMANGDLKSYLRRHRPDYENGEESSPQPPTLKQIYQMAIEIADGMAYLAAKKFVHRDLAARNCMVAEDLTVKIGDFGMTRDIYETDYYRKGTKGFLPVRWMAPESLKDGMFSSSSDVFSYGVVLWEMATLASQPYQGLTNDQVLRYVIDGGVMERPENCPDKLYELMRICWQHRASARPSFIDIIRMLLPDANDNFKRVSFFFSPDAMDNSIQPGNGNISPNIFRNLINFIVFDFVFHFIYRIVR

>AAEL002317-PA

MALSGQNMHNLGVCGSVDVRNSPAHLDRLKDCVVVEGFVHILLIDKYIDSSFENYSFPLLTEITEYLLLFRVNGLKSLRRLFPNLAVIRGDALVGDYAMVIYELMHIEEIGLISLMDITRGGVRIEKNPKLCFANTIDWKAMTVPGTNNYIKDNQKDNVCPICPAESTAVMLPNGSKQKCPAAPVRGGNKDHKRTLCWNANHCQTICPPECPKACSKTGVCCDAESCLGGCNLPNTSSCSVCRHLSIDPAGKRQCVAKCPPNTFKYHTRCVTRDECYAMKKPISLDSNPDLPDQPFIPHNGSCLMECPLDHELITELNKTRWCRKCSGTCPKRCEGSNIDNIQSAQLLKGCEIIDGSLEIQLRSRGGENIVKELENFLSSITEIKGYLKVVRSYPLLSLGFLKKLKIIHGKGNKVSNSSLYVVENQNLQELFDHNVTIGEGKLFFFNNPMLCTDRIKAVKKYNPGIEIENESQLESNNGDRAACSITELETSLKSIGSETAIIQWAPFTELSDARMLLGYVIYYIEAPYANVTFFDGRDACNTEGWRLDDISDFNMDKETTKILTQLKPYTQYAYYVKTYTLGSEGLGGQSKIKYFTTAPGTPSVVRDVEVSVNKNMLTVKWLPPLKMNGRLKEYEVFIELNADDNEQLMLRDYCEDDKLRDIVPETPTSAPPPKTSICTADQCRNYCKAPTSGGSTGTIDVTDKENQITFEDQLHNYVYIKNPLLRDKTTRRKRSTNLLFPNNTENKKNDTTDRRTEKVKDEPYYQYIFNATNETSITFPLSYFNHYSLYVFKIRACRHPGDPPAPSVRLVDVELACGNEVFENFRTPKKEGADDIPPESILIEEQSNNTQRQIRVQWKEPSKPNGPIVKFVVKYQRVDLESVSSTDICIRYSSFNQTRGALLTKLEPGNYSIRVMATTIAGDGAPSAARYVLIAKDDSMGTTLIWLGTLIVIFLCSVGFVAFYWYKYRYMSKQIRMYPEVNPDYAGVQYKVDDWEVERNHIIQLEELGQGSFGMVYKGILTQLRGEKCNQPCAIKTVNESATAREKDSFLLEASVMKQFNTHHVVRLLGVVSQGDPTLVIMELMANGDLKSYLRRHRPDYENGEDPSPQPPTLRQIIQMAIEIADGMAYLSAKKFVHRDLAARNCMVADDMTVKIGDFGMTRDIYETDYYRKGTKGFLPVRWMAPESLKDGIFSSSSDVFSYGVVLWEMATLASQPYQGLTNDQVLRYVIDGGVMERPENCPDNLYNLMRRCWQHRPTARPTFMEIISELLPDASPHFQDVAFYNSQDALDMLRGQHQTVIIDEATTPLRPGDDHDEEPGEDDDLVGHGEGHIGDVGTDDEFSMEMTNSHLVRNNGPMATIRSPHSPLR

>CG7223 Fibroblast growth factor

MAAAWSWRASHSTITMTSGSLVVLFLLLSIWQPAVQVEGRRQMANSQEMIKDHLGARSQNKTPAITNNANQSSTSSADLDDGAADDDDNKADLPVNVSSKPYWRNPKKMSFLQTRPSGSLLTLNCHALGNPEPNITWYRNGTVDWTRGYGSLKRNRWTLTMEDLVPGDCGNYTCKVCNSLGCIRHDTQVIVSDRVNHKPILMTGPLNLTLVVNSTGSMHCKYLSDLTSKKAWIFVPCHGMTNCSNNRSIIAEDKDQLDFVNVRMEQEGWYTCVESNSLGQSNSTAYLRVVRSLHVLEAGVASGSLHSTSFVYIFVFGGLIFIFMTTLFVFYAIRKMKHEKVLKQRIETVHQWTKKVIIFKPEGGGDSSGSMDTMIMPVVRIQKQRTTVLQNGNEPAPFNEYEFPLDSNWELPRSHLVLGATLGEGAFGRVVMAEVNNAIVAVKMVKEGHTDDDIASLVREMEVMKIIGRHINIINLLGCCSQNGPLYVIVEYAPHGNLKDFLYKNRPFGRDQDRDSSQPPPSPPAHVITEKDLIKFAHQIARGMDYLASRRCIHRDLAARNVLVSDDYVLKIADFGLARDIQSTDYYRKNTNGRLPIKWMAPESLQEKFYDSKSDVWSYGILLWEIMTYGQQPYPTIMSAEELYTYLMSGQRMEKPAKCSMNIYILMRQCWHFNADDRPPFTEIVEYMDKLLQTKEDYLDVDIANLDTPPSTSDEEEDETDNLQKWCNY

>AGAP003108-PA

MVSLHQKLRFERVRKTDAGYYSCGSEFHEWSNLTLSVFSQETDQYQSDGAGMANLERKSLHSSSVAAAAAATPILDNELKEGPPRILGTRDEPYPSPVVRGVGEAYRLKCDAVGQPAPHVSWLKDGAEYRDNSYKSTIVFKQLLPTDAGTYVCNVCNVYGCVNATTVLEVVESGEHEPAYIQHNTMESHSQLQGDPATQDTQVMMRALGRYYPAGATEKRPQHHTYQPEDADMDEDEDEDGEEEEEEEGPTNGTGVHGSDTTEDSAGAVNGSGTPPPDRAPIFTKKDQMTKIVSKPSGNMVRLRCPADGYPKPNITWTKDGRKIERAMGQVKKVNWAIVLEDLVPQDSGSYTCAVCNQVGCINFTTKLEVKDRFPARPHITERPKNVTALVNTTVIFSCPILSDLEPHIEWVKLGLVDLENMSIPENVTKLKRDPDNPEVLTLENVTHADEGWYTCIAANSLGATNESAYLQVLDELPPDDTPTAHPVRTHSTLIMGMTIFLCACFTVLAVIVIIVCKKLKREKMKHRAMEHVNQWTKKVIVLKQPVVESSIPGMSEALQMPIVRIEKQRSTLVQSGNCDPTMISEYEFPIDLNWEFPRNKLHLGKSLGEGMFGKVVMAEAHGLVKGHPSTVVAVKMLKEGHTDADVKDLVCEMEVMKMIGKHVNIINLLGCCCKDGPLYVIVEYAPHGNLKNFLRSHRFGSNYEATNEKEKKILTQKELISFAYQIARGMEHLASRRCIHRDLAARNILVSDNYVMKIADFGLARDIHDQEYYRKTTTGKLPIRWMAPESLEEKFYDSQSDVWSFGVLLWEIMTLGGNPYPSIPTWDNLLEHLKKGKRMEKPPLCSIEIYLFMRECWHYRPEERPTFSEIVQHLDRLVSITSNEEYLDLGLPQLETPPSSDDSDDDVEEDEEDCDDQAGTEHERVHMYRFNRSYNNDCIY

>AAEL002172-PA

MVSGFVQGLFSIELSIKYHHAYHAIHEVMSFTKISSFNSSHFRLKFYPVKKTDAGYYSCGSEYGEWSNLTLSVISQHEASDHYQNDSPGSGLGPASSTLTRVANLEKKSLLPALPLSQPSPSPAAPLDVVDNDLTDSSYPTVNGGSMITVTKQVGENYKLRCNGTAEPNSQISWIKDGEVVSNTKNRPNLMLHSLIVDDAGVYICRVCHAQGCVNSTTILEVIDDPKESYIHYKNMEIHSQVSDDEEPDDPDYVDDEHDDYEDAEEDEEVDEEISATGSQQGSADGEFTESQSAPPRANGQLPPGPPVFTKEEHMVKLMPKPSGNMVRLRCPADGNPVPNITWTKDDEKIVRSMGSVKYAKWSIVLEDLVPKDSGKYTCHVCNSHGCINFTTKLEVKDRVNHKPILTKPLTNITAVVSTNVSMECKVLSDLSMYIQWFKFKGLCHDCAIIENKVMDMSNSDNPEVLTLENVTYADEGWYTCVAANTLGASYESAYLRVVDEFLVDPPIVHPVRPHSTLITIMTTVLSGCFMILAVIVVIVCKKLKREKMKHRAMEHVNQWTKKVIVLKQPVIENSIPGVTESMQMPIVRIEKQRSTLVQSGNCDPTMISEYEFPIDLNWEFPRSKLVLGKSLGEGAFGKVVMAEANGLVKGQASTVVAVKMLKEGHTDADVKDLVCEMEVMKMIGKHVNIINLLGCCCKDGPLYVIVEYAPHGNLKDFLRSHRFGTANYEDMISGEKEKKILTQKELISFAYQIARGMEHLASRRCIHRDLAARNVLVSDGYVMKIADFGLARDIHSQEYYRKTTTGKLPIRWMAPESLEEKFYDSQSDVWSFGVLLWEIMTLGGNPYSSIPTWDNLLEHLKKGKRLEQPPLCSIDIYLFMRECWHYRPEERPTFSEIVQHLDRLVSITSNEEYLDLGLPLLETPPSSDDESEDNDNDGCERVRMYPFNHHSHTTESTF

>AAEL004932-PA

MLLKFYPVKKTDAGYYSCGSEYGEWSNLTLSVISQHEASDHYQNDSPGSGLGPASSTLTRMANLEKKSLLPALPLSQPSPSPAAPLDVVDNDLTDSSYPTVNGGSMVTVTKQVGENYKLRCNGTAEPNSQISWIKDGEVVSNTKNRPNLMLHSLIVDDAGVYICRVCHAQGCVNSTTILEVIDDPKESYIHYKNMEIHSQVSDDEEPDDPDYVDDEHDDYEDAEEDEEVDEEISATGSQQGSADGEFTESQSAPPRANGQLPPGPPVFTKEEHMVKLMPKPSGNMVRLRCPADGNPVPNITWTKDDEKIVRSMGSVKYAKWSIVLEDLVPKDSGKYTCHVCNSHGCINFTTKLEVKDRVNHKPILTKPLTNITAVVGTNVSMECKVLSDLSMYIQWFKFKGLCHDCAIIENKVMDMSNSDNPEVLTLENVTYADEGWYTCVAANTLGASYESAYLRVVDEFLVDPPIVHPVRPHSTLITIMTTVLSGCFMILAVIVVIVCKKLKREKMKHRAMEHVNQWTKKVIVLKQPVIENSIPGVTESMQMPIVRIEKQRSTLVQSGNCDPTMISEYEFPIDLNWEFPRSKLVLGKSLGEGAFGKVVMAEANGLVKGQASTVVAVKMLKEGHTDADVKDLVCEMEVMKMIGKHVNIINLLGCCCKDGPLYVIVEYAPHGNLKDFLRSHRFGTANYEDMISGEKEKKILTQKELISFAYQIARGMEHLASRRCIHRDLAARNVLVSDGYVMKIADFGLARDIHSQEYYRKTTTGKLPIRWMAPESLEEKFYDSQSDVWSFGVLLWEIMTLGGNPYSSIPTWDNLLEHLKKGKRLEQPPLCSIDIYLFMRECWHYRPEERPTFSEIVQHLDRLVSITSNEDI
