# Supplementary material for: Identification of Neuropeptides and Their Receptors in the Ectoparasitoid, Habrobracon hebetor
Source: Front Physiol. 2020 Oct 16;11:575655. doi: 10.3389/fphys.2020.575655 (PMC7596734; doi:10.3389/fphys.2020.575655)
Supplement: Supplementary file 11 [file Table_8.docx]

**Table S1. Neuropeptide genes in indicated insect species^1^**

| **Peptide** | **Hh** | **Pp** | **Nv** | **Am** | **Dm** | **Bm** | **Tc** | **Nl** | **Cs** |
| --- | --- | --- | --- | --- | --- | --- | --- | --- | --- |
| AKH1 | + | + | + | + | + | + | + | + | + |
| AKH2 | nd | nd | nd | + | nd | + | + | + | + |
| ACP | nd | + | + | + | nd | + | + | nd | + |
| AstA | + | + | + | + | + | + | nd | + | + |
| AstB | nd | nd | nd | nd | + | + | + | + | + |
| AstC | nd | nd | nd | nd | + | + | + | nd | + |
| AstCC | + | + | + | + | + | + | + | + | + |
| AstCCb | nd | nd | nd | nd | nd | nd | nd | nd | + |
| AstCCC | + | + | + | + | nd | nd | nd | + | nd |
| AT | nd | + | + | + | nd | + | + | + | + |
| inotocin | nd | + | + | nd | nd | nd | + | + | nd |
| Burα | + | + | + | + | + | + | + | + | + |
| Burβ | + | + | + | + | + | + | + | + | + |
| CAPA | + | + | - | + | + | + | + | + | + |
| CCHa1 | nd | + | + | + | + | + | + | + | + |
| CCHa 2 | nd | + | + | + | + | + | + | + | + |
| CNMa | + | + | + | + | + | nd | + | + | nd |
| Crz | + | + | + | + | + | + | nd | + | + |
| CCAP | + | + | + | + | + | + | + | + | + |
| DH31 | + | + | + | + | + | + | + | + | + |
| DH44 | + | + | + | + | + | + | + | + | + |
| DH34 | nd | nd | nd | nd | nd | + | + | nd | + |
| DH45 | nd | nd | nd | nd | nd | + | nd | nd | + |
| Ele | + | nd | nd | + | nd | nd | nd | + | nd |
| ETH | + | + | + | + | + | + | + | + | + |
| EH | + | + | + | + | + | + | + | + | + |
| FMRF | + | nd | nd | + | + | + | + | + | + |
| GPA2 | nd | nd | nd | nd | + | + | + | + | + |
| GPB5 | nd | nd | nd | nd | + | + | + | + | + |
| Hugin-PK2 | + | + | + | + | + | + | + | + | + |
| IMF | nd | nd | nd | nd | nd | + | nd | nd | + |
| ILP | + | + | + | + | + | + | + | + | + |
| ITP | + | + | + | + | + | + | + | + | + |
| ITPL | nd | nd | nd | + | + | + | + | + | + |
| LK | + | nd | nd | + | + | + | nd | + | + |
| MS | + | + | + | + | + | + | + | + | + |
| NTL | + | nd | nd | + | + | + | + | + | + |
| NP | + | + | + | + | nd | + | + | + | + |
| NPF1 | nd | nd | nd | nd | + | + | nd | + | + |
| NPF1b | nd | nd | nd | nd | + | + | nd | + | + |
| NPF2 | nd | + | + | + | nd | + | nd | + | + |
| NPLP1 | + | nd | nd | + | + | + | + | + | + |
| NPLP2 | nd | nd | nd | + | + | nd | nd | nd | nd |
| NPLP3 | nd | nd | nd | + | + | nd | nd | + | nd |
| NPLP4 | nd | nd | nd | nd | + | nd | nd | + | nd |
| NVP | + | + | + | + | nd | nd | + | nd | nd |
| OKA | + | + | + | + | + | + | + | + | + |
| OKB | nd | nd | nd | + | + | + | + | + | + |
| PDF | nd | + | + | + | + | + | nd | + | + |
| Pro | + | nd | nd | nd | + | nd | + | + | nd |
| PTTH | + | + | + | nd | + | + | + | + | + |
| RY | + | + | + | + | + | + | + | + | + |
| sNPF | + | + | + | + | + | + | + | + | + |
| sNPFb | nd | nd | nd | nd | nd | nd | nd | nd | + |
| SIF | + | + | + | + | + | + | + | + | + |
| SK | nd | nd | nd | + | + | + | + | + | + |
| SP | nd | nd | nd | nd | + | nd | nd | nd | nd |
| TK | + | + | + | + | + | + | + | + | + |
| TR | + | nd | nd | nd | + | + | + | nd | + |

Abbreviations: nd, not identified; +, identified. ^1^The data of other insects are from *Pteromalus puparum* (Xu et al., 2020), *Nasonia vitripennis* (Hauser et al., 2010), *Apis mellifera* (Hummon et al., 2006), *Drosophila melanogaster* (Hewes and Taghert, 2001), *Bombyx mori* (Roller et al., 2008), *Tribolium castaneum* (Li et al., 2008), *Nilaparvata lugens* (Tanaka et al., 2014), *Chilo suppressalis* (Xu et al., 2016).
